# Prediction of adverse maternal and perinatal outcomes associated with pre-eclampsia and hypertensive disorders of pregnancy: a systematic review and meta-analysis

**DOI:** 10.1016/j.eclinm.2024.102861

**Published:** 2024-09-27

**Authors:** Valentina Bucher, Alexandra Roddy Mitchell, Pia Gudmundsson, Jessica Atkinson, Nicole Wallin, Joline Asp, Maria Sennström, Karin Hildén, Camilla Edvinsson, Joakim Ek, Roxanne Hastie, Catherine Cluver, Lina Bergman

**Affiliations:** a Department of Obstetrics and Gynecology, Institute of Clinical Sciences, Sahlgrenska Academy, University of Gothenburg, Gothenburg, Sweden; b Translational Obstetrics Group, Department of Obstetrics and Gynaecology, Mercy Hospital for Women, University of Melbourne, Melbourne, Australia; c Region Västra Götaland, Sahlgrenska University Hospital, Department of Obstetrics and Gynecology, Gothenburg, Sweden; d Department of Women’s and Children’s Health, Clinical Obstetrics, Uppsala University, Uppsala, Sweden; e Department of Women's and Children's Health, Division for Obstetrics and Gynecology, Karolinska University Hospital, Karolinska Institute, Stockholm, Sweden; f Department of Obstetrics and Gynaecology, Faculty of Medicine and Health, Örebro University, Örebro, Sweden; g Department of Obstetrics and Gynaecology, Institute of Clinical Sciences, Lund University, Lund, Sweden; h Institute of Neuroscience and Physiology, Sahlgrenska Academy, University of Gothenburg, Gothenburg, Sweden; i Department of Obstetrics and Gynecology, Stellenbosch University, Cape Town, South Africa

**Keywords:** Prediction, Adverse outcomes, Pre-eclampsia, Hypertensive disorders, Pregnancy

## Abstract

**Background:**

Hypertensive disorders of pregnancy are a leading cause of maternal and perinatal morbidity and mortality. If women at high risk for developing complications could be identified early, level of care could be triaged, limited resources could be correctly allocated and targeted interventions to prevent complications could be implemented.

**Methods:**

We updated a systematic review and meta-analysis and added single outcomes. Women with hypertensive disorders of pregnancy were included. Exposures were tests predicting adverse maternal and/or perinatal outcomes. We searched Medline, Embase, CINAHL, and Cochrane library from January 2016–February 2024. We included studies identified from the previous review. We calculated effect measures. For similar predictive tests and outcomes, area under the receiver-operating-characteristic curve (AUROC) were pooled. This study was registered by PROSPERO: CRD42022336368.

**Findings:**

Of the 2898 studies identified, 80 were included. Thirty were added from the previous review resulting in 110 included studies with 506,178 women. Despite more than 1500 tests being performed, most outcomes could not be pooled due to heterogeneity in populations, tests, and outcome definitions. For maternal outcomes, only studies reporting on the Pre-eclampsia Integrated Estimate of RiSk (fullPIERS) model could be pooled. For the composite outcome within 48-h the AUROC was 0.78 (95% CI 0.71–0.86, N = 8). There was significant heterogeneity (*I*^*2*^ = 95.7%). For perinatal outcomes, data were pooled for pulsatility index in the umbilical artery and soluble FMS-like tyrosine kinase-1 (sFlt-1)/placental growth factor (PlGF) ratio. Biomarkers like the sFlt-1/PlGF ratio showed promising predictive performance for some outcomes but were not externally validated.

**Interpretation:**

Despite including over 100 studies with more than 1500 predictors, we were unable to pool any single maternal outcomes and only a few individual perinatal outcomes. The fullPIERS model was externally validated, showing moderate accuracy which varied across studies and should be validated in each new population. Angiogenic biomarkers showed promise but need validation. Future studies should use standardized outcome measures and validate promising tests.

**Funding:**

VB is supported by the 10.13039/501100004359Swedish Research Council, Grant number 2020-01481. 10.13039/501100005760University of Gothenburg.

**Research in context**

**Evidence before this study**

A systematic review published in 2018 did not identify any predictors that could be implemented in clinical practice to identify who is at risk of adverse maternal outcomes associated with hypertensive disorders of pregnancy (HDP). Before we conducted this updated systematic review, we searched the databases Medline, Embase, CINAHL, and Cochrane library and performed citation tracking from the previous systematic review until February 2024, to identify if there were new systematic reviews using the terms including: “Hypertension”, “Pregnancy”, “HDP”, “Predictive Value of Tests”, “clinical risk assessment” without any language or time restriction and did not identify any updated systematic reviews.

**Added value of this study**

We identified a large number of studies with predictive tests for adverse outcomes in hypertensive disorders of pregnancy. We recognized a lack of consensus definitions of populations, predictors and outcomes which made pooling data inappropriate. We suggest conducting studies to identify predictors for adverse outcomes in countries where they are most prevalent. The only validated predictive test was the Pre-eclampsia Integrated Estimate of RiSk (fullPIERS) model, however, due to high heterogeneity and an overall moderate discriminative accuracy, we recommend validation in the population of interest before clinical implementation.

**Implications of all the available evidence**

Future research should use consensus definitions of adverse outcomes. Validation of the most promising predictors identified in this review, using single outcomes is needed. Composite outcomes are challenging due to heterogeneity in populations and complications.

## Introduction

Hypertensive disorders of pregnancy include chronic hypertension, gestational hypertension, and pre-eclampsia. They complicate 5%–10% of pregnancies and are a leading cause of maternal and perinatal morbidity and mortality.^1^, ^2^, ^3^, ^4^, ^5^, ^6^ Complications occur predominantly in low- and middle-income countries (LMIC).^7^^,^^8^ These include maternal death, eclampsia (pre-eclampsia with generalized tonic-clonic seizures), pulmonary oedema, heart failure, renal failure, liver hematoma and disseminated intravascular coagulation.^9^ Perinatal complications include perinatal death, preterm birth and fetal growth restriction.^9^

If women at high risk for developing these complications could be identified early, it may help triage level of care, allocate resources, and allow for targeted interventions to prevent complications.^10^ There are currently no widely accepted clinical tools to identify these women.

A systematic review summarized the evidence in 2018.^11^ A few promising predictors were identified but these were not externally validated. Many of the included studies were judged to have a high risk of bias.^11^ The review also included multivariable prediction models which included the Pre-eclampsia Integrated Estimate of RiSk (fullPIERS) model. This model uses common clinical symptoms and signs as predictors including gestational age, presence of chest pain or dyspnoea, oxygen saturation, platelet count, serum creatinine levels and serum aspartate aminotransferase or alanine transaminase levels for a composite of maternal outcomes.^12^ The fullPIERS model performed better than single predictors and was externally validated, with moderate predictive accuracy in pre-eclampsia, but the majority of studies were underpowered.^11^

Since this review, a substantial number of studies assessing possible predictors have been published. We therefore conducted an updated systematic review and meta-analysis of current single and multivariable prediction models for maternal outcomes. We also included perinatal adverse outcomes which has not previously been assessed.

## Methods

### Search strategy and selection criteria

We updated a systematic review and meta-analysis of predictors and multivariable prediction models in women with hypertensive disorders of pregnancy. Our primary outcome was adverse maternal outcomes.^11^ Our secondary outcome was adverse perinatal outcomes. The protocol was prospectively registered on PROSPERO (registration number: CRD42022336368).

We searched the databases Medline, Embase, CINAHL, and Cochrane library. The search included studies published from January 2016 until February 2024. The initial search was performed on 12th May 2022 and updated on 12th February 2024. Detailed search subject key words for Medline are shown in Supplementary Table S1 and are the same as the previous systematic review. We also searched for any eligible registered studies on ClinicalTrails.gov and conducted citation tracking. Data from unpublished studies, such as posters or conference abstracts were identified. Because they were not peer reviewed, these studies were separated for possible later classification.^13^

We included cohort studies, case–control studies, and randomized controlled trials among women with hypertensive disorders of pregnancy (Supplementary Table S2). The studies were selected according to a predefined P(I)EO: population (P), exposure (E), and outcomes (O). The population was women with hypertensive disorders of pregnancy. Exposures were all reported risk prediction or prognostic tests for adverse maternal and neonatal outcomes. The primary outcome were adverse maternal outcomes according to the Delphi Consensus published by Duffy et al., 2020 (Supplementary Table S2).^9^ Secondary outcomes were adverse perinatal outcomes according to the same consensus (Supplementary Table S2).^9^ Inclusion and exclusion criteria and outcomes can be found in Supplementary Table S2. We included studies from the previous meta-analysis which used the same search strategy and had included studies published until 2016 if a full text was available.^11^ We included their assessment of risk of bias and study characteristics.

Included studies were required to report at least one of the following for individual tests: 1) sensitivity and specificity, 2) positive and negative likelihood ratios (LR+ and LR−), 3) positive and negative predictive values (PPVs and NPVs), 4) area under the receiver operating characteristic curve (AUROC), or 5) data which could be used to calculate the above-mentioned predictive measures, such as 2 × 2 tables. Full texts were reviewed by two independent reviewers. Discrepancies were resolved by LB or VB.

### Data analysis

Risk of bias was assessed by using a modified version of the Quality in Prognostic Studies (QUIPS) tools.^14^ The assessment of each study was conducted independently by two reviewers (combination of VB, and ARM or JeA). The robvis tool was used to visualize the quality assessment.^15^ Data extraction was conducted by two independent reviewers (VB, ARM, PG and NW). For missing data or data inadequately described, we attempted to contact the study investigators. All attempts were recorded (Supplementary Table S3).

### Statistics

Extracted data were recorded in spreadsheets. 2 × 2 tables were constructed by cross-classifying test results and the occurrence of adverse outcomes. For single outcomes, sensitivity, specificity, NPV and PPV, LR+ and LR− or AUROC were calculated using the R language and environment for statistical computing version 4.3.1 (R Core Team, Vienna, Austria) and the packages epiR (version 2.0.67) and pROC (version 1.18.5).^16^, ^17^, ^18^ The sensitivity, specificity, PPV and NPV values and 95% confidence intervals (95% CI) were calculated using the Wilson’s score method.^19^ For LR+ and LR−, 95% CIs were calculated using Simel’s method.^20^ 95% CIs for AUROC were calculated using the method of DeLong.^21^ If a cell of the 2 × 2 table was equal to zero, a 0.50 pseudo-count was added to each cell of the confusion matrix (true positives, false positives, true negatives, false negatives) to avoid division by zero. The script in R language can be found in Supplementary Table S4.

To avoid double counting of population cohorts, subgroup populations were included if all participant data was presented for the study rather than the overall population. If ranges instead of cut-offs were presented for the predictor, cut-offs were calculated using the 2 × 2 table data. For studies presenting several multivariable models, only the best predictive model was included in the table. To assess the prediction accuracy performance, LR values were evaluated. LR+ from 2.00 to 4.99 were considered as a fair performance as it generates small changes of the pre-test probability to the post-test probability. A value of 5.00 to 9.99 was considered as good and a value equal or higher than 10.00 as excellent performance. LR-values from 0.21 to 0.50 were considered as fair performance, 0.11–0.20 as good, and equal or lower than 0.10 as excellent performance.^22^^,^^23^ For Table 1, Table 2, Table 3 only tests reaching a lower limit of the 95% CI for the LR + above 2.00 or an upper limit of the 95% CI for the LR-below 0.50 are presented.

**Table 1:** Performance of predictive tests for single maternal outcomes with a lower limit of the 95% confidence interval for the positive LR > 2 or upper limit of the 95% confidence interval for negative LR < 0.50.

Confidence intervals for the sensitivity, specificity, positive and negative predictive value were calculated using the Wilson's score method. Confidence intervals for the positive and negative likelihood ratios were calculated using Simel's method. Confidence intervals for the AUROC were calculated using the method of DeLong.

Shading according to likelihood ratio scoring: light blue = fair rule in test, dark blue = good rule in test. Light green = fair rule-out test, dark green = good rule out test.

AST-aspartate aminotransferase; AUROC-area under the receiver operating characteristic curve; HELLP-hemolysis, elevated liver enzymes and low platelet count; hs-CRP-high-sensitive C-reactive protein; ICU- intensive care unit; IUGR-intrauterine growth restriction; −LR-negative likelihood ratio; +LR-positive likelihood ratio; NPV-negative predictive value; PE-pre-eclampsia; PlGF-placental growth factor; PPV-positive predictive value; SD-standard deviation; sFlt-1-soluble fms-like tyrosine kinase-1.

∗0.50 pseudo count added to each cell in the confusion matrix (true positives, false positives, true negatives, and false negatives) to avoid division by zero.

**Table 2:** Performance of prediction tests for the PIERS outcomes with a lower limit of the 95% confidence interval for the positive LR > 2 or upper limit of the 95% confidence interval for negative LR ratio <0.50.

Confidence intervals for the sensitivity, specificity, positive and negative predictive value were calculated using the Wilson's score method. Confidence intervals for the positive and negative likelihood ratios were calculated using Simel's method. Confidence intervals for the AUROC were calculated using the method of DeLong.

Shading according to likelihood ratio scoring: light blue = fair rule in test, dark blue = good rule in test. Light green = fair rule-out test, dark green = good rule out test.

AUROC, area under the receiver operating characteristic curve; Dutch PETRA, Pre-eclampsia Eclampsia Trial Amsterdam; LR+, positive likelihood ratio; LR−, negative likelihood ratio; NPV, negative predictive value; PIERS, Pre-eclampsia Integrated Estimate of RiSk; PPV, positive predictive value; PREP, Prediction of Complications in Early-Onset Pre-eclampsia.

**Table 3:** Performance of all prediction tests for single perinatal outcomes with a lower limit of the 95% confidence interval for the positive LR > 2 or upper limit of the 95% confidence interval for negative LR < 0.50.

Confidence intervals for the sensitivity, specificity, positive and negative predictive value were calculated using the Wilson's score method. Confidence intervals for the positive and negative likelihood ratios were calculated using Simel's method. Confidence intervals for the AUROC were calculated using the method of DeLong.

Shading according to likelihood ratio scoring: light blue = fair rule in test, dark blue = good rule in test. Light green = fair rule-out test, dark green = good rule out test.

AUROC- area under the receiver operating characteristic curve; BDA-bronchopulmonary dysplasia; CPR-cerebroplacental ratio; GA-gestational age; hs-CRP,-high-sensitive C-reactive protein; IVH- intraventricular haemorrhage; −LR- negative likelihood ratio; +LR-positive likelihood ratio; NICU-neonatal intensive care unit; NPV- negative predictive value; PDA-patent ductus arteriosus; PI-pulsatility index; PlGF-placental growth factor; PPV-positive predictive value; RDS- respiratory distress syndrome; ROP- retinopathy of prematurity; sFlt-soluble fms-like tyrosine kinase-1; UA-umbilical artery; UtA-uterine artery.

∗0.50 pseudo count added to each cell in the confusion matrix (true positives, false positives, true negatives, and false negatives) to avoid division by zero.

Results were pooled and meta-analysis conducted if there were results from more than one predictor of the same character on similar outcomes and presented as an AUROC curve and corresponding 95% CI. All meta-analyses were performed as random-effects meta-analyses to determine the pooled AUROC curve, 95% CI and p-value. AUROC values > 0.70 to ≤0.80 were considered moderate, AUROC > 0.80 to ≤0.90, good and AUROC >0.90 as excellent predictive performance.^24^

Two-sided p-values of <0.05 were considered statistically significant. We used StataSE version 17 (StataCorp. 2021, Stata Statistical Software: Release 17. College Station, TX) for meta-analyses.

### Ethics

Due to the nature of this study with publicly available and published aggregated data, no ethical approval was required.

### Role of the funding source

The funding sources had no role in study design, data collection, data analysis, data interpretation, or writing of the report.

## Results

**Fig. 1:**
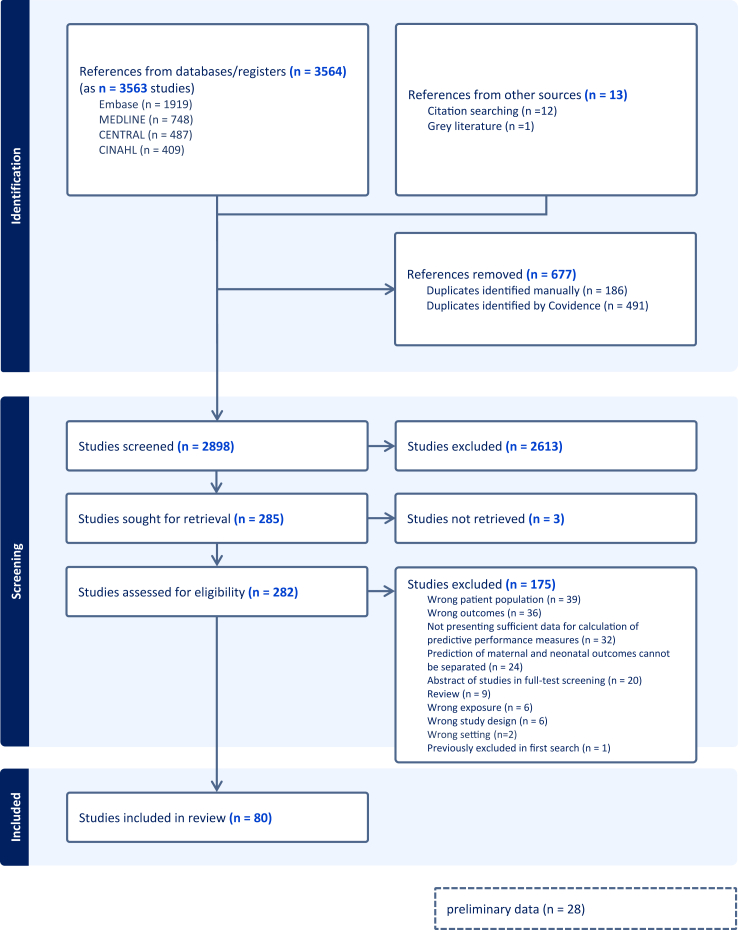
Preferred reporting items for systematic reviews and meta-analyses (PRISMA) flow chart.

**Fig. 2:**
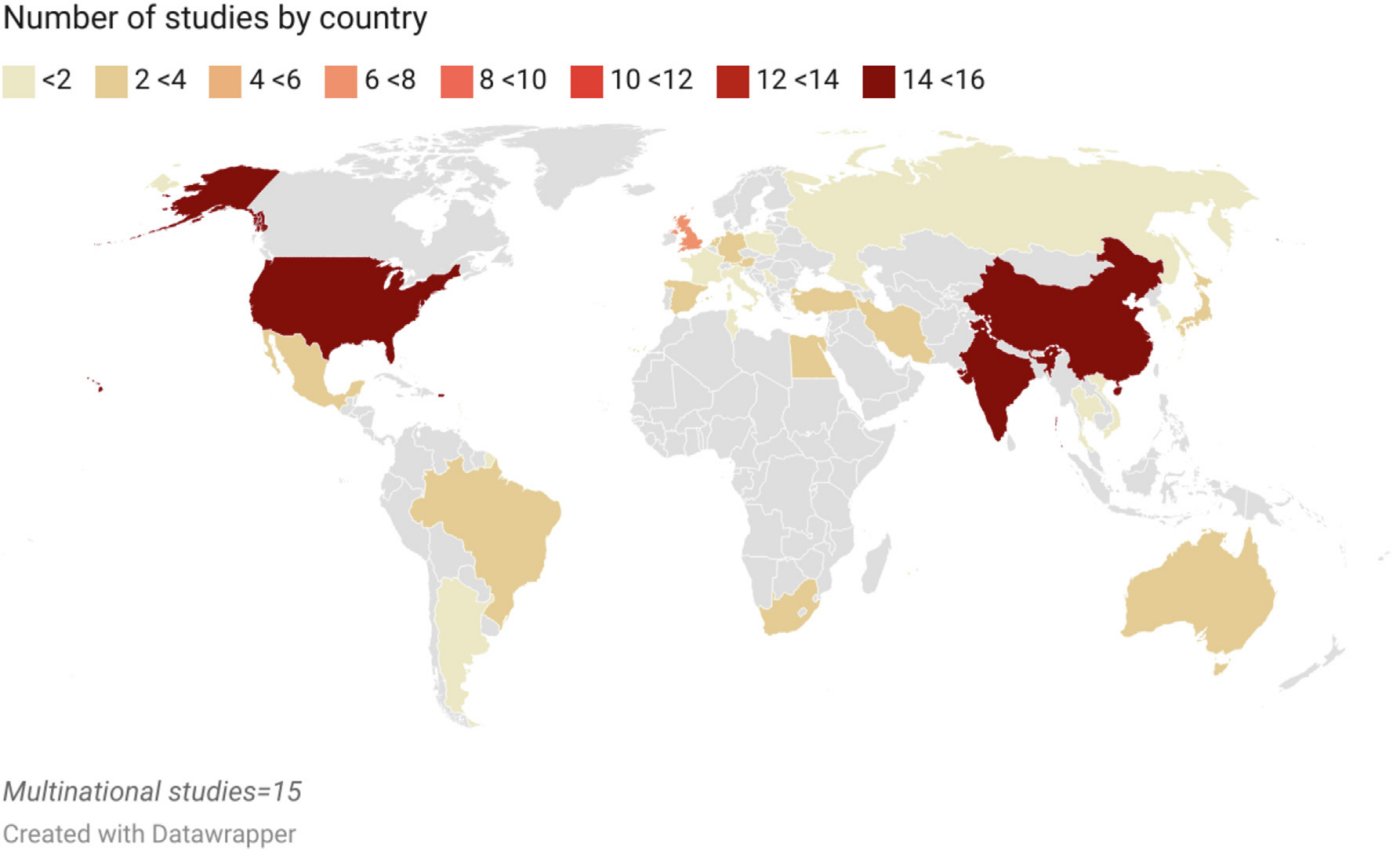
Study distribution map showing the number of studies by county, created with Datawrapper

**Fig. 3:**
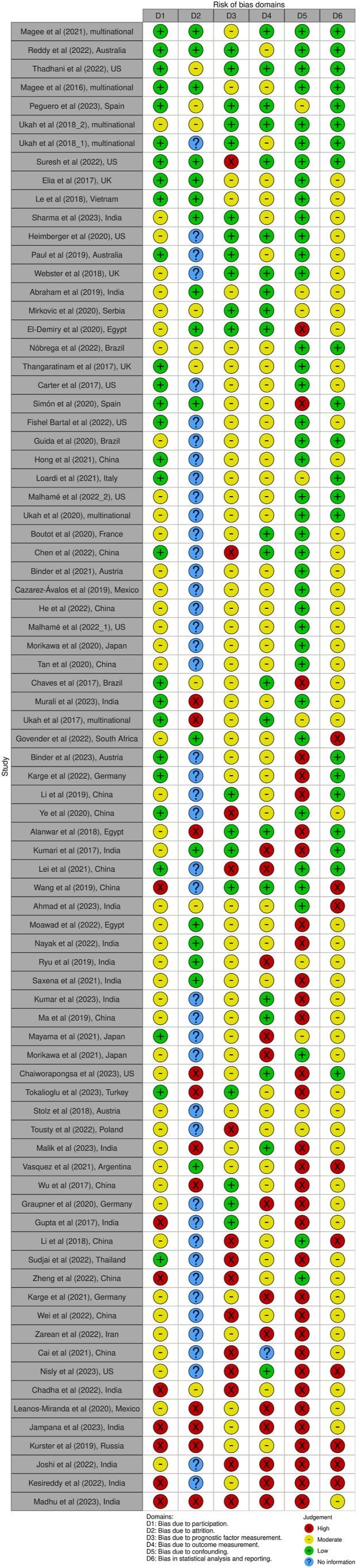
Risk of bias summary showing the authors’ judgements on each domain for the included studies using the modified Quality in Prognostic Studies (QUIPS) tool. Studies were sorted according to the number of high risk of bias in the 6 domains from top to bottom starting with lowest number of high risk of bias. Risk of bias assessment of studies retrieved from Ukah et al., 2018 can be found in Supplementary Table S5 of the corresponding publication.^11^ D1: Bias due to participation. D2: Bias due to attrition. D3: Bias due to prognostic factor measurement. D4: Bias due to outcome measurement. D5: Bias due to confounding. D6: Bias due to statistical analysis. UK–United Kingdom; US-United States.

We screened 2898 abstracts, 282 full texts and 80 studies were included (Fig. 1).^25^, ^26^, ^27^, ^28^, ^29^, ^30^, ^31^, ^32^, ^33^, ^34^, ^35^, ^36^, ^37^, ^38^, ^39^, ^40^, ^41^, ^42^, ^43^, ^44^, ^45^, ^46^, ^47^, ^48^, ^49^, ^50^, ^51^, ^52^, ^53^, ^54^, ^55^, ^56^, ^57^, ^58^, ^59^, ^60^^,^^61^, ^62^, ^63^, ^64^, ^65^, ^66^, ^67^, ^68^, ^69^, ^70^, ^71^, ^72^, ^73^, ^74^, ^75^^,^^76^, ^77^, ^78^, ^79^, ^80^, ^81^, ^82^, ^83^, ^84^, ^85^, ^86^, ^87^, ^88^, ^89^, ^90^, ^91^, ^92^, ^93^, ^94^, ^95^, ^96^, ^97^, ^98^, ^99^, ^100^, ^101^, ^102^, ^103^, ^104^ The previous review included 30 full text studies, and these were added.^11^^,^^12^^,^^105^, ^106^, ^107^, ^108^, ^109^, ^110^, ^111^, ^112^, ^113^, ^114^, ^115^, ^116^, ^117^, ^118^, ^119^, ^120^, ^121^, ^122^, ^123^, ^124^, ^125^, ^126^, ^127^, ^128^, ^129^, ^130^, ^131^, ^132^, ^133^ In total, 506,178 women in 110 studies were included. Characteristics of these studies are presented in Supplementary Table S5.^11^ Details on exclusions can be found in Supplementary Table S6. Fifty-seven studies were conducted in low- and middle-income countries. Fifteen were multinational (Fig. 2). Twenty-eight were multicenter and 82 were singlecenter. Cohort studies were most common (76/110). Fifty-eight studies were prospective, 43 were retrospective and for nine studies it could not be determined (Supplementary Table S5). Hypertensive disorders of pregnancy were defined differently, generating several subgroups (Supplementary Table S5). The risk of bias for the 80 studies identified is summarized in Fig. 3. Forty-eight were classified as high risk of bias in at least one domain. The risk of bias of the studies identified in the previous systematic review was moderate to high overall.^11^

## Single maternal adverse outcomes

We divided single maternal adverse outcomes into 13 categories: maternal death, admission to intensive care unit (ICU), eclampsia, neurological complications, retinal disease, cardiac disease, pulmonary oedema/respiratory failure, renal injury, liver dysfunction, hemolysis, elevated liver enzymes and low platelet count (HELLP) syndrome, disseminated intravascular coagulation (DIC), ascites and haematological complications. All extracted prediction tests and corresponding single maternal outcomes are presented in Supplementary Table S7.

Table 1 includes all outcomes with at least fair prognostic performance. No studies of single maternal adverse outcomes had the same predictors and outcomes, so we were unable to perform any meta-analyses.

### Individual outcomes

#### Admission to ICU

22 tests from 11 studies were identified. The rate of admission to ICU varied from 0.6% to 28.4%. No predictors achieved good performance (Table 1).

#### Eclampsia

37 predictive tests from 15 studies were extracted. The rate of eclampsia varied from 0.4% to 34.2%. No test achieved a good predictive performance.

#### Retinal disease

17 tests from four studies were extracted. The rate of retinal disease varied from 1.4% to 14.6%. Serum total protein level at delivery ≤42 g/L had LR+ of 68.25 (95% CI 8.97–519.47) for prediction of central serous chorioretinopathy, but there were only four women in the outcome group.

#### Cardiac disease

10 tests from five studies were identified. Cardiac disease occurred in 0.2% to 4.4% of cases. No test achieved a good predictive performance (Table 1).

#### Renal injury

26 predictors from 14 studies were identified. The definition, laboratory methods and cut-off values for renal injury differed between studies. Renal injury occurred in 0.6%–15.9% of cases. No predictors with good performance were identified (Table 1).

#### Liver dysfunction

21 predictors from seven studies were identified. Liver complications were defined differently in the studies. The rate of liver complications varied from 1.0% to 31.0%. A soluble FMS-like tyrosine kinase-1 (sFlt-1)/placental growth factor (PlGF) ratio ≥85 achieved LR− 0.06 (95% CI 0.02–0.19) for the prediction of liver enzymes ≥ double the reference value.

#### HELLP syndrome

27 tests from 15 studies were identified. The rate of HELLP varied from 0.5% to 22.1%. No predictors with good performance were identified (Table 1).

#### Haematological outcomes

78 tests from 28 studies were identified. The rate of placental abruption ranged from 0.5% to 20.7% and the rate of thrombocytopenia from 1.8% to 29.8%. A multivariable model based on the machine-learning algorithm multi-layer perceptron presented a LR + value of 99.12 (95% CI 5.39–1822.83) for the prediction of placental abruption.

#### Other outcomes

No prognostic test fulfilled the prespecified requirement of performance reliability with a lower limit for the LR+ > 2.00 or upper limit for LR− < 0.50 for the single outcomes, maternal death, DIC, pulmonary oedema, ascites, or neurological complications. These outcomes all had a low outcome rate in the included studies (Supplementary Table S7).

## Composite maternal outcomes

### Meta-analyses

All studies with composite outcomes are presented in Supplementary Table S8. Composite outcomes often included different sub-components. We were only able to perform meta-analyses for studies comparing the same predictors and composite outcomes. The fullPIERS model was the only one to meet these requirements (Table 2). Meta-analyses were performed for the composite fullPIERS outcome 1) within 48 h and 2) within 7 days (Supplementary Fig. S1A and B).

For the fullPIERS composite outcome within 48 h, eight external validation studies were included. The pooled AUROC was 0.78 (95% CI 0.71–0.86, N = 8), suggesting a moderate to good predictive performance. However, there was significant heterogeneity (*I*^2^ = 95.7%). A subgroup analysis of studies in high-income countries achieved a pooled AUROC of 0.82 (95% CI 0.71–0.93, N = 4) still with significant heterogeneity (*I*^*2*^ = 96.2%). The pooled AUROC of studies in LMIC was 0.75 (95% CI 0.65–0.84, N = 4) with significant heterogeneity (*I*^*2*^ = 93.3%). The risk of bias was moderate to high for this composite outcome.

For the fullPIERS composite outcome at seven days, five studies were included. The pooled AUROC was 0.75 (95% CI 0.69–0.82, N = 5) suggesting moderate predictive performance. The heterogeneity across the studies was high (*I*^*2*^ = 80.2%). The subgroup analysis revealed an AUROC of 0.74 (95% CI 0.68–0.80, N = 3) for studies in high-income countries and an AUROC of 0.75 (95%CI 0.55–0.96, N = 2) in LMIC. Both subgroup analyses had significant heterogeneity (*I*^*2*^ = 75.2% and *I*^*2*^ = 76.8% respectively). There were two external validation studies of the fullPIERS model that did not specify the timeframe for prediction and were not included in meta-analysis.

### Individual studies

Fourteen studies used the fullPIERS composite outcome but with different predictors (Supplementary Table S9). A multivariable model based on support vector machine with imputation showed LR+ value of 12.06 (95% CI 8.66–16.78), LR-value of 0.08 (95% CI 0.05–0.14) and AUROC value of 0.53 (95% CI 0.50–0.56) (Table 2).

## Individual perinatal adverse outcomes

### Meta-analyses

We were able to pool estimates for perinatal outcomes in four meta-analyses (Supplementary Fig. S2). Two studies reported uterine artery pulsatility index as a predictor for fetal growth restriction, with a pooled AUROC of 0.72 (95% CI 0.67–0.77, N = 2) (Supplementary Fig. S2A). Two studies used the uterine artery pulsatility index as a predictor for respiratory distress syndrome. The AUROC was 0.50 (95% CI 0.15–0.84, N = 2) (Supplementary Fig. S2B). Two studies investigated sFlt-1/PlGF ratio ≥85 to predict neonatal death resulting in an AUROC of 0.76 (95% CI 0.74–0.78, N = 2) (Supplementary Fig. S2C). sFlt-1/PlGF ratio ≥85 was used to predict neonatal intensive care unit (NICU) admission (AUROC of 0.64 (95% CI 0.59–0.70) (Supplementary Fig. S2D). All pooled estimates demonstrated a low to moderate discriminatory accuracy.

### Individual studies

Individual perinatal outcomes were divided into six subgroups (Table 3, Supplementary Table S10).

#### Perinatal death

75 tests from 28 studies were identified. The rate of perinatal death varied from 0.3% to 26.0%. Absent end diastolic flow on umbilical artery Doppler had a LR + value of 102.78 (95% CI 6.41–1647.36). The sFlt-1/PlGF ratio ≥85 had a LR-value of 0.06 (95% CI 0.02–0.16).

#### Admission to neonatal intensive care unit (NICU)

49 tests in 23 studies were identified. Admission to NICU occurred in 5.4% to 57.2% of cases. No predictors achieved good performance (Table 3).

#### Poor neonatal outcomes (patent ductus arteriosus, necrotizing enterocolitis, intraventricular haemorrhage, retinopathy of prematurity, and neonatal seizures)

27 tests in nine studies were identified. Poor neonatal outcomes occurred in 0.1% to 14.8% of cases. No predictors achieved good performance (Table 3).

#### Respiratory outcomes

39 tests in 14 studies were identified. Respiratory outcomes were poorly defined. Respiratory distress syndrome was the most common definition occurring in 4.5% to 76.7% of cases. No predictors achieved good performance (Table 3).

#### Preterm birth

136 tests in 21 studies were identified. Preterm birth was defined as <37, 34 or 32 weeks of gestation. The rate for preterm birth <37 weeks varied from 15.6% to 84.3% and for <34 weeks from 6.8% to 54.9%. The sFlt-1/PlGF ratio ≥85 had LR+ of 11.58 (95% CI 6.32–21.19) for the prediction of preterm birth <37 weeks.

#### Low birthweight/fetal growth restriction/small for gestational age/intrauterine growth restriction

129 predictors in 30 studies were identified. Many were defined differently. A high sensitivity serum C-reactive protein >3.0 mg/mL had a LR-value of 0.05 (95% CI 0.01–0.20) for the prediction of low birth weight.

## Composite perinatal outcome

No composite perinatal outcomes were the same, and pooling could therefore not be performed (Supplementary Table S11).

## Discussion

In this large systematic review, which included 110 studies with over 1500 predictive performance tests, only six meta-analyses could be performed. We could not pool any data for single maternal adverse outcomes. The meta-analyses of the fullPIERS model, assessing a composite of maternal adverse outcomes within 48 h and within 7 days showed moderate to good and weak discriminative performance respectively. The four pooled estimates for single perinatal outcomes included only a few studies of mostly poor quality and no data could be pooled for composite perinatal outcomes. Angiogenic markers showed promise as a predictor for both maternal and perinatal outcomes but were only assessed in individual studies and need validation.

To be able to assess evidence through meta-analyses of pooled data, it is important for studies to use similar predictors and outcomes with the same definitions. In this review it was not possible to pool results for most predictors and outcomes as different predictors, outcomes and definitions were used. This is a common problem in obstetric research.^134^ A core outcome set like the Delphi consensus of maternal and perinatal adverse outcomes for pre-eclampsia should ideally be used for future studies.^9^^,^^11^

For composite outcomes, we could only pool results for the fullPIERS prediction model. For composite adverse maternal outcomes within 48 h, heterogeneity was high, and the risk of bias was moderate to high. The fullPIERS model only reached moderate performance with a wide confidence interval. This is in contrast to the good to excellent predictive performance of the original study of the fullPIERS model.^12^ Heterogeneity may be due to the diverse settings and different populations included. There were also different compositions of the composite outcome that affected the results. We conducted subgroup analyses according to country income settings. Even within these subgroups, heterogeneity was still significant. We pooled the data and used a random-effects model due to the heterogeneity. Because of the large variance in results between studies and settings, it is important to still validate the model in each new population of interest. Five external validations studies of the fullPIERS model identified in this search were not included in our systematic review due to uncertainty of the design or because fetal and maternal outcomes could not be separated (Supplementary Table S6).

Composite outcomes can be problematic. There may be contradicting pathophysiological mechanisms of included components, for example when combining a neurological outcome such as eclampsia with a bleeding outcome such as postpartum haemorrhage.^135^ Less severe, more common components can drive results.^135^ Therefore, studies using single outcomes may provide more solid evidence. Our systematic review aimed to focus on important single maternal and fetal outcomes in pre-eclampsia to summarize the most promising predictors.

We identified only a few predictors for single maternal and fetal outcomes that merit further investigation and external validation. The most broadly reported and promising predictors were the angiogenic markers sFlt-1 and PlGF. These biomarkers are already used in clinical practice to predict and diagnose pre-eclampsia but are not yet recommended for prediction of complications.^136^ Even though they were included in 15 studies in our review, the cut-off values ranged from 38 to 794 and the outcomes varied, making comparison and pooling impossible. We found a fair to good predictive performance of sFlt-1/PlGF ratio ≥85 for some single outcomes, such as rule-out test for maternal liver disease, rule-out test for perinatal death and rule-in test for preterm birth but all still require validation. Several manufacturers provide analytic platforms to measure angiogenic markers with different ranges, specific to the analytic platform used. There is a need for translation of cut-offs between different analytic platforms to facilitate comparisons between studies.

Strengths of this systematic review and meta-analysis include the large number of studies and predictors included. We also analysed adverse maternal as well as perinatal outcomes covering all maternal, fetal, and neonatal complications according to the Delphi Consensus.^9^ We were able to divide the single outcomes according to the involved organ systems, increasing clinical applicability.^135^

A limitation is the lack of external validation of the identified predictors. This was mainly due to discrepancies in definition and choice of predictors and outcomes from included studies. Another limitation lies in the moderate to high risk of bias found in most studies and the small number of events in the single outcomes. This was particularly evident in important outcomes such as mortality and eclampsia. These outcomes are rare in high-income countries where many of the included studies were conducted.

The prediction of adverse maternal and perinatal outcomes in women with hypertensive disorders of pregnancy is important to inform clinical decisions about optimal care and interventions. All women with hypertensive disorders of pregnancy are at an increased risk for maternal and perinatal adverse outcomes. A test in this setting that is clinically useful needs to have a high sensitivity to ensure women or neonates that will experience an adverse outcome are captured. Often, this comes at a cost of a lower specificity, meaning that a large group of women with hypertensive disorders of pregnancy will be classified as high risk even if they will not be affected by adverse outcomes. For these tests, it is crucial to evaluate that the test 1) does not lead to unnecessary and potentially harmful interventions and 2) is cost-effective. After summarizing the current evidence, we cannot suggest any predictive test to be implemented in clinical practice at this stage.

A defined core outcome set and external validation of promising prediction tests using predefined cut-off values for future research should be performed. We recommend conducting studies for discovery of predictors for serious maternal adverse outcomes in countries where these complications are most prevalent.

The fullPIERS model within 48 h only had a moderate performance that varied across populations. In addition, the choice of a composite outcome is problematic since different sub-components can drive the result. We suggest assessing the model in the population of interest and including a cost-effectiveness analysis to ensure clinical usefulness in specific settings before considering implementation.

A potential next step could be the performance of an individual participant data meta-analysis. The International Prediction of Pregnancy Complication Collaborative Network has previously conducted an individual participant data meta-analysis for the validation of prediction model for pre-eclampsia.^137^ Interestingly, this study concluded that there is a high heterogeneity between the included studies and the measurement of predictors needs standardization similar to our conclusion.

The evidence for the prediction of adverse maternal and perinatal outcomes in hypertensive disorders of pregnancy is inadequate. This is due to poor study design and heterogeneity in the definition, population, predictors, and outcomes in included studies. Only the fullPIERS model showed moderate accuracy, which varied across studies and should be validated in each population of interest. Validation studies using core outcome sets and well-defined promising predictors are needed.

## Contributors

VB, CC, LB conceptualized the study. CC, LB and RH provided overall supervision. VB, ARM, JeA, PG and NW extracted and assessed the data. VB, ARM, JeA, PG and NW verified the underlying data. ARM, VB and RH did the statistical analysis. VB wrote the first draft of the report with input from LB, CC, RH and ARM. All authors searched and screened the original articles. All authors edited, validated, critically revised the manuscript and approved the final version of the manuscript. All authors had direct access to the full dataset and had the final responsibility for the decision to submit for publication.

## Data sharing statement

All datasets generated and analysed, including the PRISMA protocol, search strategy, list of the included and excluded studies, attempts to contact authors, data extracted, analysis and calculation plans, quality assessment, are available in the article and Supplementary material and upon request from the corresponding author.

## Editor note

The Lancet Group takes a neutral position with respect to territorial claims in published maps and institutional affiliations.

## Declaration of interests

We declare no competing interests.
