# Supplementary material for: Prediction of adverse maternal and perinatal outcomes associated with pre-eclampsia and hypertensive disorders of pregnancy: a systematic review and meta-analysis
Source: eClinicalMedicine. 2024 Sep 27;76:102861. doi: 10.1016/j.eclinm.2024.102861 (PMC11465897; doi:10.1016/j.eclinm.2024.102861)
Supplement: Supplementary Figs. S1 and S2 and Tables S1–S11 [file mmc1.docx]

**Supplementary material for “Prediction of adverse maternal and perinatal outcomes associated with pre-eclampsia and hypertensive disorders of pregnancy: a systematic review and meta-analysis”**

**Table of Content**

**Supplementary table 1. Search strategy in MeSH terms in Medline** 2

**Supplementary table 2: Inclusion and exclusion criteria according to P(I)EO** 3

**Supplemental table 3: Additional requests for information** 5

**Supplementary table 4. Example and full script in R language used to calculate effect measures** 7

**Supplementary table 5: Table of all included studies** 12

**Supplementary table 6: Studies excluded and awaiting classification** 39

**Supplementary table 7: Predictive performance of all prediction tests for single maternal outcomes.** 42

**Supplementary table 8: Predictive performance of all prediction tests for composite maternal outcomes.** 79

**Supplementary table 9: Predictive performance of all prediction tests other than fullPIERS model for PIERS outcomes** 130

**Supplementary table 10: Predictive performance of all prediction tests for single perinatal outcomes.** 135

**Supplementary table 11: Predictive performance of all prediction tests for composite perinatal outcomes.** 188

**Supplementary figure 1** 230

**Supplementary figure 2** 232

**References relating to excluded studies** 234

**Supplementary table 1. Search strategy in MeSH terms in Medline**

|  | **Searches** | **Results** |
| --- | --- | --- |
| 1 | exp Hypertension, Pregnancy-Induced/ | 40,535 |
| 2 | (HDP or HDPs).mp. | 2,080 |
| 3 | (preeclamp* or pre-eclamp*).mp. | 47,832 |
| 4 | ((Chronic hypertens* or essential hypertens* or preexisting hypertens* or pre-existing hypertens*) adj3 (pregnan* or gestation*)).mp. | 936 |
| 5 | HELLP Syndrome/ | 1,937 |
| 6 | HELLP.mp. | 3,140 |
| 7 | or/1-6 | 54,891 |
| 8 | nomograms/ | 6,115 |
| 9 | Models, statistical/ | 97,987 |
| 10 | logistic models/ | 150,178 |
| 11 | "Predictive Value of Tests"/ | 220,799 |
| 12 | Risk assessment/ | 299,210 |
| 13 | clinical risk assessment*.mp. | 496 |
| 14 | prognos* model*.mp. | 7,690 |
| 15 | predict* model*.mp. | 52,252 |
| 16 | (AUC or AUROC or area under the receiver or ROC or ROCs or Receiver operating curve*).mp. | 189,781 |
| 17 | sensitivit*.mp. | 1,270,486 |
| 18 | specificit*.mp. | 1,129,633 |
| 19 | (LR* or likelihood ratio*).mp. | 74,877 |
| 20 | negative predictive value*.mp. | 52,190 |
| 21 | positive predictive value*.mp. | 52,406 |
| 22 | or/8-21 | 2,670,939 |
| 23 | ((risk* or predict* or prognos*) adj6 (adverse or complication* or outcome* or event* or situation*)).mp. | 466,411 |
| 24 | ((risk* or predict* or prognos*) adj6 (morbid* or mortality)).mp. | 179,590 |
| 25 | ((risk* or predict* or prognos*) adj6 (Hepatic or GCS or Glasgow or Stroke or Cortical or RIND or retinal or Dialysis or renal or PIS or Positive inotropic support or Infusion or Myocardial or MI or Intubation or thrombocytop?nia)).mp. | 186,375 |
| 26 | 23 or 24 or 25 | 744,121 |
| 27 | 7 and 22 and 26 | 1,435 |
| 28 | 7 and 22 and 26 | 1,435 |
| 29 | limit 28 to yr="2016 -Current" | 657 |
| **Field labels:** exp/ = exploded MeSH term; / = non exploded MeSH term; .ti,ab,kf. = title, abstract and author keywords; adjx = within x words, regardless of order; * = truncation of word for alternate endings | | |

**Supplementary table 2: Inclusion and exclusion criteria according to P(I)EO**

|  | **Inclusion** | **Exclusion** |
| --- | --- | --- |
| **Population** | Women with any HDPs:   - gestational hypertension - chronic hypertension - pre-eclampsia - super-imposed pre-eclampsia - HELLP syndrome | Studies not recruiting women with HDP |
| **Intervention/Exposure** | reporting risk prediction or prediction test for adverse maternal and neonatal outcomes  predictive measures:   - Sensitivity - Specificity - LR+ and LR- - PPV and NPV - AUROC - result data can be used to calculate one of above-mentioned measures | Studies that do not report prediction tests  OR  studies not presenting sufficient data for calculation |
| **Control** | None |  |
| **Outcomes** | Adverse maternal outcomes:  maternal mortality or one or more serious   - CNS (eclampsia (≥1), Glasgow coma score <13,   stroke or reversible ischemic neurological deficit  transient ischemic attack, cortical blindness or retinal detachment,  posterior reversible encephalopathy syndrome)   - cardiorespiratory (positive inotropic support, infusion of a third parenteral antihypertensive drug, myocardial ischemia or infarction, SpO2 <90%, ≥50%, FiO2 for >1 h, intubation and ventilation (other than for caesarean section), pulmonary oedema) - hepatic (dysfunction (raised liver enzymes), hematoma or rupture) - renal (acute renal insufficiency (creatinine >150 μmol/L; no pre-existing renal disease), acute renal failure (creatinine >200 μmol/L; pre-existing renal disease), dialysis) - haematological (transfusion of any blood product, platelet count <50×10⁹ per L, with no transfusion) - Placental abruption - Severe ascites, Bell's palsy - Death - PPH - admission to ICU - DIC | Studies without outcome measures  OR  Studies that report on combined fetal and maternal outcome and the prediction of maternal outcome cannot be separated |
| **Additional outcomes** | Adverse neonatal outcomes:   - stillbirth - preterm birth - low birthweight - SGA - neonatal mortality - seizures - admission to NICU - respiratory support | Studies without outcome measures  OR  Studies that report on combined fetal and maternal outcome and the prediction of maternal outcome cannot be separated |
| **Types of Studies** | - cohort studies - case-control studies - RCT |  |
| **Language** | All languages |  |
| **Publication year limit** | 2016- current | Before 2016 |
| **Abbreviations**: AUROC, area under the receiver operating characteristic curve; CNS, central nervous system; DIC, disseminated intravascular coagulation; FiO2, Fraction of inspired oxygen; HELLP, hemolysis, elevated liver enzymes and low platelet count; ICU, intensive care unit; LR-, negative likelihood ratio; LR+, positive likelihood ratio; NICU, neonatal intensive care unit.; NPV, negative predicate value; PPH, postpartum haemorrhage; PPV, positive predictive value; SGA, small for gestation age; SpO2, oxygen saturation; HDP, Hypertensive disorder of pregnancy | | |

**Supplemental table 3: Additional requests for information**

|  | **Doi** | **Corresponding author** | **Date** | **Question** | **Response** |
| --- | --- | --- | --- | --- | --- |
| **Binder et al, 2022** | 10.1002/uog.23711 | julia.binder@meduniwien.ac.at | 03.04.2023 | Outcome rate of composite adverse neonatal outcomes | No answer |
| **Chadha et al, 2022** | 10.7759/cureus.23341 | drarzoochadha@gmail.com | 03.04.2023 | Outcome rate of composite adverse neonatal and maternal outcomes | No answer |
| **Le et al, 2019** | 10.1002/ijgo.12697 | leminhtam@huemed-univ.edu.vn | 03.04.2023 | Outcome rate of composite adverse neonatal outcomes | No answer |
| **Moawad et al, 2022** | 10.1186/s12884-022-04704-0 | Iman.moawad@kasralainy.edu.eg and eimanmoawad@yahoo.com | 03.04.2023 | Outcome rate of composite adverse neonatal and maternal outcomes | No answer |
| **Ngwenya et al, 2020** | 10.1016/j.preghy.2020.05.004 | drsolngwe@yahoo.co.uk | 03.04.2023 | - The study published in 2020 which was conducted from January 1, 2016 to December 31, 2018 and the study published in 2021 which was conducted from January 1, 2014 to December 31, 2018 have the same number of participants although the same inclusion criteria were applied, but article 1 has two more years of inclusion?  - Table 1, 4 and 5 have wrongly formatted rows so that corresponding description and data is not presented in the same row  - The numbers of table 2 and 3 and 4 are conflicting. | No answer |
| **Ngwenya et al, 2021** | 10.1016/j.preghy.2020.10.011 | drsolngwe@yahoo.co.uk | 03.04.2023 | - Table 2 is wrongly formatted, data cannot be extracted.  - No description of the study dataset from miniPIERS. | No answer |
| **Saxena et al, 2021** | 10.7860/JCDR/2021/47801.15307 | upma_saxena@hotmail.com | 03.04.2023 | Outcome rate of composite adverse neonatal outcomes | No answer |
| **Tan et al, 2020** | 10.1038/s41598-020-72527-0 | xinghuiliu@163.com and sunx79@hotmail.com | 03.04.2023 | - unclear labeling in the supplemental table 1  - Numbers of supplemental table 1 do not add up to the total included participants, but no explanation is given. | No answer |
| **Thangaratinam et al, 2017** | 10.1186/s12916-017-0827-3 | j.allotey@qmul.ac.uk and s.thangaratinam.1@bham.ac.uk | 03.04.2023 and 14.06.2023 | Data for adverse maternal and neonatal outcomes available individually? | Answer received |
| **Tanacan et al, 2019** | 10.1080/10641955.2019.1590718 | atakantanacan@yahoo.com | 03.04.2023 and 14.06.2023 | - Description of composite outcomes and data in table 2 are not corresponding. More cases in one subcomponent of the composite outcome than in the composite outcome.  Reply to answer: Uncertainties still remain. Clarification of the problem using an example: 8 women are presented with at least HELLP syndrome in table 2. All these 8 women should be presented in the composite outcomes according to the composite outcome definition. It can be that some of them have additional other features such as HELLP and placental abruption. These women are not counted twice. But per definition at least all participants suffering from HELLP syndrome should be included in composite adverse maternal outcomes, so the minimum number that can be in this line is 8. In table 2, this number is 5. | Answer received  No answer to reply |
| **Reddy et al, 2022** | 10.1002/uog.24851 | Maya.Reddy@monash.edu | 03.04.2023 | The description OA PR and OA PSV are used to describe the same data. Which variable was measured? | OA-PSV and OA PR are synonyms. (04.04.2023) |
| **Li et al, 2018** | 10.1080/10641955.2018.1487564 | yanghuixia@bjmu.edu.cn | 03.04.2023 | - Table 2 describe thrombocytopenia in two columns.  - ALT and AST have been used interchangeably. Which variable has been measured? | The third row in table 2 should be thrombocytopenia and the fourth row should be cerebral or visual symptoms. In our study, increased liver enzymes refers to ALT. (28.04.2023) |
| **Kawakita et al, 2023** | 10.1016/j.ajog.2022.11.404 | Tetsuya.x.kawakita@gmail.com | 15.05.2023 | - Asking if full-data set for PIERS external validation was available | No answer |
| **Nathan et al, 2018_1** | 10.1016/j.preghy.2017.11.003 | hannah.nathan@kcl.ac.uk and andrew.shennan@kcl.ac.uk | 05.07.2023 | - The number of participants experiencing preterm birth are different in table 2, 3 and 4.  - The total number of live births are different in table 3 and 4. | No answer |
| **Nathan et al, 2018_2** | 10.7189/jogh.08.020401 | hannah.nathan@kcl.ac.uk and andrew.shennan@kcl.ac.uk | 05.07.2023 | - The same cohort with the same inclusion and exclusion criteria has been analysed as in the pervious article. However, the number of participants measuring kidney injury is different. | No answer |
| **Ye et al, 2020** | 10.1097/MD.0000000000019349 | zhourong_hx@scu.edu.cn | 05.07.2023 | - Two participants are missing in the measurement of the prediction factor hemoglobin without explanation. | No answer |
| **Chaudhary et al, 2024** | - | anchal16.maheshwari@gmail.com | 21.02.2024 | - only 167 women with preeclampsia were admitted to the recruitment site, but 300 women were included in the study. How could more women be recruited?  - no definition of adverse outcomes. | No answer |
| **Binder et al, 2023** | 10.1002/uog.26276 | julia.binder@meduniwien.ac.at | 28.02.2024 | - Definition of “sFlt-1/PlGF ratio abnormal” | No answer |
| **Govender et al, 2022** | 10.1002/ijgo.14495 | vaeochan@gmail.com | 28.02.2024 | - Definition of “DVD abnormal” | No answer |
| **Madhu et al, 2023** | - | aims@bgsaims.edu.in | 28.02.2024 | - How was CPR calculated and elevated Doppler measures defined?  - Text and table 2 show different data for the same test, which is correct? | No answer |
| **Boutot et al, 2019** | 10.1016/j.gofs.2020.01.002 | tristan.gauthier@chu-limoges.fr | 28.02.2024 | - Text and figure 2 show different data for the same test, which is correct?  - In table 2, n of women with adverse outcomes is possibly not correct. | - data in the text was confirmed after recalculation  - n= 34 of women with adverse outcomes. (24.03.2024) |
| **Liao et al, 2018** | - | xinghuiliu@163.com and 526543200@qq.com | 06.03.2024 and 07.03.2024 | - Information about data in table 1 is missing. How has missing data been handled? | No answer |
| **Srivastava et al, 2017** | 10.18203/2320-1770.ijrcog20170889 | drshubhasrivastava76@gmail.com | 12.03.2024 | -Are composite outcomes combining fetal and maternal outcomes? Definitions of outcomes are inconsistent. | No answer |
| **Abbreviations:** ALT, alanine aminotransferase; AST, aspartate transaminase; CPR, cerebroplacental ratio; DVD, ductus venosus doppler; HELLP, hemolysis, elevated liver enzymes and low platelet count; OA PR, ophthalmic artery peak ratio; OA PSV, ophthalmic artery systolic velocity ratio; PlGF, placental groeth factor; PIERS, Pre-eclampsia Integrated Estimate of RiSk; sFlt-1, soluble fms-like tyrosine kinase-1; | | | | | |

**Supplementary table 4. Example and full script in R language used to calculate effect measures**

| ################################################################################  # Systematic Review Short Demo.R  #  # Description:  # Demo code to calculate sensitivity, specificity, AUC, and positive and negative  # likelihood ratios with 95% confidence intervals.  #  # Date: 2024-01-24  #  ################################################################################  # Clean-up.  rm(list = ls())  gc()  cat("\14")  # Init --------------------------------------------------------------------  # Make sure required packages are installed.  # install.packages('epiR')  # install.packages('pROC')  # Load packages.  library("epiR")  library("pROC")  # Helper function to calculate ROC AUC with 95% CI.  calculate_auc <- function(TP, FP, FN, TN) {  df_TP <- matrix(rep(c(1,1), TP), ncol=2, byrow=TRUE)  df_FP <- matrix(rep(c(0,1), FP), ncol=2, byrow=TRUE)  df_FN <- matrix(rep(c(1,0), FN), ncol=2, byrow=TRUE)  df_TN <- matrix(rep(c(0,0), TN), ncol=2, byrow=TRUE)    df <- as.data.frame(rbind(df_TP, df_FP, df_TN, df_FN))  colnames(df) <- c('outcome','prediction')    auc <- roc(df$outcome, df$prediction,levels=c(0, 1), direction="<")  ci <- ci.auc(auc)    return(data.frame(est = ci[2], lower = ci[1], upper = ci[2],  label = "ROC AUC: ",  result = sprintf("%.2f, (%.2f, %.2f)", ci[2], ci[1], ci[3])))    }  # Analysis ----------------------------------------------------------------  # Input data.  # Change FP,TP,FN and TN as needed.  input <- data.frame(TP = 4, FP = 122, TN = 15, FN = 0)  # In case any cell is zero:  # Add 0.5 pseudo-count to make positive or negative likelihood ratio well-defined.  # input <- data.frame(TP = 4, FP = 122, TN = 15, FN = 0) + 0.5  # Confidence intervals for sensitivity, specificity, positive and negative  # predictive value will be calculated using the Wilson's score method.  # Confidence intervals for the positive and negative likelihood ratios will be  # calculated # using Simel's method (default in epi.tests).  res <- epi.tests(c(input$TP, input$FP, input$FN, input$TN),  method = "wilson",  digits = 2,  conf.level = 0.95)  # Print results  print(res)  cat("\n")  # Calculate AUC with 95% CI.  auc <- calculate_auc(input$TP, input$FP, input$FN, input$TN)  print(auc[, c("label", "result")], row.names = FALSE) |
| --- |
| # Needed packages  install.packages('epiR')  install.packages('pROC')  library(epiR)  library(pROC)  # Write your input like this but with your values instead of FP,TP,FN and TN.  # vector <- c(FP,TP,FN,TN)  vec <- data.frame(FP = 122, TP = 4, FN = 0, TN = 15)  # After you double checked that your input is in the right order click on run until  # you reach the end of this script.  rownames <- c('AUC', 'LR+', 'LR-','Sensitivity', 'Specificity',  'PPV', 'NPV')  colnames <- c('Estimate','Lower bound', 'Upper bound')  Result <- data.frame(matrix(data = NA, nrow = 7, ncol = 3),  row.names = rownames)  colnames(Result) <- colnames  data.AUC <- \(TP,FP,TN,FN){  # Create a function that creates a data frame of binary columns stating the  # outcome and prediction. Used to roc and AUC. 1 if it has happend/tested positive. 0 otherwise.    dff.AUC <- data.frame(matrix(ncol = 2, nrow = 0))  TP.vec <- matrix(rep(c(1,1), TP), ncol=2, byrow=TRUE)  FP.vec <- matrix(rep(c(0,1), FP), ncol=2, byrow=TRUE)  TN.vec <- matrix(rep(c(0,0), TN), ncol=2, byrow=TRUE)  FN.vec <- matrix(rep(c(1,0), FN), ncol=2, byrow=TRUE)    dff.AUC <- rbind(dff.AUC, TP.vec)  dff.AUC <- rbind(dff.AUC, FP.vec)  dff.AUC <- rbind(dff.AUC, TN.vec)  dff.AUC <- rbind(dff.AUC, FN.vec)    colnames(dff.AUC) <- c('outcome','prediction')  return(dff.AUC)  }  # create matrix with two columnes, outcome & prediction which are full of 0 & 1  # dependent on TP, FP, TN and FN  df.AUC <- data.AUC(vec$TP,vec$FP,vec$TN,vec$FN)  #create confusion matrix in the right order in the right form  conf.matrix <- c(vec$TP,vec$FP,vec$FN,vec$TN)  # Confidence intervals for sensitivity, specificity, positive and negative  # predictive value will be calculated using the Wilson's score method. Confidence  # intervals for the positive and negative likelihood ratios will be calculated  # using Simel's method, by default of epi.tests.  epi.test.row <- epi.tests(conf.matrix, method = "wilson", digits = 2, conf.level = 0.95)$detail  # The AUC will be calculated using the trapezoidal rule. Confidence intervals  # will be calculated using the method of DeLong.  rocs <- roc(df.AUC$outcome, df.AUC$prediction,levels=c(0, 1), direction="<")  Result['AUC','Lower bound'] <- sprintf("%.3f", ci.auc(rocs)[1])  Result['AUC','Estimate'] <- sprintf("%.3f", ci.auc(rocs)[2])  Result['AUC','Upper bound'] <- sprintf("%.3f", ci.auc(rocs)[3])  #Get and set values from epi.tests for each row/each study into result table  Result['LR+','Estimate'] <- sprintf("%.3f", epi.test.row[11,2])  Result['LR+','Lower bound'] <- sprintf("%.3f", epi.test.row[11,3])  Result['LR+','Upper bound'] <- sprintf("%.3f", epi.test.row[11,4])  Result['LR-','Estimate'] <- sprintf("%.3f", epi.test.row[12,2] )  Result['LR-','Lower bound'] <- sprintf("%.3f", epi.test.row[12,3])  Result['LR-','Upper bound'] <- sprintf("%.3f", epi.test.row[12,4])  Result['Sensitivity', 'Lower bound'] <- sprintf("%.3f", epi.test.row[3,3])  Result['Sensitivity', 'Estimate'] <- sprintf("%.3f", epi.test.row[3,2])  Result['Sensitivity', 'Upper bound'] <- sprintf("%.3f", epi.test.row[3,4])  Result['Specificity', 'Lower bound'] <- sprintf("%.3f", epi.test.row[4,3])  Result['Specificity', 'Estimate'] <- sprintf("%.3f", epi.test.row[4,2])  Result['Specificity', 'Upper bound'] <- sprintf("%.3f", epi.test.row[4,4])  Result['PPV', 'Lower bound'] <- sprintf("%.3f", epi.test.row[9,3])  Result['PPV', 'Estimate'] <- sprintf("%.3f", epi.test.row[9,2])  Result['PPV', 'Upper bound'] <- sprintf("%.3f", epi.test.row[9,4])  Result['NPV', 'Lower bound'] <- sprintf("%.3f", epi.test.row[10,3])  Result['NPV', 'Estimate'] <- sprintf("%.3f", epi.test.row[10,2])  Result['NPV', 'Upper bound'] <- sprintf("%.3f", epi.test.row[10,4])  # Firth correction using the result directly from the calculation since  # the result in our Result table is in the wrong format.  if (is.na(epi.test.row[11,3]) || is.na(epi.test.row[11,4]) ||  is.na(epi.test.row[12,3]) || is.na(epi.test.row[12,4])){  # If any positive or negative likelihood ratio bounds is NaN, then do a firth  # correction and mark it with *    # firth correction with "+0.5"  conf.matrix <- c(vec$TP + 0.5, vec$FP + 0.5,  vec$FN + 0.5, vec$TN + 0.5)  epi.test.row <- epi.tests(conf.matrix, method = "wilson", digits = 2, conf.level = 0.95)$detail    Result['LR+','Estimate'] <- sprintf("%f*", epi.test.row[11,2])  Result['LR+','Lower bound'] <- sprintf("%f*", epi.test.row[11,3])  Result['LR+','Upper bound'] <- sprintf("%f*", epi.test.row[11,4])    Result['LR-','Estimate'] <- sprintf("%f*", epi.test.row[12,2])  Result['LR-','Lower bound'] <- sprintf("%f*", epi.test.row[12,3])  Result['LR-','Upper bound'] <- sprintf("%f*", epi.test.row[12,4])  }  #Finished!  View(Result) |

**Supplementary table 5: Table of all included studies**

|  | **Population** | **Study Design** | **Dates** | **Outcome(s)** | **Participants** | **Predictor(s)** | **Prediction method** |
| --- | --- | --- | --- | --- | --- | --- | --- |
| **Argentina** | | | | | | | |
| Vasquez et al (2021) | women with HDP | multi-center prospective cohort study | 2012–12 | maternal:  eclampsia HELLP | 172 | headache  hyperreflexia epigastric pain  nausea and vomiting visual symptoms  right upper quadrant pain  renal disturbances | univariable |
| **Australia** | | | | | | | |
| Paul et al (2019) | women with pre-eclampsia | single-center retrospective cohort study | 2015–17 | maternal composite:  SBP ≥ 160 mm Hg or DBP ≥ 110 mm Hg on two occasions thrombocytopenia (platelet count < 100,000/microliter) impaired liver function (abnormally elevated liver enzymes to twice the normal concentration) progressive renal insufficiency (elevated serum creatinine concentration) pulmonary oedema new onset cerebral/visual disturbance ICU admission PPH | 124 | multivariable model:  SBP (> 125 mmHg) essential hypertension hemoglobin (> 70 g/L) serum creatinine concentration (>30 µmol/L) | multivariable |
| Reddy et al (2022) | women with HDP | single-center prospective prognostic accuracy study | 2018–20 | maternal composite:  need for parenteral antihypertensive agents ICU admission eclampsia placental abruption HELLP DIC platelets < 100×10^9/L creatinine > 90μmol/L ALT > 100 U/L   fetal composite:  preterm birth < 34 weeks NICU admission  RDS IVH HIE NEC confirmed fetal infection retinopathy of prematurity | 126 | sFlt-1/PlGF ratio serum PlGF concentration serum sFlt-1 concentration  analytic platform: BRAHMS Kryptor compact plus (Thermo Scientific) EFW percentile  Doppler assessment: UtA-PI AC OA PSV ratio  AC percentile UA-PI percentile MCA-PI percentile CPR percentile | univariable; multivariable |
| **Austria** | | | | | | | |
| Binder et al (2021) | women with chronic hypertension and/or super-imposed pre-eclampsia | retrospective analysis of single-center prospectively collected data | 2013–19 | maternal: ICU admission pulmonary oedema renal insufficiency (creatinine levels > 1.7 mg/dL) liver dysfunction (elevated liver enzymes with transaminase levels twice the upper limit of normal)  fetal:  stillbirth or early neonatal death  RDS ventilation support NICU admission IVH | 145 | angiogenic imbalance  analytic platform: Elecsys platform (Roche Diagnostics) proteinuria IUGR other symptoms | multivariable |
| Binder et al (2023) | women with pre-eclampsia | single-center retrospective cohort study | 2013–20 | maternal composite:  ICU admission (due to severe hypertension with the need for continuous blood pressure monitoring, liver dysfunction defined as elevated liver enzymes with transaminase levels twice the upper limit of normal or acute kidney injury determined as creatinine concentration > 1.0 mg/dL) pulmonary oedema eclampsia death  fetal composite:  stillbirth neonatal death IVH retinopathy of prematurity NEC | 323 | sFlt-1/PlGF ratio  analytic platform: Elecsys platform  (Roche Diagnostics) serum creatinine concentration  serum transmainase concentration LDH  thrombocytopenia | univariable |
| Stolz et al (2018) | women with pre-eclampsia | single-center retrospective cohort study | 2013–16 | maternal: ICU admission  fetal:  neonatal death fetal death/stillbirth SGA RDS NICU admission | 60 | sFlt-1/PlGF ratio  analytic platform: Elecsys platform  (Roche Diagnostics) | univariable |
| **Brazil** | | | | | | | |
| Chaves et al (2017) | women with pre-eclampsia | single-center prospective cohort study | 2014–15 | maternal composite and single: HELLP ICU admission CNS injury (PRES) maternal death hypertensive crisis (SBP ≥ 160 mmHg or DBP ≥ 110 mmHg)  fetal composite and single:  neonatal death fetal death/stillbirth preterm birth < 32 weeks SGA FGR  NICU admission neonatal acidemia (pH <7.0 and base deficit ≥2 mmol/L) 5-min Apgar score < 7 | 58 | Doppler assessment: abnormal OA PR | univariable |
| Guida et al (2020) | women with pre-eclampsia | single-center cross-sectional study | 2017–18 | maternal PIERS composite:  eclampsia  HELLP  placental abruption  maternal haemorrhage and the need for blood transfusion pulmonary oedema stroke or other central nervous system bleedings renal or hepatic failure maternal death | 208 | fullPIERS model:  gestational age chest pain or dyspnea SpO2 platelet count serum creatinine serum AST | multivariable |
| Nóbrega et al (2022) | women with severe pre-eclampsia | single-center cohort study | 2015–17 | maternal PIERS composite: maternal death eclampsia stroke Bell’s palsy pulmonary oedema PPH platelet count < 50x10^9/L hepatic dysfunction acute renal insufficiency placental abruption | 78 | serum hs-CRP concentration | univariable |
| **China** | | | | | | | |
| Cai et al (2021) | women with pre-eclampsia | single-center retrospective cohort study | 2017–18 | maternal: HELLP   fetal: iatrogenic prematurity (delivery < 37 weeks) | 102 | urinary congophilia (positive CapCord test) | univariable |
| Chen et al (2022) | women with HDP | single-center retrospective cohort study | 2012–19 | maternal PIERS composite: HELLP  eclampsia cerebrovascular complications placental abruption acute kidney injury pulmonary oedema liver dysfunction DIC death ICU admission  fetal composite: preterm birth FGR SGA  NICU admission low Apgar scores (<7) neonatal death | 1829 | machine learning algorithm multivariable models | multivariable |
| He et al (2022) | women with HDP | single-center prospective cohort study | 2018–21 | fetal composite:  low birth weight  birth asphyxia  SGA premature delivery NICU admission perinatal death Low 1 min Apgar score (> 7) | 196 | relative expression of miRNA-204 in serum | univariable |
| Hong et al (2021) | women with pre-eclampsia | single-center retrospective cohort study | 2017–19 | maternal composite:  eclampsia placental abruption hemolysis abnormal liver function (the liver enzyme > 3 times the normal value, and the normal value is about 40 U/L) HELLP cardiac insufficiency  acute renal insufficiency (creatinine > 150 μmol/L) central nervous system complications  body cavity effusion  pulmonary hypertension   fetal composite:  SGA  fetal distress fetal death stillbirth oligohydramnios neonatal asphyxia | 284 | B-type natriuretic peptide  multivariable model:  age  hemoglobin BMI proteinuria | univariable; multivariable |
| Lei et al (2021) | women with pre-eclampsia | single-center retrospective cohort study | 2015–20 | maternal composite and single:  Organ dysfunction (kidney, liver or heart failure) eclampsia HELLP  hypoproteinemia pleural or peritoneal effusion pericardial effusion oligohydramnios placental abruption death retinal disease DIC PPH  fetal composite and single:  death stillbirth low birth weight preterm delivery ( <37 weeks) induced labor FGR fetal distress neonatal asphyxia HIE fetal malformation | 275 | 24-h proteinuria excretion | univariable |
| Li et al (2018) | women with HDP | multi-center retrospective cohort study | 2015–15 | maternal composite and single:  maternal death  eclampsia pulmonary oedema renal insufficiency placental abruption heart failure elevated liver enzyme severe hypoproteinemia (serum albumin < 25 g/L) | 1738 | 24 h protein excretion   multivariable model:  24 h protein excretion (≥ 2.0 g/24 h) serum creatinine concentration (> 97.25 µmol/L) serum AST (> 40 U/L) thrombocytopenia  hypoproteinemia  upper abdominal pain palpitations or dyspnea gestational age < 34 weeks | univariable; multivariable |
| Li et al (2019) | women with gestational hypertension and SLE | single-center retrospective cohort study | 2015–18 | fetal composite: SGA intrauterine distress  premature delivery (< 37 weeks GA) neonatal asphyxia | 124 | Doppler assessment:  UA PI UA RI UA S/D | univariable |
| Ma et al (2019) | women with HELLP | single-center retrospective cohort study | 2012–18 | maternal:  postpartum haemorrhage (blood loss >/= 500 ml vaginal delivery or </= 1000 ml CS)   fetal composite:  fetal death  fetal distress (abnormal fetal movement; abnormal fetal heart rate before and during delivery; fetal biophysical score ≤ 4 points; abnormal fetal Doppler ultrasound blood flow; fetal acidosis) | 127 | prothrombin time  activated partial thromboplastin time  serum fibrinogen  serum D-dimer | univariable |
| Tan et al (2020) | women with HDP | single-center retrospective cohort study | 2005–14 | maternal composite: death near-miss (series of organ dysfunction, including respiratory dysfunction, cardiovascular dysfunction, renal dysfunction, coagulation/blood dysfunction, liver function disorder, neurological dysfunction, and uterine dysfunction) cortical blindness/retinal detachment Bell’s palsy | 2793 | multivariable models: gestational age placenta previa HBsAg positivity cardiac diseases IDA dyspnea systolic blood pressure at admission log-transformed platelets log-transformed fibrinogen log-transformed aspartate transferase log-transformed total bilirubin log-transformed creatinine qualitative proteinuria | multivariable |
| Wang et al (2019) | women with HDP | multi-center retrospective cohort study | 2007–12 | maternal PIERS composite: maternal mortality hepatic dysfunction  hematoma, or rupture one or more seizures of eclampsia Glasgow coma score < 13 stroke reversible ischemic neurological deficit transient ischemic attack PRES cortical blindness or retinal detachment need positive inotrope support infusion of a third parenteral antihypertensive myocardial ischemia or infarction acute renal insufficiency or failure dialysis pulmonary oedema, SpO2 < 90%, requirement of ≥ 50% fractional inspired oxygen (FiO2) for more than one hour intubation transfusion of any blood product severe thrombocytopenia (< 50*10^9/l) in the absence of blood transfusion placental abruption three or more kinds of antihypertensive drugs | 1430 | fullPIERS model:  gestational age chest pain or dyspnea SpO2 platelet count serum creatinine serum AST | univariable; multivariable |
| Wei et al (2022) | women with pre-eclampsia | single-center retrospective cohort study | 2019–21 | maternal composite and single:  HELLP  ICU admission respiratory failure (serious disorder of pulmonary ventilation and/or ventilation function caused by various reason) PPH  placental abruption  heart failure  hypertensive retinopathy CKD (decline of renal function, which is manifested by GFR lower than 60 ml/min per 1.73 m^2) | 733 | prognostic nutritional index (PNI) | univariable |
| Wu et al (2017) | women with severe pre-eclampsia | single-center prospective cohort study | 2015–16 | fetal:  perinatal death NICU admission  five-minute Apgar score < 7 | 120 | serum gamma glutamyl transferase  Doppler assessment: UA S/D ratio | univariable; multivariable |
| Ye et al (2020) | women with severe pre-eclampsia | single-center retrospective cohort study | 2013–16 | maternal: PPH placental abruption retinopathy stage 3/4 (retinal oedema, haemorrhages, exudates and optical disc oedema)   fetal:  fetal death/stillbirth preterm birth <37 weeks SGA | 534 | retinopathy staging group 3/4 albumin  white blood cell count early onset pre-eclampsia late onset pre-eclampsia severe hypertension  serum ALT  serum AST | univariable |
| Zheng et al (2022) | women with pre-eclampsia | multi-center retrospective cohort study | 2007–17 | maternal composite and single:  maternal mortality eclampsia stroke cortical blindness retinal detachment pulmonary oedema acute kidney injury liver capsule hematoma or rupture placental abruption PPH raised liver enzymes low platelets ICU admission intubation mechanical ventilation  fetal composite and single:  stillbirth preterm birth low birth weight SGA neonatal mortality neonatal seizures NICU admission respiratory support | 733 | machine learning multivariable models | univariable; multivariable |
| **Egypt** | | | | | | | |
| Alanwar et al (2018) | women with severe pre-eclampsia | single-center prospective cohort study | 2017–17 | fetal:  NICU admission | 100 | Doppler assessment: CPR | univariable |
| El-Demiry et al (2020) | women with severe pre-eclampsia | single-center prospective cohort study | 2017–19 | fetal composite:  Apgar score at 1 and 5 min SGA RDS NICU admission  perinatal death | 60 | Doppler assessment: UA PI UA RI MCA PI MCA RI CPR DV PVIV DV PSV | univariable |
| Moawad et al (2022) | women with severe pre-eclampsia | single-center prospective cross-sectional study | 2018–19 | fetal composite and single:  IUGR NICU admission 1-and 5-min Apgar score < 7 metabolic acidosis at birth  low birth weight (< 2500g) | 60 | Doppler assessment: UA PI UA RI  UA S/D MCA PI MCA RI  MCA S/D  CPR | univariable |
| **France** | | | | | | | |
| Boutot et al (2020) | women with pre-eclampsia and/or HELLP | single-center retrospective cohort study | 2014–18 | maternal PIERS composite:  eclampsia  coma (Glasgow score < 13)  stroke  PRES  retinal detachment  positive inotropic support  myocardial infarction  parenteral antihypertensive treatment  SpO2 < 90%  FiO2 > 50%  thrombocytopenia < 50 000 platelets/L  blood transfusion  hepatic failure  subcapsular hepatic hematoma  renal failure (creatinine > 150 µmol/L), acute renal failure (creatinine > 200 µmol/L), Dialysis  ascites  placental abruption  Bell's Palsy  HELLP  maternal death | 217 | fullPIERS model:  gestational age chest pain or dyspnea oxygen saturation (SpO2) platelet count serum creatinine serum AST | multivariable |
| **Germany** | | | | | | | |
| Graupner et al (2019) | women with pre-eclampsia and/or HELLP | single-center retrospective cohort study | 2010–18 | fetal composite and single:  pH value umbilical cord artery ≤ 7.0 and/or base excess ≤ -12 mmol/L  5min Apgar score ≤ 7 NICU admission RDS respiratory support > 4 h SGA | 67 | sFlt/PIGF ratio  analytic platform: Electrochemiluminescence immunoassay (Roche diagnostics)  Doppler assessment:  UA-PI MCA-PI CPR UtA-PI | univariable; multivariable |
| Karge et al (2021) | women with pre-eclampsia and/or HELLP in twin pregnancy | single-center retrospective cohort study | 2010–18 | fetal composite:  NICU admission  intubation of the newborn RDS | 49 | sFlt-1/PlGF ratio  analytic platform: Electrochemiluminescence immunoassay (Roche diagnostics) | univariable |
| Karge et al (2022) | women with pre-eclampsia and/or HELLP | single-center retrospective cohort study | 2018–20 | maternal composite:  eclampsia pulmonary oedema acute kidney injury (defined as elevated serum creatinine greater than 1.1 mg/dL or a doubling of serum creatinine in the absence of other renal disease) placental abruption HELLP  ICU admission need of intubation mechanical ventilation   fetal composite:  SGA neonatal mortality neonatal seizures NICU admission respiratory support | 141 | sFlt-1/PIGF ratio  analytic platform: Electrochemiluminescence immunoassay (Roche diagnostics) pre-pregnancy BMI | univariable |
| **India** | | | | | | | |
| Abraham et al (2019) | women with HDP | single-center cross-sectional study | NA | fetal: preterm birth < 37 weeks low birth weight (less than 2500 g)  SGA fetal mortality (intra-uterine and perinatal deaths) | 174 | TOH SBP DBP serum ADMA  serum MDA  TAS | univariable; multivariable |
| Ahmad et al (2023) | women with pre-eclampsia | single-center prospective cohort study | 2020–21 | maternal PIERS composite:  eclampsia placental abruption thrombocytopenia acute renal failure HELLP pulmonary oedema postpartum haemorrhage thromboembolic episodes cortical blindness ICU admission maternal death  fetal composite:  prematurity  Apgar score < 4 at the time of birth Meconium-stained liquor NICU admission neonatal death intrauterine death | 384 | fullPIERS model:  gestational age chest pain or dyspnea oxygen saturation (SpO2) platelet count serum creatinine serum AST | univariable; multivariable |
| Chadha et al (2022) | women with pre-eclampsia | single-center prospective observational study | 2019–21 | maternal composite and single:  severe hypertension (≥ 160/110mmHg) Renal insufficiency (serum creatinine >1.2mg/dL) or oliguria (<400mg/dL) increased liver enzyme (AST > 40u/l) thrombocytopenia (platelet count < 150x10^9/l) HELLP  placental abruption Eclampsia ICU admission  Neurological involvement  Maternal death   fetal:  preterm delivery < 37 weeks FGR Low birth weight (< 2500 g) Intrauterine death  Neonatal death | 152 | urinary protein to creatinine ratio (UPCR) | univariable |
| Gupta et al (2017) | women with gestational hypertension | single-center case-control study, descriptive type of observational study | 2014–15 | fetal composite and single:  low birth weight perinatal death  emergency lower-segment cesarean section for fetal distress low 5 min Apgar score (< 7) NICU admission | 100 | Doppler assessment:  UtA UA  MCA | univariable |
| Jampana et al (2022) | women with HDP | single-center unknown study design | 2021–22 | maternal:  eclampsia placental abruption  PPH HELLP  severe renal failure maternal death   fetal:  preterm birth birth weight < 2500g perinatal death | 86 | serum uric acid concentration (> 5.5 mg/dL) | univariable |
| Joshi et al (2022) | women with HDP | single-center prospective cohort study | NA (3 years) | maternal composite and single:  placental abruption acute liver injury acute kidney injury DIC  HELLP neurological symptoms (including eclampsia)   fetal composite and single:  non-stress test abnormalities meconium-stained liquor  birth weight < 2500g preterm birth < 37 weeks NICU admission intrauterine fetal demise neonatal mortality | 132 | serum hs-CRP concentration | univariable |
| Kesireddy et al (2021) | women with gestational hypertension | single-center retrospective observational case-control study | NA | maternal:  abruption uncontrolled HTN requiring intravenous Labetalol abnormal PE profile imminent eclampsia HELLP   fetal:  gestational age at birth birth weight  static growth Doppler flow abnormalities CTG abnormalities neonatal mortality FGR  intra uterine fetal death | 51 | glycosylated fibronectin (GlyFn) | univariable |
| Kumar et al (2023) | women with pre-eclampsia | single-center prospective cohort study | 2020–22 | maternal composite:  intravenous antihypertensive agents ICU admission eclampsia placental abruption HELLP DIC platelets <100x10^9/L creatinine >1.1 mg/dL ALT >100 U/L stroke cortical blindness acute pulmonary oedema liver capsule rupture or hematoma maternal death  fetal composite:  preterm birth at ≤34 week  NICU admission RDS hypoxic ischemic encephalopathy (HIE) NEC retinopathy of prematurity neonatal seizures perinatal death | 91 | MAP (≥ 127.85 mmHg) serum sFlt-1 concentration  serum PlGF concentration  sFlt-1/PlGF ratio serum creatinine concentration  serum uric acid concentration  LDH concentration | univariable |
| Kumari et al (2017) | women with pre-eclampsia | single-center prospective observational study | 2015–16 | maternal composite:  eclampsia congestive heart failure HELLP pulmonary oedema cerebrovascular accident  renal dysfunction hypertensive retinopathy placental abruption | 45 | N terminal pro-Brain Natriuretic Peptide | univariable |
| Madhu et al (2023) | women with gestational hypertension | single-center prospective cohort study | 2017–19 | fetal:  birth weight < 2500g preterm delivery  IUGR NICU admission | 150 | Doppler assessment:  UA S/D ratio UA RI UA PI MCA PI CPR (< 1.08) UA REDF | univariable |
| Malik et al (2023) | women with HDP (except chronic hypertension) | single-center prospective observational study | NA | fetal composite and single:  cesarean section for fetal distress instrumental delivery for fetal distress SGA (birth weight less than 10th percentile for gestational age) meconium stained liquor APGAR score < 7 at 1 and 5 minutes NICU admission stillbirth/perinatal death | 100 | Doppler assessment: UA PI (> 95th percentile) MCA PI (< 5th percentile) MCA S/D ratio (< 3) UA S/D ratio (> 3) CRP (< 1.08) | univariable |
| Murali et al (2023) | women with severe pre-eclampsia | single-center prospective cohort study | 2019–21 | maternal composite:  eclampsia hypertensive urgency placental abruption pulmonary oedema HELLP acute renal failure PRES  fetal:  FGR stillbirth 5 Apgar score <7 NICU admission respiratory distress hypoxic Ischemic encephalopathy intracranial bleed sepsis neonatal death | 202 | 24h protein secretion UPCR | univariable |
| Nayak et al (2022) | women with HDP | single-center prospective observational cohort study | NA (2 years) | fetal composite and single:  delivery by lower segment cesarean section or vaginal delivery low birth weight  Apgar scores 1 and 5 min NICU admission neonatal complications (hypoglycemia, polycythemia, neonatal asphyxia, acidosis, neonatal sepsis) ventilation neonatal death | 65 | Nonstress Test (NST)  Doppler assessment:  CRP | univariable |
| Saxena et al (2021) | women with HDP | single-center prospective cohort study | 2018–20 | fetal composite and single:  cesarean section for fetal distress  low birth weight preterm birth Meconium-stained Liquor  Apgar score 5min < 7 stillbirth  NICU admission  early neonatal death | 150 | estimated fetal weight < 2.5 kg Doppler assessment: CPR | univariable; multivariable |
| Sharma et al (2023) | women with severe pre-eclampisa | single-center prospective cohort study | 2021–22 | maternal PIER composite:  maternal death hepatic dysfunction (hematoma/rupture/failure) eclampsia stroke RIND transient ischemic attack  PRES cortical blindness/retinal detachment need for positive inotropic/vasopressors/third parenteral antihypertensive support myocardial ischemia/infarction acute renal insufficiency/failure requirement for hemodialysis pulmonary oedema requirement for oxygenation (SpO2 below 90%/fraction of inspired oxygen [FiO2] of ≥50% for >1 h/invasive ventilation) transfusion of blood products severe thrombocytopenia without blood transfusion placental abruption | 256 | fullPIERS model: gestational age chest pain or dyspnea oxygen saturation (SpO2) platelet count serum creatinine serum AST | multivariable |
| **Iran** | | | | | | | |
| Zarean et al (2022) | women with HDP | single-center descriptive-comparative study | NA | fetal composite and single:  stillbirth neonatal death cesarean delivery because of fetal distress NICU admission 5 min Apgar score < 7  IUGR birth weight respiratory problems acidemia seizure SGA  requirement of assisted aspiration | 100 | Doppler assessment: CPR | univariable |
| **Italy** | | | | | | | |
| Loardi et al (2021) | women with HDP | single-center retrospective cohort study | 2011–17 | maternal composite and single:  HELLP acute kidney failure pulmonary oedema neurologic complications DIC placental abruption  fetal composite and single:  stillbirth neonatal death RDS IVH sepsis NEC FGR | 494 | timepoint of diagnosis of pre-eclampsia Doppler assessment: UtA PI | univariable |
| **Japan** | | | | | | | |
| Mayama et al (2021) | women with pre-eclampsia | multi-center retrospective cross-sectional study | 2010–19 | maternal:  organ damage   fetal composite and single:  NICU admission preterm delivery < 34 weeks utero-placental dysfunction (fetal growth restriction, abnormal artery Doppler waveform or stillbirth) | 264 | mild thrombocytopenia (≥ 100 x 10^9/L and < 150 x 10^9/L platelet count) severe thrombocytopenia (< 100 x 10^9/L platelet count) | univariable |
| Morikawa et al (2020) | women with pre-eclampsia | single-center retrospective cohort study | 2009–17 | maternal:  pulmonary oedema placental abruption peripartum cardiomyopathy HELLP /acute fatty liver of pregnancy  Eclampsia/ PRES central serous chorioretinopathy   fetal:  Stillbirth or early neonatal death | 94 | serum total protein at pre-eclampsia diagnosis and delivery | univariable |
| Morikawa et al (2021) | women with pre-eclampsia | single-center retrospective cohort study | 2009–17 | maternal composite and single:  pulmonary oedema placental abruption peripartum cardiomyopathy HELLP/ Acute fatty liver of pregnancy eclampsia/PRES central serous chorioretinopathy  maternal death ICU admission blood transfusion  fetal:  stillbirth or early neonatal death  NICU admission ventilation performed | 94 | gestational weight gain during the week prior to delivery | univariable |
| **Korea** | | | | | | | |
| Ryu et al (2019) | women with pre-eclampsia | single-center prospective observational study | 2015–16 | fetal:  low birth weight  preterm labor SGA | 65 | serum uric acid hemoglobin serum total bilirubin | univariable; multivariable |
| **Mexico** | | | | | | | |
| Cazarez-Ávalos et al (2019) | women with pre-eclampsia | single-center retrospective cohort study | 2018–19 | maternal PIERS composite and single: HELLP palcental abruption  pulmonary oedema DIC  renal insufficiency  subcapsular hematoma | 100 | fullPIERS model: gestational age chest pain or dyspnea oxygen saturation (SpO2) platelet count serum creatinine serum AST | mulitvariable |
| Leanos-Miranda et al (2020) | women with pre-eclampsia | single-center unknown study design | 2015–18 | maternal composite and single:  thrombocytopenia (Platelet count <100x10^3 μl) abnormal liver enzymes levels (to twice normal concentration) hepatic hematoma or rupture  pulmonary oedema  need for positive inotropic support  intubation  acute kidney injury (Creatinine >1.5 mg/dL) placental abruption  cerebral haemorrhage maternal death  fetal:  preterm delivery at <34 weeks preterm delivery at <37 weeks IVH fetal/neonatal death SGA | 810 | sFlt-1/PlGF ratio  analytic platform: Elecsys platform  (Roche Diagnostics) | univariable |
| **Multinational** | | | | | | | |
| Magee et al (2016) | women with chronic or gestational hypertension | retrospective analysis of multi-center randomized controlled trial (CHIPS trail cohort in 15 countries) | 2009–14 | maternal composite:  death stroke eclampsia blindness uncontrolled hypertension the use of inotropic agents pulmonary oedema, respiratory failure myocardial ischemia or infarction,  hepatic dysfunction hepatic hematoma or rupture renal failure transfusion  fetal: pregnancy loss NICU admission birthweight <10th percentile preterm delivery at <34 or <37 weeks | 987 | multivariable model and single:  less tight or tight controlled BP maternal age  Ethnicity  BMI  conceived through ART gestational age at randomization nulliparity type of hypertension (preexisting/gestational)  prior severe hypertension in this pregnancy, antihypertensive therapy at randomization type of antihypertensive therapy at randomization  SBP (mmHg) within 1 week before randomization DBP within 1 week before randomization  in hospital at enrolment gestational diabetes at randomization cigarette smoking during this pregnancy aspirin at enrolment folic acid and/or a PNV at enrolment perinatal mortality ratio of recruiting country | multivariable |
| Magee et al (2021) | women with chronic or gestational hypertension | retrospective analysis of multi-center randomized controlled trial (CHIPS trail cohort in 15 countries) | 2009–14 | maternal composite:  death  stroke eclampsia blindness uncontrolled hypertension use of inotropic agents pulmonary oedema respiratory failure myocardial ischemia or infarction hepatic dysfunction hepatic hematoma or rupture renal failure transfusion severe hypertension admission to hospital before delivery  fetal composite and single:  stillbirth  neonatal death NICU admission low birth weight (<19th centile) preterm delivery <34 weeks or 37 weeks | 987 | Pre-eclampsia definitions:  Group A: development of proteinuria  Group B: development of proteinuria and one or more symptoms, signs, abnormal laboratory test Group C: one or more symptoms, signs Group D: one or more symptoms, signs, abnormal laboratory test Group E: development of proteinuria or one or more symptoms, signs, abnormal laboratory test Group F: development of proteinuria or one or more symptoms, signs | multivariable |
| Ukah et al (2017) | women with HDP | retrospective analysis of multi-center prospectively collected data (miniPIERS cohort in 5 countries: Fiji, Uganda, South Africa, Brazil, and Pakistan) | 2008–12 | maternal PIERS composite:  Glasgow coma scale <13  stroke cortical blindness RIND retinal detachment acute renal insufficiency dialysis platelet count < 50 000 without blood transfusion  transfusion of blood products positive inotropic support  myocardial ischemia/infarction require > 50% oxygen for greater than one hour intubation  pulmonary oedema eclampsia (eclamptic seizures) Bell's palsy severe ascites hysterectomy | 757 | fullPIERS model:  gestational age chest pain or dyspnea oxygen saturation (SpO2) platelet count serum creatinine serum AST | multivariable |
| Ukah et al (2018_1) | women with pre-eclampsia | retrospective analysis of multi-center prospectively collected data (Canada: BCW cohort, Netherlands: Dutch PETRA cohort, UK: PREP cohort) | 2000–03; 2011–14 | maternal PIERS composite:  Glasgow coma scale <13  stroke cortical blindness RIND retinal detachment acute renal insufficiency dialysis platelet count < 50 000 without blood transfusion  transfusion of blood products positive inotropic support  myocardial ischemia/infarction require > 50% oxygen for greater than one hour intubation  pulmonary oedema eclampsia (eclamptic seizures) Bell's palsy severe ascites hysterectomy | 954 | fullPIERS model:  gestational age chest pain or dyspnea oxygen saturation (SpO2) platelet count serum creatinine serum AST | multivariable |
| Ukah et al (2018_2) | women with pre-eclampsia or suspected pre-eclampsia | retrospective analysis of multi-center prospectively collected data (Canada: BCW cohort, US: PETRA cohort, Finland: FINNPEC cohort, UK: PELICAN cohort and Oxford cohort | 2012–16; 2008–11; 2011-12; 2011–12; 2003–06 | maternal PIERS composite:  maternal mortality hepatic dysfunction hepatic hematoma or rupture  Glasgow coma scale <13  stroke cortical blindness RIND retinal detachment acute renal insufficiency dialysis platelet count < 50 000 without blood transfusion  transfusion of blood products positive inotropic support  myocardial ischemia/infarction require > 50% oxygen for greater than one hour intubation  pulmonary oedema | 2429 | fullPIERS model:  gestational age chest pain or dyspnea oxygen saturation (SpO2) platelet count serum creatinine serum AST or ALT | multivariable |
| Ukah et al (2020) | women with pre-eclampsia | retrospective analysis of multi-center prospectively collected data (US: PETRA cohort) | 2010–12 | maternal PIERS outcome: maternal mortality hepatic dysfunction hepatic hematoma or rupture  Glasgow coma scale <13  stroke cortical blindness RIND retinal detachment acute renal insufficiency dialysis platelet count < 50 000 without blood transfusion  transfusion of blood products positive inotropic support  myocardial ischemia/infarction require > 50% oxygen for greater than one hour intubation  pulmonary oedema | 541 | serum PlGF concentration  analytic platform: Triage PlGF Test (Quidel)  fullPIERS model:  gestational age chest pain or dyspnea oxygen saturation (SpO2) platelet count serum creatinine serum AST | multivariable |
| **Poland** | | | | | | | |
| Tousty et al (2022) | women with pre-eclampsia | single-center retrospective cohort study | 2018–20 | fetal composite and single:  congenital or late-onset infections RDS PDA NEC IVH Retinopathy of prematurity  Bronchopulmonary dysplasia  neonatal death | 77 | serum sFlt-1 concentration  serum PlGF concentration sFlt-1/PlGF ratio  analytic platform: Cobas e801 (Roche Diagnostics) gestational age | univariable |
| **Russia** | | | | | | | |
| Kurtser et al (2019) | women with severe pre-eclampsia | single-center, unknown study design | 2016-18 | fetal:  perinatal death  perinatal distress (5 min Apgar score </= 7, ph < 7.2, lactate < 4.8 mmol/L) | 28 | serum sFlt-1 concentration serum PlGF concentration sFlt-1/PlGF ratio (≥ 520)  analytic platform: Elecsys Cobas e411 (Roche Diagnostics) | univariable |
| **Serbia** | | | | | | | |
| Mirkovic et al (2020) | women with pre-eclampsia | single-center prospective cohort study | 2013–19 | maternal composite: maternal mortality  or ≥ 1 serious CNS, cardiorespiratory, renal, hepatic or haematological morbidity | 61 | sFlt-1/PlGF ratio  analytic platform: Elecsys platform  (Roche Diagnostics)  fullPIERS model:  gestational age chest pain or dyspnea oxygen saturation (SpO2) platelet count serum creatinine serum AST | univariable; multivariable |
| **South Africa** | | | | | | | |
| Govender et al (2022) | women with severe pre-eclampsia | single-center prospective observational study | 2020–21 | maternal:  placental abruption  HELLP syndrome renal impairment   fetal:  abnormal umbilical artery blood gas (pH <7, base excess <−12) 5-minute APGAR score less than 7 intubation and ventilation meconium aspiration syndrome neonatal seizures IVH periventricular leukomalacia neonatal sepsis | 61 | Doppler assessment: ductus venosus Doppler abnormal | univariable |
| **Spain** | | | | | | | |
| Peguero et al (2023) | women with severe preeclampsia | single-center prospective cohort study | 2017–19 | fetal composite:  stillbirth neonatal death  neonatal metabolic acidosis (umbilical artery pH < 7.0 plus base deficit ≥ -16) 5-min Apgar score < 7 bronchopulmonary dysplasia  NEC  IVH cystic periventricular leukomalacia stage 3–5 retinopathy of prematurity hypoxic ischemic encephalopathy  acute renal failure (serum creatinine >1.5 mg/dL) cardiac failure (requiring inotropic support) | 68 | PREP-L multivariable model (maternal age, maternal pre-existing chronic hypertension, maternal renal disease, maternal diabetes mellitus, maternal autoimmune disease and/or previous history of preeclampsia, systolic BP, urine protein/creatinine ratio, serum urea concentration and platelet count, gestational age, need for antihypertensive treatment or magnesium sulfate)  Doppler assessment:  severe FGR (calculated by the Hadlock formula) UA PI MCA PI  DV PI UtA PI UA AEDV or REDV CPR    serum sFlt-1 concentration serum PlGF concentration  analytic platform: Elecsys platform  (Roche Diagnostics) | univariable; multivariable |
| Simón et al (2020) | women with pre-eclampsia | single-center observational prospective cohort study | 2016–18 | maternal composite:  HELLP neurological events (eclampsia, stroke, cortical blindness, retinal detachment, posterior reversible encephalopathy syndrome) acute myocardial infarction hepatic subcapsular hematoma pulmonary oedema acute renal failure (serum creatinine > 1.2 mg/dL or need for dialysis)  placental abruption maternal death  fetal composite: Apgar at 5 min arterial pH of the umbilical cord NICU admission perinatal death  bronchopulmonary dysplasia  IVH NEC retinopathy of prematurity | 76 | sFlt-1/PlGF ratio  analytic platform: Elecsys platform, Cobas 6000 e701(Roche Diagnostics) Mean blood pressure Urine P/Cr  Serum creatinine  platelet count serum AST  serum PlGF concentration | univariable |
| **Thailand** | | | | | | | |
| Sudjai et al (2022) | women with pre-eclampsia | single-center retrospective cohort study | 2015–19 | maternal:  eclampsia HELLP placental abruption thrombocytopenia renal involvement (serum creatinine concentration greater than 1.1 mg/dL or a doubling of the serum creatinine concentration) caesarean section rate PPH  fetal:  preterm birth low birthweight Apgar score < 7 IUGR RDS NICU admission neonatal death | 400 | serum uric acid concentration | univariable |
| **Turkey** | | | | | | | |
| Tokalioglu et al (2023) | women with preeclampsia | single-center prospective cohort study | 2022–23 | fetal:  NICU admission neonatal death  5 min Apgar score < 7  preterm birth < 34 weeks birth weight < 2500 g intercranial haemorrhage RDS | 55 | Doppler assessment: umbilical artery half peak systolic velocity deceleration time (UA hPSV-DT) <5th percentile | univariable |
| **United Kingdom** | | | | | | | |
| Elia et al (2017) | women with suspected pre-eclampsia | multi-center retrospective cohort study | 2009–12 | maternal composite:  use of intravenous magnesium sulfate  use of intravenous antihypertensives ICU admission placental abruption eclampsia HELLP   fetal composite:  preterm delivery <34 weeks birth weight <5th centile (calculated from sex-specific birth weight centile charts) abnormal umbilical artery Doppler [absent or reversed end-diastolic flow] arterial cord pH<7.1 need for ventilation neonatal or intrauterine death | 717 | ACR gestational age at ACR measurement essential hypertension preexisting diabetes gestational diabetes social deprivation index BMI MAP current smoking status parity maternal age recorded from the clinical record at booking (< 14 weeks) | univariable; multivariable |
| Thangaratinam et al (2017) | women with pre-eclampsia and suspected pre-eclampsia | multi-center retrospective cohort study | 2011–14 | maternal composite:  mortality  hepatic dysfunction DIC  hepatic hematoma or rupture Glasgow coma score <13  stroke  cortical blindness RIND retinal detachment  acute renal insufficiency (serum creatinine >200 uM) dialysis  transfusion of blood products positive ionotropic support  myocardial ischemia/ infarction  require >50% oxygen for greater than one hour intubation  pulmonary oedema PPH early preterm delivery < 34 weeks  fetal composite and single: perinatal or infant mortality  bronchopulmonary dysplasia  NEC  IVH Cystic periventricular leukomalacia  retinopathy of prematurity  HIE  Apgar score ≤ 5 at 10 mins and/or pH 7.00 in first 60 minutes of life and/or Base deficit ≥ -16 in first 60 minutes | 954 | multivariable PREP model (maternal age, maternal pre-existing chronic hypertension, maternal renal disease, maternal diabetes mellitus, maternal autoimmune disease and/or previous history of preeclampsia, systolic BP, urine protein/creatinine ratio, serum urea concentration and platelet count, gestational age, need for antihypertensive treatment or magnesium sulfate) | univariable; multivariable |
| Webster et al (2018) | women with chronic hypertension | multi-center retrospective cohort study | 2000–14 | maternal:  ICU admission  fetal:  birthweight < 10th centile or < 3rd centile  preterm birth < 37 weeks  early preterm birth < 34 weeks  stillbirth NICU admission | 4481 | Ethnicity | univariable |
| **United States** | | | | | | | |
| Carter et al (2017) | women with severe pre-eclampsia | single-center retrospective cohort study | 2004–08 | maternal: placental abruption  fetal:  NICU admission  neonatal death | 902 | SGA | univariable |
| Chaiworapongsa et al (2023) | women with pre-eclampsia | single-center case-control (cohort 1) and case-series (cohort 2) study | 2006–10; NA | maternal composite and single:  eclampsia blindness stroke myocardial ischemia pulmonary oedema elevated liver enzymes (ALT or AST >/= 70 IU/L) hepatic hematoma thrombocytopenia (platelet count <100x10^9/L) acute kidney injury (serum creatinine >1.1 mg/dL) placental abruption DIC maternal death  fetal:  requirement of oxygen supplementation continuous positive airway pressure (CPAP) or mechanical ventilation RDS confirmed sepsis NEC IVH periventricular leukomalacia meconium aspiration syndrome chronic lung disease neonatal death SGA | 603 | abnormal angiogenic profile (defined as a plasma ratio of the PlGF and sFlt-1 concentrations expressed as a multiple of the median (MoM) <10th percentile for gestational age)  analytic platform: Human VEGFR1/Flt-1 [Catalog #DVR100B] and Human PlGF Quantikine ELISA (R&D Systems) | univariable |
| Fishel Bartal et al (2022) | women with chronic hypertension | multi-center population-based retrospective cohort study | 2014–19 | maternal composite and single:  other hypertensive disorders (gestational hypertension and pre-eclampsia) primary cesarean delivery ICU admission maternal transfusion uterine rupture unplanned hysterectomy  fetal composite and single: preterm birth < 37 weeks SGA Apgar's score < 5 at 5 min assisted ventilation longer than 6 hours neonatal seizures neonatal death earlier and infant death | 389347 | Ethnicity | univariable |
| Heimberger et al (2020) | women with chronic hypertension and/or super-imposed pre-eclampsia | single-center retrospective analysis of prospective observational study | 2015–17 | maternal composite and single:  HELLP AST > 74U/L or ALT > 70U/L platelet count (< 100,000/μL) renal insufficiency (serum creatinine > 1.1mg/dL or a doubling of the baseline creatinine) transfusion of more than four units packed red blood cells  DIC placental abruption  eclampsia cerebral haemorrhage  cardiomyopathy pulmonary oedema  maternal death  fetal:  intrauterine fetal demise or perinatal death NICU admission birth-weight < 10th percentile | 115 | sFlt-1/PlGF ratio  analytic platform: BRAHMS KRYPTOR Compact plus system (Thermo Scientific) | univariable |
| Malhamé et al (2022_2) | women with pre-eclampsia, severe pre-eclampsia, superimposed pre-eclampsia, eclampsia or HELLP syndrome | single-center retrospective cohort study | 2007–17 | maternal composite:  pulmonary oedema acute heart failure acute myocardial infarction aneurysm cardiac arrest/ventricular fibrillation heart failure/arrest during surgery or procedure puerperal cerebrovascular disorders  cardiogenic shock conversion of cardiac rhythm  difficult-to-control severe hypertension (SBP/DBP ≥ 160/110 mmHg) | 4171 | multivariable model:  maternal age maternal education  previous live births previous cesarean delivery multiple gestation  chronic hypertension gestational diabetes gestational age at delivery (weeks) | multivariable |
| Malhamé et al (2022_1) | women with pre-eclampsia and/or eclampsia | multi-center population-based study | 2010–14 | maternal:  ICU admission acute renal failure pulmonary oedema pulmonary embolism congestive heart failure cardiomyopathy stroke death  maternal composite cardiovascular: congestive heart failure cardiomyopathy pulmonary oedema stroke  fetal:  preterm birth | 71159 | obstructive sleep apnea | univariable |
| Nisly et al (2023) | women with severe pre-eclampsia | single-center retrospective cohort study | 2013–19 | maternal composite:  maternal death  sepsis ICU admission  acute kidney injury (creatinine > 1.2 mg/dL)  postpartum dilation and curettage postpartum hysterectomy  venous thromboembolism  PPH  postpartum wound complication (within 30 days of delivery)  postpartum endometriosis pelvic abscess postpartum pneumonia readmission blood transfusion | 260 | maternal characteristics: advanced maternal age Ethnicity  White  Black  Asian  Other  Hispanic/Latin  Multiple gestation  multiparous private insurance  chronic hypertension  preexisting diabetes tobacco use in pregnancy marijuana use in pregnancy depression  prior cesarean delivery admission BMI (> 39.9) preeclampsia < 28 weeks GA preexisting IUGR on admission  Latency ≥ 7 days vaginal delivery candidate who attempted labor  delivery before 28 weeks GA  anemia at delivery (hematocrit < 25 %)  cesarean at delivery chorioamnionitis | univariable |
| Suresh et al (2022) | women with pre-eclampsia | secondary analysis of a single-center prospective cohort study | 2009–12 | maternal composite:  HELLP  eclampsia cerebral haemorrhage pulmonary oedema acute kidney injury DIC placental abruption death  fetal composite:  preterm birth < 37 weeks low birth weight (less than the 10th percentile) fetal or neonatal death | 1043 | sFlt-1/PlGF ratio  analytic platform: unknown | univariable |
| Thadhani et al (2022) | women with HDP | multi-center prospective cohort study | 2019–21 | maternal composite and single: severe hypertension (≥ 160 mmHg or ≥110 mmHg)  placental abruption cerebral haemorrhage seizure  pulmonary oedema DIC  AST or ALT levels > 80 U/l renal insufficiency (serum creatinine > 1.4 mg/dl) platelet count <100,000/µl   fetal:  SGA  fetal/neonatal death | 556 | sFlt-1/PlGF ratio  analytic platform: BRAHMS PlGF and sFlt-1 KRYPTOR (Thermo Scientific) | univariable |
| **Vietnam** | | | | | | | |
| Le et al (2018) | women with pre-eclampsia | single-center prospective cross-sectional study | 2015–17 | fetal composite and single:  IUGR preterm delivery Apgar score < 7 in 1 min fetal death/stillbirth neonatal death | 205 | serum uric acid concentration | univariable |
| **Abbreviations**: AC, Abdominal circumference; ACR, albumin creatinine ratio; ADMA, asymmetric dimethyl arginine; ALT, alanine transaminase; ART, assisted reproductive technology; AST, aspartate transaminase; BCW, British Columbia Women; BMI, body mass index; BP, blood pressure; CHIPS, Control of Hypertension In Pregnancy Study; CKD, chronic kidney disease; CNS, central nervous system; CPR, cerebroplacental ratio; CTG, cardiotocograph; DBP, diastolic blood pressure; DIC, disseminated intravascular coagulation; Dutch PETRA, Preeclampsia Eclampsia Trial Amsterdam; DV, ductus venosus; EFW, estimated fetal weight; FGR, fetal growth restriction; FINNPEC, Finnish Genetics of Preeclampsia Consortium; FiO2, Fraction of inspired oxygen; GA, gestational age; GFR, glomerular filtration rate; GlyFn, glycosylated fibronectin; HBsAg, Hepatitis B surface antigen; HDP, Hypertensive disorder of pregnancy; HELLP, hemolysis, elevated liver enzymes and low platelet count; HIE, hypoxic-ischemic encephalopathy; hs-CRP, high-sensitive C-reactive protein; HTN, hypertension; ICU, intensive care unit; IDA, iron deficiency anemia; IUGR, intrauterine growth restriction; IVH, intraventricular haemorrhage; MAP, mean arterial pressure; MCA, middle cerebral artery; MDA, malondialdehyde; miRNA, micro ribonucleic acid; NEC, necrotizing enterocolitis; NICU, neonatal intensive care unit; NST, non-stress test; OA, ophthalmic artery; OSA, obstructive sleep apnea; PDA, patent ductus arteriosus; PE, pre-eclampsia; PELICAN, Pre-EcLampsIa: Clinical ApplicatioN of PlGF study; PETRA, Pre-Eclampsia Triage by Rapid Assay; PI, pulsatility index; PIERS, Pre-eclampsia Integrated Estimate of RiSk; PlGF, placental growth factor; PNI, prognostic nutritional index; PNV, prenatal vitamin; PPH, postpartum haemorrhage; PR, peak ratio; PREP, Prediction of Complications in Early-Onset Preeclampsia; PRES, eclampsia or posterior reversible encephalopathy syndrome; PSV, peak systolic velocity; PVIV, peak velocity index; RDS, respiratory distress syndrome; REDF, reversed end diastolic flow; RI, resistance index; RIND, reversible ischemic neurological deficit; S/D, systolic/diastolic ratio; SBP, systolic blood pressure; sFlt-1, soluble fms-like tyrosine kinase-1; SGA, small for gestation age; SLE, systemic lupus erythematosus; SpO2, oxygen saturation; TAS, total antioxidant status; TOH, time of onset of hypertension; UA, umbilical artery; UPCR, urinary protein to creatinine ratio; UtA, uterine artery.  Characteristics of studies retrieved from Ukah et al, 2018 can be found in Supplemental table S4 of the corresponding publication | | | | | | | |

**Supplementary table 6: Studies excluded and awaiting classification**

| **Wrong patient population** | Agrawal et al, 2016^1^  Al Khalaf et al, 2021^2^  Al Khalaf et al, 2023^3^  Álvarez-Fernández et al, 2016^4^  Ashwal et al, 2022^5^  Balyan et al 2023^6^  Bian et al, 2019^7^  Boutin et al, 2021^8^  Duhig et al, 2019^9^  Elasy et al, 2022^10^  Escouto et al, 2018^11^  Gaccioli et al, 2018^12^  Haritha et al, 2022^13^  Herraiz et al, 2018^14^  Hopkins et al, 2021^15^  Hughes et al, 2022^16^  Hughes et al, 2023^17^  Jha et al, 2021^18^  Lin et al, 2022^19^  Lin et al, 2023^20^  Melo et al, 2023^21^  Nan et al, 2023^22^  Naz et al, 2021^23^  Neuman et al, 2020_1^24^  Neuman et al, 2020_2^25^  Nila et al, 2021^26^  Ohkuchi et al, 2021^27^  Oztas et al, 2016_2^28^  Parchem et al, 2020^29^  Rana et al, 2018^30^  Rani et al, 2016^31^  Saleh et al, 2016^32^  Saleh et al, 2017^33^  Saleh et al, 2018^34^  Sharp et al, 2018^35^  Singh et al, 2021^36^  Suresh et al,2023^37^  Teefey et al, 2019^38^  Zhang et al, 2021^39^ |
| --- | --- |
| **Wrong outcomes** | Ajmi et al, 2018^40^  Awan et al, 2022^41^  Barton et al, 2020^42^  Bromfield et al 2023^43^  Chelli et al, 2016^44^  Duhig et al, 2020^45^  Duhig et al, 2021_1^46^  Duhig et al, 2021_2^47^  Gao et al, 2021^48^  Gencheva et al, 2021^49^  Gottipati et al, 2019^50^  Hadley et al, 2017^51^  Hoffman et al, 2021^52^  Hopkins et al, 2019^53^  Hoppu et al, 2022^54^  Joslyn et al, 2022^55^  Kumar et al, 2021^56^  Liu et al, 2020^57^  Liu et al, 2023^58^  Lou et al, 2019^59^  Muñoz-Hernández et al, 2017^60^  Nguefack et al, 2018^61^  Oliveira et al, 2016^62^  Ozkan et al, 2023^63^  Oztas et al, 2016_1^64^  Perry et al, 2020^65^  Stitterich et al, 2021^66^  Yan et al, 2023^67^  Ye et al, 2021^68^  Zeisler et al, 2016^69^  Zhao et al, 2021^70^  Zhang et al, 2023^71^  Zhang et al, 2023^72^  Zheng et al, 2022^73^  Zhou et al, 2020^74^  Zhou et al, 2022^75^ |
| **Not presenting sufficient data for calculation of predictive performance measures** | Adami et al, 2020^76^  Baltajian et al, 2016^77^  Binder et al, 2022^78^  Chang et al, 2017^79^  Chaudary et al, 2023^80^  De Guzman Cielo et al, 2021^81^  Deng et al, 2022^82^  Gadhavi et al, 2023^83^  Gaspar et al, 2021^84^  Gyamfi-Bannerman et al, 2020^85^  Hoffmann et al, 2017^86^  Jayasingh et al, 2022^87^  Jeon et al, 2021^88^  Kalapahar et al, 2022^89^  Leanos-Miranda et al, 2017^90^  Li et al, 2021^91^  Li et al, 2018^92^  Liao et al, 2018^93^  Magee et al, 2020^94^  Mahesh et al, 2020^95^  Molina Pérez et al, 2021^96^  Naruse et al, 2021^97^  Nathan et al, 2018_1^98^  Nathan et al, 2018_2^99^  Ngwenya et al, 2020^100^  Ngwenya et al, 2021^101^  Srivastava et al, 2017^102^  Tanacan et al, 2019^103^  Umezuluike et al, 2021^104^  Willy et al, 2022^105^  Yu et al, 2022^106^  Zhang et al, 2022^107^ |
| **Prediction of maternal and neonatal outcomes cannot be separated** | Abbas et al, 2018^108^  Almeida et al, 2017^109^  Ben Gharbia et al 2022^110^  Bhorat et al, 2018^111^  Chen et al, 2020^112^  Droge et al, 2021_1^113^  Droge et al, 2021_2^114^  He et al, 2022^115^  Lau et al 2023^116^  Lv et al, 2022^117^  Ozdemir et al, 2020^118^  Peguero et al, 2021^119^  Salahuddin et al, 2016^120^  Saleh et al, 2021^121^  Samal et al 2023^122^  Schmidt et al, 2022^123^  Sroka et al, 2023^124^  Sun et al, 2021^125^  Sun et al, 2023^126^  Supakulwanit et al, 2019^127^  Wang et al, 2024^128^  Wen et al,^129^  Zhang et al 2023,^130^  Zhou et al, 2022^131^ |
| **Abstract of studies in full-text screening** | Hadley et al, 2016^132^  Ajmi et al, 2017^133^  Allotey et al, 2016^134^  Allotey et al, 2018^135^  Amorim et al, 2016^136^  Bian et al, 2018^137^  Duhig et al, 2016^138^  Mirkovic et al, 2019^139^  Nathan et al, 2017^140^  Pecoraro et al, 2019^141^  Rekik et al, 2021^142^  Robb et al, 2016^143^  Saleh et al, 2017^144^  Schmidt et al, 2021^145^  Sitterich et al, 2019^146^  Suresh et al, 2023^147^  Szklanna et al, 2021_2^148^  Tan et al, 2020^149^  Thangaratinam et al, 2016^150^  Ukah et al, 2017^151^ |
| **Review** | Ashworth et al, 2020^152^  Bellos et al, 2020^153^  Hurrell et al, 2020^154^  Josephine et al, 2021^155^  Lim et al, 2021^156^  Pecoraro et al, 2020^157^  Stepan et al, 2020^158^  Ukah et al, 2017^159^  Verlohren et al, 2022^160^ |
| **Wrong exposure** | Barton et al, 2020^161^  Mantel et al, 2023^162^  Mohamed Shaker El-Sayed Azzaz et al, 2016^163^  Villalain et al, 2022^164^  Wang et al, 2022^165^  Woelkers et al, 2016^166^ |
| **Wrong study design** | Avorgbedor et al, 2022^167^  Avalos et al, 2023^168^  Lim et al, 2020^169^  Magee et al, 2016^170^  Tardif et al, 20181^71^  Yang et al, 2022^172^ |
| **Wrong setting** | Elizalde-Valdés et al, 2016^173^  Li et al, 2023^174^ |
| **Previously excluded in last search** | Cheung et al, 2016^175^ |
| **Awaiting classification** | |
| **Preliminary data** | Allotey et al, 2019^176^  Cosban et al, 2023^177^  Dillon et al, 2022^178^  De Oliveira et al, 2023^179^  Di Martino, 2023^180^  Edvinsson et al, 2023^181^  Emeruwa et al, 2022^182^  Escouto et al, 2016^183^  Groenningsaeter et al, 2016^184^  Habib et al, 2020^185^  Herraiz et al, 2019^186^  Kawakita et al, 2023^187^  Ko et al, 2020^188^  Kubo et al, 2019^189^  Kuper et al, 2016^190^  Montenegro-Martínez et al, 2023^191^  Morton et al, 2019^192^  Myers et al, 2018^193^  Nadeau et al, 2016^194^  Nagabhushana et al, 2021^195^  Nan et al, 2021^196^  Rieger et al, 2021^197^  Sanusi et al, 2023^198^  Szklanna et al, 2021_1^199^  Tan et al, 2023^200^  Wang et al, 2020^201^  Winger et al, 2017^202^  Zahid et al, 2023^203^ |
| **Full-text not available** | Birukov et al, 2019^204^  Okwor et al, 2020^205^  Sheen et al, 2019^206^ |

**Supplementary table 7: Predictive performance of all prediction tests for single maternal outcomes.**

| **Study** | **Predictor** | **Outcome** | **Sample size** | **Event rate, n (%)** | **Sensitivity** | **Specificity** | **PPV** | **NPV** | **LR+** | **LR-** | **AUROC** |
| --- | --- | --- | --- | --- | --- | --- | --- | --- | --- | --- | --- |
| **Maternal death** | | | | | | | | | | | |
| Chadha et al (2022), India | UPCR ≥ 0.3 | maternal death | 141 | 4 (2.80) | 1.00 (0.51-1.00) | 0.11 (0.07-0.17) | 0.03 (0.01-0.08) | 1.00 (0.80-1.00) | 1.01 (0.75-1.37)* | 0.89 (0.06-12.87)* | 0.55 (0.53-0.58) |
| Jampana et al (2022), India | maternal serum uric acid level > 5.5 mg/dL | maternal death | 86 | 4 (4.65) | 0.75 (0.30-0.95) | 0.41 (0.31-0.52) | 0.06 (0.02-0.16) | 0.97 (0.85-0.99) | 1.28 (0.71-2.32) | 0.60 (0.11-3.36) | 0.58 (0.33-0.83) |
| Lei et al (2021), China | 24 h Proteinuria excretion < 0.3 g/24h | maternal death | 275 | 1 (0.40) | 0.00 (0.00-0.79) | 0.81 (0.76-0.85) | 0.00 (0.00-0.07) | 1.00 (0.97-1.00) | 1.29 (0.12-14.34)* | 0.93 (0.42-2.08)* | NA |
| Lei et al (2021), China | 24 h Proteinuria excretion ≥ 0.3 g/24h | maternal death | 275 | 1 (0.40) | 0.00 (0.00-0.79) | 0.57 (0.52-0.63) | 0.00 (0.00-0.03) | 0.99 (0.97-1.00) | 0.59 (0.05-6.50)* | 1.31 (0.58-2.93)* | NA |
| Lei et al (2021), China | 24 h Proteinuria excretion ≥ 2.0 g/24h | maternal death | 275 | 1 (0.40) | 0.00 (0.00-0.79) | 0.81 (0.76-0.85) | 0.00 (0.00-0.07) | 1.00 (0.97-1.00) | 1.29 (0.12-14.34)* | 0.93 (0.42-2.08)* | NA |
| Li et al (2018), China | 24 h Proteinuria excretion ≥ 0.3 g/24h | maternal death | 1,738 | 2 (0.12) | 0.50 (0.09-0.91) | 0.19 (0.17-0.21) | 0.00 (0.00-0.00) | 1.00 (0.98-1.00) | 0.62 (0.15-2.46) | 2.65 (0.66-10.65) | 0.34 (0.00-0.83) |
| Li et al (2018), China | 24 h Proteinuria excretion ≥ 2.0 g/24h | maternal death | 1,738 | 2 (0.12) | 0.50 (0.09-0.91) | 0.56 (0.53-0.58) | 0.00 (0.00-0.01) | 1.00 (0.99-1.00) | 1.13 (0.28-4.51) | 0.90 (0.22-3.60) | 0.53 (0.04-1.00) |
| Li et al (2018), China | 24 h Proteinuria excretion ≥ 5.0 g/24h | maternal death | 1,738 | 2 (0.12) | 0.50 (0.09-0.91) | 0.76 (0.74-0.78) | 0.00 (0.00-0.01) | 1.00 (1.00-1.00) | 2.08 (0.52-8.32) | 0.66 (0.16-2.63) | 0.63 (0.14-1.00) |
| Malhamé et al (2022_1), US | obstructive sleep apnea | maternal death (occurring during delivery hospitalization) | 71,159 | 13 (0.02) | 0.00 (0.00-0.23) | 1.00 (1.00-1.00) | 0.00 (0.00-0.01) | 1.00 (1.00-1.00) | 9.39 (0.62-143.23)* | 0.97 (0.88-1.07)* | 0.50 (0.50-0.50) |
| Yucesoy et al (2005), Turkey | platelets < 50000/mm^3^ | maternal death | 44 | 4 (9.10) | 0.25 (0.013-0.78) | 0.62 (0.013-0.77) | NA | NA | 0.70 (0.10-3.80) | 1.20 (0.60-2.2) | NA |
| **Admission to ICU** | | | | | | | | | | | |
| Binder et al (2021), Austria | angiogenic imbalance (sFlt-1/PlGF ratio above the 97th percentile and/or PlGF levels below 2.5th centile) without proteinuria | ICU admission (at any time) | 145 | 4 (2.80) | 0.75 (0.30-0.95) | 0.74 (0.66-0.80) | 0.07 (0.03-0.20) | 0.99 (0.95-1.00) | 2.86 (1.52-5.37) | 0.34 (0.06-1.86) | 0.74 (0.50-0.99) |
| Binder et al (2021), Austria | angiogenic imbalance (sFlt-1/PlGF ratio above the 97th percentile and/or PlGF levels below 2.5th centile) without IUGR | ICU admission (at any time) | 145 | 4 (2.80) | 1.00 (0.51-1.00) | 0.77 (0.70-0.83) | 0.11 (0.04-0.25) | 1.00 (0.97-1.00) | 3.93 (2.58-5.99)* | 0.13 (0.01-1.80)* | 0.89 (0.85-0.92) |
| Binder et al (2021), Austria | angiogenic imbalance (sFlt-1/PlGF ratio above the 97th percentile and/or PlGF levels below 2.5th centile) and/or proteinuria | ICU admission (at any time) | 145 | 4 (2.80) | 1.00 (0.51-1.00) | 0.62 (0.54-0.70) | 0.07 (0.03-0.17) | 1.00 (0.96-1.00) | 2.39 (1.67-3.43)* | 0.16 (0.01-2.23)* | 0.81 (0.77-0.85) |
| Binder et al (2021), Austria | proteinuria, other symptoms or IUGR without angiogenic imbalance | ICU admission (at any time) | 145 | 4 (2.80) | 0.00 (0.00-0.49) | 0.91 (0.86-0.95) | 0.00 (0.00-0.24) | 0.97 (0.93-0.99) | 1.14 (0.08-16.61)* | 0.99 (0.73-1.33)* | 0.46 (0.43-0.48) |
| Chaves et al (2017), Brazil | abnormal PR (ratio of the flow velocity of the second peak to that of the initial systolic velocity peak) ≥ 0.78 | ICU admission | 56 | 1 (1.79) | 1.00 (0.21-1.00) | 0.13 (0.06-0.24) | 0.02 (0.00-0.11) | 1.00 (0.65-1.00) | 0.87 (0.39-1.94)* | 1.87 (0.15-22.54)* | NA |
| Chaves et al (2017), Brazil | abnormal PR (ratio of the flow velocity of the second peak to that of the initial systolic velocity peak) ≥ 0.99 | ICU admission | 56 | 1 (1.79) | 0.00 (0.00-0.79) | 0.71 (0.58-0.81) | 0.00 (0.00-0.19) | 0.97 (0.87-1.00) | 0.85 (0.07-9.68)* | 1.06 (0.47-2.41)* | NA |
| Fishel Bartal et al (2022), US | Ethnicity: Non-Hispanic White | ICU admission | 389,054 | 2278 (0.59) | 0.36 (0.34-0.38) | 0.50 (0.50-0.50) | 0.00 (0.00-0.00) | 0.99 (0.99-0.99) | 0.71 (0.67-0.75) | 1.29 (1.25-1.33) | 0.43 (0.42-0.44) |
| Fishel Bartal et al (2022), US | Ethnicity: Non-Hispanic Black | ICU admission | 389,054 | 2278 (0.59) | 0.42 (0.40-0.44) | 0.70 (0.70-0.71) | 0.01 (0.01-0.01) | 1.00 (0.99-1.00) | 1.43 (1.36-1.50) | 0.82 (0.79-0.85) | 0.56 (0.55-0.57) |
| Fishel Bartal et al (2022), US | Ethnicity: Hispanic | ICU admission | 389,054 | 2278 (0.59) | 0.16 (0.14-0.17) | 0.85 (0.84-0.85) | 0.01 (0.01-0.01) | 0.99 (0.99-0.99) | 1.02 (0.93-1.12) | 1.00 (0.98-1.01) | 0.50 (0.49-0.51) |
| Fishel Bartal et al (2022), US | Ethnicity: Non-Hispanic Asian American, Native Hawaiian and other Pacific Islander | ICU admission | 389,054 | 2278 (0.59) | 0.05 (0.04-0.06) | 0.96 (0.96-0.97) | 0.01 (0.01-0.01) | 0.99 (0.99-0.99) | 1.31 (1.09-1.58) | 0.99 (0.98-1.00) | 0.51 (0.50-0.51) |
| Fishel Bartal et al (2022), US | Ethnicity: Non-Hispanic American Indian and Alaska Native | ICU admission | 389,054 | 2278 (0.59) | 0.02 (0.01-0.02) | 0.99 (0.99-0.99) | 0.01 (0.01-0.01) | 0.99 (0.99-0.99) | 1.50 (1.09-2.07) | 0.99 (0.99-1.00) | 0.50 (0.50-0.51) |
| Heimberger et al (2020), US | sFlt-1/PlGF ratio ≥ 85 | ICU admission | 115 | 1 (0.87) | 0.00 (0.00-0.79) | 0.68 (0.58-0.75) | 0.00 (0.00-0.09) | 0.99 (0.93-1.00) | 0.77 (0.07-8.58)* | 1.11 (0.49-2.50)* | NA |
| Malhamé et al (2022_1), US | obstructive sleep apnea | ICU admission (occurring during delivery hospitalization) | 71,159 | 7737 (10.87) | 0.01 (0.01-0.01) | 1.00 (1.00-1.00) | 0.24 (0.19-0.30) | 0.89 (0.89-0.89) | 2.60 (1.97-3.43) | 0.99 (0.99-1.00) | 0.50 (0.50-0.50) |
| Morikawa et al (2021), Japan | gestational weight gain during the week prior delivery ≥ 1.6 kg | ICU admission | 94 | 3 (3.20) | 1.00 (0.44-1.00) | 0.52 (0.42-0.62) | 0.06 (0.02-0.17) | 1.00 (0.92-1.00) | 1.81 (1.18-2.77)* | 0.24 (0.02-3.26)* | 0.76 (0.71-0.81) |
| Stolz et al (2018), Austria | sFlt-1/PlGF ratio ≥ 655 | ICU admission | 60 | 14 (23.30) | 0.43 (0.21-0.67) | 0.50 (0.36-0.64) | 0.21 (0.10-0.40) | 0.73 (0.56-0.86) | 0.86 (0.44-1.68) | 1.14 (0.67-1.96) | 0.46 (0.31-0.62) |
| Wei et al (2022), China | PNI ≤ 37 | ICU admission | 733 | 33 (4.50) | 0.70 (0.53-0.83) | 0.60 (0.56-0.63) | 0.08 (0.05-0.11) | 0.98 (0.96-0.99) | 1.72 (1.35-2.20) | 0.51 (0.30-0.86) | 0.65 (0.56-0.73) |
| Chadha et al (2022), India | UPCR ≥ 0.3 | HDU admission | 141 | 40 (28.40) | 0.97 (0.87-1.00) | 0.14 (0.08-0.22) | 0.31 (0.24-0.39) | 0.93 (0.70-0.99) | 1.13 (1.03-1.24) | 0.18 (0.02-1.33) | 0.56 (0.52-0.60) |
| Webster et al (2018), UK | Ethnicity: White | HDU admission (after delivery) | 4,481 | 417 (9.35) | 0.36 (0.32-0.41) | 0.52 (0.50-0.53) | 0.07 (0.06-0.08) | 0.89 (0.87-0.90) | 0.75 (0.65-0.85) | 1.24 (1.15-1.34) | 0.44 (0.41-0.46) |
| Webster et al (2018), UK | Ethnicity: Black | HDU admission (after delivery) | 4,481 | 417 (9.35) | 0.48 (0.43-0.53) | 0.66 (0.64-0.67) | 0.13 (0.11-0.14) | 0.92 (0.91-0.93) | 1.40 (1.26-1.56) | 0.79 (0.72-0.87) | 0.57 (0.54-0.59) |
| Webster et al (2018), UK | Ethnicity: Asian | HDU admission (after delivery) | 4,481 | 417 (9.35) | 0.06 (0.04-0.09) | 0.91 (0.90-0.92) | 0.07 (0.05-0.10) | 0.90 (0.90-0.91) | 0.72 (0.49-1.06) | 1.03 (1.00-1.05) | 0.49 (0.48-0.50) |
| Leanos-Miranda et al (2020), Mexico | sFlt-1/PlGF ratio ≥ 85 | Intubation (other than solely for caesarean section) | 810 | 6 (0.74) | 1.00 (0.61-1.00) | 0.48 (0.45-0.51) | 0.01 (0.01-0.03) | 1.00 (0.99-1.00) | 1.79 (1.44-2.22)* | 0.15 (0.01-2.15)* | 0.74 (0.72-0.76) |
| Leanos-Miranda et al (2020), Mexico | sFlt-1/PlGF ratio ≥ 38 | Intubation (other than solely for caesarean section) | 810 | 6 (0.74) | 1.00 (0.61-1.00) | 0.35 (0.32-0.39) | 0.01 (0.01-0.02) | 1.00 (0.99-1.00) | 1.44 (1.16-1.77)* | 0.20 (0.01-2.93)* | 0.68 (0.66-0.69) |
| **Eclampsia** | | | | | | | | | | | |
| Chadha et al (2022), India | UPCR ≥ 0.3 | eclampsia | 141 | 7 (5.00) | 1.00 (0.65-1.00) | 0.11 (0.07-0.18) | 0.06 (0.03-0.11) | 1.00 (0.80-1.00) | 1.06 (0.88-1.28)* | 0.54 (0.04-8.30)* | 0.56 (0.53-0.58) |
| Chaiworapongsa et al (2023), US | abnormal angiogenic profile (sFlt-1/PlGF ratio < 10th percentile for gestational age) in early-onset pre-eclampsia (cohort case-series) | eclampsia | 89 | 1 (1.12) | 1.00 (0.21-1.00) | 0.02 (0.01-0.08) | 0.01 (0.00-0.06) | 1.00 (0.34-1.00) | 0.77 (0.35-1.72)* | 8.90 (0.60-131.59)* | NA |
| Chaiworapongsa et al (2023), US | abnormal angiogenic profile (sFlt-1/PlGF ratio < 10th percentile for gestational age) in intermediate pre-eclampsia (cohort case-series) | eclampsia | 105 | 1 (0.95) | 1.00 (0.21-1.00) | 0.20 (0.14-0.29) | 0.01 (0.00-0.06) | 1.00 (0.85-1.00) | 0.94 (0.42-2.11)* | 1.22 (0.11-13.87)* | NA |
| Chaiworapongsa et al (2023), US | abnormal angiogenic profile (sFlt-1/PlGF ratio < 10th percentile for gestational age) in term pre-eclampsia (cohort case-series) | eclampsia | 258 | 2 (0.78) | 1.00 (0.34-1.00) | 0.46 (0.40-0.52) | 0.01 (0.00-0.05) | 1.00 (0.97-1.00) | 1.54 (0.91-2.58)* | 0.36 (0.03-4.59)* | 0.73 (0.70-0.76) |
| Hall et al (2002), South Africa | 24 h urine increased by ≥2g | eclampsia | 74 | 1 (1.40) | 1.00 (0.06-1) | 0.62 (0.50-0.73) | NA | NA | 2.60  (1.90- 3.50) | NA | NA |
| Jampana et al (2022), India | maternal serum uric acid level > 5.5 mg/dL | eclampsia | 86 | 12 (13.95) | 0.83 (0.55-0.95) | 0.45 (0.34-0.56) | 0.20 (0.11-0.32) | 0.94 (0.81-0.98) | 1.50 (1.09-2.08) | 0.37 (0.10-1.36) | 0.64 (0.52-0.76) |
| Karge et al (2022), Germany | BMI ≥ 25 kg/m^2 | eclampsia | 141 | 1 (0.71) | 1.00 (0.21-1.00) | 0.60 (0.52-0.68) | 0.02 (0.00-0.09) | 1.00 (0.96-1.00) | 1.87 (0.82-4.27)* | 0.42 (0.04-4.62)* | NA |
| Karge et al (2022), Germany | BMI ≥ 30 kg/m^2 | eclampsia | 141 | 1 (0.71) | 0.00 (0.00-0.79) | 0.80 (0.73-0.86) | 0.00 (0.00-0.12) | 0.99 (0.95-1.00) | 1.24 (0.11-13.95)* | 0.94 (0.42-2.10)* | NA |
| Leanos-Miranda et al (2020), Mexico | sFlt-1/PlGF ratio ≥ 38 | eclampsia | 810 | 3 (0.37) | 1.00 (0.44-1.00) | 0.35 (0.32-0.39) | 0.01 (0.00-0.02) | 1.00 (0.99-1.00) | 1.35 (0.93-1.96)* | 0.36 (0.03-4.75)* | 0.68 (0.66-0.69) |
| Leanos-Miranda et al (2020), Mexico | sFlt-1/PlGF ratio ≥ 85 | eclampsia | 810 | 3 (0.73) | 1.00 (0.44-1.00) | 0.48 (0.44-0.51) | 0.01 (0.00-0.02) | 1.00 (0.99-1.00) | 1.68 (1.15-2.44)* | 0.26 (0.02-3.50)* | 0.74 (0.72-0.76) |
| Lei et al (2021), China | 24 h Proteinuria excretion < 0.3 g/24h | eclampsia | 275 | 1 (0.40) | 0.00 (0.00-0.79) | 0.81 (0.76-0.85) | 0.00 (0.00-0.07) | 1.00 (0.97-1.00) | 1.29 (0.12-14.34)* | 0.93 (0.42-2.08)* | NA |
| Lei et al (2021), China | 24 h Proteinuria excretion ≥ 0.3 g/24h | eclampsia | 275 | 1 (0.40) | 0.00 (0.00-0.79) | 0.57 (0.51-0.63) | 0.00 (0.00-0.03) | 0.99 (0.97-1.00) | 0.59 (0.05-6.48)* | 1.31 (0.58-2.93)* | NA |
| Lei et al (2021), China | 24 h Proteinuria excretion ≥ 2.0 g/24h | eclampsia | 275 | 1 (0.40) | 0.00 (0.00-0.79) | 0.81 (0.76-0.85) | 0.00 (0.00-0.07) | 1.00 (0.97-1.00) | 1.29 (0.12-14.34)* | 0.93 (0.42-2.08)* | NA |
| Li et al (2018), China | 24 h Proteinuria excretion ≥ 0.3 g/24h | eclampsia | 1,738 | 10 (0.58) | 1.00 (0.72-1.00) | 0.19 (0.17-0.21) | 0.01 (0.00-0.01) | 1.00 (0.99-1.00) | 1.18 (1.03-1.34)* | 0.24 (0.02-3.60)* | 0.59 (0.59-0.60) |
| Li et al (2018), China | 24 h Proteinuria excretion ≥ 2.0 g/24h | eclampsia | 1,738 | 10 (0.58) | 0.90 (0.60-0.98) | 0.56 (0.53-0.58) | 0.01 (0.01-0.02) | 1.00 (0.99-1.00) | 2.04 (1.65-2.52) | 0.18 (0.03-1.15) | 0.73 (0.63-0.83) |
| Li et al (2018), China | 24 h Proteinuria excretion ≥ 5.0 g/24h | eclampsia | 1,738 | 10 (0.58) | 0.70 (0.40-0.89) | 0.76 (0.74-0.78) | 0.02 (0.01-0.03) | 1.00 (0.99-1.00) | 2.94 (1.94-4.44) | 0.39 (0.15-1.02) | 0.73 (0.58-0.88) |
| Romero et al (1988), US | AST 2SD above mean | eclampsia | 275 | 7 (2.50) | 0.71 (0.30-0.95) | 0.80 (0.75-0.85) | NA | NA | 3.60 (2.10-6.10) | 0.40 (0.10-1.20) | NA |
| Sudjai et al (2022), Thailand | maternal serum uric acid level < 5.0 mg/dL | eclampsia | 400 | 5 (1.25) | 0.00 (0.00-0.43) | 0.76 (0.71-0.80) | 0.00 (0.00-0.04) | 0.98 (0.96-0.99) | 0.34 (0.02-4.89)* | 1.21 (0.95-1.55)* | 0.38 (0.36-0.40) |
| Sudjai et al (2022), Thailand | maternal serum uric acid level ≥ 5.0 mg/dL | eclampsia | 400 | 5 (1.25) | 1.00 (0.57-1.00) | 0.24 (0.20-0.29) | 0.02 (0.01-0.04) | 1.00 (0.96-1.00) | 1.21 (0.95-1.55)* | 0.34 (0.02-4.89)* | 0.62 (0.60-0.64) |
| Sudjai et al (2022), Thailand | maternal serum uric acid level ≥ 7.0 mg/dL | eclampsia | 400 | 5 (1.25) | 0.60 (0.23-0.88) | 0.69 (0.65-0.74) | 0.02 (0.01-0.07) | 0.99 (0.97-1.00) | 1.96 (0.94-4.07) | 0.58 (0.20-1.69) | 0.65 (0.41-0.89) |
| Thadhani et al (2022), US | sFlt-1/PlGF ratio ≥ 40 | eclampsia (within 2 weeks of enrolment) | 556 | 2 (0.40) | 1.00 (0.34-1.00) | 0.52 (0.48-0.56) | 0.01 (0.00-0.03) | 1.00 (0.99-1.00) | 1.74 (1.04-2.91)* | 0.32 (0.03-4.02)* | 0.76 (0.74-0.78) |
| Witlin et al (1999), US | MAP > 105 mm Hg | eclampsia | 445 | 40 (9.00) | 0.93 (0.79-0.98) | 0.03 (0.02-0.06) | NA | NA | 1.00  (0.90- 1.90) | 2.30 (0.90-8.00) | NA |
| Ben Salem et al (2003), Tunisia | headache | eclampsia | 120 | 41 (34.20) | 0.98 (0.86-1.00) | 0.27 (0.18-0.38) | NA | NA | 1.30 (1.20-1.50) | 0.10 (0.00-0.70) | NA |
| Ben Salem et al (2003), Tunisia | visual symptoms | eclampsia | 120 | 41 (34.20) | 0.85 (0.70-0.94) | 0.66 (0.54-0.76) | NA | NA | 2.50 (1.80-3.50) | 0.20 (0.10-0.50) | NA |
| Ben Salem et al (2003), Tunisia | vivid deep tendon reflexes | eclampsia | 120 | 41 (34.20) | 0.98 (0.86-1.00) | 0.47 (0.36-0.58) | NA | NA | 1.80 (1.50-2.30) | 0.10 (0.00-0.40) | NA |
| Ben Salem et al (2003), Tunisia | SBP ≥ 160 mmHg | eclampsia | 120 | 41 (34.20) | 0.93 (0.79-0.98) | 0.24 (0.15-0.35) | NA | NA | 1.20 (1.00-1.40) | 0.30 (0.10-1.00) | NA |
| Ben Salem et al (2003), Tunisia | dipstick > 3 + | eclampsia | 120 | 41 (34.20) | 0.85 (0.70-0.94) | 0.53 (0.42-0.64) | NA | NA | 1.8 (1.4-2.4) | 0.3 (0.1-0.6) | NA |
| Ben Salem et al (2003), Tunisia | 24 h urine > 3g/d | eclampsia | 120 | 41 (34.20) | 0.37 (0.23-0.53) | 0.91 (0.82-0.96) | NA | NA | 4.10 (1.80-9.30) | 0.70 (0.60-0.90) | NA |
| Ben Salem et al (2003), Tunisia | creatinine > 100 µmol/L | eclampsia | 120 | 41 (34.20) | 0.39 (0.25-0.56) | 0.81 (0.70-0.89) | NA | NA | 2.10 (1.10-3.70) | 0.80 (0.60-1.0) | NA |
| Ben Salem et al (2003), Tunisia | uric acid ≥ 350 µmol/L | eclampsia | 120 | 41 (34.20) | 0.83 (0.67-0.92) | 0.66 (0.54-0.92) | NA | NA | 2.40 (1.70-3.40) | 0.30 (0.10-0.50) | NA |
| Ben Salem et al (2003), Tunisia | AST > 30 IU/L | eclampsia | 120 | 41 (34.20) | 0.63 (0.47-0.78) | 0.71 (0.59-0.80) | NA | NA | 2.20 (1.40-3.30) | 0.50 (0.40-0.80) | NA |
| Vasquez et al (2021), Argentina | epigastric pain | eclampsia (seizures in patient with preeclampsia) | 172 | 31 (18.02) | 0.17 (0.07-0.35) | 0.59 (0.51-0.67) | 0.08 (0.03-0.18) | 0.77 (0.68-0.84) | 0.40 (0.17-0.90) | 1.42 (1.15-1.74) | 0.38 (0.30-0.45) |
| Vasquez et al (2021), Argentina | nausea and vomiting | eclampsia (seizures in patient with preeclampsia) | 172 | 31 (18.02) | 0.09 (0.03-0.25) | 0.87 (0.81-0.92) | 0.14 (0.05-0.35) | 0.81 (0.74-0.87) | 0.74 (0.23-2.35) | 1.04 (0.91-1.19) | 0.48 (0.42-0.54) |
| Vasquez et al (2021), Argentina | hyperreflexia | eclampsia (seizures in patient with preeclampsia) | 172 | 31 (18.02) | 0.24 (0.12-0.42) | 0.65 (0.56-0.72) | 0.13 (0.06-0.24) | 0.80 (0.72-0.86) | 0.64 (0.32-1.28) | 1.19 (0.95-1.49) | 0.44 (0.35-0.52) |
| Vasquez et al (2021), Argentina | visual symptoms (photopsias or scotomas, blurred vision, photophobia, or blindness) | eclampsia (seizures in patient with preeclampsia) | 172 | 31 (18.02) | 0.55 (0.38-0.72) | 0.54 (0.46-0.63) | 0.20 (0.13-0.31) | 0.85 (0.76-0.91) | 1.19 (0.83-1.72) | 0.83 (0.55-1.26) | 0.54 (0.45-0.64) |
| Vasquez et al (2021), Argentina | headache | eclampsia (seizures in patient with preeclampsia) | 172 | 31 (18.02) | 0.77 (0.59-0.88) | 0.43 (0.35-0.52) | 0.23 (0.16-0.32) | 0.89 (0.80-0.95) | 1.35 (1.07-1.72) | 0.53 (0.27-1.04) | 0.60 (0.52-0.69) |
| Yucesoy et al (2005), Turkey | platelets < 50000/mm^3^ | eclampsia | 44 | 13 (29.50) | 0.39 (0.15-0.68) | 0.65 (0.45-0.80) | NA | NA | 1.10 (0.50-2.50) | 1.00 (0.60-1.50) | NA |
| **Neurological complications** | | | | | | | | | | | |
| Malhamé et al (2022_1), US | obstructive sleep apnea | stroke (occurring during delivery hospitalization) | 71,159 | 33 (0.05) | 0.03 (0.01-0.15) | 1.00 (1.00-1.00) | 0.00 (0.00-0.02) | 1.00 (1.00-1.00) | 8.01 (1.16-55.41) | 0.97 (0.92-1.03) | 0.51 (0.48-0.54) |
| Leanos-Miranda et al (2020), Mexico | sFlt-1/PlGF ratio ≥ 38 | cerebral haemorrhage | 810 | 1 (0.12) | 1.00 (0.21-1.00) | 0.35 (0.32-0.38) | 0.00 (0.00-0.01) | 1.00 (0.99-1.00) | 1.16 (0.52-2.58)* | 0.71 (0.06-7.86)* | NA |
| Leanos-Miranda et al (2020), Mexico | sFlt-1/PlGF ratio ≥ 85 | cerebral haemorrhage | 810 | 1 (0.12) | 1.00 (0.21-1.00) | 0.48 (0.44-0.51) | 0.00 (0.00-0.01) | 1.00 (0.99-1.00) | 1.43 (0.64-3.20)* | 0.52 (0.05-5.78)* | NA |
| Chaves et al (2017), Brazil | abnormal PR (ratio of the flow velocity of the second peak to that of the initial systolic velocity peak) ≥ 0.78 | CNS injury (eclampsia or PRES) | 56 | 3 (5.36) | 1.00 (0.44-1.00) | 0.13 (0.07-0.25) | 0.06 (0.02-0.17) | 1.00 (0.65-1.00) | 1.02 (0.69-1.49)* | 0.90 (0.06-13.08)* | 0.57 (0.52-0.61) |
| Chaves et al (2017), Brazil | abnormal PR (ratio of the flow velocity of the second peak to that of the initial systolic velocity peak) ≥ 0.99 | CNS injury (eclampsia or PRES) | 56 | 3 (5.36) | 0.33 (0.06-0.79) | 0.72 (0.58-0.82) | 0.06 (0.01-0.28) | 0.95 (0.83-0.99) | 1.18 (0.22-6.17) | 0.93 (0.41-2.11) | 0.53 (0.19-0.86) |
| **Retinal disease** | | | | | | | | | | | |
| Lei et al (2021), China | 24 h Proteinuria excretion < 0.3 g/24h | retinal disease | 275 | 19 (6.90) | 0.32 (0.15-0.54) | 0.36 (0.30-0.42) | 0.04 (0.02-0.07) | 0.88 (0.80-0.93) | 0.49 (0.25-0.96) | 1.90 (1.35-2.69) | 0.34 (0.23-0.45) |
| Lei et al (2021), China | 24 h Proteinuria excretion ≥ 0.3 g/24h | retinal disease | 275 | 19 (6.90) | 0.26 (0.12-0.49) | 0.56 (0.50-0.62) | 0.04 (0.02-0.10) | 0.91 (0.86-0.95) | 0.60 (0.28-1.29) | 1.31 (0.98-1.75) | 0.41 (0.31-0.52) |
| Lei et al (2021), China | 24 h Proteinuria excretion ≥ 2.0 g/24h | retinal disease | 275 | 19 (6.90) | 0.16 (0.06-0.38) | 0.80 (0.75-0.85) | 0.06 (0.02-0.15) | 0.93 (0.89-0.96) | 0.81 (0.28-2.35) | 1.05 (0.85-1.28) | 0.48 (0.39-0.57) |
| Morikawa et al (2020), Japan | serum total protein level at PE diagnosis ≤ 49 g/L | CSC (continuously during pregnancy) | 94 | 4 (4.30) | 0.75 (0.30-0.95) | 0.78 (0.68-0.85) | 0.13 (0.05-0.32) | 0.99 (0.92-1.00) | 3.38 (1.70-6.70) | 0.32 (0.06-1.76) | 0.76 (0.52-1.00) |
| Morikawa et al (2020), Japan | serum total protein level at delivery ≤ 42 g/L | CSC (continuously during pregnancy) | 94 | 4 (4.30) | 0.75 (0.30-0.95) | 0.99 (0.94-1.00) | 0.75 (0.30-0.95) | 0.99 (0.94-1.00) | 68.25 (8.97-519.47) | 0.25 (0.05-1.38) | 0.87 (0.62-1.00) |
| Morikawa et al (2021), Japan | gestational weight gain during the week prior delivery ≥ 1.6 kg | CSC | 94 | 4 (4.30) | 0.75 (0.30-0.95) | 0.51 (0.41-0.61) | 0.06 (0.02-0.17) | 0.98 (0.89-1.00) | 1.53 (0.84-2.81) | 0.49 (0.09-2.70) | 0.63 (0.38-0.88) |
| Morikawa et al (2021), Japan | gestational weight gain during the week prior delivery ≥ 2.2 kg | CSC | 94 | 4 (4.30) | 0.75 (0.30-0.95) | 0.68 (0.58-0.77) | 0.09 (0.03-0.24) | 0.98 (0.92-1.00) | 2.35 (1.24-4.47) | 0.37 (0.07-2.01) | 0.72 (0.47-0.97) |
| Wei et al (2022), China | PNI ≤ 37 | hypertensive retinopathy | 733 | 10 (1.36) | 0.60 (0.31-0.83) | 0.59 (0.55-0.62) | 0.02 (0.01-0.04) | 0.99 (0.98-1.00) | 1.45 (0.87-2.42) | 0.68 (0.32-1.46) | 0.59 (0.43-0.75) |
| Ye et al (2020), China | albumin < 30 g /L | retinopathy stage 3/4 (retinal oedema, haemorrhages, exudates and optical disc oedema) | 534 | 78 (14.61) | 0.55 (0.44-0.66) | 0.54 (0.49-0.58) | 0.17 (0.13-0.22) | 0.88 (0.83-0.91) | 1.20 (0.96-1.50) | 0.83 (0.64-1.08) | 0.55 (0.49-0.61) |
| Ye et al (2020), China | albumin ≥ 30 g /L | retinopathy stage 3/4 (retinal oedema, haemorrhages, exudates and optical disc oedema) | 534 | 78 (14.61) | 0.45 (0.34-0.56) | 0.46 (0.42-0.51) | 0.12 (0.09-0.17) | 0.83 (0.78-0.87) | 0.83 (0.64-1.08) | 1.20 (0.96-1.50) | 0.45 (0.39-0.51) |
| Ye et al (2020), China | white blood cell ≥ 10,000x10^9/L | retinopathy stage 3/4 (retinal oedema, haemorrhages, exudates and optical disc oedema) | 534 | 78 (14.61) | 0.49 (0.38-0.60) | 0.36 (0.32-0.41) | 0.12 (0.09-0.16) | 0.81 (0.75-0.85) | 0.77 (0.60-0.97) | 1.41 (1.10-1.81) | 0.43 (0.37-0.49) |
| Ye et al (2020), China | white blood cell > 10,000x10^9/L | retinopathy stage 3/4 (retinal oedema, haemorrhages, exudates and optical disc oedema) | 534 | 78 (14.61) | 0.51 (0.40-0.62) | 0.64 (0.59-0.68) | 0.19 (0.15-0.25) | 0.88 (0.84-0.91) | 1.41 (1.10-1.81) | 0.77 (0.60-0.97) | 0.57 (0.51-0.63) |
| Ye et al (2020), China | early onset pre-eclampsia | retinopathy stage 3/4 (retinal oedema, haemorrhages, exudates and optical disc oedema) | 534 | 78 (14.61) | 0.72 (0.61-0.81) | 0.44 (0.40-0.49) | 0.18 (0.14-0.23) | 0.90 (0.86-0.93) | 1.29 (1.10-1.51) | 0.64 (0.44-0.92) | 0.58 (0.53-0.64) |
| Ye et al (2020), China | late onset pre-eclampsia | retinopathy stage 3/4 (retinal oedema, haemorrhages, exudates and optical disc oedema) | 534 | 78 (14.61) | 0.28 (0.19-0.39) | 0.56 (0.51-0.60) | 0.10 (0.07-0.14) | 0.82 (0.77-0.86) | 0.64 (0.44-0.92) | 1.29 (1.10-1.51) | 0.42 (0.36-0.47) |
| Ye et al (2020), China | severe hypertension SBP/DBP ≥ 160/110 mmHg | retinopathy stage 3/4 (retinal oedema, haemorrhages, exudates and optical disc oedema) | 534 | 78 (14.61) | 0.83 (0.74-0.90) | 0.29 (0.25-0.34) | 0.17 (0.13-0.21) | 0.91 (0.85-0.95) | 1.18 (1.05-1.32) | 0.57 (0.34-0.95) | 0.56 (0.52-0.61) |
| Ye et al (2020), China | ALT (elevated) | retinopathy stage 3/4 (retinal oedema, haemorrhages, exudates and optical disc oedema) | 534 | 78 (14.61) | 0.24 (0.16-0.35) | 0.73 (0.69-0.77) | 0.13 (0.09-0.20) | 0.85 (0.81-0.88) | 0.90 (0.59-1.36) | 1.04 (0.91-1.19) | 0.49 (0.43-0.54) |
| Ye et al (2020), China | AST (elevated) | retinopathy stage 3/4 (retinal oedema, haemorrhages, exudates and optical disc oedema) | 534 | 78 (14.61) | 0.60 (0.49-0.70) | 0.50 (0.45-0.54) | 0.17 (0.13-0.22) | 0.88 (0.83-0.91) | 1.19 (0.98-1.46) | 0.80 (0.60-1.07) | 0.55 (0.49-0.61) |
| **Cardicac disease** | | | | | | | | | | | |
| Malhamé et al (2022_1), US | obstructive sleep apnea | congestive heart failure | 71,159 | 160 (0.22) | 0.01 (0.00-0.03) | 1.00 (1.00-1.00) | 0.00 (0.00-0.02) | 1.00 (1.00-1.00) | 1.65 (0.23-11.68) | 1.00 (0.99-1.01) | 0.50 (0.50-0.51) |
| Li et al (2018), China | 24 h Proteinuria excretion ≥ 0.3 g/24h | heart failure | 1,738 | 20 (1.15) | 0.95 (0.76-0.99) | 0.19 (0.17-0.21) | 0.01 (0.01-0.02) | 1.00 (0.98-1.00) | 1.17 (1.06-1.30) | 0.26 (0.04-1.78) | 0.57 (0.52-0.62) |
| Li et al (2018), China | 24 h Proteinuria excretion ≥ 2.0 g/24h | heart failure | 1,738 | 20 (1.15) | 0.75 (0.53-0.89) | 0.56 (0.54-0.58) | 0.02 (0.01-0.03) | 0.99 (0.99-1.00) | 1.70 (1.31-2.20) | 0.45 (0.21-0.96) | 0.65 (0.56-0.75) |
| Li et al (2018), China | 24 h Proteinuria excretion ≥ 5.0 g/24h | heart failure | 1,738 | 20 (1.15) | 0.35 (0.18-0.57) | 0.76 (0.74-0.78) | 0.02 (0.01-0.03) | 0.99 (0.98-0.99) | 1.46 (0.80-2.67) | 0.86 (0.62-1.18) | 0.56 (0.45-0.66) |
| Wei et al (2022), China | PNI ≤ 37 | heart failure (series of symptoms (dyspnea, open breathing, lower limb swelling) and signs (elevated jugular pressure, pulmonary congestion) | 733 | 32 (4.37) | 0.69 (0.51-0.82) | 0.59 (0.56-0.63) | 0.07 (0.05-0.11) | 0.98 (0.96-0.99) | 1.70 (1.32-2.18) | 0.53 (0.31-0.88) | 0.64 (0.56-0.72) |
| Morikawa et al (2021), Japan | gestational weight gain during the week prior delivery ≥ 1.6 kg | peripartum cardiomyopathy | 94 | 3 (3.20) | 1.00 (0.44-1.00) | 0.52 (0.42-0.62) | 0.06 (0.02-0.17) | 1.00 (0.92-1.00) | 1.81 (1.18-2.77)* | 0.24 (0.02-3.26)* | 0.76 (0.71-0.81) |
| Morikawa et al (2021), Japan | gestational weight gain during the week prior delivery ≥ 3.7 kg | peripartum cardiomyopathy | 94 | 3 (3.20) | 0.67 (0.21-0.94) | 0.86 (0.77-0.91) | 0.13 (0.04-0.38) | 0.99 (0.93-1.00) | 4.56 (1.77-11.74) | 0.39 (0.08-1.94) | 0.76 (0.43-1.00) |
| Morikawa et al (2020), Japan | serum total protein level at delivery ≤ 43 g/L | peripartum cardiomyopathy | 94 | 3 (3.20) | 0.67 (0.21-0.94) | 0.96 (0.90-0.98) | 0.33 (0.10-0.70) | 0.99 (0.94-1.00) | 15.67 (4.49-54.62) | 0.35 (0.07-1.73) | 0.81 (0.48-1.00) |
| Morikawa et al (2020), Japan | serum total protein level at PE diagnosis ≤ 45 g/L | peripartum cardiomyopathy | 94 | 3 (3.20) | 0.67 (0.21-0.94) | 0.94 (0.88-0.98) | 0.29 (0.08-0.64) | 0.99 (0.94-1.00) | 12.67 (3.93-40.80) | 0.35 (0.07-1.74) | 0.81 (0.48-1.00) |
| Malhamé et al (2022_1), US | obstructive sleep apnea (OSA) | cardiomyopathy | 71,159 | 167 (0.23) | 0.02 (0.01-0.05) | 1.00 (1.00-1.00) | 0.01 (0.00-0.03) | 1.00 (1.00-1.00) | 4.78 (1.55-14.75) | 0.99 (0.97-1.01) | 0.51 (0.50-0.52) |
| **Pulmonary oedema and respiratory failure** | | | | | | | | | | | |
| Binder et al (2021), Austria | angiogenic imbalance (sFlt-1/PlGF ratio above the 97th percentile and/or PlGF levels below 2.5th centile) without proteinuria | pulmonary oedema (at any time) | 145 | 1 (0.70) | 1.00 (0.21-1.00) | 0.73 (0.65-0.80) | 0.02 (0.00-0.13) | 1.00 (0.96-1.00) | 2.75 (1.18-6.40)* | 0.34 (0.03-3.80)* | NA |
| Binder et al (2021), Austria | angiogenic imbalance (sFlt-1/PlGF ratio above the 97th percentile and/or PlGF levels below 2.5th centile) without IUGR | pulmonary oedema (at any time) | 145 | 1 (0.70) | 1.00 (0.21-1.00) | 0.76 (0.68-0.82) | 0.03 (0.00-0.14) | 1.00 (0.97-1.00) | 3.06 (1.31-7.16)* | 0.33 (0.03-3.66)* | NA |
| Binder et al (2021), Austria | angiogenic imbalance (sFlt-1/PlGF ratio above the 97th percentile and/or PlGF levels below 2.5th centile) and/or proteinuria | pulmonary oedema (at any time) | 145 | 1 (0.70) | 1.00 (0.21-1.00) | 0.61 (0.53-0.69) | 0.02 (0.00-0.09) | 1.00 (0.96-1.00) | 1.92 (0.84-4.40)* | 0.41 (0.04-4.53)* | NA |
| Binder et al (2021), Austria | proteinuria, other symptoms or IUGR without angiogenic imbalance | pulmonary oedema (at any time) | 145 | 1 (0.70) | 0.00 (0.00-0.79) | 0.92 (0.86-0.95) | 0.00 (0.00-0.24) | 0.99 (0.96-1.00) | 2.90 (0.25-33.88)* | 0.82 (0.37-1.83)* | NA |
| Chaiworapongsa et al (2023), US | abnormal angiogenic profile (sFlt-1/PlGF ratio < 10th percentile for gestational age) in early-onset pre-eclampsia (cohort case-series) | pulmonary oedema | 89 | 4 (4.49) | 1.00 (0.51-1.00) | 0.02 (0.01-0.08) | 0.05 (0.02-0.11) | 1.00 (0.34-1.00) | 0.93 (0.69-1.24)* | 3.44 (0.19-62.48)* | 0.51 (0.50-0.53) |
| Chaiworapongsa et al (2023), US | abnormal angiogenic profile (sFlt-1/PlGF ratio < 10th percentile for gestational age) in intermediate pre-eclampsia (cohort case-series) | pulmonary oedema | 105 | 1 (0.95) | 1.00 (0.21-1.00) | 0.20 (0.14-0.29) | 0.01 (0.00-0.06) | 1.00 (0.85-1.00) | 0.94 (0.42-2.11)* | 1.22 (0.11-13.87)* | NA |
| Chaiworapongsa et al (2023), US | abnormal angiogenic profile (sFlt-1/PlGF ratio < 10th percentile for gestational age) in term pre-eclampsia (cohort case-series) | pulmonary oedema | 258 | 3 (1.16) | 0.67 (0.21-0.94) | 0.45 (0.39-0.52) | 0.01 (0.00-0.05) | 0.99 (0.95-1.00) | 1.22 (0.55-2.74) | 0.73 (0.15-3.65) | 0.56 (0.23-0.89) |
| Hall et al 2002), South Africa | 24 h urine increased by ≥ 2g | pulmonary oedema | 74 | 1 (1.40) | 0.00 (0.00-0.95) | 0.60 (0.48-0.71) | NA | NA | NA | 1.70 (1.60-1.70) | NA |
| Leanos-Miranda et al (2020), Mexico | sFlt-1/PlGF ratio ≥ 38 | pulmonary oedema (clinical diagnosis and with radiographic confirmation) | 810 | 6 (0.74) | 1.00 (0.61-1.00) | 0.35 (0.32-0.39) | 0.01 (0.01-0.02) | 1.00 (0.99-1.00) | 1.44 (1.16-1.77)* | 0.20 (0.01-2.93)* | 0.68 (0.66-0.69) |
| Leanos-Miranda et al (2020), Mexico | sFlt-1/PlGF ratio ≥ 85 | pulmonary oedema (clinical diagnosis and with radiographic confirmation) | 810 | 6 (0.74) | 1.00 (0.61-1.00) | 0.48 (0.45-0.51) | 0.01 (0.01-0.03) | 1.00 (0.99-1.00) | 1.79 (1.44-2.22)* | 0.15 (0.01-2.15)* | 0.74 (0.72-0.76) |
| Li et al (2018), China | 24 h Proteinuria excretion ≥ 0.3 g/24h | pulmonary oedema | 1,738 | 8 (0.46) | 0.88 (0.53-0.98) | 0.19 (0.17-0.21) | 0.00 (0.00-0.01) | 1.00 (0.98-1.00) | 1.08 (0.83-1.40) | 0.66 (0.11-4.15) | 0.53 (0.41-0.65) |
| Li et al (2018), China | 24 h Proteinuria excretion ≥ 2.0 g/24h | pulmonary oedema | 1,738 | 8 (0.46) | 0.62 (0.31-0.86) | 0.56 (0.53-0.58) | 0.01 (0.00-0.02) | 1.00 (0.99-1.00) | 1.41 (0.82-2.42) | 0.67 (0.28-1.65) | 0.59 (0.41-0.77) |
| Li et al (2018), China | 24 h Proteinuria excretion ≥ 5.0 g/24h | pulmonary oedema | 1,738 | 8 (0.46) | 0.12 (0.02-0.47) | 0.76 (0.74-0.78) | 0.00 (0.00-0.01) | 0.99 (0.99-1.00) | 0.52 (0.08-3.24) | 1.15 (0.89-1.50) | 0.44 (0.32-0.56) |
| Malhamé et al (2022_1), US | obstructive sleep apnea | pulmonary oedema (occurring during delivery hospitalization) | 71,159 | 139 (0.20) | 0.02 (0.01-0.06) | 1.00 (1.00-1.00) | 0.01 (0.00-0.03) | 1.00 (1.00-1.00) | 5.74 (1.86-17.70) | 0.98 (0.96-1.01) | 0.51 (0.50-0.52) |
| Morikawa et al (2020), Japan | serum total protein level at PE diagnosis ≤ 50 g/L | pulmonary oedema (at any time) | 94 | 13 (13.80) | 0.53 (0.29-0.77) | 0.76 (0.65-0.84) | 0.30 (0.15-0.49) | 0.90 (0.80-0.95) | 2.25 (1.17-4.32) | 0.61 (0.33-1.11) | 0.65 (0.50-0.80) |
| Morikawa et al (2020), Japan | serum total protein level at delivery ≤ 54 g/L | pulmonary oedema (at any time) | 94 | NA (13.80) | 1.00 | 0.47 | 0.26 | 1.00 | NA | NA | 0.787 |
| Morikawa et al (2021), Japan | gestational weight gain during the week prior delivery ≥ 1.6 kg | pulmonary oedema | 94 | 15 (15.90) | 0.80 (0.55-0.93) | 0.56 (0.45-0.66) | 0.26 (0.15-0.40) | 0.94 (0.83-0.98) | 1.81 (1.27-2.57) | 0.36 (0.13-1.01) | 0.68 (0.56-0.80) |
| Morikawa et al (2021), Japan | gestational weight gain during the week prior delivery ≥ 1.5 kg | pulmonary oedema | 94 | 15 (15.90) | 0.87 (0.62-0.96) | 0.54 (0.44-0.64) | 0.27 (0.15-0.37) | 0.96 (0.87-0.99) | 1.88 (1.39-2.54) | 0.25 (0.07-0.91) | 0.70 (0.60-0.81) |
| Romero et al (1988), US | AST 2SD above mean | pulmonary oedema | 275 | 3 (1.10) | 0.67 (0.13-0.98) | 0.79 (0.74-0.84) | NA | NA | 3.20 (1.40-7.50) | 0.40 (0.10-2.10) | NA |
| Thadhani et al (2022), US | sFlt-1/PlGF ratio ≥ 40 | pulmonary oedema (within 2 weeks of enrolment) | 556 | 7 (1.30) | 0.71 (0.36-0.92) | 0.52 (0.48-0.56) | 0.02 (0.01-0.04) | 0.99 (0.98-1.00) | 1.50 (0.93-2.41) | 0.55 (0.17-1.77) | 0.62 (0.44-0.80) |
| Wei et al (2022), China | PNI ≤ 37 | respiratory failure (serious disorder of pulmonary ventilation and/or ventilation function caused by various reason) | 733 | 1 (0.14) | 1.00 (0.21-1.00) | 0.58 (0.55-0.62) | 0.00 (0.00-0.02) | 1.00 (0.99-1.00) | 1.80 (0.80-4.02)* | 0.43 (0.04-4.73)* | NA |
| **Renal injury** | | | | | | | | | | | |
| Chadha et al (2022), India | UPCR ≥ 0.3 | renal dysfunction (serum creatinine >1.2 mg/dL) or oliguria (< 400 mg/dL)) | 141 | 20 (14.20) | 0.90 (0.70-0.97) | 0.11 (0.06-0.18) | 0.14 (0.09-0.21) | 0.87 (0.62-0.96) | 1.01 (0.86-1.18) | 0.93 (0.23-3.82) | 0.50 (0.43-0.58) |
| Chaiworapongsa et al (2023), US | abnormal angiogenic profile (sFlt-1/PlGF ratio < 10th percentile for gestational age) in early-onset pre-eclampsia (cohort case-control) | acute kidney injury (serum creatinine > 1.1 mg/dL) | 29 | 2 (6.90) | 1.00 (0.34-1.00) | 0.11 (0.04-0.28) | 0.08 (0.02-0.24) | 1.00 (0.44-1.00) | 0.95 (0.56-1.61)* | 1.33 (0.09-20.11)* | 0.56 (0.50-0.62) |
| Chaiworapongsa et al (2023), US | abnormal angiogenic profile (sFlt-1/PlGF ratio < 10th percentile for gestational age) in term pre-eclampsia (cohort case-control) | acute kidney injury (serum creatinine > 1.1 mg/dL) | 98 | 2 (2.04) | 0.50 (0.09-0.91) | 0.64 (0.54-0.72) | 0.03 (0.00-0.14) | 0.98 (0.91-1.00) | 1.37 (0.33-5.62) | 0.79 (0.20-3.17) | 0.57 (0.08-1.00) |
| Chaiworapongsa et al (2023), US | abnormal angiogenic profile (sFlt-1/PlGF ratio < 10th percentile for gestational age) in early-onset pre-eclampsia (cohort case-series) | acute kidney injury (serum creatinine > 1.1 mg/dL) | 84 | 3 (3.57) | 1.00 (0.44-1.00) | 0.02 (0.01-0.09) | 0.04 (0.01-0.10) | 1.00 (0.34-1.00) | 0.90 (0.62-1.31)* | 4.10 (0.23-72.00)* | 0.51 (0.50-0.53) |
| Chaiworapongsa et al (2023), US | abnormal angiogenic profile (sFlt-1/PlGF ratio < 10th percentile for gestational age) in term pre-eclampsia (cohort case-series) | acute kidney injury (serum creatinine > 1.1 mg/dL) | 216 | 5 (2.31) | 0.80 (0.38-0.96) | 0.45 (0.39-0.52) | 0.03 (0.01-0.08) | 0.99 (0.94-1.00) | 1.47 (0.93-2.31) | 0.44 (0.08-2.55) | 0.63 (0.43-0.83) |
| Jampana et al (2022), India | maternal serum uric acid level > 5.5 mg/dL | severe renal failure | 86 | 2 (2.33) | 0.50 (0.09-0.91) | 0.40 (0.31-0.51) | 0.02 (0.00-0.10) | 0.97 (0.85-0.99) | 0.84 (0.21-3.40) | 1.24 (0.30-5.06) | 0.45 (0.00-0.95) |
| Joshi et al (2022), India | raised serum hs-CRP > 3.0 mg/dL | acute kidney injury | 132 | 4 (3.03) | 0.75 (0.30-0.95) | 0.55 (0.47-0.64) | 0.05 (0.02-0.14) | 0.99 (0.93-1.00) | 1.68 (0.93-3.06) | 0.45 (0.08-2.48) | 0.65 (0.40-0.90) |
| Karge et al (2022), Germany | BMI ≥ 25 kg/m^2 | acute kidney injury (elevated serum creatinine > 1.1 mg/dL or a doubling of serum creatinine in the absence of other renal disease) | 141 | 15 (10.64) | 0.20 (0.07-0.45) | 0.57 (0.48-0.65) | 0.05 (0.02-0.14) | 0.86 (0.77-0.92) | 0.47 (0.17-1.31) | 1.40 (1.04-1.88) | 0.39 (0.27-0.50) |
| Karge et al (2022), Germany | BMI ≥ 30 kg/m^2 | acute kidney injury (elevated serum creatinine > 1.1 mg/dL or a doubling of serum creatinine in the absence of other renal disease) | 141 | 15 (10.64) | 0.07 (0.01-0.30) | 0.79 (0.71-0.85) | 0.04 (0.01-0.18) | 0.88 (0.80-0.92) | 0.31 (0.05-2.13) | 1.19 (1.01-1.40) | 0.43 (0.35-0.50) |
| Leanos-Miranda et al (2020), Mexico | sFlt-1/PlGF ratio ≥ 38 | acute kidney injury (creatinine > 1.5 mg/dL) | 810 | 18 (2.22) | 1.00 (0.82-1.00) | 0.36 (0.33-0.39) | 0.03 (0.02-0.05) | 1.00 (0.99-1.00) | 1.52 (1.39-1.66)* | 0.07 (0.00-1.13)* | 0.68 (0.66-0.70) |
| Leanos-Miranda et al (2020), Mexico | sFlt-1/PlGF ratio ≥ 85 | acute kidney injury (creatinine > 1.5 mg/dL) | 810 | 18 (2.22) | 1.00 (0.82-1.00) | 0.49 (0.45-0.52) | 0.04 (0.03-0.07) | 1.00 (0.99-1.00) | 1.90 (1.72-2.10)* | 0.05 (0.00-0.83)* | 0.74 (0.73-0.76) |
| Loardi et al (2021), Italy | abnormal UtA PI > 95th percentile | acute kidney injury | 311 | 2 (0.64) | 0.50 (0.09-0.91) | 0.40 (0.35-0.45) | 0.01 (0.00-0.03) | 0.99 (0.96-1.00) | 0.83 (0.21-3.33) | 1.26 (0.31-5.06) | 0.45 (0.00-0.94) |
| Malhamé et al (2022_1), US | obstructive sleep apnea | acute renal failure (occurring during delivery hospitalization) | 71,159 | 668 (0.94) | 0.00 (0.00-0.01) | 1.00 (1.00-1.00) | 0.01 (0.00-0.03) | 0.99 (0.99-0.99) | 1.19 (0.38-3.69) | 1.00 (0.99-1.00) | 0.50 (0.50-0.50) |
| Binder et al (2021), Austria | angiogenic imbalance (sFlt-1/PlGF ratio above the 97th percentile and/or PlGF levels below 2.5th centile) without proteinuria | renal insufficiency (creatinine levels > 1.7 mg/dL) | 145 | 2 (1.40) | 0.00 (0.00-0.66) | 0.72 (0.64-0.79) | 0.00 (0.00-0.09) | 0.98 (0.93-0.99) | 0.59 (0.05-7.54)* | 1.16 (0.69-1.94)* | 0.36 (0.32-0.40) |
| Binder et al (2021), Austria | angiogenic imbalance (sFlt-1/PlGF ratio above the 97th percentile and/or PlGF levels below 2.5th centile) without IUGR | renal insufficiency (creatinine levels > 1.7 mg/dL) | 145 | 2 (1.40) | 1.00 (0.34-1.00) | 0.76 (0.69-0.82) | 0.06 (0.02-0.18) | 1.00 (0.97-1.00) | 3.48 (1.94-6.24)* | 0.22 (0.02-2.76)* | 0.88 (0.85-0.92) |
| Binder et al (2021), Austria | angiogenic imbalance (sFlt-1/PlGF ratio above the 97th percentile and/or PlGF levels below 2.5th centile) and/or proteinuria | renal insufficiency (creatinine levels > 1.7 mg/dL) | 145 | 2 (1.40) | 1.00 (0.34-1.00) | 0.62 (0.53-0.69) | 0.04 (0.01-0.12) | 1.00 (0.96-1.00) | 2.16 (1.25-3.73)* | 0.27 (0.02-3.42)* | 0.81 (0.77-0.85) |
| Binder et al (2021), Austria | proteinuria, other symptoms or IUGR without angiogenic imbalance | renal insufficiency (creatinine levels > 1.7 mg/dL) | 145 | 2 (1.40) | 0.00 (0.00-0.66) | 0.92 (0.86-0.95) | 0.00 (0.00-0.24) | 0.98 (0.95-1.00) | 1.92 (0.14-25.47)* | 0.91 (0.55-1.52)* | 0.46 (0.44-0.48) |
| Govender et al (2022), South Africa | ductus venosus Doppler abnormal | renal impairment | 61 | 4 (6.56) | 0.25 (0.05-0.70) | 0.46 (0.33-0.58) | 0.03 (0.01-0.16) | 0.90 (0.74-0.96) | 0.46 (0.08-2.55) | 1.64 (0.87-3.10) | 0.35 (0.10-0.61) |
| Li et al (2018), China | 24 h Proteinuria excretion ≥ 0.3 g/24h | renal insufficiency | 1,738 | 49 (2.82) | 0.96 (0.86-0.99) | 0.19 (0.17-0.21) | 0.03 (0.03-0.04) | 0.99 (0.98-1.00) | 1.19 (1.12-1.26) | 0.21 (0.05-0.82) | 0.58 (0.55-0.61) |
| Li et al (2018), China | 24 h Proteinuria excretion ≥ 2.0 g/24h | renal insufficiency | 1,738 | 49 (2.82) | 0.69 (0.55-0.80) | 0.56 (0.54-0.59) | 0.04 (0.03-0.06) | 0.98 (0.97-0.99) | 1.59 (1.31-1.93) | 0.54 (0.36-0.83) | 0.63 (0.56-0.69) |
| Li et al (2018), China | 24 h Proteinuria excretion ≥ 5.0 g/24h | renal insufficiency | 1,738 | 49 (2.82) | 0.43 (0.30-0.57) | 0.76 (0.74-0.78) | 0.05 (0.03-0.08) | 0.98 (0.97-0.99) | 1.82 (1.30-2.54) | 0.75 (0.59-0.95) | 0.60 (0.53-0.67) |
| Thadhani et al (2022), US | sFlt-1/PlGF ratio ≥ 40 | renal insufficiency (serum creatinine > 1.4 mg/dL) | 556 | 5 (0.90) | 0.60 (0.23-0.88) | 0.52 (0.48-0.56) | 0.01 (0.00-0.03) | 0.99 (0.98-1.00) | 1.25 (0.61-2.58) | 0.77 (0.26-2.25) | 0.56 (0.32-0.80) |
| Sudjai et al (2022), Thailand | maternal serum uric acid level < 5.0 mg/dL | renal involvement (serum creatinine concentration > 1.1 mg/dL or a doubling of the serum creatinine concentration) | 400 | 29 (7.25) | 0.03 (0.01-0.17) | 0.74 (0.70-0.79) | 0.01 (0.00-0.06) | 0.91 (0.87-0.94) | 0.13 (0.02-0.93) | 1.30 (1.18-1.42) | 0.39 (0.35-0.43) |
| Sudjai et al (2022), Thailand | maternal serum uric acid level ≥ 5.0 mg/dL | renal involvement (serum creatinine concentration > 1.1 mg/dL or a doubling of the serum creatinine concentration) | 400 | 29 (7.25) | 0.97 (0.83-0.99) | 0.26 (0.21-0.30) | 0.09 (0.06-0.13) | 0.99 (0.94-1.00) | 1.30 (1.18-1.42) | 0.13 (0.02-0.93) | 0.61 (0.57-0.65) |
| Sudjai et al (2022), Thailand | maternal serum uric acid level ≥ 7.0 mg/dL | renal involvement (serum creatinine concentration > 1.1 mg/dL or a doubling of the serum creatinine concentration) | 400 | 29 (7.25) | 0.76 (0.58-0.88) | 0.73 (0.68-0.77) | 0.18 (0.12-0.25) | 0.97 (0.95-0.99) | 2.76 (2.12-3.59) | 0.33 (0.17-0.64) | 0.74 (0.66-0.82) |
| Yucesoy et al (2005), Turkey | platelets < 50000/mm^3^ | acute renal failure | 44 | 7 (15.90) | 0.71 (0.30-0.95) | 0.70 (0.53-0.84) | NA | NA | 2.40 (1.20-4.80) | 0.40 (0.10-1.30) | NA |
| **Liver dysfunction** | | | | | | | | | | | |
| Chadha et al (2022), India | UPCR ≥ 0.3 | elevated liver enzymes (AST > 40 U/L) | 141 | 29 (20.60) | 0.90 (0.74-0.96) | 0.11 (0.06-0.18) | 0.21 (0.14-0.29) | 0.80 (0.55-0.93) | 1.00 (0.87-1.15) | 0.97 (0.29-3.20) | 0.50 (0.44-0.57) |
| Chaiworapongsa et al (2023), US | abnormal angiogenic profile (sFlt-1/PlGF ratio < 10th percentile for gestational age) in early-onset pre-eclampsia (cohort case-control) | elevated liver enzymes (serum ALT ≥ 70 IU/mL) | 26 | 7 (26.92) | 0.86 (0.49-0.97) | 0.11 (0.03-0.31) | 0.26 (0.13-0.46) | 0.67 (0.21-0.94) | 0.96 (0.68-1.35) | 1.36 (0.14-12.73) | 0.48 (0.32-0.64) |
| Chaiworapongsa et al (2023), US | abnormal angiogenic profile (sFlt-1/PlGF ratio < 10th percentile for gestational age) in early-onset pre-eclampsia (cohort case-series) | elevated liver enzymes (serum ALT ≥ 70 IU/mL) | 72 | 12 (16.67) | 1.00 (0.76-1.00) | 0.03 (0.01-0.11) | 0.17 (0.10-0.28) | 1.00 (0.34-1.00) | 1.00 (0.89-1.13)* | 0.94 (0.05-18.42)* | 0.52 (0.49-0.54) |
| Chaiworapongsa et al (2023), US | abnormal angiogenic profile (sFlt-1/PlGF ratio < 10th percentile for gestational age) in intermediate pre-eclampsia (cohort case-series) | elevated liver enzymes (serum ALT ≥ 70 IU/mL) | 82 | 7 (8.54) | 0.57 (0.25-0.84) | 0.20 (0.13-0.30) | 0.06 (0.02-0.15) | 0.83 (0.61-0.94) | 0.71 (0.37-1.37) | 2.14 (0.81-5.64) | 0.39 (0.18-0.59) |
| Chaiworapongsa et al (2023), US | abnormal angiogenic profile (sFlt-1/PlGF ratio < 10th percentile for gestational age) in term pre-eclampsia (cohort case-series) | elevated liver enzymes (serum ALT ≥ 70 IU/mL) | 148 | 4 (2.70) | 1.00 (0.51-1.00) | 0.45 (0.37-0.53) | 0.05 (0.02-0.12) | 1.00 (0.94-1.00) | 1.64 (1.18-2.28)* | 0.22 (0.02-3.09)* | 0.73 (0.68-0.77) |
| Chaiworapongsa et al (2023), US | abnormal angiogenic profile (sFlt-1/PlGF ratio < 10th percentile for gestational age) in early-onset pre-eclampsia (cohort case-control) | elevated liver enzymes (serum AST ≥ 70 IU/mL) | 29 | 9 (31.03) | 0.89 (0.57-0.98) | 0.10 (0.03-0.30) | 0.31 (0.17-0.50) | 0.67 (0.21-0.94) | 0.99 (0.75-1.30) | 1.11 (0.12-10.73) | 0.49 (0.37-0.62) |
| Chaiworapongsa et al (2023), US | abnormal angiogenic profile (sFlt-1/PlGF ratio < 10th percentile for gestational age) in term pre-eclampsia (cohort case-control) | elevated liver enzymes (serum AST ≥ 70 IU/mL) | 104 | 1 (0.97) | 1.00 (0.21-1.00) | 0.61 (0.52-0.70) | 0.02 (0.00-0.13) | 1.00 (0.94-1.00) | 1.93 (0.84-4.44)* | 0.41 (0.04-4.54)* | NA |
| Chaiworapongsa et al (2023), US | abnormal angiogenic profile (sFlt-1/PlGF ratio < 10th percentile for gestational age) in early-onset pre-eclampsia (cohort case-series) | elevated liver enzymes (serum AST ≥ 70 IU/mL) | 89 | 18 (20.22) | 1.00 (0.82-1.00) | 0.03 (0.01-0.10) | 0.21 (0.14-0.30) | 1.00 (0.34-1.00) | 1.01 (0.93-1.10)* | 0.76 (0.04-15.13)* | 0.51 (0.49-0.53) |
| Chaiworapongsa et al (2023), US | abnormal angiogenic profile (sFlt-1/PlGF ratio < 10th percentile for gestational age) in intermediate pre-eclampsia (cohort case-series) | elevated liver enzymes (serum AST ≥ 70 IU/mL) | 102 | 11 (10.78) | 0.82 (0.52-0.95) | 0.21 (0.14-0.30) | 0.11 (0.06-0.20) | 0.90 (0.71-0.97) | 1.03 (0.77-1.39) | 0.87 (0.23-3.25) | 0.51 (0.39-0.64) |
| Chaiworapongsa et al (2023), US | abnormal angiogenic profile (sFlt-1/PlGF ratio < 10th percentile for gestational age) in term pre-eclampsia (cohort case-series) | elevated liver enzymes (serum AST ≥ 70 IU/mL) | 251 | 9 (3.59) | 1.00 (0.70-1.00) | 0.46 (0.40-0.52) | 0.06 (0.03-0.12) | 1.00 (0.97-1.00) | 1.76 (1.46-2.11)* | 0.11 (0.01-1.63)* | 0.73 (0.70-0.76) |
| Leanos-Miranda et al (2020), Mexico | sFlt-1/PlGF ratio ≥ 38 | abnormal liver enzymes levels (twice normal concentration) | 810 | 90 (11.11) | 0.98 (0.92-0.99) | 0.39 (0.36-0.43) | 0.17 (0.14-0.20) | 0.99 (0.97-1.00) | 1.61 (1.50-1.72) | 0.06 (0.01-0.22) | 0.68 (0.66-0.71) |
| Leanos-Miranda et al (2020), Mexico | sFlt-1/PlGF ratio ≥ 85 | abnormal liver enzymes levels (twice normal concentration) | 810 | 90 (11.11) | 0.97 (0.91-0.99) | 0.53 (0.50-0.57) | 0.21 (0.17-0.25) | 0.99 (0.98-1.00) | 2.07 (1.89-2.25) | 0.06 (0.02-0.19) | 0.75 (0.72-0.78) |
| Li et al (2018), China | 24 h Proteinuria excretion ≥ 0.3 g/24h | elevated liver enzyme | 1,738 | 186 (10.70) | 0.83 (0.77-0.88) | 0.63 (0.61-0.65) | 0.21 (0.18-0.24) | 0.97 (0.96-0.98) | 2.25 (2.05-2.46) | 0.26 (0.19-0.37) | 0.73 (0.70-0.76) |
| Li et al (2018), China | 24 h Proteinuria excretion ≥ 2.0 g/24h | elevated liver enzyme | 1,738 | 186 (10.70) | 0.49 (0.42-0.57) | 0.80 (0.77-0.81) | 0.22 (0.19-0.27) | 0.93 (0.91-0.94) | 2.41 (2.03-2.88) | 0.64 (0.55-0.73) | 0.64 (0.61-0.68) |
| Li et al (2018), China | 24 h Proteinuria excretion ≥ 5.0 g/24h | elevated liver enzyme | 1,738 | 186 (10.70) | 0.31 (0.24-0.38) | 0.77 (0.75-0.79) | 0.14 (0.11-0.17) | 0.90 (0.88-0.92) | 1.31 (1.04-1.66) | 0.90 (0.82-1.00) | 0.54 (0.50-0.57) |
| Thadhani et al (2022), US | sFlt-1/PlGF ratio ≥ 40 | liver dysfunction (AST or ALT levels > 80 U/L) | 556 | 28 (5.00) | 0.93 (0.77-0.98) | 0.54 (0.50-0.59) | 0.10 (0.07-0.14) | 0.99 (0.98-1.00) | 2.03 (1.77-2.34) | 0.13 (0.03-0.50) | 0.74 (0.68-0.79) |
| Binder et al (2021), Austria | angiogenic imbalance (sFlt-1/PlGF ratio above the 97th percentile and/or PlGF levels below 2.5th centile) without proteinuria | liver dysfunction (elevated liver enzymes with transaminase levels twice the upper limit of normal) | 145 | 5 (3.40) | 0.60 (0.23-0.88) | 0.74 (0.66-0.80) | 0.07 (0.03-0.20) | 0.98 (0.93-0.99) | 2.27 (1.05-4.89) | 0.54 (0.18-1.60) | 0.67 (0.43-0.91) |
| Binder et al (2021), Austria | angiogenic imbalance (sFlt-1/PlGF ratio above the 97th percentile and/or PlGF levels below 2.5th centile) without IUGR | liver dysfunction (elevated liver enzymes with transaminase levels twice the upper limit of normal) | 145 | 5 (3.40) | 0.60 (0.23-0.88) | 0.76 (0.69-0.83) | 0.08 (0.03-0.22) | 0.98 (0.94-0.99) | 2.55 (1.17-5.53) | 0.52 (0.18-1.54) | 0.68 (0.44-0.92) |
| Binder et al (2021), Austria | angiogenic imbalance (sFlt-1/PlGF ratio above the 97th percentile and/or PlGF levels below 2.5th centile) and/or proteinuria | liver dysfunction (elevated liver enzymes with transaminase levels twice the upper limit of normal) | 145 | 5 (3.40) | 0.80 (0.38-0.96) | 0.62 (0.54-0.70) | 0.07 (0.03-0.17) | 0.99 (0.94-1.00) | 2.11 (1.30-3.44) | 0.32 (0.06-1.87) | 0.71 (0.51-0.91) |
| Binder et al (2021), Austria | proteinuria, other symptoms or IUGR without angiogenic imbalance | liver dysfunction (elevated liver enzymes with transaminase levels twice the upper limit of normal) | 145 | 5 (3.40) | 0.00 (0.00-0.43) | 0.91 (0.86-0.95) | 0.00 (0.00-0.24) | 0.96 (0.92-0.98) | 0.94 (0.06-14.07)* | 1.01 (0.79-1.29)* | 0.46 (0.43-0.48) |
| Joshi et al (2022), India | raised serum hs-CRP > 3.0 mg/dL | acute liver injury | 132 | 18 (13.64) | 1.00 (0.82-1.00) | 0.63 (0.54-0.71) | 0.30 (0.20-0.43) | 1.00 (0.95-1.00) | 2.63 (2.05-3.38)* | 0.04 (0.00-0.65)* | 0.82 (0.77-0.86) |
| **HELLP syndrome** | | | | | | | | | | | |
| Cazarez-Ávalos et al (2019), Mexico | fullPIERS model | HELLP syndrome | 100 | 5 (5.00) | 1.00 (0.57-1.00) | 0.94 (0.87-0.97) | 0.46 (0.21-0.72) | 1.00 (0.08-1.00) | NA | NA | 0.98 (0.94-1.00) |
| Chadha et al (2022), India | UPCR ≥ 0.3 | HELLP syndrome | 141 | 17 (12.10) | 0.94 (0.73-0.99) | 0.11 (0.07-0.18) | 0.13 (0.08-0.20) | 0.93 (0.70-0.99) | 1.06 (0.93-1.21) | 0.52 (0.07-3.72) | 0.53 (0.46-0.59) |
| Chaves et al (2017), Brazil | abnormal PR (ratio of the flow velocity of the second peak to that of the initial systolic velocity peak) ≥ 0.78 | HELLP syndrome | 56 | 6 (10.71) | 0.83 (0.44-0.97) | 0.12 (0.06-0.24) | 0.10 (0.04-0.22) | 0.86 (0.49-0.97) | 0.95 (0.65-1.37) | 1.39 (0.20-9.67) | 0.48 (0.31-0.65) |
| Chaves et al (2017), Brazil | abnormal PR (ratio of the flow velocity of the second peak to that of the initial systolic velocity peak) ≥ 0.99 | HELLP syndrome | 56 | 6 (10.71) | 0.17 (0.03-0.56) | 0.70 (0.56-0.81) | 0.06 (0.01-0.28) | 0.88 (0.74-0.95) | 0.56 (0.09-3.49) | 1.19 (0.80-1.78) | 0.43 (0.26-0.61) |
| Govender et al (2022), South Africa | ductus venosus Doppler abnormal | HELLP syndrome | 61 | 13 (21.31) | 0.46 (0.23-0.71) | 0.46 (0.33-0.60) | 0.19 (0.09-0.35) | 0.76 (0.58-0.88) | 0.85 (0.45-1.62) | 1.17 (0.65-2.12) | 0.46 (0.30-0.62) |
| Jampana et al (2022), India | maternal serum uric acid level > 5.5 mg/dL | HELLP syndrome | 86 | 4 (4.65) | 0.75 (0.30-0.95) | 0.41 (0.31-0.52) | 0.06 (0.02-0.16) | 0.97 (0.85-0.99) | 1.28 (0.71-2.32) | 0.60 (0.11-3.36) | 0.58 (0.33-0.83) |
| Joshi et al (2022), India | raised serum hs-CRP > 3.0 mg/dL | HELLP syndrome | 132 | 13 (9.85) | 0.92 (0.67-0.99) | 0.60 (0.51-0.68) | 0.20 (0.12-0.32) | 0.99 (0.93-1.00) | 2.29 (1.75-2.99) | 0.13 (0.02-0.85) | 0.76 (0.67-0.85) |
| Karge et al (2022), Germany | BMI ≥ 25 kg/m^2 | early-onset HELLP syndrome | 141 | 10 (7.09) | 0.20 (0.06-0.51) | 0.60 (0.52-0.68) | 0.04 (0.01-0.12) | 0.91 (0.84-0.96) | 0.51 (0.14-1.78) | 1.32 (0.94-1.86) | 0.40 (0.27-0.54) |
| Karge et al (2022), Germany | BMI ≥ 25 kg/m^2 | late-onset HELLP syndrome | 141 | 9 (6.38) | 0.22 (0.06-0.55) | 0.58 (0.50-0.66) | 0.04 (0.01-0.12) | 0.92 (0.84-0.96) | 0.53 (0.15-1.84) | 1.33 (0.91-1.95) | 0.40 (0.25-0.55) |
| Karge et al (2022), Germany | BMI ≥ 30 kg/m^2 | early-onset HELLP syndrome | 141 | 10 (7.09) | 0.20 (0.06-0.51) | 0.80 (0.73-0.86) | 0.07 (0.02-0.23) | 0.93 (0.87-0.96) | 1.01 (0.28-3.65) | 1.00 (0.72-1.38) | 0.50 (0.37-0.64) |
| Karge et al (2022), Germany | BMI ≥ 30 kg/m^2 | late-onset HELLP syndrome | 141 | 9 (6.38) | 0.11 (0.02-0.43) | 0.80 (0.72-0.86) | 0.04 (0.01-0.18) | 0.93 (0.87-0.96) | 0.54 (0.08-3.55) | 1.12 (0.87-1.43) | 0.45 (0.34-0.57) |
| Kesireddy et al (2021), India | GlyFn > 350 µg/mL | HELLP syndrome | 51 | 4 (7.84) | 1.00 (0.51-1.00) | 0.45 (0.31-0.59) | 0.13 (0.05-0.30) | 1.00 (0.85-1.00) | 1.63 (1.11-2.40)* | 0.22 (0.02-3.15)* | 0.72 (0.65-0.80) |
| Leanos-Miranda et al (2020), Mexico | sFlt-1/PlGF ratio ≥ 38 | HELLP syndrome | 810 | 51 (6.30) | 1.00 (0.93-1.00) | 0.37 (0.34-0.41) | 0.10 (0.07-0.13) | 1.00 (0.99-1.00) | 1.58 (1.49-1.68)* | 0.03 (0.00-0.41)* | 0.69 (0.67-0.70) |
| Leanos-Miranda et al (2020), Mexico | sFlt-1/PlGF ratio ≥ 85 | HELLP syndrome | 810 | 51 (6.30) | 1.00 (0.93-1.00) | 0.51 (0.47-0.54) | 0.12 (0.09-0.15) | 1.00 (0.99-1.00) | 2.02 (1.87-2.18)* | 0.02 (0.00-0.30)* | 0.75 (0.74-0.77) |
| Loardi et al (2021), Italy | abnormal UtA PI > 95th percentile | HELLP syndrome | 311 | 34 (10.93) | 0.74 (0.57-0.85) | 0.42 (0.36-0.47) | 0.13 (0.09-0.19) | 0.93 (0.87-0.96) | 1.26 (1.00-1.57) | 0.64 (0.36-1.14) | 0.58 (0.49-0.66) |
| Sudjai et al (2022), Thailand | maternal serum uric acid level < 5.0 mg/dL | HELLP syndrome | 400 | 2 (0.50) | 0.00 (0.00-0.66) | 0.76 (0.71-0.80) | 0.00 (0.00-0.04) | 0.99 (0.98-1.00) | 0.69 (0.05-8.71)* | 1.10 (0.66-1.83)* | 0.38 (0.36-0.40) |
| Sudjai et al (2022), Thailand | maternal serum uric acid level ≥ 5.0 mg/dL | HELLP syndrome | 400 | 2 (0.50) | 1.00 (0.34-1.00) | 0.24 (0.20-0.29) | 0.01 (0.00-0.02) | 1.00 (0.96-1.00) | 1.10 (0.66-1.83)* | 0.69 (0.05-8.71)* | 0.62 (0.60-0.64) |
| Sudjai et al (2022), Thailand | maternal serum uric acid level ≥ 7.0 mg/dL | HELLP syndrome | 400 | 2 (0.50) | 0.50 (0.09-0.91) | 0.69 (0.64-0.73) | 0.01 (0.00-0.04) | 1.00 (0.98-1.00) | 1.62 (0.40-6.52) | 0.72 (0.18-2.90) | 0.60 (0.10-1.00) |
| Vasquez et al (2021), Argentina | epigastric pain | HELLP syndrome | 172 | 38 (22.09) | 0.57 (0.41-0.72) | 0.69 (0.61-0.76) | 0.33 (0.23-0.46) | 0.86 (0.76-0.91) | 1.87 (1.30-2.69) | 0.61 (0.41-0.90) | 0.63 (0.55-0.72) |
| Vasquez et al (2021), Argentina | nausea and vomiting | HELLP syndrome | 172 | 38 (22.09) | 0.18 (0.09-0.33) | 0.90 (0.83-0.94) | 0.33 (0.17-0.55) | 0.79 (0.72-0.85) | 1.84 (0.79-4.29) | 0.91 (0.77-1.07) | 0.54 (0.47-0.61) |
| Vasquez et al (2021), Argentina | renal disturbances (increment of creatinine ≥ 0.9 mg/dL, RIFLE R, RIFLE I, RIFLE F, or end-stage renal disease) | HELLP syndrome | 172 | 38 (22.09) | 0.54 (0.38-0.69) | 0.69 (0.60-0.76) | 0.32 (0.22-0.45) | 0.84 (0.76-0.90) | 1.78 (1.21-2.62) | 0.65 (0.45-0.94) | 0.62 (0.53-0.71) |
| Vasquez et al (2021), Argentina | visual symptoms (photopsias or scotomas, blurred vision, photophobia, or blindness) | HELLP syndrome | 172 | 38 (22.09) | 0.51 (0.36-0.67) | 0.54 (0.45-0.62) | 0.23 (0.15-0.34) | 0.80 (0.71-0.87) | 1.08 (0.75-1.56) | 0.93 (0.65-1.32) | 0.52 (0.43-0.61) |
| Vasquez et al (2021), Argentina | right upper quadrant pain | HELLP syndrome | 172 | 38 (22.09) | 0.26 (0.14-0.42) | 0.93 (0.87-0.96) | 0.50 (0.29-0.71) | 0.83 (0.75-0.88) | 3.87 (1.74-8.62) | 0.79 (0.65-0.96) | 0.60 (0.52-0.67) |
| Vasquez et al (2021), Argentina | hyperreflexia | HELLP syndrome | 172 | 38 (22.09) | 0.23 (0.12-0.39) | 0.64 (0.55-0.72) | 0.15 (0.07-0.26) | 0.75 (0.67-0.83) | 0.66 (0.36-1.21) | 1.19 (0.96-1.48) | 0.44 (0.36-0.52) |
| Vasquez et al (2021), Argentina | headache | HELLP syndrome | 172 | 38 (22.09) | 0.49 (0.33-0.64) | 0.37 (0.29-0.45) | 0.17 (0.11-0.26) | 0.73 (0.61-0.82) | 0.79 (0.56-1.12) | 1.35 (0.92-1.99) | 0.43 (0.34-0.52) |
| Wei et al (2022), China | PNI ≤ 37 | HELLP syndrome | 733 | 24 (3.27) | 0.75 (0.55-0.88) | 0.59 (0.56-0.63) | 0.06 (0.04-0.09) | 0.99 (0.97-0.99) | 1.85 (1.44-2.36) | 0.42 (0.21-0.84) | 0.67 (0.58-0.76) |
| Cai et al (2016), China | urinary congophilia (CapCord Test) | HELLP syndrome | 102 | 18 (17.65) | 0.94 (0.74-0.99) | 0.17 (0.10-0.26) | 0.20 (0.13-0.29) | 0.93 (0.70-0.99) | 1.13 (0.98-1.31) | 0.33 (0.05-2.38) | 0.56 (0.49-0.62) |
| **DIC** | | | | | | | | | | | |
| Joshi et al (2022), India | raised serum hs-CRP > 3.0 mg/dL | DIC | 132 | 4 (3.03) | 1.00 (0.51-1.00) | 0.56 (0.48-0.65) | 0.07 (0.03-0.16) | 1.00 (0.95-1.00) | 2.05 (1.45-2.92)* | 0.18 (0.01-2.48)* | 0.78 (0.74-0.82) |
| Lei et al (2021), China | 24 h Proteinuria excretion < 0.3 g/24h | DIC | 275 | 2 (0.70) | 0.00 (0.00-0.66) | 0.81 (0.75-0.85) | 0.00 (0.00-0.07) | 0.99 (0.97-1.00) | 0.85 (0.07-10.84)* | 1.04 (0.62-1.72)* | 0.40 (0.38-0.43) |
| Lei et al (2021), China | 24 h Proteinuria excretion ≥ 0.3 g/24h | DIC | 275 | 2 (0.70) | 0.00 (0.00-0.66) | 0.57 (0.51-0.63) | 0.00 (0.00-0.03) | 0.99 (0.96-1.00) | 0.39 (0.03-4.90)* | 1.46 (0.87-2.45)* | 0.29 (0.26-0.32) |
| Lei et al (2021), China | 24 h Proteinuria excretion ≥2 g/24h | DIC | 275 | 2 (0.70) | 0.00 (0.00-0.66) | 0.81 (0.75-0.85) | 0.00 (0.00-0.07) | 0.99 (0.97-1.00) | 0.85 (0.07-10.84)* | 1.04 (0.62-1.72)* | 0.40 (0.38-0.43) |
| Loardi et al (2021), Italy | abnormal UtA PI > 95th percentile | DIC | 311 | 7 (2.25) | 1.00 (0.65-1.00) | 0.41 (0.35-0.46) | 0.04 (0.02-0.08) | 1.00 (0.97-1.00) | 1.58 (1.29-1.94)* | 0.15 (0.01-2.25)* | 0.70 (0.68-0.73) |
| Thadhani et al (2022), US | sFlt-1/PlGF ratio ≥ 40 | DIC (within 2 weeks of enrolment) | 556 | 2 (0.40) | 1.00 (0.34-1.00) | 0.52 (0.48-0.56) | 0.01 (0.00-0.03) | 1.00 (0.99-1.00) | 1.74 (1.04-2.91)* | 0.32 (0.03-4.02)* | 0.76 (0.74-0.78) |
| Yucesoy et al (2005), Turkey | platelets < 50000/mm^3^ | DIC | 44 | 8 (18.20) | 0.75 (0.36-0.96) | 0.72 (0.55-0.85) | NA | NA | 2.70 (1.40-5.20) | 0.30 (0.10-1.20) | NA |
| **Haematological outcomes** | | | | | | | | | | | |
| Fishel Bartal et al (2022), US | Ethnicity: Non-Hispanic White | blood transfusion | 389,054 | 2568 (0.66) | 0.42 (0.40-0.44) | 0.50 (0.50-0.50) | 0.01 (0.01-0.01) | 0.99 (0.99-0.99) | 0.84 (0.80-0.88) | 1.16 (1.12-1.20) | 0.46 (0.45-0.47) |
| Fishel Bartal et al (2022), US | Ethnicity: Non-Hispanic Black | blood transfusion | 389,054 | 2568 (0.66) | 0.36 (0.34-0.38) | 0.70 (0.70-0.71) | 0.01 (0.01-0.01) | 0.99 (0.99-0.99) | 1.21 (1.15-1.28) | 0.91 (0.89-0.94) | 0.53 (0.52-0.54) |
| Fishel Bartal et al (2022), US | Ethnicity: Hispanic | blood transfusion | 389,054 | 2568 (0.66) | 0.16 (0.15-0.18) | 0.85 (0.84-0.85) | 0.01 (0.01-0.01) | 0.99 (0.99-0.99) | 1.04 (0.96-1.14) | 0.99 (0.98-1.01) | 0.50 (0.50-0.51) |
| Fishel Bartal et al (2022), US | Ethnicity: Non-Hispanic Asian American, Native Hawaiian and other Pacific Islander | blood transfusion | 389,054 | 2568 (0.66) | 0.04 (0.03-0.05) | 0.96 (0.96-0.96) | 0.01 (0.01-0.01) | 0.99 (0.99-0.99) | 1.17 (0.97-1.41) | 0.99 (0.99-1.00) | 0.50 (0.50-0.51) |
| Fishel Bartal et al (2022), US | Ethnicity: Non-Hispanic American Indian and Alaska Native | blood transfusion | 389,054 | 2568 (0.66) | 0.02 (0.01-0.02) | 0.99 (0.99-0.99) | 0.01 (0.01-0.01) | 0.99 (0.99-0.99) | 1.40 (1.03-1.92) | 1.00 (0.99-1.00) | 0.50 (0.50-0.50) |
| Morikawa et al (2021), Japan | gestational weight gain during the week prior delivery ≥ 1.5 kg | Blood transfusion | 94 | 5 (5.30) | 0.40 (0.12-0.77) | 0.49 (0.39-0.60) | 0.04 (0.01-0.14) | 0.94 (0.83-0.98) | 0.79 (0.27-2.36) | 1.21 (0.58-2.56) | 0.45 (0.20-0.69) |
| Ghosh et al (2012), India | serum PlGF < 122 pg/mL | PPH | 766 | 67 (8.70) | 0.73 (0.61-0.83) | 0.77 (0.73-0.80) | NA | NA | 3.14 (2.57-3.82) | 0.35 (0.24-0.52) | NA |
| Jampana et al (2022), India | maternal serum uric acid level > 5.5 mg/dL | PPH | 86 | 5 (5.81) | 0.80 (0.38-0.96) | 0.42 (0.32-0.53) | 0.08 (0.03-0.18) | 0.97 (0.85-0.99) | 1.38 (0.86-2.22) | 0.48 (0.08-2.80) | 0.61 (0.41-0.81) |
| Karge et al (2022), Germany | BMI ≥ 25 kg/m^2 | PPH | 141 | 2 (1.42) | 0.50 (0.09-0.91) | 0.60 (0.51-0.67) | 0.02 (0.00-0.09) | 0.99 (0.94-1.00) | 1.24 (0.31-5.04) | 0.84 (0.21-3.37) | 0.55 (0.06-1.00) |
| Karge et al (2022), Germany | BMI ≥ 30 kg/m^2 | PPH | 141 | 2 (1.42) | 0.50 (0.09-0.91) | 0.81 (0.73-0.86) | 0.04 (0.01-0.18) | 0.99 (0.95-1.00) | 2.57 (0.62-10.72) | 0.62 (0.15-2.49) | 0.65 (0.16-1.00) |
| Lei et al (2021), China | 24 h Proteinuria excretion < 0.3 g/24h | PPH | 275 | 3 (1.00) | 0.00 (0.00-0.56) | 0.81 (0.75-0.85) | 0.00 (0.00-0.07) | 0.99 (0.96-1.00) | 0.64 (0.05-8.62)* | 1.09 (0.75-1.58)* | 0.40 (0.38-0.43) |
| Lei et al (2021), China | 24 h Proteinuria excretion ≥ 0.3 g/24h | PPH | 275 | 3 (1.00) | 0.33 (0.06-0.79) | 0.57 (0.51-0.63) | 0.01 (0.00-0.05) | 0.99 (0.96-1.00) | 0.78 (0.16-3.90) | 1.16 (0.52-2.60) | 0.45 (0.13-0.78) |
| Lei et al (2021), China | 24 h Proteinuria excretion ≥ 2.0 g/24h | PPH | 275 | 3 (1.00) | 0.00 (0.00-0.56) | 0.81 (0.75-0.85) | 0.00 (0.00-0.07) | 0.99 (0.96-1.00) | 0.64 (0.05-8.62)* | 1.09 (0.75-1.58)* | 0.40 (0.38-0.43) |
| Ma et al (2019), China | serum fibrinogen ≥ 3.04 g/L | PPH (amount of bleeding in vaginal delivery ≥500 mL, and cesarean section ≥1000 mL) | 127 | 14 (11.02) | 0.71 (0.45-0.88) | 0.75 (0.48-0.89) | 0.71 (0.45-0.88) | 0.74 (0.48-0.89) | 2.68 (1.09-6.60) | 0.39 (0.16-0.94) | 0.72 (0.56-0.89) |
| Ma et al (2019), China | prothrombin time | PPH (amount of bleeding in vaginal delivery ≥500 mL, and cesarean section ≥1000 mL) | 127 | 14 (11.02) | NA | NA | NA | NA | NA | NA | 0.60 (0.51-0.68) |
| Ma et al (2019), China | activated partial thromboplastin time | PPH (amount of bleeding in vaginal delivery ≥500 mL, and cesarean section ≥1000 mL) | 127 | 14 (11.02) | NA | NA | NA | NA | NA | NA | 0.62 (0.53-0.70) |
| Ma et al (2019), China | serum D-dimer | PPH (amount of bleeding in vaginal delivery ≥500 mL, and cesarean section ≥1000 mL) | 127 | 14 (11.02) | NA | NA | NA | NA | NA | NA | 0.51 (0.41-0.60) |
| Sudjai et al (2022), Thailand | maternal serum uric acid level < 5.0 mg/dL | PPH | 400 | 11 (2.75) | 0.00 (0.00-0.26) | 0.75 (0.71-0.79) | 0.00 (0.00-0.04) | 0.96 (0.94-0.98) | 0.17 (0.01-2.55)* | 1.27 (1.12-1.45)* | 0.38 (0.36-0.40) |
| Sudjai et al (2022), Thailand | maternal serum uric acid level ≥ 5.0 mg/dL | PPH | 400 | 11 (2.75) | 1.00 (0.74-1.00) | 0.25 (0.21-0.29) | 0.04 (0.02-0.06) | 1.00 (0.96-1.00) | 1.27 (1.12-1.45)* | 0.17 (0.01-2.55)* | 0.62 (0.60-0.64) |
| Sudjai et al (2022), Thailand | maternal serum uric acid level ≥ 7.0 mg/dL | PPH | 400 | 11 (2.75) | 0.36 (0.15-0.65) | 0.69 (0.64-0.74) | 0.03 (0.01-0.08) | 0.97 (0.95-0.99) | 1.18 (0.53-2.61) | 0.92 (0.59-1.45) | 0.53 (0.38-0.68) |
| Wei et al (2022), China | PNI ≤ 37 | PPH (amount of bleeding in vaginal delivery ≥500 mL, and cesarean section ≥1000 within 24 hours) | 733 | 64 (8.73) | 0.42 (0.31-0.54) | 0.58 (0.55-0.62) | 0.09 (0.06-0.13) | 0.91 (0.88-0.94) | 1.01 (0.75-1.37) | 0.99 (0.80-1.23) | 0.50 (0.44-0.57) |
| Ye et al (2020), China | retinopathy staging group 3/4 | postpartum haemorrhage | 534 | 25 (4.68) | 0.04 (0.01-0.20) | 0.85 (0.81-0.88) | 0.01 (0.00-0.07) | 0.95 (0.92-0.96) | 0.26 (0.04-1.82) | 1.13 (1.04-1.24) | 0.44 (0.40-0.49) |
| Lei et al (2021), China | 24 h Proteinuria excretion < 0.3 g/24h | hemorrhagic anemia | 275 | 6 (2.20) | 0.00 (0.00-0.39) | 0.80 (0.75-0.85) | 0.00 (0.00-0.07) | 0.97 (0.94-0.99) | 0.36 (0.02-5.27)* | 1.16 (0.94-1.43)* | 0.40 (0.38-0.43) |
| Lei et al (2021), China | 24 h Proteinuria excretion ≥ 0.3 g/24h | hemorrhagic anemia | 275 | 6 (2.20) | 0.17 (0.03-0.56) | 0.57 (0.51-0.63) | 0.01 (0.00-0.05) | 0.97 (0.93-0.99) | 0.39 (0.06-2.33) | 1.47 (1.01-2.13) | 0.37 (0.20-0.53) |
| Lei et al (2021), China | 24 h Proteinuria excretion ≥ 2.0 g/24h | hemorrhagic anemia | 275 | 6 (2.20) | 0.17 (0.03-0.56) | 0.81 (0.76-0.85) | 0.02 (0.00-0.10) | 0.98 (0.95-0.99) | 0.86 (0.14-5.25) | 1.03 (0.72-1.48) | 0.49 (0.32-0.65) |
| Chadha et al (2022), India | UPCR ≥ 0.3 | thrombocytopenia (platelet count < 150x10^9/L) | 141 | 42 (29.80) | 0.98 (0.88-1.00) | 0.14 (0.09-0.22) | 0.33 (0.25-0.41) | 0.93 (0.70-0.99) | 1.14 (1.04-1.25) | 0.17 (0.02-1.24) | 0.56 (0.52-0.60) |
| Chaiworapongsa et al (2023), US | abnormal angiogenic profile (sFlt-1/PlGF ratio < 10th percentile for gestational age) in early-onset pre-eclampsia (cohort case-control) | thrombocytopenia (platelet count < 100x10^9/L) | 29 | 3 (10.34) | 1.00 (0.44-1.00) | 0.12 (0.04-0.29) | 0.12 (0.04-0.29) | 1.00 (0.44-1.00) | 1.01 (0.68-1.50)* | 0.96 (0.06-15.40)* | 0.56 (0.50-0.62) |
| Chaiworapongsa et al (2023), US | abnormal angiogenic profile (sFlt-1/PlGF ratio < 10th percentile for gestational age) in intermediate pre-eclampsia (cohort case-control) | thrombocytopenia (platelet count < 100x10^9/L) | 15 | 1 (6.67) | 1.00 (0.21-1.00) | 0.00 (0.00-0.22) | 0.07 (0.01-0.30) | NA | 0.78 (0.35-1.74)* | 7.50 (0.20-283.32)* | NA |
| Chaiworapongsa et al (2023), US | abnormal angiogenic profile (sFlt-1/PlGF ratio < 10th percentile for gestational age) in term pre-eclampsia (cohort case-control) | thrombocytopenia (platelet count < 100x10^9/L) | 106 | 4 (3.77) | 0.75 (0.30-0.95) | 0.63 (0.53-0.72) | 0.07 (0.03-0.19) | 0.98 (0.92-1.00) | 2.01 (1.08-3.74) | 0.40 (0.07-2.19) | 0.69 (0.44-0.94) |
| Chaiworapongsa et al (2023), US | abnormal angiogenic profile (sFlt-1/PlGF ratio < 10th percentile for gestational age) in early-onset pre-eclampsia (cohort case-series) | thrombocytopenia (platelet count < 100x10^9/L) | 89 | 16 (17.98) | 1.00 (0.81-1.00) | 0.03 (0.01-0.09) | 0.18 (0.12-0.28) | 1.00 (0.34-1.00) | 1.00 (0.92-1.10)* | 0.87 (0.04-17.32)* | 0.51 (0.49-0.53) |
| Chaiworapongsa et al (2023), US | abnormal angiogenic profile (sFlt-1/PlGF ratio < 10th percentile for gestational age) in intermediate pre-eclampsia (cohort case-series) | thrombocytopenia (platelet count < 100x10^9/L) | 104 | 12 (11.54) | 0.83 (0.55-0.95) | 0.21 (0.14-0.30) | 0.12 (0.07-0.21) | 0.90 (0.71-0.97) | 1.05 (0.80-1.38) | 0.81 (0.21-3.04) | 0.52 (0.40-0.64) |
| Chaiworapongsa et al (2023), US | abnormal angiogenic profile (sFlt-1/PlGF ratio < 10th percentile for gestational age) in term pre-eclampsia (cohort case-series) | thrombocytopenia (platelet count < 100x10^9/L) | 258 | 7 (2.71) | 0.71 (0.36-0.92) | 0.46 (0.40-0.52) | 0.04 (0.02-0.08) | 0.98 (0.94-1.00) | 1.32 (0.81-2.13) | 0.62 (0.19-2.03) | 0.59 (0.40-0.77) |
| Leanos-Miranda et al (2020), Mexico | sFlt-1/PlGF ratio ≥ 38 | thrombocytopenia (platelet count < 100x10^9/L) | 810 | 44 (5.43) | 0.98 (0.88-1.00) | 0.37 (0.34-0.40) | 0.08 (0.06-0.11) | 1.00 (0.98-1.00) | 1.55 (1.44-1.66) | 0.06 (0.01-0.43) | 0.67 (0.65-0.70) |
| Leanos-Miranda et al (2020), Mexico | sFlt-1/PlGF ratio ≥ 85 | thrombocytopenia (platelet count < 100x10^9/L) | 810 | 44 (5.43) | 0.95 (0.85-0.99) | 0.50 (0.47-0.54) | 0.10 (0.07-0.13) | 0.99 (0.98-1.00) | 1.91 (1.74-2.11) | 0.09 (0.02-0.35) | 0.73 (0.69-0.76) |
| Sudjai et al (2022), Thailand | maternal serum uric acid level < 5.0 mg/dL | thrombocytopenia | 400 | 9 (2.25) | 0.00 (0.00-0.30) | 0.75 (0.71-0.79) | 0.00 (0.00-0.04) | 0.97 (0.94-0.98) | 0.20 (0.01-3.04)* | 1.26 (1.08-1.47)* | 0.38 (0.36-0.40) |
| Sudjai et al (2022), Thailand | maternal serum uric acid level ≥ 5.0 mg/dL | thrombocytopenia | 400 | 9 (2.25) | 1.00 (0.70-1.00) | 0.25 (0.21-0.29) | 0.03 (0.02-0.06) | 1.00 (0.96-1.00) | 1.26 (1.08-1.47)* | 0.20 (0.01-3.04)* | 0.62 (0.60-0.64) |
| Sudjai et al (2022), Thailand | maternal serum uric acid level ≥ 7.0 mg/dL | thrombocytopenia | 400 | 9 (2.25) | 0.56 (0.27-0.81) | 0.70 (0.65-0.74) | 0.04 (0.02-0.09) | 0.99 (0.96-0.99) | 1.83 (1.00-3.34) | 0.64 (0.31-1.33) | 0.63 (0.45-0.80) |
| Thadhani et al (2022), US | sFlt-1/PlGF ratio ≥ 40 | thrombocytopenia (platelet count < 100,000/µl) within 2 weeks of enrolment | 556 | 10 (1.80) | 0.90 (0.60-0.98) | 0.53 (0.49-0.57) | 0.03 (0.02-0.06) | 1.00 (0.98-1.00) | 1.90 (1.52-2.38) | 0.19 (0.03-1.22) | 0.71 (0.61-0.81) |
| Ankumah et al (2014), US | SBP and/or DBP > 140/90 mmHg | placental abruption | 759 | 11 (1.40) | 0.36 (0.12-0.68) | 0.63 (0.59-0.66) | NA | NA | 1.00 (0.40-2.10) | 1.00 (0.60-1.90) | NA |
| Bouzari et al (2014), Iran | 24 h urine > 1.75 g/d | placental abruption | 298 | 18 (5.90) | 0.94 (0.69-1.00) | 0.64 (0.58-0.69) | NA | NA | 2.60 (2.10-3.10) | 0.10 (0.00-0.60) | 0.78 |
| Carter et al (2017), US | SGA (birthweight less than 10th% on the Alexander growth standard) | placental abruption | 902 | 37 (4.10) | 0.14 (0.06-0.28) | 0.83 (0.80-0.85) | 0.03 (0.01-0.07) | 0.96 (0.94-0.97) | 0.77 (0.34-1.77) | 1.05 (0.92-1.19) | 0.48 (0.42-0.54) |
| Chadha et al (2022), India | UPCR ≥ 0.3 | placental abruption (haemorrhage where the bleeding occurs due to premature separation of normally situated placenta) | 141 | 12 (8.50) | 0.92 (0.65-0.99) | 0.11 (0.07-0.17) | 0.09 (0.05-0.15) | 0.93 (0.70-0.99) | 1.03 (0.86-1.23) | 0.77 (0.11-5.35) | 0.51 (0.43-0.60) |
| Chaiworapongsa et al (2023), US | abnormal angiogenic profile (sFlt-1/PlGF ratio < 10th percentile for gestational age) in early-onset pre-eclampsia (cohort case-control) | placental abruption | 29 | 6 (20.69) | 0.83 (0.44-0.97) | 0.09 (0.02-0.27) | 0.19 (0.09-0.38) | 0.67 (0.21-0.94) | 0.91 (0.62-1.33) | 1.92 (0.21-17.75) | 0.46 (0.29-0.63) |
| Chaiworapongsa et al (2023), US | abnormal angiogenic profile (sFlt-1/PlGF ratio < 10th percentile for gestational age) in intermediate pre-eclampsia (cohort case-control) | placental abruption | 16 | 1 (6.25) | 1.00 (0.21-1.00) | 0.00 (0.00-0.20) | 0.06 (0.01-0.28) | NA | 0.77 (0.35-1.73)* | 8.00 (0.21-302.87)* | NA |
| Chaiworapongsa et al (2023), US | abnormal angiogenic profile (sFlt-1/PlGF ratio < 10th percentile for gestational age) in early-onset pre-eclampsia (cohort case-series) | placental abruption | 89 | 6 (6.74) | 1.00 (0.61-1.00) | 0.02 (0.01-0.08) | 0.07 (0.03-0.14) | 1.00 (0.34-1.00) | 0.96 (0.78-1.18)* | 2.40 (0.13-45.25)* | 0.51 (0.50-0.53) |
| Chaiworapongsa et al (2023), US | abnormal angiogenic profile (sFlt-1/PlGF ratio < 10th percentile for gestational age) in intermediate pre-eclampsia (cohort case-series) | placental abruption | 105 | 2 (1.90) | 1.00 (0.34-1.00) | 0.20 (0.14-0.29) | 0.02 (0.01-0.08) | 1.00 (0.85-1.00) | 1.05 (0.63-1.76)* | 0.81 (0.06-10.41)* | 0.60 (0.56-0.64) |
| Chaiworapongsa et al (2023), US | abnormal angiogenic profile (sFlt-1/PlGF ratio < 10th percentile for gestational age) in term pre-eclampsia (cohort case-series) | placental abruption | 258 | 2 (0.78) | 1.00 (0.34-1.00) | 0.46 (0.40-0.52) | 0.01 (0.00-0.05) | 1.00 (0.97-1.00) | 1.54 (0.91-2.58)* | 0.36 (0.03-4.59)* | 0.73 (0.70-0.76) |
| Govender et al (2022), South Africa | ductus venosus Doppler abnormal | placental abruption | 61 | 8 (13.11) | 0.88 (0.53-0.98) | 0.53 (0.40-0.66) | 0.22 (0.11-0.39) | 0.97 (0.83-0.99) | 1.85 (1.26-2.73) | 0.24 (0.04-1.51) | 0.70 (0.56-0.84) |
| Hall et al (2002), South Africa | 24 h urine increased by ≥ 2 g | placental abruption | 74 | 10 (13.50) | 0.30 (0.08-0.65) | 0.59 (0.46-0.71) | NA | NA | 0.70 (0.30-2.00) | 1.20 (0.80-1.80) | NA |
| Jampana et al (2022), India | maternal serum uric acid level > 5.5 mg/dL | placental abruption | 86 | 6 (6.98) | 0.67 (0.30-0.90) | 0.41 (0.31-0.52) | 0.08 (0.03-0.18) | 0.94 (0.81-0.98) | 1.13 (0.63-2.06) | 0.81 (0.25-2.58) | 0.54 (0.33-0.75) |
| Joshi et al (2022), India | raised serum hs-CRP > 3.0 mg/dL | placental abruption | 132 | 5 (3.79) | 1.00 (0.57-1.00) | 0.57 (0.48-0.65) | 0.08 (0.04-0.18) | 1.00 (0.95-1.00) | 2.11 (1.55-2.89)* | 0.15 (0.01-2.10)* | 0.78 (0.74-0.83) |
| Karge et al (2022), Germany | BMI ≥ 25 kg/m^2 | placental abruption | 141 | 3 (2.13) | 0.00 (0.00-0.56) | 0.59 (0.50-0.67) | 0.00 (0.00-0.06) | 0.96 (0.90-0.99) | 0.30 (0.02-4.07)* | 1.49 (1.00-2.22)* | 0.29 (0.25-0.33) |
| Karge et al (2022), Germany | BMI ≥ 30 kg/m^2 | placental abruption | 141 | 3 (2.13) | 0.00 (0.00-0.56) | 0.80 (0.72-0.86) | 0.00 (0.00-0.12) | 0.97 (0.92-0.99) | 0.61 (0.04-8.32)* | 1.10 (0.75-1.61)* | 0.40 (0.36-0.43) |
| Kesireddy et al (2021), India | GlyFn > 350 µg/mL | placental abruption | 51 | 3 (5.88) | 0.67 (0.21-0.94) | 0.42 (0.29-0.56) | 0.07 (0.02-0.21) | 0.95 (0.77-0.99) | 1.14 (0.50-2.63) | 0.80 (0.16-4.10) | 0.54 (0.21-0.88) |
| Leanos-Miranda et al (2020), Mexico | sFlt-1/PlGF ratio ≥ 38 | placental abruption (clinical or pathological) | 810 | 36 (4.44) | 1.00 (0.90-1.00) | 0.37 (0.33-0.40) | 0.07 (0.05-0.09) | 1.00 (0.99-1.00) | 1.56 (1.46-1.66)* | 0.04 (0.00-0.58)* | 0.68 (0.67-0.70) |
| Leanos-Miranda et al (2020), Mexico | sFlt-1/PlGF ratio ≥ 85 | placental abruption (clinical or pathological) | 810 | 36 (4.44) | 1.00 (0.90-1.00) | 0.50 (0.46-0.53) | 0.08 (0.06-0.12) | 1.00 (0.99-1.00) | 1.97 (1.82-2.13)* | 0.03 (0.00-0.43)* | 0.75 (0.73-0.77) |
| Lei et al (2021), China | 24 h Proteinuria excretion < 0.3 g/24h | placental abruption | 275 | 10 (3.60) | 0.10 (0.02-0.40) | 0.80 (0.75-0.85) | 0.02 (0.00-0.10) | 0.96 (0.92-0.98) | 0.51 (0.08-3.32) | 1.12 (0.90-1.39) | 0.45 (0.35-0.55) |
| Lei et al (2021), China | 24 h Proteinuria excretion ≥ 0.3 g/24h | placental abruption | 275 | 10 (3.60) | 0.40 (0.17-0.69) | 0.57 (0.51-0.63) | 0.03 (0.01-0.08) | 0.96 (0.92-0.98) | 0.94 (0.43-2.03) | 1.05 (0.62-1.75) | 0.49 (0.32-0.65) |
| Lei et al (2021), China | 24 h Proteinuria excretion ≥ 2.0 g/24h | placental abruption | 275 | 10 (3.60) | 0.10 (0.02-0.40) | 0.80 (0.75-0.85) | 0.02 (0.00-0.10) | 0.96 (0.92-0.98) | 0.51 (0.08-3.32) | 1.12 (0.90-1.39) | 0.45 (0.35-0.55) |
| Li et al (2018), China | 24 h Proteinuria excretion ≥ 0.3 g/24h | placental abruption | 1,738 | 31 (1.78) | 0.84 (0.67-0.93) | 0.19 (0.17-0.21) | 0.02 (0.01-0.03) | 0.98 (0.96-0.99) | 1.03 (0.88-1.21) | 0.85 (0.38-1.91) | 0.51 (0.45-0.58) |
| Li et al (2018), China | 24 h Proteinuria excretion ≥ 2.0 g/24h | placental abruption | 1,738 | 31 (1.78) | 0.48 (0.32-0.65) | 0.56 (0.53-0.58) | 0.02 (0.01-0.03) | 0.98 (0.97-0.99) | 1.09 (0.76-1.58) | 0.93 (0.66-1.31) | 0.52 (0.43-0.61) |
| Li et al (2018), China | 24 h Proteinuria excretion ≥ 5.0 g/24h | placental abruption | 1,738 | 31 (1.78) | 0.29 (0.16-0.47) | 0.76 (0.74-0.78) | 0.02 (0.01-0.04) | 0.98 (0.97-0.99) | 1.21 (0.69-2.11) | 0.93 (0.74-1.17) | 0.53 (0.44-0.61) |
| Loardi et al (2021), Italy | abnormal UtA PI > 95th percentile | placental abruption | 311 | 2 (0.64) | 1.00 (0.34-1.00) | 0.40 (0.35-0.46) | 0.01 (0.00-0.04) | 1.00 (0.97-1.00) | 1.39 (0.83-2.33)* | 0.41 (0.03-5.23)* | 0.70 (0.67-0.73) |
| Morikawa (2020), Japan | serum total protein level at PE diagnosis ≤ 49 g/L | placental abruption (continuously during pregnancy) | 94 | NA (3.20) | 1.00 | 0.78 | 0.13 | 1.00 | NA | NA | NA |
| Morikawa (2020), Japan | serum total protein level at delivery ≤ 49 g/L | placental abruption (continuously during pregnancy) | 94 | NA (3.20) | 1.00 | 0.68 | 0.09 | 1.00 | NA | NA | NA |
| Morikawa (2021), Japan | gestational weight gain during the week prior delivery ≥ 1.6 kg | placental abruption | 94 | 3 (3.20) | 0.67 (0.21-0.94) | 0.51 (0.40-0.61) | 0.04 (0.01-0.14) | 0.98 (0.89-1.00) | 1.35 (0.59-3.08) | 0.66 (0.13-3.31) | 0.59 (0.26-0.92) |
| Morikawa (2021), Japan | gestational weight gain during the week prior delivery ≥ 2.2 kg | placental abruption | 94 | NA (3.20) | 1.00 | 0.33 | 0.05 | 1.00 | NA | NA | NA |
| Schiff et al (1996), US | 24 h urine increased by ≥ 2g | placental abruption | 2002 | 106 (5.30) | 0.40 (0.07-0.83) | 0.64 (0.51-0.76) | NA | NA | 1.10 (0.40-3.40) | 0.90 (0.50-2.00) | NA |
| Sudjai et al (2022), Thailand | maternal serum uric acid level < 5.0 mg/dL | placental abruption | 400 | 2 (0.50) | 0.00 (0.00-0.66) | 0.76 (0.71-0.80) | 0.00 (0.00-0.04) | 0.99 (0.98-1.00) | 0.69 (0.05-8.71)* | 1.10 (0.66-1.83)* | 0.38 (0.36-0.40) |
| Sudjai et al (2022), Thailand | maternal serum uric acid level ≥ 5.0 mg/dL | placental abruption | 400 | 2 (0.50) | 1.00 (0.34-1.00) | 0.24 (0.20-0.29) | 0.01 (0.00-0.02) | 1.00 (0.96-1.00) | 1.10 (0.66-1.83)* | 0.69 (0.05-8.71)* | 0.62 (0.60-0.64) |
| Sudjai et al (2022), Thailand | maternal serum uric acid level ≥ 7.0 mg/dL | placental abruption | 400 | 2 (0.50) | 0.00 (0.00-0.66) | 0.69 (0.64-0.73) | 0.00 (0.00-0.03) | 0.99 (0.97-1.00) | 0.53 (0.04-6.74)* | 1.21 (0.73-2.02)* | 0.34 (0.32-0.37) |
| Thadhani et al (2022), US | sFlt-1/PlGF ratio ≥ 40 | placental abruption (within 2 weeks of enrolment) | 556 | 17 (3.10) | 0.71 (0.47-0.87) | 0.53 (0.48-0.57) | 0.04 (0.03-0.08) | 0.98 (0.96-0.99) | 1.49 (1.08-2.05) | 0.56 (0.27-1.17) | 0.62 (0.50-0.73) |
| Wei et al (2022), China | PNI ≤ 37 | placental abruption (premature separation of the placenta partially or completely before the delivery of the fetus) | 733 | 25 (3.41) | 0.64 (0.45-0.80) | 0.59 (0.55-0.63) | 0.05 (0.03-0.08) | 0.98 (0.96-0.99) | 1.56 (1.15-2.12) | 0.61 (0.36-1.03) | 0.62 (0.52-0.71) |
| Witlin et al (1999), US | MAP > 105 mm Hg | placental abruption | 445 | 32 (7.20) | 0.88 (0.70-0.96) | 0.02 (0.01-0.04) | NA | NA | 0.90 (0.80-1.00) | 5.70 (1.90-17.80) | NA |
| Witlin et al (1999), US | platelets < 60000/mm^3^ | placental abruption | 445 | 32 (7.20) | 0.38 (0.22-0.56) | 0.85 (0.81-0.88) | NA | NA | 2.50 (1.50-4.10) | 0.70 (0.60-1.00) | NA |
| Ye et al (2020), China | retinopathy staging group 3/4 | placental abruption | 534 | 25 (4.68) | 0.28 (0.14-0.48) | 0.86 (0.83-0.89) | 0.09 (0.04-0.17) | 0.96 (0.94-0.97) | 2.01 (1.03-3.90) | 0.84 (0.65-1.07) | 0.57 (0.48-0.66) |
| Yucesoy et al (2005), Turkey | platelets < 50000/mm^3^ | placental abruption | 44 | 5 (11.40) | 0.40 (0.07-0.83) | 0.64 (0.47-0.78) | NA | NA | 1.10 (0.40-3.50) | 0.90 (0.40-2.00) |  |
| Zheng et al (2022), China | multi-layer perceptron (with imputation) | placental abruption | 733 | 71 (9.69) | 0.06 (0.02-0.14) | 1.00 (1.00-1.00) | 1.00 (0.51-1.00) | 0.92 (0.90-0.94) | 99.12 (5.39-1822.83)* | 0.94 (0.88-1.00)* | 0.53 (0.50-0.56) |
| **Ascites** | | | | | | | | | | | |
| Hall et al (2002), South Africa | 24 h urine increased by ≥ 2g | ascites | 74 | 8 (10.80) | 0.62 (0.26-0.90) | 0.64 (0.51-0.75) | NA | NA | 1.70 (0.90-3.20) | 0.60 (0.20-1.50) | NA |
| Confidence intervals for the sensitivity, specificity, positive and negative predictive value were calculated using the Wilson's score method. Confidence intervals for the positive and negative likelihood ratios were calculated using Simel's method. Confidence intervals for AUROC were calculated using the method of DeLong. * 0.5 pseudo count added to each cell in the confusion matrix (true positives, false positives, true negatives, and false negatives) to avoid division by zero. **Abbreviations:** AUROC, area under the receiver operating characteristic curve; ALT, alanine transaminase; AST, aspartate transaminase; BMI, body mass index; CNS, central nervous system; CSC, central serous chorioretinopathy; DBP, diastolic blood pressure; DIC, disseminated intravascular coagulation; GlyFn, glycosylated fibronectin; HDU, high dependency unit; HELLP, hemolysis, elevated liver enzymes and low platelet count; hs-CRP, high-sensitive C-reactive protein; ICU, intensive care unit; IUGR, intrauterine growth restriction; LR-, negative likelihood ratio; LR+, positive likelihood ratio; MAP, mean arterial pressure; NPV, negative predictive value; PE, pre-eclampsia; PI, pulsatility index; PlGF, placental growth factor; PNI, prognostic nutritional index; PPH, postpartum haemorrhage; PPV, positive predictive value; PR, peak ratio; PRES, eclampsia or posterior reversible encephalopathy syndrome; SBP, systolic blood pressure; SD, standard deviation; sFlt-1, soluble fms-like tyrosine kinase-1; SGA, small for gestation age; UPCR, urinary protein to creatinine ratio; UtA, uterine artery. | | | | | | | | | | | |

**Supplementary table 8:** **Predictive performance of all prediction tests for composite maternal outcomes.**

|  | | | | | **2x2 Table** | | | |  | | | | | | |
| --- | --- | --- | --- | --- | --- | --- | --- | --- | --- | --- | --- | --- | --- | --- | --- |
| **Study** | **Predictor** | **Outcome** | **Sample size** | **Event rate n (%)** | **FP** | **TP** | **TN** | **FN** | **Sensitivity** | **Specificity** | **PPV** | **NPV** | **LR+** | **LR-** | **AUROC** |
| **maternal characteristics and preexisting disease** | | | | | | | | | | | | | | | |
| Nisly et al (2023), US | advanced maternal age | maternal death  sepsis  ICU admission  acute kidney injury (creatinine > 1.2 mg/dL)  postpartum dilation and curettage  postpartum hysterectomy  venous thromboembolism  PPH  postpartum wound complication (within 30 days of delivery)  postpartum endometriosis  pelvic abscess  postpartum pneumonia  readmission  blood transfusion | 260 | 77 (29.6) | 48 | 33 | 135 | 44 | NA | NA | NA | NA | NA | NA | NA |
| Nisly et al (2023), US | admission BMI > 39.9 | maternal death  sepsis  ICU admission  acute kidney injury (creatinine > 1.2 mg/dL)  postpartum dilation and curettage  postpartum hysterectomy  venous thromboembolism  PPH  postpartum wound complication (within 30 days of delivery)  postpartum endometriosis  pelvic abscess  postpartum pneumonia  readmission  blood transfusion | 260 | 77 (29.6) | 54 | 19 | 129 | 58 | NA | NA | NA | NA | NA | NA | NA |
| Li et al (2018), China | BMI | eclampsia placental abruption pulmonary oedema heart failure maternal death HELLP syndrome  severe hypoproteinemia (serum albumin < 25 g/L) | 1738 | 111 (6.39) | NA | NA | NA | NA | NA | NA | NA | NA | NA | NA | 0.550 (0.508–0.592) |
| Nisly et al (2023), US | multiple gestation | maternal death  sepsis  ICU admission  acute kidney injury (creatinine > 1.2 mg/dL)  postpartum dilation and curettage  postpartum hysterectomy  venous thromboembolism  PPH  postpartum wound complication (within 30 days of delivery)  postpartum endometriosis  pelvic abscess  postpartum pneumonia  readmission  blood transfusion | 260 | 77 (29.6) | 11 | 20 | 172 | 57 | NA | NA | NA | NA | NA | NA | NA |
| Nisly et al (2023), US | multiparous | maternal death  sepsis  ICU admission  acute kidney injury (creatinine > 1.2 mg/dL)  postpartum dilation and curettage  postpartum hysterectomy  venous thromboembolism  PPH  postpartum wound complication (within 30 days of delivery)  postpartum endometriosis  pelvic abscess  postpartum pneumonia  readmission  blood transfusion | 260 | 77 (29.6) | 81 | 39 | 102 | 38 | NA | NA | NA | NA | NA | NA | NA |
| Nisly et al (2023), US | private insurance | maternal death  sepsis  ICU admission  acute kidney injury (creatinine > 1.2 mg/dL)  postpartum dilation and curettage  postpartum hysterectomy  venous thromboembolism  PPH  postpartum wound complication (within 30 days of delivery)  postpartum endometriosis  pelvic abscess  postpartum pneumonia  readmission  blood transfusion | 260 | 77 (29.6) | 96 | 46 | 87 | 31 | NA | NA | NA | NA | NA | NA | NA |
| Nisly et al (2023), US | tobacco use in pregnancy | maternal death  sepsis  ICU admission  acute kidney injury (creatinine > 1.2 mg/dL)  postpartum dilation and curettage  postpartum hysterectomy  venous thromboembolism  PPH  postpartum wound complication (within 30 days of delivery)  postpartum endometriosis  pelvic abscess  postpartum pneumonia  readmission  blood transfusion | 260 | 77 (29.6) | 16 | 11 | 167 | 66 | NA | NA | NA | NA | NA | NA | NA |
| Nisly et al (2023), US | marijuana use in pregnancy | maternal death  sepsis  ICU admission  acute kidney injury (creatinine > 1.2 mg/dL)  postpartum dilation and curettage  postpartum hysterectomy  venous thromboembolism  PPH  postpartum wound complication (within 30 days of delivery)  postpartum endometriosis  pelvic abscess  postpartum pneumonia  readmission  blood transfusion | 260 | 77 (29.6) | 11 | 2 | 172 | 75 | NA | NA | NA | NA | NA | NA | NA |
| Li et al (2018), China | gestational age < 34 weeks | eclampsia placental abruption pulmonary oedema heart failure maternal death HELLP syndrome  severe hypoproteinemia (serum albumin < 25 g/L) | 1738 | 111 (6.39) | NA | NA | NA | NA | NA | NA | NA | NA | NA | NA | 0.685 (0.649–0.721) |
| Nisly et al (2023), US | pre-eclampsia before 28 weeks gestational age | maternal death  sepsis  ICU admission  acute kidney injury (creatinine > 1.2 mg/dL)  postpartum dilation and curettage  postpartum hysterectomy  venous thromboembolism  PPH  postpartum wound complication (within 30 days of delivery)  postpartum endometriosis  pelvic abscess  postpartum pneumonia  readmission  blood transfusion | 260 | 77 (29.6) | 51 | 27 | 132 | 50 | NA | NA | NA | NA | NA | NA | NA |
| Nisly et al (2023), US | delivery before 28 weeks gestational age | maternal death  sepsis  ICU admission  acute kidney injury (creatinine > 1.2 mg/dL)  postpartum dilation and curettage  postpartum hysterectomy  venous thromboembolism  PPH  postpartum wound complication (within 30 days of delivery)  postpartum endometriosis  pelvic abscess  postpartum pneumonia  readmission  blood transfusion | 260 | 77 (29.6) | 35 | 23 | 148 | 54 | NA | NA | NA | NA | NA | NA | NA |
| Li et al (2018), China | referral | eclampsia placental abruption pulmonary oedema heart failure maternal death HELLP syndrome  severe hypoproteinemia (serum albumin < 25 g/L) | 1738 | 111 (6.39) | NA | NA | NA | NA | NA | NA | NA | NA | NA | NA | 0.591 (0.550–0.632) |
| Fishel Bartal et al (2022), US | Ethnicity: Non–Hispanic White | other hypertensive disorders (gestational hypertension and preeclampsia) primary cesarean delivery ICU admission blood transfusion uterine rupture unplanned hysterectomy | 389069 | 112324 (28.87) | 138077 | 57222 | 138594 | 55176 | NA | NA | NA | NA | NA | NA | NA |
| Fishel Bartal et al (2022), US | Ethnicity: Non–Hispanic Black | other hypertensive disorders (gestational hypertension and preeclampsia) primary cesarean delivery ICU admission blood transfusion uterine rupture unplanned hysterectomy | 389069 | 112324 (28.87) | 81784 | 33634 | 194887 | 78764 | NA | NA | NA | NA | NA | NA | NA |
| Fishel Bartal et al (2022), US | Ethnicity: Hispanic | other hypertensive disorders (gestational hypertension and preeclampsia) primary cesarean delivery ICU admission blood transfusion uterine rupture unplanned hysterectomy | 389069 | 112324 (28.87) | 44036 | 16230 | 232635 | 96168 | NA | NA | NA | NA | NA | NA | NA |
| Fishel Bartal et al (2022), US | Ethnicity: Non–Hispanic Asian American, Native Hawaiian and other Pacific Islander | other hypertensive disorders (gestational hypertension and preeclampsia) primary cesarean delivery ICU admission blood transfusion uterine rupture unplanned hysterectomy | 389069 | 112324 (28.87) | 9650 | 4214 | 267021 | 108184 | NA | NA | NA | NA | NA | NA | NA |
| Fishel Bartal et al (2022), US | Ethnicity: Non–Hispanic American Indian and Alaska Native | other hypertensive disorders (gestational hypertension and preeclampsia) primary cesarean delivery ICU admission blood transfusion uterine rupture unplanned hysterectomy | 389069 | 112324 (28.87) | 3124 | 1098 | 273547 | 111300 | NA | NA | NA | NA | NA | NA | NA |
| Nisly et al (2023), US | Ethnicity: White | maternal death  sepsis  ICU admission  acute kidney injury (creatinine > 1.2 mg/dL)  postpartum dilation and curettage  postpartum hysterectomy  venous thromboembolism  PPH  postpartum wound complication (within 30 days of delivery)  postpartum endometriosis  pelvic abscess  postpartum pneumonia  readmission  blood transfusion | 260 | 77 (29.6) | 67 | 27 | 116 | 50 | NA | NA | NA | NA | NA | NA | NA |
| Nisly et al (2023), US | Ethnicity: Black | maternal death  sepsis  ICU admission  acute kidney injury (creatinine > 1.2 mg/dL)  postpartum dilation and curettage  postpartum hysterectomy  venous thromboembolism  PPH  postpartum wound complication (within 30 days of delivery)  postpartum endometriosis  pelvic abscess  postpartum pneumonia  readmission  blood transfusion | 260 | 77 (29.6) | 92 | 41 | 91 | 36 | NA | NA | NA | NA | NA | NA | NA |
| Nisly et al (2023), US | Ethnicity: Asian | maternal death  sepsis  ICU admission  acute kidney injury (creatinine > 1.2 mg/dL)  postpartum dilation and curettage  postpartum hysterectomy  venous thromboembolism  PPH  postpartum wound complication (within 30 days of delivery)  postpartum endometriosis  pelvic abscess  postpartum pneumonia  readmission  blood transfusion | 260 | 77 (29.6) | 8 | 1 | 175 | 76 | NA | NA | NA | NA | NA | NA | NA |
| Nisly et al (2023), US | Ethnicity: other than White, Black or Asian | maternal death  sepsis  ICU admission  acute kidney injury (creatinine > 1.2 mg/dL)  postpartum dilation and curettage  postpartum hysterectomy  venous thromboembolism  PPH  postpartum wound complication (within 30 days of delivery)  postpartum endometriosis  pelvic abscess  postpartum pneumonia  readmission  blood transfusion | 260 | 77 (29.6) | 2 | 2 | 181 | 75 | NA | NA | NA | NA | NA | NA | NA |
| Nisly et al (2023), US | Ethnicity: Hispanic/Latin | maternal death  sepsis  ICU admission  acute kidney injury (creatinine > 1.2 mg/dL)  postpartum dilation and curettage  postpartum hysterectomy  venous thromboembolism  PPH  postpartum wound complication (within 30 days of delivery)  postpartum endometriosis  pelvic abscess  postpartum pneumonia  readmission  blood transfusion | 260 | 77 (29.6) | 14 | 6 | 169 | 71 | NA | NA | NA | NA | NA | NA | NA |
| Nisly et al (2023), US | preexisting IUGR on admission | maternal death  sepsis  ICU admission  acute kidney injury (creatinine > 1.2 mg/dL)  postpartum dilation and curettage  postpartum hysterectomy  venous thromboembolism  PPH  postpartum wound complication (within 30 days of delivery)  postpartum endometriosis  pelvic abscess  postpartum pneumonia  readmission  blood transfusion | 260 | 77 (29.6) | 64 | 26 | 119 | 51 | NA | NA | NA | NA | NA | NA | NA |
| Nisly et al (2023), US | latency ≥ 7 days | maternal death  sepsis  ICU admission  acute kidney injury (creatinine > 1.2 mg/dL)  postpartum dilation and curettage  postpartum hysterectomy  venous thromboembolism  PPH  postpartum wound complication (within 30 days of delivery)  postpartum endometriosis  pelvic abscess  postpartum pneumonia  readmission  blood transfusion | 260 | 77 (29.6) | 54 | 26 | 129 | 51 | NA | NA | NA | NA | NA | NA | NA |
| Nisly et al (2023), US | vaginal delivery candidate who attempted labor | maternal death  sepsis  ICU admission  acute kidney injury (creatinine > 1.2 mg/dL)  postpartum dilation and curettage  postpartum hysterectomy  venous thromboembolism  PPH  postpartum wound complication (within 30 days of delivery)  postpartum endometriosis  pelvic abscess  postpartum pneumonia  readmission  blood transfusion | 260 | 77 (29.6) | 95 | 25 | 48 | 27 | NA | NA | NA | NA | NA | NA | NA |
| Nisly et al (2023), US | anemia at delivery | maternal death  sepsis  ICU admission  acute kidney injury (creatinine > 1.2 mg/dL)  postpartum dilation and curettage  postpartum hysterectomy  venous thromboembolism  PPH  postpartum wound complication (within 30 days of delivery)  postpartum endometriosis  pelvic abscess  postpartum pneumonia  readmission  blood transfusion | 260 | 77 (29.6) | 49 | 21 | 134 | 56 | NA | NA | NA | NA | NA | NA | NA |
| Nisly et al (2023), US | cesarean at delivery | maternal death  sepsis  ICU admission  acute kidney injury (creatinine > 1.2 mg/dL)  postpartum dilation and curettage  postpartum hysterectomy  venous thromboembolism  PPH  postpartum wound complication (within 30 days of delivery)  postpartum endometriosis  pelvic abscess  postpartum pneumonia  readmission  blood transfusion | 260 | 77 (29.6) | 142 | 65 | 41 | 12 | NA | NA | NA | NA | NA | NA | NA |
| Nisly et al (2023), US | prior cesarean delivery | maternal death  sepsis  ICU admission  acute kidney injury (creatinine > 1.2 mg/dL)  postpartum dilation and curettage  postpartum hysterectomy  venous thromboembolism  PPH  postpartum wound complication (within 30 days of delivery)  postpartum endometriosis  pelvic abscess  postpartum pneumonia  readmission  blood transfusion | 260 | 77 (29.6) | 17 | 14 | 166 | 63 | NA | NA | NA | NA | NA | NA | NA |
| Malhamé et al (2022_1), US | obstructive sleep apnea | ICU admission acute renal failure pulmonary oedema pulmonary embolism congestive heart failure cardiomyopathy stroke death | 71159 | 8368 (11.76) | 201 | 69 | 62587 | 8302 | NA | NA | NA | NA | NA | NA | NA |
| Malhamé et al (2022_1), US | obstructive sleep apnea | congestive heart failure cardiomyopathy pulmonary oedema stroke | 71159 | 398 (0.56) | 262 | 8 | 70498 | 391 | NA | NA | NA | NA | NA | NA | NA |
| Nisly et al (2023), US | depression | maternal death  sepsis  ICU admission  acute kidney injury (creatinine > 1.2 mg/dL)  postpartum dilation and curettage  postpartum hysterectomy  venous thromboembolism  PPH  postpartum wound complication (within 30 days of delivery)  postpartum endometriosis  pelvic abscess  postpartum pneumonia  readmission  blood transfusion | 260 | 77 (29.6) | 30 | 18 | 153 | 59 | NA | NA | NA | NA | NA | NA | NA |
| Nisly et al (2023), US | chronic hypertension | maternal death  sepsis  ICU admission  acute kidney injury (creatinine > 1.2 mg/dL)  postpartum dilation and curettage  postpartum hysterectomy  venous thromboembolism  PPH  postpartum wound complication (within 30 days of delivery)  postpartum endometriosis  pelvic abscess  postpartum pneumonia  readmission  blood transfusion | 260 | 77 (29.6) | 58 | 33 | 125 | 44 | NA | NA | NA | NA | NA | NA | NA |
| Nisly et al (2023), US | preexisting diabetes | maternal death  sepsis  ICU admission  acute kidney injury (creatinine > 1.2 mg/dL)  postpartum dilation and curettage  postpartum hysterectomy  venous thromboembolism  PPH  postpartum wound complication (within 30 days of delivery)  postpartum endometriosis  pelvic abscess  postpartum pneumonia  readmission  blood transfusion | 260 | 77 (29.6) | 14 | 13 | 169 | 64 | NA | NA | NA | NA | NA | NA | NA |
| Nisly et al (2023), US | chorioamnionitis | maternal death  sepsis  ICU admission  acute kidney injury (creatinine > 1.2 mg/dL)  postpartum dilation and curettage  postpartum hysterectomy  venous thromboembolism  PPH  postpartum wound complication (within 30 days of delivery)  postpartum endometriosis  pelvic abscess  postpartum pneumonia  readmission  blood transfusion | 260 | 77 (29.6) | 2 | 1 | 181 | 76 | NA | NA | NA | NA | NA | NA | NA |
| **blood pressure measurement** | | | | | | | | | | | | | | | |
| Li et al (2018), China | severe hypertension (SBP ≥ 160 mmHg, DBP ≥ 110 mmHg) | eclampsia placental abruption pulmonary oedema heart failure maternal death HELLP syndrome  severe hypoproteinemia (serum albumin < 25 g/L) | 1738 | 111 (6.39) | NA | NA | NA | NA | NA | NA | NA | NA | NA | NA | 0.604 (0.566–0.642) |
| Kumar et al (2023), India | MAP ≥ 127.85 mmHg | intravenous antihypertensive agents  ICU admission  eclampsia  placental abruption  HELLP syndrome  DIC  platelets <100x10^9/L,  creatinine >1.1 mg/dL  ALT >100 U/L  stroke  cortical blindness  acute pulmonary oedema  liver capsule rupture or hematoma  maternal death | 91 | 44 (48.9) | NA | NA | NA | NA | 0.06 | 0.70 | NA | NA | NA | NA | NA |
| Simón et al (2020), Spain | MAP ≥ 122.7 mmHg | HELLP neurological events (eclampsia, stroke, cortical blindness, retinal detachment, PRES) acute myocardial infarction hepatic subcapsular hematoma pulmonary oedema acute renal failure (serum creatinine > 1.2 mg/dL or need for dialysis)  placental abruption maternal death | 76 | 25 (32.89) | NA | NA | NA | NA | 0.53 (0.31–0.72) | 0.9 | 0.72 (0.51–0.87) | 0.79 (0.72–0.85) | 5.3 (2.1–13.2) |  | 0.42 (0.26–0.59) |
| **proteinuria** | | | | | | | | | | | | | | | |
| Lei et al (2021), China | 24 h Proteinuria excretion < 0.3 g/24h | organ dysfunction (kidney, liver or heart failure) eclampsia HELLP hypoproteinemia pleural or peritoneal effusion pericardial effusion oligohydramnios placental abruption death retinal disease DIC PPH | 275 | 92 (33.5) | 42 | 11 | 141 | 81 | NA | NA | NA | NA | NA | NA | NA |
| Lei et al (2021), China | 24 h Proteinuria excretion 0.3-2.0 g/24h | organ dysfunction (kidney, liver or heart failure) eclampsia HELLP hypoproteinemia pleural or peritoneal effusion pericardial effusion oligohydramnios placental abruption death retinal disease DIC PPH | 275 | 92 (33.5) | 43 | 21 | 140 | 71 | NA | NA | NA | NA | NA | NA | NA |
| Lei et al (2021), China | 24 h Proteinuria excretion ≥ 2.0 g/24h | organ dysfunction (kidney, liver or heart failure) eclampsia HELLP hypoproteinemia pleural or peritoneal effusion pericardial effusion oligohydramnios placental abruption death retinal disease DIC PPH | 275 | 92 (33.5) | 27 | 26 | 156 | 66 | NA | NA | NA | NA | NA | NA | NA |
| Lei et al (2021), China | 24 h Proteinuria excretion < 0.3 g/24h | organ dysfunction (kidney, liver or heart failure) | 275 | 20 (7.3) | 50 | 3 | 205 | 17 | NA | NA | NA | NA | NA | NA | NA |
| Lei et al (2021), China | 24 h Proteinuria excretion 0.3-2.0 g/24h | organ dysfunction (kidney, liver or heart failure) | 275 | 20 (7.3) | 64 | 0 | 191 | 20 | NA | NA | NA | NA | NA | NA | NA |
| Lei et al (2021), China | 24 h Proteinuria excretion ≥ 2.0 g/24h | organ dysfunction (kidney, liver or heart failure) | 275 | 20 (7.3) | 46 | 7 | 209 | 13 | NA | NA | NA | NA | NA | NA | NA |
| Li et al (2018), China | 24 h Proteinuria excretion ≥ 0.3-< 2.0 g/24h | eclampsia placental abruption pulmonary oedema heart failure maternal death HELLP syndrome  severe hypoproteinemia (serum albumin < 25 g/L) | 1738 | 111 (6.39) | 609 | 29 | 1018 | 82 | NA | NA | NA | NA | NA | NA | NA |
| Li et al (2018), China | 24 h Proteinuria excretion ≥ 2.0-< 5.0 g/24h | eclampsia placental abruption pulmonary oedema heart failure maternal death HELLP syndrome  severe hypoproteinemia (serum albumin < 25 g/L) | 1738 | 111 (6.39) | 327 | 26 | 1300 | 85 | NA | NA | NA | NA | NA | NA | NA |
| Li et al (2018), China | 24 h Proteinuria excretion ≥ 5.0 g/24h | eclampsia placental abruption pulmonary oedema heart failure maternal death HELLP syndrome  severe hypoproteinemia (serum albumin < 25 g/L) | 1738 | 111 (6.39) | 376 | 43 | 1251 | 68 | NA | NA | NA | NA | NA | NA | NA |
| Li et al (2018), China | 24 h Proteinuria excretion ≥ 2.0 g/24h | eclampsia placental abruption pulmonary oedema heart failure maternal death HELLP syndrome  severe hypoproteinemia (serum albumin < 25 g/L) | 1738 | 111 (6.39) | NA | NA | NA | NA | NA | NA | NA | NA | NA | NA | 0.668 (0.632–0.705) |
| Murali et al (2023), India | 24h Proteinuria excretion ≥ 2490 mg/24h | ICU admission  pulmonary oedema  eclampsia  maternal death | 202 | 70 (34.65) | NA | NA | NA | NA | 0.61 | 0.44 | NA | NA | NA | NA | 0.484 (0.40-0.57) |
| Chadha et al (2022), India | UPCR ≥0.3 | severe hypertension (≥160/110mmHg) renal insufficiency (serum creatinine >1.2mg/dL) or oliguria (<400mg/dL) Increased liver enzyme (AST >40u/l) thrombocytopenia (platelet count <150x10^9/l) HELLP placental abruption eclampsia ICU admission  neurological involvement  maternal death | 141 | NA | NA | NA | NA | NA | 0.79 (0.71–0.86) | 0.47 (0.21–0.73) | 0.93 (0.89–0.95) | 0.21(0.12–0.34) | NA | NA | 0.848 (0.878–0.910) |
| Murali et al (2023), India | 24h Proteinuria excretion ≥ 2490 mg/24h | ICU admission  pulmonary oedema  eclampsia  maternal death | 202 | 70 (34.65) | NA | NA | NA | NA | 0.6 | 0.4 | NA | NA | NA | NA | 0.495 (0.4-0.58) |
| Simón et al (2020), Spain | UPCR ≥ 4.8 mg/mg | HELLP neurological events (eclampsia, stroke, cortical blindness, retinal detachment, posterior reversible encephalopathy syndrome) acute myocardial infarction hepatic subcapsular hematoma pulmonary oedema acute renal failure (serum creatinine > 1.2 mg/dL or need for dialysis)  placental abruption maternal death | 76 | 25 (32.89) | NA | NA | NA | NA | 0.11 (0.03–0.31) | 0.9 | 0.38 (0.14–0.70) | 0.68 (0.64–0.71) | 1.2 (0.3–4.7) | NA | 0.49 (0.32–0.65) |
| Gangaram et al (2009), South Africa | spot urine ACR ≥ 300 mg/g | placental abruption eclampsia  intensive care admission | 155 | 4 (2.60) | NA | NA | NA | NA | 0.00 | 0.55 (0.47-0.63) | NA | NA | NA | 1.80 (1.80-1.80) | NA |
| **angiogenic biomarkers** | | | | | | | | | | | | | | | |
| Karge et al (2022), Germany | serum PlGF | eclampsia pulmonary oedema acute kidney injury (serum creatinine greater than 1.1 mg/dL or a doubling of serum creatinine in the absence of other renal disease) placental abruption HELLP  ICU admission intubation mechanical ventilation | 141 | 36 (25.53) | NA | NA | NA | NA | NA | NA | NA | NA | NA | NA | 0.605 (0.495–0.715) |
| Kumar et al (2023), India | serum PlGF ≥ 1680.0 pg/mL | intravenous antihypertensive agents  ICU admission  eclampsia  placental abruption  HELLP syndrome  DIC  platelets <100x10^9/L,  creatinine >1.1 mg/dL  ALT >100 U/L  stroke  cortical blindness  acute pulmonary oedema  liver capsule rupture or hematoma  maternal death | 91 | 44 (48.9) | NA | NA | NA | NA | 0 | 0.98 | NA | NA | NA | NA | 0.25 (0.15-0.35) |
| Reddy et al (2022), Australia | serum PlGF | need for parenteral antihypertensive agents ICU admission  eclampsia placental abruption HELLP DIC thrombocytopenia (platelets <100×10^9/L) creatinine >90μmol/L ALT >100 U/L | 126 | 49 (38.9) | NA | NA | NA | NA | 0.06 (0.0–0.43) | NA | NA | NA | NA | NA | 0.65 (0.55–0.75) |
| Reddy et al (2022), Australia | serum PlGF | ICU admission HELLP placental abruption eclampsia | 126 | 10 (7.94) | NA | NA | NA | NA | 0.11 (0.00–0.44) | NA | NA | NA | NA | NA | 0.64 (0.48–0.79) |
| Simón et al (2020), Spain | serum PlGF ≤ 16.5 ng/mL | HELLP neurological events (eclampsia, stroke, cortical blindness, retinal detachment, posterior reversible encephalopathy syndrome) acute myocardial infarction hepatic subcapsular hematoma pulmonary oedema acute renal failure (serum creatinine > 1.2 mg/dL or need for dialysis)  placental abruption maternal death | 76 | 25 (32.89) | NA | NA | NA | NA | 0.28 (0.97–0.54) | 90 | 0.45 (0.22–0.71) | 0.79 (0.74–0.84) | 2.6 (0.9–7.5) | NA | 0.62 (0.45–0.78) |
| Karge et al (2022), Germany | serum sFlt-1 | eclampsia pulmonary oedema acute kidney injury (serum creatinine greater than 1.1 mg/dL or a doubling of serum creatinine in the absence of other renal disease) placental abruption HELLP  ICU admission intubation mechanical ventilation | 141 | 36 (25.53) | NA | NA | NA | NA | NA | NA | NA | NA | NA | NA | 0.694 (0.598–0.790) |
| Kumar et al (2023), India | serum sFlt-1 ≥ 8169.5 pg/mL | intravenous antihypertensive agents  ICU admission  eclampsia  placental abruption  HELLP syndrome  DIC  platelets <100x10^9/L,  creatinine >1.1 mg/dL  ALT >100 U/L  stroke  cortical blindness  acute pulmonary oedema  liver capsule rupture or hematoma  maternal death | 91 | 44 (48.9) | NA | NA | NA | NA | 0.66 | 0.83 | NA | NA | NA | NA | 0.79 (0.70-0.88) |
| Reddy et al (2022), Australia | serum sFlt-1 | need for parenteral antihypertensive agents ICU admission  eclampsia placental abruption HELLP DIC thrombocytopenia (platelets <100×10^9/L) creatinine >90μmol/L ALT >100 U/L | 126 | 49 (38.9) | NA | NA | NA | NA | 0.18 (0.04–0.33) | NA | NA | NA | NA | NA | 0.69 (0.60–0.79) |
| Reddy et al (2022), Australia | serum sFlt-1 | ICU admission HELLP placental abruption eclampsia | 126 | 10 (7.94) | NA | NA | NA | NA | 0.00 (0.00–0.33) | NA | NA | NA | NA | NA | 0.60 (0.46–0.75) |
| Binder et al (2023), Austria | sFlt-1/PlGF ratio abnormal | ICU admission  pulmonary oedema  eclampsia  maternal death | 323 | 37 (11.5) | 222 | 34 | 64 | 3 | NA | NA | NA | NA | NA | NA | NA |
| Chaiworapongsa et al (2023), US | abnormal angiogenic profile (sFlt-1/PlGF ratio as a multiple of the median (MoM) < 10th percentile for gestational age in early onset pre-eclampsia (cohort 1) | eclampsia  blindness  stroke  myocardial ischemia  pulmonary oedema  elevated liver enzymes (ALT or AST ≥ 70 IU/L)  hepatic hematoma  thrombocytopenia (platelet count <100x10^9/L)  acute kidney injury (serum creatinine >1.1 mg/dL)  placental abruption  DIC  maternal death | 29 | 13 (44.83) | 15 | 11 | 1 | 2 | NA | NA | NA | NA | NA | NA | NA |
| Chaiworapongsa et al (2023), US | abnormal angiogenic profile (sFlt-1/PlGF ratio as a multiple of the median (MoM) < 10th percentile for gestational age in intermediate pre-eclampsia (cohort 1) | eclampsia  blindness  stroke  myocardial ischemia  pulmonary oedema  elevated liver enzymes (ALT or AST ≥ 70 IU/L)  hepatic hematoma  thrombocytopenia (platelet count <100x10^9/L)  acute kidney injury (serum creatinine >1.1 mg/dL)  placental abruption  DIC  maternal death | 16 | 1 (6.25) | 15 | 1 | 0 | 0 | NA | NA | NA | NA | NA | NA | NA |
| Chaiworapongsa et al (2023), US | abnormal angiogenic profile (sFlt-1/PlGF ratio as a multiple of the median (MoM) < 10th percentile for gestational age in term pre-eclampsia (cohort 1) | eclampsia  blindness  stroke  myocardial ischemia  pulmonary oedema  elevated liver enzymes (ALT or AST ≥ 70 IU/L)  hepatic hematoma  thrombocytopenia (platelet count <100x10^9/L)  acute kidney injury (serum creatinine >1.1 mg/dL)  placental abruption  DIC  maternal death | 106 | 6 (5.66) | 37 | 4 | 63 | 2 | NA | NA | NA | NA | NA | NA | NA |
| Chaiworapongsa et al (2023), US | abnormal angiogenic profile (sFlt-1/PlGF ratio as a multiple of the median (MoM) < 10th percentile for gestational age in early onset pre-eclampsia (cohort 2) | eclampsia  blindness  stroke  myocardial ischemia  pulmonary oedema  elevated liver enzymes (ALT or AST ≥ 70 IU/L)  hepatic hematoma  thrombocytopenia (platelet count <100x10^9/L)  acute kidney injury (serum creatinine >1.1 mg/dL)  placental abruption  DIC  maternal death | 89 | 33 (37.08) | 54 | 33 | 2 | 0 | NA | NA | NA | NA | NA | NA | NA |
| Chaiworapongsa et al (2023), US | abnormal angiogenic profile (sFlt-1/PlGF ratio as a multiple of the median (MoM) < 10th percentile for gestational age in intermediate pre-eclampsia (cohort 2) | eclampsia  blindness  stroke  myocardial ischemia  pulmonary oedema  elevated liver enzymes (ALT or AST ≥ 70 IU/L)  hepatic hematoma  thrombocytopenia (platelet count <100x10^9/L)  acute kidney injury (serum creatinine >1.1 mg/dL)  placental abruption  DIC  maternal death | 105 | 20 (19.05) | 68 | 16 | 17 | 4 | NA | NA | NA | NA | NA | NA | NA |
| Chaiworapongsa et al (2023), US | abnormal angiogenic profile (sFlt-1/PlGF ratio as a multiple of the median (MoM) < 10th percentile for gestational age in term pre-eclampsia (cohort 2) | eclampsia  blindness  stroke  myocardial ischemia  pulmonary oedema  elevated liver enzymes (ALT or AST ≥ 70 IU/L)  hepatic hematoma  thrombocytopenia (platelet count <100x10^9/L)  acute kidney injury (serum creatinine >1.1 mg/dL)  placental abruption  DIC  maternal death | 258 | 25 (9.69) | 122 | 19 | 111 | 6 | NA | NA | NA | NA | NA | NA | NA |
| Karge et al (2022), Germany | sFlt-1/PlGF ratio | eclampsia pulmonary oedema acute kidney injury (serum creatinine greater than 1.1 mg/dL or a doubling of serum creatinine in the absence of other renal disease) placental abruption HELLP  ICU admission intubation mechanical ventilation | 141 | 36 (25.53) | NA | NA | NA | NA | NA | NA | NA | NA | NA | NA | 0.667 (0.566–0.768) |
| Kumar et al (2023), India | sFlt-1/PlGF ratio ≥ 71.92 | intravenous antihypertensive agents  ICU admission  eclampsia  placental abruption  HELLP syndrome  DIC  platelets <100x10^9/L,  creatinine >1.1 mg/dL  ALT >100 U/L  stroke  cortical blindness  acute pulmonary oedema  liver capsule rupture or hematoma  maternal death | 91 | 44 (48.9) | NA | NA | NA | NA | 0.84 | 0.74 | NA | NA | NA | NA | 0.81 (0.72-0.90) |
| Leaños–Miranda et al (2013), Mexico | serum sFlt-1/PlGF ratio ≥ 871 | hepatic hematoma or rupture (confirmed by ultrasound or laparotomy)  pulmonary oedema (clinical diagnosis and with radiographic confirmation)  need for positive inotropic support, intubation (other than solely for caesarean section) acute renal failure (creatinine ≥198 µmol/L)  placental abruption (clinical or pathological). | 501 | 48 (9.60) | NA | NA | NA | NA | 0.52 (0.37-0.67) | 0.78 (0.74-0.82) | NA | NA | 2.36 (1.1-3.26) | 0.61 (0.46-0.83) | NA |
| Leanos–Miranda et al (2020), Mexico | sFlt-1/PlGF ratio > 38-< 85 | thrombocytopenia (Platelet count <100x10^3 μl) abnormal liver enzymes levels (to twice normal concentration) hepatic hematoma or rupture  pulmonary oedema  need for positive inotropic support  Intubation  acute kidney injury (creatinine >1.5 mg/dL) placental abruption  cerebral haemorrhage maternal death | 810 | 198 (24.44) | 102 | 0 | 510 | 198 | NA | NA | NA | NA | NA | NA | NA |
| Leanos–Miranda et al (2020), Mexico | sFlt-1/PlGF ratio ≥ 85 | thrombocytopenia (Platelet count <100x10^3 μl) abnormal liver enzymes levels (to twice normal concentration) hepatic hematoma or rupture  pulmonary oedema  need for positive inotropic support  Intubation  acute kidney injury (creatinine >1.5 mg/dL) placental abruption  cerebral haemorrhage maternal death | 810 | 198 (24.44) | 226 | 198 | 386 | 0 | NA | NA | NA | NA | NA | NA | NA |
| Mirkovic et al (2020), Serbia | sFlt-1/PlGF ratio ≥275 | maternal mortality or ≥1 serious CNS, cardiorespiratory, renal, hepatic or haematological morbidity | 61 | 48 (78.69) | NA | NA | NA | NA | 0.85 | 0.62 | NA | NA | NA | NA | NA |
| Mirkovic et al (2020), Serbia | sFlt-1/PlGF ratio ≥ 377 | maternal mortality or ≥ 1 serious CNS, cardiorespiratory, renal, hepatic or haematological morbidity | 61 | 48 (78.69) | NA | NA | NA | NA | 0.75 | 0.92 | NA | NA | NA | NA | NA |
| Mirkovic et al (2020), Serbia | sFlt-1/PlGF ratio ≥ 695 | maternal mortality or ≥1 serious CNS, cardiorespiratory, renal, hepatic or haematological morbidity | 61 | 48 (78.69) | NA | NA | NA | NA | 0.412 | 0.92 | NA | NA | NA | NA | NA |
| Mirkovic et al (2020), Serbia | sFlt-1/PlGF ratio | maternal mortality or ≥1 serious CNS, cardiorespiratory, renal, hepatic or haematological morbidity | 61 | 48 (78.69) | NA | NA | NA | NA | NA | NA | NA | NA | NA | NA | 0.853 (0.733–0.972) |
| Palomaki et al (2015), US | sFlt-1/PlGF ratio ≥ 85 | severe hypertension (BP ≥ 160/110)  elevated liver function test(s)  DIC  placental abruption  pulmonary oedema  cerebral haemorrhage  maternal death  eclampsia  acute renal failure  HELLP syndrome | 237 | 21 (8.90) | NA | NA | NA | NA | 0.62 (0.39-0.81) | 0.69 (0.63-0.75) | NA | NA | 2.20 (1.40-3.00) | 0.50 (0.30-1.00) | NA |
| Rana et al (2013), US | sFlt-1/PlGF ratio ≥ 85 | hypertension (blood pressure ≥140/90 mm Hg  plus one of the following:  (AST or ALT, ≥ 80 U/L)  platelet count 100x10^9/L  DIC  abruption (clinical and/or pathological)  pulmonary oedema  cerebral haemorrhage  seizure  acute renal failure (creatinine ≥114.4 µmol/L)  maternal death | 97 | 8 (8.20) | NA | NA | NA | NA | 1.00 (0.60-1.00) | 0.52 (0.41-0.62) | NA | NA | 2.10 (1.70-2.60) | ∞ | NA |
| Reddy et al (2022), Australia | sFlt-1/PlGF ratio | need for parenteral antihypertensive agents ICU admission  eclampsia placental abruption HELLP DIC thrombocytopenia (platelets <100×10^9/L) creatinine >90μmol/L ALT >100 U/L | 126 | 49 (38.9) | NA | NA | NA | NA | 0.14 (0.02–0.41) | NA | NA | NA | NA | NA | 0.69 (0.59–0.78) |
| Reddy et al (2022), Australia | sFlt-1/PlGF ratio | ICU admission HELLP placental abruption eclampsia | 126 | 10 (7.94) | NA | NA | NA | NA | 0.11 (0.00–0.33) | NA | NA | NA | NA | NA | 0.62 (0.46–0.78) |
| Saleh et al (2016), the Netherlands | sFlt-1/PlGF ratio > 85 | PIERS composite outcomes  minus transfusion of blood products | 62 | 6 (9.70) | NA | NA | NA | NA | 1.00 (0.52-1.00) | 0.11 (0.04-0.22) | NA | NA | 1.10 (1.00-1.20) | NA | NA |
| Simón et al (2020), Spain | sFlt-1/PlGF ratio ≥ 794 | HELLP neurological events (eclampsia, stroke, cortical blindness, retinal detachment, posterior reversible encephalopathy syndrome) acute myocardial infarction hepatic subcapsular hematoma pulmonary oedema acute renal failure (serum creatinine > 1.2 mg/dL or need for dialysis)  placental abruption maternal death | 76 | 25 (32.89) | NA | NA | NA | NA | 0.26 (0.12–0.49) | 90 | 0.58 (0.33–0.80) | 0.72 (0.66–0.77) | 2.9 (1.0–8.1) | NA | 0.59 (0.42–0.75) |
| Suresh et al (2022), US | sFlt-1/PlGF ratio ≥ 38 | HELLP eclampsia cerebral haemorrhage pulmonary oedema acute kidney injury DIC placental abruption death | 269 | NA | NA | NA | NA | NA | 0.86 | 0.5 | 0.55 | 0.83 | 1.72 | 0.28 | 0.68 (0.63– 0.73) |
| Suresh et al (2022), US | sFlt-1/PlGF ratio ≥ 85 | HELLP eclampsia cerebral haemorrhage pulmonary oedema acute kidney injury DIC placental abruption death | 269 | NA | NA | NA | NA | NA | 0.66 | 0.76 | 0.67 | 0.76 | 2.75 | 0.45 | 0.71 (0.66– 0.77) |
| Thadhani et al (2022), US | sFlt-1/PlGF ratio ≥ 40 | severe hypertension (≥ 160 mmHg or ≥ 110 mmHg)  placental abruption cerebral haemorrhage seizure without underlying seizure disorder pulmonary oedema DIC AST or ALT levels > 80 U/l) renal insufficiency (serum creatinine > 1.4 mg/dl) thrombocytopenia (platelet count <100,000/µl) | 556 | 51 (9.2) | 224 | 43 | 281 | 8 | NA | NA | NA | NA | NA | NA | NA |
| **Doppler assessment** | | | | | | | | | | | | | | | |
| Loardi et al (2021), Italy | UtA PI > 95th percentile | HELLP  acute kidney failure pulmonary oedema neurologic complications DIC placental abruption | 311 | 41 (13.2) | NA | NA | NA | NA | 0.76 (0.60–0.88) | 0.42 (0.36–0.48) | 0.17 (0.11–0.23) | 0.92 (0.86–0.96) | 1.31 (1.07–1.60) | 0.58 (0.331–1.01) | NA |
| Reddy et al (2022), Australia | UtA PI | ICU admission HELLP placental abruption eclampsia | 126 | 10 (7.94) | NA | NA | NA | NA | 0.33 (0.00–0.67) | NA | NA | NA | NA | NA | 0.69 (0.49–0.88) |
| Chaves et al (2017), Brazil | abnormal PR (ratio of the flow velocity of the second peak to that of the initial systolic velocity peak) ≥ 0.78 – < 0.98 | CNS injury (eclampsia or PRES) HELLP severe hypertension (SBP ≥ 160 mmHg or DBP ≥ 110 mmHg) ICU admission maternal death | 56 | 39 (69.64) | 13 | 20 | 4 | 19 | NA | NA | NA | NA | NA | NA | NA |
| Chaves et al (2017), Brazil | abnormal PR (ratio of the flow velocity of the second peak to that of the initial systolic velocity peak) ≥ 0.99 | CNS injury (eclampsia or PRES) HELLP severe hypertension (SBP ≥ 160 mmHg or DBP ≥ 110 mmHg) ICU admission maternal death | 56 | 39 (69.64) | 0 | 16 | 17 | 23 | NA | NA | NA | NA | NA | NA | NA |
| Chaves et al (2017), Brazil | abnormal PR (ratio of the flow velocity of the second peak to that of the initial systolic velocity peak) ≥ 0.905 | CNS injury (eclampsia or PRES) HELLP severe hypertension (SBP ≥ 160 mmHg or DBP ≥ 110 mmHg) ICU admission maternal death | 56 | 39 (69.64) | NA | NA | NA | NA | 0.692 | 0.941 | NA | NA | NA | NA | 0.821 (0.715–0.928) |
| **signs and/or symptoms** | | | | | | | | | | | | | | | |
| Li et al (2018), China | dizziness and headache | eclampsia placental abruption pulmonary oedema heart failure maternal death HELLP syndrome  severe hypoproteinemia (serum albumin < 25 g/L) | 1738 | 111 (6.39) | NA | NA | NA | NA | NA | NA | NA | NA | NA | NA | 0.576 (0.535–0.618) |
| Li et al (2018), China | palpitations and dyspnea | eclampsia placental abruption pulmonary oedema heart failure maternal death HELLP syndrome  severe hypoproteinemia (serum albumin < 25 g/L) | 1738 | 111 (6.39) | NA | NA | NA | NA | NA | NA | NA | NA | NA | NA | 0.527 (0.458–0.568) |
| Li et al (2018), China | nausea or vomiting | eclampsia placental abruption pulmonary oedema heart failure maternal death HELLP syndrome  severe hypoproteinemia (serum albumin < 25 g/L) | 1738 | 111 (6.39) | NA | NA | NA | NA | NA | NA | NA | NA | NA | NA | 0.527 (0.486–0.569) |
| Li et al (2018), China | visual symptoms | eclampsia placental abruption pulmonary oedema heart failure maternal death HELLP syndrome  severe hypoproteinemia (serum albumin < 25 g/L) | 1738 | 111 (6.39) | NA | NA | NA | NA | NA | NA | NA | NA | NA | NA | 0.526 (0.484–0.567) |
| Li et al (2018), China | upper abdominal pain | eclampsia placental abruption pulmonary oedema heart failure maternal death HELLP syndrome  severe hypoproteinemia (serum albumin < 25 g/L) | 1738 | 111 (6.39) | NA | NA | NA | NA | NA | NA | NA | NA | NA | NA | 0.515 (0.474–0.556) |
| Aziz et al (2011),  India | headache | DIC  acute renal failure  PPH  abruptio placenta cerebral or pulmonary oedema liver infarcts or rupture  subcapsular liver hematoma  maternal death | 74 | 20 (27.00) | NA | NA | NA | NA | 0.30 (0.12-0.54) | 0.46 (0.33-0.60) | NA | NA | 0.60 (0.30-1.10) | 1.50 (1.00-2.30) | 0.40 (0.20-0.50) |
| Aziz et al (2011),  India | vomiting | DIC  acute renal failure  PPH  abruptio placenta cerebral or pulmonary oedema liver infarcts or rupture  subcapsular liver hematoma  maternal death | 74 | 20 (27.00) | NA | NA | NA | NA | 0.10 (0.01-0.32) | 0.78 (0.64-0.88) | NA | NA | 0.50 (0.10-1.80) | 1.20 (0.90-1.40) | 0.40 (0.30-0.50) |
| Aziz et al (2011),  India | epigastric pain | DIC  acute renal failure  PPH  abruptio placenta cerebral or pulmonary oedema liver infarcts or rupture  subcapsular liver hematoma  maternal death | 74 | 20 (27.00) | NA | NA | NA | NA | 0.10 (0.01-0.32) | 0.70 (0.56-0.82) | NA | NA | 0.30 (0.10-1.30) | 1.30 (1.00-1.60) | 0.40 (0.30-0.50) |
| Aziz et al (2011),  India | non-specific viral symptoms | DIC  acute renal failure  PPH  abruptio placenta cerebral or pulmonary oedema liver infarcts or rupture  subcapsular liver hematoma  maternal death | 74 | 20 (27.00) | NA | NA | NA | NA | 0.65 (0.41-0.85) | 0.87 (0.75-0.95) | NA | NA | 5.00 (2.30-10.70) | 0.40 (0.20-0.70) | 0.80 (0.60-0.90) |
| Millman et al (2011), US | chest pain and/or dyspnea | non-respiratory PIERS composite outcomes | 1534 | 67 (4.40) | NA | NA | NA | NA | NA | NA | NA | NA | NA | NA | 0.53 (0.45-0.60) |
| Reddy et al (2022), Australia | abdominal circumference | ICU admission HELLP placental abruption eclampsia | 126 | 10 (7.94) | NA | NA | NA | NA | 0.28 (0.00–0.78) | NA | NA | NA | NA | NA | 0.76 (0.58–0.93) |
| Morikawa et al (2021), Japan | gestational weight gain during the week prior delivery ≥ | HELLP AFLP | 94 | 19 (20.2) | 35 | 12 | 40 | 7 | NA | NA | NA | NA | NA | NA | NA |
| Morikawa et al (2021), Japan | gestational weight gain during the week prior delivery ≥ 0.7kg | HELLP AFLP | 94 | 19 (20.2) | NA | NA | NA | NA | 0.895 | 0.427 | 0.283 | 0.941 | NA | NA | 0.629 |
| Morikawa et al (2021), Japan | gestational weight gain during the week prior delivery ≥ 1.6 kg | eclampsia PRES | 94 | 5 (5.3) | 45 | 2 | 44 | 3 | NA | NA | NA | NA | NA | NA | NA |
| Morikawa et al (2021), Japan | gestational weight gain during the week prior delivery ≥ 3.3 kg | eclampsia PRES | 94 | 5 (5.3) | NA | NA | NA | NA | 1 | 0.191 | 0.065 | 1 | NA | NA | 0.49 |
| Loardi et al (2021), Italy | pre-eclampsia diagnosis | HELLP  acute kidney failure pulmonary oedema neurologic complications DIC placental abruption | 311 | 41 (13.2) | NA | NA | NA | NA | 0.97 (0.87–0.99) | 0.36 (0.30–0.42) | 0.19 (0.14–0.25) | 0.99 (0.94–100) | 1.51 (1.37–1.67) | 0.69 (0.01–0.48) | NA |
| **biomedical/laboratory tests** | | | | | | | | | | | | | | | |
| Aziz et al (2011),  India | platelets ≤ 100 ×10^5/L | DIC  acute renal failure  PPH  abruptio placenta cerebral or pulmonary oedema liver infarcts or rupture  subcapsular liver hematoma  maternal death | 74 | 20 (27.00) | NA | NA | NA | NA | 0.70 (0.46-0.88) | 0.20 (0.11-0.34) | NA | NA | 0.90 (0.60-1.20) | 1.50 (0.60-3.40) | 0.40 (0.30-0.60) |
| Binder et al (2023), Austria | thrombocytopenia | ICU admission  pulmonary oedema  eclampsia  maternal death | 323 | 37 (11.5) | 25 | 7 | 261 | 30 | NA | NA | NA | NA | NA | NA | NA |
| Simón et al (2020), Spain | platelet count ≥ 308.5 platelets/µL | HELLP neurological events (eclampsia, stroke, cortical blindness, retinal detachment, posterior reversible encephalopathy syndrome) acute myocardial infarction hepatic subcapsular hematoma pulmonary oedema acute renal failure (serum creatinine > 1.2 mg/dL or need for dialysis)  placental abruption maternal death | 76 | 25 (32.89) | NA | NA | NA | NA | 0.05 (0.001–0.20) | 0.9 | 0.17 (0.02–0.62) | 0.66 (0.63–0.68) | 0.4 (0.1–3.3) | NA | 0.37 (0.21–0.52) |
| Aziz et al (2011),  India | AST ≥ 70 IU/L | DIC  acute renal failure  PPH  abruptio placenta cerebral or pulmonary oedema liver infarcts or rupture  subcapsular liver hematoma  maternal death | 74 | 20 (27.00) | NA | NA | NA | NA | 0.60 (0.36-0.81) | 0.43 (0.29-0.57) | NA | NA | 1.10 (0.70-1.60) | 0.90 (0.50-1.80) | 0.50 (0.40-0.60) |
| Simón et al (2020), Spain | AST concentration ≤ 32.5 U/L | HELLP neurological events (eclampsia, stroke, cortical blindness, retinal detachment, posterior reversible encephalopathy syndrome) acute myocardial infarction hepatic subcapsular hematoma pulmonary oedema acute renal failure (serum creatinine > 1.2 mg/dL or need for dialysis)  placental abruption maternal death | 76 | 25 (32.89) | NA | NA | NA | NA | 0.16 (0.045–0.36) | 0.9 | 0.44 (0.19–0.73) | 0.69 (0.64–0.73) | 1.6 (0.5–5.6) | NA | 0.53 (0.36–0.70) |
| Aziz et al (2011),  India | ALT ≥ 70 IU/L | DIC  acute renal failure  PPH  abruptio placenta cerebral or pulmonary oedema liver infarcts or rupture  subcapsular liver hematoma  maternal death | 74 | 20 (27.00) | NA | NA | NA | NA | 0.55 (0.32-0.77) | 0.26 (0.15-0.40) | NA | NA | 0.70 (0.50-1.10) | 1.70 (0.90-3.40) | 0.40 (0.30-0.50) |
| Li et al (2018), China | ALT concentration > 40 U/L | eclampsia placental abruption pulmonary oedema heart failure maternal death HELLP syndrome  severe hypoproteinemia (serum albumin < 25 g/L) | 1738 | 111 (6.39) | NA | NA | NA | NA | NA | NA | NA | NA | NA | NA | 0.582 (0.538–0.625) |
| Binder et al (2023), Austria | serum transaminase > 66 IU/L | ICU admission  pulmonary oedema  eclampsia  maternal death | 323 | 37 (11.5) | 53 | 5 | 233 | 32 | NA | NA | NA | NA | NA | NA | NA |
| Aziz et al (2011),  India | LDH ≥ 600 IU/L | DIC  acute renal failure  PPH  abruptio placenta cerebral or pulmonary oedema liver infarcts or rupture  subcapsular liver hematoma  maternal death | 74 | 20 (27.00) | NA | NA | NA | NA | 0.75. (0.51-0.91) | 0.56 (0.41-0.62) | NA | NA | 1.70 (1.10-2.50) | 0.50 (0.20-1.00) | 0.70 (0.60-0.80) |
| Binder et al (2023), Austria | LDH > 280 IU/L | ICU admission  pulmonary oedema  eclampsia  maternal death | 323 | 37 (11.5) | 86 | 17 | 200 | 20 | NA | NA | NA | NA | NA | NA | NA |
| Kumar et al (2023), India | LDH ≥ 615 | intravenous antihypertensive agents  ICU admission  eclampsia  placental abruption  HELLP syndrome  DIC  platelets <100x10^9/L,  creatinine >1.1 mg/dL  ALT >100 U/L  stroke  cortical blindness  acute pulmonary oedema  liver capsule rupture or hematoma  maternal death | 91 | 44 (48.9) | NA | NA | NA | NA | 0.5 | 0.85 | NA | NA | NA | NA | NA |
| Millman et al (2011), US | SpO_2_ < 93% | non-respiratory PIERS | 1534 | 67 (4.40) | NA | NA | NA | NA | NA | NA | NA | NA | NA | NA | 0.64 (0.57-0.71) |
| Scazzochio et al (2013), Spain | maternal NGAL > 100 ng/mL | eclampsia HELLP syndrome (lactate dehydrogenase >600 IU/l, aspartate transaminase >62 IU/l, platelet count)  acute renal failure defined as creatinine >1.2 mg/dl  subcapsular hepatic hematoma  pulmonary oedema DIC | 67 | 12 (17.90) | NA | NA | NA | NA | 0.42 (0.17-0.71) | 0.66 (0.51-0.77) | NA | NA | 1.20 (0.60-2.60) | 0.90 (0.50-1.50) | NA |
| Binder et al (2023), Austria | serum creatinine > 1 mg/dL | ICU admission  pulmonary oedema  eclampsia  maternal death | 323 | 37 (11.5) | 17 | 4 | 269 | 33 | NA | NA | NA | NA | NA | NA | NA |
| Kumar et al (2023), India | serum creatinine ≥ 0.79 mg/dL | intravenous antihypertensive agents  ICU admission  eclampsia  placental abruption  HELLP syndrome  DIC  platelets <100x10^9/L,  creatinine >1.1 mg/dL  ALT >100 U/L  stroke  cortical blindness  acute pulmonary oedema  liver capsule rupture or hematoma  maternal death | 91 | 44 (48.9) | NA | NA | NA | NA | 0.73 | 0.72 | NA | NA | NA | NA | NA |
| Li et al (2018), China | serum creatinine ≥ 97.25 µmol/L | eclampsia placental abruption pulmonary oedema heart failure maternal death HELLP syndrome  severe hypoproteinemia (serum albumin < 25 g/L) | 1738 | 111 (6.39) | NA | NA | NA | NA | NA | NA | NA | NA | NA | NA | 0.539 (0.495–0.583) |
| Simón et al (2020), Spain | serum creatinine ≥ 0.8 mg/dL | HELLP neurological events (eclampsia, stroke, cortical blindness, retinal detachment, posterior reversible encephalopathy syndrome) acute myocardial infarction hepatic subcapsular hematoma pulmonary oedema acute renal failure (serum creatinine > 1.2 mg/dL or need for dialysis)  placental abruption maternal death | 76 | 25 (32.89) | NA | NA | NA | NA | 0.11 (0.03–0.31) | 0.9 | 0.38 (0.14–0.70) | 0.68 (0.64–0.71) | 1.2 (0.3–4.7) | NA | 0.55 (0.40–0.70) |
| Kumar et al (2023), India | serum uric acid ≥ 7.55 mg/dL | intravenous antihypertensive agents  ICU admission  eclampsia  placental abruption  HELLP syndrome  DIC  platelets <100x10^9/L,  creatinine >1.1 mg/dL  ALT >100 U/L  stroke  cortical blindness  acute pulmonary oedema  liver capsule rupture or hematoma  maternal death | 91 | 44 (48.9) | NA | NA | NA | NA | 0.61 | 0.85 | NA | NA | NA | NA | NA |
| Joshi et al (2022), India | raised serum hs-CRP > 3.0 mg/dL | placental abruption  acute liver injury acute kidney injury DIC  HELLP neurological symptoms (including eclampsia) | 132 | 25 (18.94) | 37 | 23 | 70 | 2 | NA | NA | NA | NA | NA | NA | NA |
| Hong et al (2021), China | B–type natriuretic peptide > 118 pg/mL | eclampsia placental abruption HELLP cardiac insufficiency abnormal liver function (the liver enzyme > 3 times the normal value, and the normal value is about 40 U/L) acute renal insufficiency (creatinine > 150 μmol/L) central nervous system complications (such as cerebral haemorrhage and PRES) body cavity effusion (new–onset pleural effusion, pericardial effusion and ascites) pulmonary hypertension | 284 | 89 (31.3) | NA | NA | NA | NA | NA | NA | NA | NA | NA | NA | 0.739 (0.684–0.789) |
| Kumari et al (2017), India | N terminal pro–brain natriuretic peptide > 100 pg/mL | eclampsia congestive heart failure HELLP pulmonary oedema cerebrovascular accident  renal dysfunction hypertensive retinopathy placental abruption | 45 | 19 (42.2) | NA | NA | NA | NA | 0.94 | 0.5 | 0.58 | 0.93 | NA | NA | NA |
| Kumari et al (2017), India | N terminal pro–brain natriuretic peptide > 200 pg/mL | eclampsia congestive heart failure HELLP pulmonary oedema cerebrovascular accident  renal dysfunction hypertensive retinopathy placental abruption | 45 | 19 (42.2) | NA | NA | NA | NA | 0.89 | 0.73 | 0.71 | 0.9 | NA | NA | NA |
| Kumari et al (2017), India | N terminal pro–brain natriuretic peptide > 500 pg/mL | eclampsia congestive heart failure HELLP pulmonary oedema cerebrovascular accident  renal dysfunction hypertensive retinopathy placental abruption | 45 | 19 (42.2) | NA | NA | NA | NA | 0.78 | 0.9 | 0.83 | 0.87 | NA | NA | NA |
| Elia et al (2017), UK | log transformed ACR | use of intravenous magnesium sulfate for seizure prophylaxis use of intravenous antihypertensives ICU admission placental abruption eclampsia HELLP | 717 | 204 (28.45) | NA | NA | NA | NA | NA | NA | NA | NA | NA | NA | 0.70 (0.66–0.74) |
| Morikawa et al (2020), Japan | serum total protein level at PE diagnosis ≥ 49 g/L | eclampsia PRES | 94 | 50 (5.3) | NA | NA | NA | NA | 0.4 | 0.764 | 0.087 | 0.958 | NA | NA | 0.521 |
| Morikawa et al (2020), Japan | serum total protein level at delivery ≥ 50 g/L | eclampsia PRES | 94 | 50 (5.3) | NA | NA | NA | NA | 0.6 | 0.629 | 0.083 | 0.966 | NA | NA | 0.593 |
| Morikawa et al (2020), Japan | serum total protein level at PE diagnosis ≥ 49 g/L | HELLP AFLP | 94 | 20 (20.2) | NA | NA | NA | NA | 0.368 | 0.787 | 0.304 | 0.831 | NA | NA | 0.503 |
| Morikawa et al (2020), Japan | serum total protein level at delivery ≥ 54 g/L | HELLP AFLP | 94 | 20 (20.2) | NA | NA | NA | NA | 0.895 | 0.467 | 0.298 | 0.946 | NA | NA | 0.586 |
| **fetal variables** | | | | | | | | | | | | | | | |
| Reddy et al (2022), Australia | EFW percentile | need for parenteral antihypertensive agents ICU admission  eclampsia placental abruption HELLP DIC thrombocytopenia (platelets <100×10^9/L) creatinine >90μmol/L ALT >100 U/L | 126 | 49 (38.9) | NA | NA | NA | NA | 0.12 (0.05–0.31) | NA | NA | NA | NA | NA | 0.61 (0.50 – 0.71) |
| Reddy et al (2022), Australia | EFW percentile | ICU admission HELLP placental abruption eclampsia | 126 | 10 (7.94) | NA | NA | NA | NA | 0.26 (0.59–0.56) (not reliable) | NA | NA | NA | NA | NA | 0.72 (0.53–0.90) |
| **Diagnosis** | | | | | | | | | | | | | | | |
| Wei et al (2022), China | PNI | HELLP  hypertensive retinopathy postpartum haemorrhage placental abruption  heart failure respiratory failure  CKD | 733 | 163 (22.24) | 209 | 97 | 361 | 66 | 0.62 | 0.38 | NA | NA | NA | NA | 0.628 |
| **multivariable models** | | | | | | | | | | | | | | | |
| Chan et al (2005), Australia | spot urine protein/creatinine >500  maternal age > 35 years | severe hypertension (> 170/110 mmHg)  renal insufficiency (creatinine > 90 µmol/L)  liver disease (AST >40 U/L)  cerebral irritation (hyperreflexia with clonus or repeated visual scotomata, requiring magnesium sulphate)  thrombocytopenia (platelets <150×10^9/L | 321 | 109 (34.00) | NA | NA | NA | NA | 0.10 (0.05-0.18) | 1.00 (0.98-1.00) | NA | NA | NA | 0.90 (0.80-1.00) | 0.67 (0.55-0.71) |
| Reddy et al (2022), Australia | EFW percentiles serum sFlt-1 | need for parenteral antihypertensive agents ICU admission  eclampsia placental abruption HELLP DIC thrombocytopenia (platelets <100×10^9/L) creatinine >90μmol/L ALT >100 U/L | 126 | 49 (38.9) | NA | NA | NA | NA | 0.22(0.08–0.43) | NA | NA | NA | NA | NA | 0.71 (0.61–0.80) |
| Girling et al (1997), UK | AST=30 U/L  ALT= 32 U/L  bilirubin= 14 U/L  GGT= 41 U/L | Maternal complications considered to be serious medical problems secondary to pre-eclampsia | 35 | 7 (20.00) | NA | NA | NA | NA | 1.00 (0.56-1.00) | 0.57 (0.37-0.75) | NA | NA | 2.30 (1.50-3.60) | NA | NA |
| Hong et al (2021), China | B–type natriuretic peptide SBP AST plasma albumin  blood creatinine | eclampsia placental abruption HELLP cardiac insufficiency abnormal liver function (the liver enzyme > 3 times the normal value, and the normal value is about 40 U/L) acute renal insufficiency (creatinine > 150 μmol/L) central nervous system complications (such as cerebral haemorrhage and PRES) body cavity effusion (new–onset pleural effusion, pericardial effusion and ascites) pulmonary hypertension | 284 | 89 (31.3) | NA | NA | NA | NA | NA | NA | NA | NA | NA | NA | 0.844 (0.796–0.884) |
| Paul et al (2019), Australia | SBP (> 125 mmHg) essential hypertension haemoglobin (> 70 g/L) serum creatinine (>30 µmol/L) | severe hypertension  thrombocytopenia (platelet count < 100,000/µL) impaired liver function (abnormally elevated liver enzymes to twice the normal concentration) renal insufficiency (elevated serum creatinine concentration) pulmonary oedema new onset cerebral/visual disturbance ICU admission PPH | 124 | 55 (44.35) | NA | NA | NA | NA | 0.7 | 0.9 | 0.87 | 0.8 | NA | NA | 0.88 (0.82–0.94) |
| Elia et al (2017), UK | log transformed ACR  gestational age at ACR measurement essential hypertension preexisting diabetes gestational diabetes social deprivation index BMI MAP current smoking status parity maternal age | use of intravenous magnesium sulfate for seizure prophylaxis use of intravenous antihypertensives ICU admission placental abruption eclampsia HELLP | 717 | 204 (28.45) | NA | NA | NA | NA | NA | NA | NA | NA | NA | NA | 0.76 (0.72–0.80) |
| Tan et al (2020), China | gestational age placenta previa HBsAg positivity cardiac diseases IDA dyspnea systolic blood pressure at admission log–transformed platelets log–transformed fibrinogen log–transformed aspartate transferase log–transformed total bilirubin log–transformed creatinine qualitative proteinuria | maternal death near–miss (series of organ dysfunction, including respiratory dysfunction, cardiovascular dysfunction, renal dysfunction, coagulation/blood dysfunction, liver function disorder, neurological dysfunction, and uterine dysfunction) cortical blindness/retinal detachment Bell’s palsy | 2793 | 397 (14.21) | NA | NA | NA | NA | 0.73 | 0.76 | 0.36 | 0.94 | NA | NA | 82.15 (79.60–84.70) |
| Tan et al (2020), China | gestational age placenta previa HBsAg positivity cardiac diseases IDA dyspnea systolic blood pressure at admission log–transformed platelets log–transformed fibrinogen log–transformed aspartate transferase log–transformed total bilirubin log–transformed creatinine qualitative proteinuria | acute azotemia  massive blood transfusion | 2793 | 269 (9.63) | NA | NA | NA | NA | 0.66 | 0.87 | 0.37 | 0.96 | NA | NA | 84.91 (82.17–87.65) |
| Malhamé et al (2022_2), US | maternal age maternal education  previous live births previous cesarean delivery multiple gestation  chronic hypertension gestational diabetes gestational age at delivery (weeks) | pulmonary oedema acute heart failure acute myocardial infarction aneurysm cardiac arrest/ventricular fibrillation heart failure/arrest during surgery or procedure puerperal cerebrovascular disorders  cardiogenic shock conversion of cardiac rhythm  difficult–to–control severe hypertension (SBP/DBP ≥ 160/110 mmHg) | 4171 | 376 (9.01) | NA | NA | NA | NA | NA | NA | NA | NA | NA | NA | 0.72 (0.69–0.74) |
| Malhamé et al (2022_2), US | maternal age maternal education  previous live births previous cesarean delivery multiple gestation  chronic hypertension gestational diabetes gestational age at delivery (weeks) | pulmonary oedema acute heart failure acute myocardial infarction aneurysm cardiac arrest/ventricular fibrillation heart failure/arrest during surgery or procedure puerperal cerebrovascular disorders  cardiogenic shock conversion of cardiac rhythm | 4171 | 99 (2.37) | NA | NA | NA | NA | NA | NA | NA | NA | NA | NA | 0.78 (0.74–0.83) |
| Li et al (2018), China | 24 h proteinuria (≥ 2.0 g/24 h) renal insufficiency (Serum creatinine > 97.25 µmol/L) liver dysfunction (ALT > 40 U/L) thrombocytopenia (platelet count < 100x10^9/L) hypoproteinemia (serum albumin < 25g/L) upper abdominal pain palpitations or dyspnea gestational age < 34 weeks predicted probability 0.3 | eclampsia placental abruption pulmonary oedema heart failure maternal death HELLP severe hypoproteinemia (serum albumin < 25 g/L) | 1738 | 111 (6.39) | NA | NA | NA | NA | 0.4 | 0.93 | NA | NA | 5.40 (4.2–6.9) |  | 0.800 (0.769–0.830) |
| **Abbreviations:** ACR, creatinine/albumin ratio; AFLP, acute fatty liver of pregnancy; ALT, alanine transaminase; AST, aspartate transaminase; AUROC, area under the receiver operating characteristic curve; BMI, Body mass index; CKD, chronic kidney disease; CNS, central nervous system; DBP, diastolic blood pressure; DIC, disseminated intravascular coagulation; EFW, estimated fetal weight; FN, false negative; FP, false positive; GGT; gamma glutamyl transferase; HBsAg, Hepatitis B surface antigen; HELLP, hemolysis, elevated liver enzymes and low platelet count; hs-CRP, high-sensitive C-reactive protein; ICU, intensive care unit; IDA, iron deficiency anemia; LDH, lactate dehydrogenase; LR-, negative likelihood ratio; LR+, positive likelihood ratio; MAP, mean arterial pressure; NA, not available; NGAL, neutrophil gelatinase-associated lipocalin; NPV, negative predictive value; PE, pre-eclampsia; PI, pulsatility index; PlGF, placental growth factor; PNI, prognostic nutritional index; PPH, postpartum haemorrhage; PPV, positive predictive value; PR, peak ratio; PRES, posterior reversible encephalopathy syndrome; SBP, systolic blood pressure; sFlt-1, soluble fms-like tyrosine kinase-1; SpO_2_, oxygen saturation; TN, true negative; TP, true positive; UPCR, urinary protein to creatinine ratio; UtA, uterine artery. | | | | | | | | | | | | | | | |

**Supplementary table 9: Predictive performance of all prediction tests other than fullPIERS model for PIERS outcomes**

| **Study** | **Predictor** | **Outcome** | **Sample size** | **Event rate, n (%)** | **Sensitivity** | **Specificity** | **PPV** | **NPV** | **LR+** | **LR-** | **AUROC** |
| --- | --- | --- | --- | --- | --- | --- | --- | --- | --- | --- | --- |
| Ahmad et al (2023), India | gestational age < 34 at diagnosis | PIERS composite outcomes | 384 | 104 (27.10) | 0.14 (0.09-0.22) | 0.90 (0.86-0.93) | 0.36 (0.23-0.51) | 0.74 (0.69-0.78) | 1.50 (0.83-2.70) | 0.95 (0.87-1.03) | 0.52 (0.49-0.56) |
| Ahmad et al (2023), India | gestational age < 36 at diagnosis | PIERS composite outcomes | 384 | 104 (27.10) | 0.63 (0.54-0.72) | 0.64 (0.58-0.69) | 0.40 (0.32-0.47) | 0.82 (0.77-0.87) | 1.76 (1.42-2.18) | 0.57 (0.44-0.75) | 0.64 (0.58-0.69) |
| Ahmad et al (2023), India | gestational age < 39 at diagnosis | PIERS composite outcomes | 384 | 104 (27.10) | 1.00 (0.96-1.00) | 0.13 (0.09-0.17) | 0.30 (0.25-0.35) | 1.00 (0.90-1.00) | 1.14 (1.09-1.20)* | 0.04 (0.00-0.59)* | 0.56 (0.54-0.58) |
| Payne et al (2011), US | dipstick | PIERS composite outcomes | 2002 | 106 (5.30) | NA | NA | NA | NA | NA | NA | 0.55 (0.49-0.61) |
| Payne et al (2011), US | 24 h urine | PIERS composite outcomes | 2002 | 106 (5.30) | NA | NA | NA | NA | NA | NA | 0.55 (0.47-0.63) |
| Payne et al (2011), US | spot urine protein creatinine ratio | PIERS composite outcomes | 2002 | 106 (5.30) | NA | NA | NA | NA | NA | NA | 0.48 (0.42-0.55) |
| Nóbrega et al (2022), Brazil | serum sFlt-1 ≥ 7439 pg/mL | PIERS composite outcomes | 60 | 13 (21.70) | 0.77 | 0.76 | NA | NA | NA | NA | 0.77 (0.65–0.89) |
| Heimberger et al (2020), US | sFlt-1/PlGF ratio ≥ 85 | PIERS composite outcomes | 115 | 12 (10.43) | 0.75 (0.47-0.91) | 0.73 (0.64-0.80) | 0.24 (0.13-0.40) | 0.96 (0.89-0.99) | 2.76 (1.75-4.35) | 0.34 (0.13-0.92) | 0.74 (0.60-0.87) |
| Yen et al (2011), US | headache | PIERS composite outcomes | 2020 | 143 (7.10) | NA | NA | NA | NA | NA | NA | 0.53 (0.47-0.58) |
| Ahmad et al (2023), India | chest pain | PIERS composite outcomes | 384 | 104 (27.10) | 0.51 (0.41-0.60) | 0.92 (0.88-0.94) | 0.70 (0.59-0.79) | 0.83 (0.79-0.87) | 6.20 (4.02-9.58) | 0.53 (0.44-0.65) | 0.71 (0.66-0.76) |
| Millman et al (2011), US | chest pain and/or dyspnea | PIERS composite outcomes | 1534 | 94 (6.10) | NA | NA | NA | NA | NA | NA | 0.59 (0.52-0.65) |
| Yen et al (2011), US | chest pain or dyspnea | PIERS composite outcomes | 2020 | 143 (7.10) | NA | NA | NA | NA | NA | NA | 0.58 (0.52-0.64) |
| Yen et al (2011), US | nausea/vomiting | PIERS composite outcomes | 2020 | 143 (7.10) | NA | NA | NA | NA | NA | NA | 0.54 (0.48-0.60) |
| Yen et al (2011), US | right upper quadrant or epigastric pain | PIERS composite outcomes | 2020 | 143 (7.10) | NA | NA | NA | NA | NA | NA | 0.61 (0.55-0.66) |
| Yen et al (2011), US | abdominal pain or vaginal bleeding | PIERS composite outcomes | 2020 | 143 (7.10) | NA | NA | NA | NA | NA | NA | 0.57 (0.47-0.67) |
| Yen et al (2011), US | visual symptoms | PIERS composite outcomes | 2020 | 143 (7.10) | NA | NA | NA | NA | NA | NA | 0.50 (0.45-0.56) |
| Ahmad et al (2023), India | SpO_2_ < 94.9 % | PIERS composite outcomes | 384 | 104 (27.10) | 0.60 (0.50-0.69) | 0.88 (0.83-0.91) | 0.64 (0.54-0.73) | 0.85 (0.81-0.89) | 4.77 (3.37-6.75) | 0.46 (0.36-0.59) | 0.74 (0.68-0.79) |
| Millman et al (2011), US | SpO_2_ < 93% | PIERS composite outcomes | 1534 | 94 (6.10) | NA | NA | NA | NA | NA | NA | 0.71 (0.65-0.77) |
| Ahmad et al (2023), India | platelet count < 100 000/cumm | PIERS composite outcomes | 384 | 104 (27.10) | 0.34 (0.25-0.43) | 0.82 (0.77-0.86) | 0.41 (0.31-0.51) | 0.77 (0.72-0.81) | 1.85 (1.28-2.67) | 0.81 (0.70-0.94) | 0.58 (0.53-0.63) |
| Laskin et al (2011), US | platelets ≤ 100×10^9/L | PIERS composite outcomes | 1405 | 152 (10.80) | 0.16 (0.11-0.23) | 0.92 (0.91-0.94) | NA | NA | 2.00 (1.20-3.10) | 0.90 (0.90-1.00) | NA |
| Livingston et al (2014), US | uric acid 345 > µmol/L | PIERS composite outcomes | 1487 | 198 (13.30) | 0.80 (0.71.0.88) | 0.28 (0.26-0.31) | NA | NA | 1.10 (1.00-1.20) | 0.70 (0.50-1.00) | 0.62 (0.56-0.69) |
| Kozic et al (2011), US | serum albumin | PIERS composite outcomes | 2008 | 102 (5.10) | NA | NA | NA | NA | NA | NA | 0.63 (0.57-0.69) |
| Kozic et al (2011), US | ALT | PIERS composite outcomes | 2008 | 102 (5.10) | NA | NA | NA | NA | NA | NA | 0.73 (0.67-0.79) |
| Ahmad et al (2023), India | serum AST > 40 IU/L | PIERS composite outcomes | 384 | 104 (27.10) | 0.94 (0.88-0.97) | 0.26 (0.22-0.32) | 0.32 (0.27-0.38) | 0.92 (0.85-0.97) | 1.28 (1.18-1.39) | 0.22 (0.10-0.49) | 0.60 (0.57-0.64) |
| Kozic et al (2011), US | AST | PIERS composite outcomes | 2008 | 102 (5.10) | NA | NA | NA | NA | NA | NA | 0.73 (0.67-0.79) |
| Wang et al (2019), China | LDH ≥ 243.5 U/L | PIERS composite outcomes | 1,430 | 262 (18.32) | NA | NA | NA | NA | NA | NA | 0.61 |
| Kozic et al (2011), US | LDH | PIERS composite outcomes | 2008 | 102 (5.10) | NA | NA | NA | NA | NA | NA | 0.74 (0.68-0.81) |
| Ahmad et al (2023), India | serum creatinine > 1.4 mg/dL | PIERS composite outcomes | 384 | 104 (27.10) | 0.20 (0.14-0.29) | 0.88 (0.83-0.91) | 0.38 (0.26-0.51) | 0.75 (0.70-0.79) | 1.62 (0.99-2.64) | 0.91 (0.82-1.01) | 0.54 (0.50-0.58) |
| Kozic et al (2011), US | total bilirubin | PIERS composite outcomes | 2008 | 102 (5.10) | NA | NA | NA | NA | NA | NA | 0.68 (0.61-0.74) |
| Nóbrega et al (2022), Brazil | serum hs-CRP ≥ 28.75 mg/L | PIERS composite outcomes | 60 | 13 (21.70) | 0.61 | 0.79 | NA | NA | NA | NA | 0.71 (0.56–0.86) |
| Kozic et al (2011), US | INR | PIERS composite outcomes | 2008 | 102 (5.10) | NA | NA | NA | NA | NA | NA | 0.65 (0.58-0.71) |
| Magee et al (2021), multinational | pre-eclampsia definition A (development of proteinuria) in chronic hypertension | PIERS composite outcomes | 731 | 18 (2.46) | 0.44 (0.25-0.66) | 0.74 (0.71-0.77) | 0.04 (0.02-0.08) | 0.98 (0.97-0.99) | 1.70 (1.00-2.90) | 0.75 (0.50-1.14) | 0.59 (0.47-0.71) |
| Magee et al (2021), multinational | pre-eclampsia definition B (development of proteinuria AND one or more symptoms, signs, abnormal laboratory test) in chronic hypertension | PIERS composite outcomes | 731 | 18 (2.46) | 0.39 (0.20-0.61) | 0.87 (0.84-0.89) | 0.07 (0.03-0.13) | 0.98 (0.97-0.99) | 2.92 (1.59-5.36) | 0.71 (0.49-1.02) | 0.63 (0.51-0.74) |
| Magee et al (2021), multinational | pre-eclampsia definition C (one or more symptoms, sings) in chronic hypertension | PIERS composite outcomes | 731 | 18 (2.46) | 0.72 (0.49-0.88) | 0.69 (0.65-0.72) | 0.06 (0.03-0.09) | 0.99 (0.98-1.00) | 2.31 (1.70-3.14) | 0.40 (0.19-0.85) | 0.70 (0.60-0.81) |
| Magee et al (2021), multinational | pre-eclampsia definition D (one or more symptoms, signs, abnormal laboratory test) in chronic hypertension | PIERS composite outcomes | 731 | 18 (2.46) | 0.78 (0.55-0.91) | 0.68 (0.65-0.71) | 0.06 (0.03-0.09) | 0.99 (0.98-1.00) | 2.43 (1.86-3.18) | 0.33 (0.14-0.78) | 0.73 (0.63-0.83) |
| Magee et al (2021), multinational | pre-eclampsia definition E (development of proteinuria OR one or more symptoms, signs, abnormal laboratory test) in chronic hypertension | PIERS composite outcomes | 731 | 18 (2.46) | 0.83 (0.61-0.94) | 0.55 (0.52-0.59) | 0.04 (0.03-0.07) | 0.99 (0.98-1.00) | 1.86 (1.49-2.33) | 0.30 (0.11-0.85) | 0.69 (0.60-0.78) |
| Magee et al (2021), multinational | pre-eclampsia definition F (development of proteinuria development of proteinuria OR one or more symptoms, signs) in chronic hypertension | PIERS composite outcomes | 731 | 18 (2.46) | 0.78 (0.55-0.91) | 0.56 (0.52-0.59) | 0.04 (0.03-0.07) | 0.99 (0.97-1.00) | 1.76 (1.36-2.28) | 0.40 (0.17-0.95) | 0.67 (0.57-0.77) |
| Magee et al (2021), multinational | pre-eclampsia definition A (development of proteinuria) in gestational hypertension | PIERS composite outcomes | 248 | 10 (4.03) | 0.40 (0.17-0.69) | 0.66 (0.59-0.71) | 0.05 (0.02-0.11) | 0.96 (0.92-0.98) | 1.16 (0.53-2.53) | 0.92 (0.55-1.53) | 0.53 (0.36-0.69) |
| Magee et al (2021), multinational | pre-eclampsia definition B (development of proteinuria AND one or more symptoms, signs, abnormal laboratory test) in gestational hypertension | PIERS composite outcomes | 248 | 10 (4.03) | 0.30 (0.11-0.60) | 0.79 (0.73-0.84) | 0.06 (0.02-0.15) | 0.96 (0.93-0.98) | 1.43 (0.54-3.80) | 0.89 (0.59-1.34) | 0.54 (0.39-0.70) |
| Magee et al (2021), multinational | pre-eclampsia definition C (one or more symptoms, sings) in gestational hypertension | PIERS composite outcomes | 248 | 10 (4.03) | 0.50 (0.24-0.76) | 0.59 (0.52-0.65) | 0.05 (0.02-0.11) | 0.97 (0.92-0.99) | 1.21 (0.64-2.30) | 0.85 (0.45-1.59) | 0.54 (0.38-0.71) |
| Magee et al (2021), multinational | pre-eclampsia definition D (one or more symptoms, signs, abnormal laboratory test) in gestational hypertension | PIERS composite outcomes | 248 | 10 (4.03) | 0.50 (0.24-0.76) | 0.58 (0.52-0.64) | 0.05 (0.02-0.11) | 0.97 (0.92-0.99) | 1.20 (0.64-2.27) | 0.86 (0.46-1.61) | 0.54 (0.38-0.71) |
| Magee et al (2021), multinational | pre-eclampsia definition E (development of proteinuria OR one or more symptoms, signs, abnormal laboratory test) in gestational hypertension | PIERS composite outcomes | 248 | 10 (4.03) | 0.60 (0.31-0.83) | 0.45 (0.39-0.51) | 0.04 (0.02-0.09) | 0.96 (0.91-0.99) | 1.09 (0.65-1.83) | 0.89 (0.41-1.93) | 0.52 (0.36-0.69) |
| Magee et al (2021), multinational | pre-eclampsia definition F (development of proteinuria development of proteinuria OR one or more symptoms, signs) in gestational hypertension | PIERS composite outcomes | 248 | 10 (4.03) | 0.60 (0.31-0.83) | 0.45 (0.39-0.51) | 0.04 (0.02-0.09) | 0.96 (0.91-0.99) | 1.09 (0.65-1.83) | 0.89 (0.41-1.93) | 0.52 (0.36-0.69) |
| Ukah et al (2020), multinational | fullPIERs model  serum PlGF < 100 pg/mL | PIERS composite outcomes | 541 | 44 (8.10) | 0.66 | 0.16 | NA | NA | NA | NA | 0.67 (0.59–0.75) |
| Chen et al (2022), China | multivariable model based on random forest method (all 77 variables) | PIERS composite outcomes | 1,829 | 102 (5.58) | 0.78 (0.73-0.82) | 0.99 (0.99-1.00) | NA | NA | NA | NA | 0.99 (0.98–0.99) |
| Millman et al (2011), US | Chest pain and/or dyspnea  SpO_2_ | PIERS composite outcomes | 1534 | 94 (6.10) | NA | NA | NA | NA | NA | NA | 0.73 (0.67-0.78) |
| Zheng et al (2022), China | multivariable model based on support vector machine (with imputation | PIERS composite outcomes | 733 | 182 (24.83) | 0.92 (0.88-0.95) | 0.92 (0.89-0.94) | 0.83 (0.78-0.88) | 0.97 (0.94-0.98) | 12.06 (8.66-16.78) | 0.08 (0.05-0.14) | 0.92 (0.90-0.95) |
| Confidence intervals for the sensitivity, specificity, positive and negative predictive value were calculated using the Wilson's score method. Confidence intervals for the positive and negative likelihood ratios were calculated using Simel's method. Confidence intervals for AUROC were calculated using the method of DeLong. **Abbreviations:** ALT, alanine transaminase; AST, aspartate transaminase; AUROC, area under the receiver operating characteristic curve; hs-CRP, high-sensitive C-reactive protein; INR, international normalized ratio; LDH, lactate dehydrogenase; LR+, positive likelihood ratio; LR-, negative likelihood ratio; NPV, negative predictive value; NA, not available; PIERS, Pre-eclampsia Integrated Estimate of RiSk; PlGF, placental growth factor; PPV, positive predictive value; sFlt-1, soluble fms-like tyrosine kinase-1; SpO_2_, oxygen saturation. | | | | | | | | | | | |

**Supplementary table 10: Predictive performance of all prediction tests for single perinatal outcomes.**

| **Study** | **Predictor** | **Outcome** | **Sample size** | **Event rate, n (%)** | **Sensitivity** | **Specificity** | **PPV** | **NPV** | **LR+** | **LR-** | **AUROC** |
| --- | --- | --- | --- | --- | --- | --- | --- | --- | --- | --- | --- |
| **Death** | | | | | | | | | | | |
| Carter et al (2017), US | SGA (birthweight less than 10th% on the Alexander growth standard) | neonatal death | 902 | 31 (3.44) | 0.06 (0.02-0.21) | 0.82 (0.80-0.85) | 0.01 (0.00-0.05) | 0.96 (0.94-0.97) | 0.36 (0.09-1.40) | 1.14 (1.03-1.25) | 0.44 (0.40-0.49) |
| Chadha et al (2022), India | UPCR ≥ 0.3 | neonatal death | 130 | 4 (2.80) | 1.00 (0.51-1.00) | 0.11 (0.07-0.17) | 0.03 (0.01-0.08) | 1.00 (0.80-1.00) | 1.01 (0.75-1.37)* | 0.89 (0.06-12.87)* | 0.55 (0.53-0.58) |
| Chaves et al (2017), Brazil | abnormal PR (ratio of the flow velocity of the second peak to that of the initial systolic velocity peak) ≥ 0.78 | neonatal death | 56 | 3 (5.36) | 1.00 (0.44-1.00) | 0.13 (0.07-0.25) | 0.06 (0.02-0.17) | 1.00 (0.65-1.00) | 1.02 (0.69-1.49)* | 0.90 (0.06-13.08)* | 0.57 (0.52-0.61) |
| Chaves et al (2017), Brazil | abnormal PR (ratio of the flow velocity of the second peak to that of the initial systolic velocity peak) ≥ 0.99 | neonatal death | 56 | 3 (5.36) | 0.67 (0.21-0.94) | 0.74 (0.60-0.84) | 0.12 (0.03-0.36) | 0.97 (0.87-1.00) | 2.52 (1.01-6.32) | 0.45 (0.09-2.26) | 0.70 (0.37-1.00) |
| Fishel Bartal et al (2022), US | Ethnicity: Non-Hispanic White | neonatal death | 389,347 | 1202 (0.31) | 0.39 (0.36-0.42) | 0.50 (0.50-0.50) | 0.00 (0.00-0.00) | 1.00 (1.00-1.00) | 0.78 (0.73-0.84) | 1.22 (1.17-1.28) | 0.44 (0.43-0.46) |
| Fishel Bartal et al (2022), US | Ethnicity: Non-Hispanic Black | neonatal death | 389,347 | 1202 (0.31) | 0.43 (0.40-0.46) | 0.70 (0.70-0.71) | 0.00 (0.00-0.00) | 1.00 (1.00-1.00) | 1.46 (1.37-1.56) | 0.81 (0.77-0.85) | 0.57 (0.55-0.58) |
| Fishel Bartal et al (2022), US | Ethnicity: Hispanic | neonatal death | 389,347 | 1202 (0.31) | 0.13 (0.12-0.15) | 0.85 (0.84-0.85) | 0.00 (0.00-0.00) | 1.00 (1.00-1.00) | 0.86 (0.75-1.00) | 1.02 (1.00-1.05) | 0.49 (0.48-0.50) |
| Fishel Bartal et al (2022), US | Ethnicity: Non-Hispanic Asian American, Native Hawaiian and other Pacific Islander | neonatal death | 389,347 | 1202 (0.31) | 0.03 (0.02-0.04) | 0.96 (0.96-0.96) | 0.00 (0.00-0.00) | 1.00 (1.00-1.00) | 0.82 (0.59-1.13) | 1.01 (1.00-1.02) | 0.50 (0.49-0.50) |
| Fishel Bartal et al (2022), US | Ethnicity: Non-Hispanic American Indian and Alaska Native | neonatal death | 389,347 | 1202 (0.31) | 0.01 (0.01-0.02) | 0.99 (0.99-0.99) | 0.00 (0.00-0.01) | 1.00 (1.00-1.00) | 1.30 (0.81-2.09) | 1.00 (0.99-1.00) | 0.50 (0.50-0.50) |
| Gupta et al (2017), India | absent end diastolic flow | neonatal death | 100 | 26 (26.00) | 0.69 (0.50-0.83) | 1.00 (0.95-1.00) | 1.00 (0.82-1.00) | 0.90 (0.82-0.95) | 102.78 (6.41-1647.36)* | 0.32 (0.18-0.55)* | 0.85 (0.76-0.94) |
| Gupta et al (2017), India | reduced end diastolic flow | neonatal death | 100 | 26 (26.00) | 0.08 (0.02-0.24) | 1.00 (0.95-1.00) | 1.00 (0.34-1.00) | 0.76 (0.66-0.83) | 13.89 (0.69-280.18)* | 0.91 (0.81-1.03)* | 0.54 (0.49-0.59) |
| Joshi et al (2022), India | raised serum hs-CRP > 3.0 mg/mL | neonatal death | 132 | 5 (3.79) | 1.00 (0.57-1.00) | 0.57 (0.48-0.65) | 0.08 (0.04-0.18) | 1.00 (0.95-1.00) | 2.11 (1.55-2.89)* | 0.15 (0.01-2.10)* | 0.78 (0.74-0.83) |
| Karge et al (2022), Germany | BMI ≥ 25 kg/m^2 | neonatal death | 141 | 1 (0.71) | 1.00 (0.21-1.00) | 0.60 (0.52-0.68) | 0.02 (0.00-0.09) | 1.00 (0.96-1.00) | 1.87 (0.82-4.27)* | 0.42 (0.04-4.62)* | NA |
| Karge et al (2022), Germany | BMI ≥ 30 kg/m^2 | neonatal death | 141 | 1 (0.71) | 1.00 (0.21-1.00) | 0.81 (0.73-0.86) | 0.04 (0.01-0.18) | 1.00 (0.97-1.00) | 3.85 (1.61-9.16)* | 0.31 (0.03-3.43)* | NA |
| Kesireddy et al (2021), India | GlyFn > 350 µg/mL | neonatal death | 51 | ≥0 (0.00) | 0.00 | 0.41 (0.29-0.55) | 0.00 (0.00-0.11) | 1.00 (0.85-1.00) | 0.85 (0.12-6.13)* | 1.21 (0.17-8.82)* | NA |
| Le et al (2018), Vietnam | serum uric acid level > 378 µmol/L | neonatal death (first 4 weeks after birth) | 205 | NA (4.90) | 0.90 | 0.60 | NA | NA | NA | NA | 0.76 |
| Loardi et al (2021), Italy | abnormal UtA PI > 95th percentile | neonatal death | 311 | 7 (2.25) | 1.00 (0.65-1.00) | 0.41 (0.35-0.46) | 0.04 (0.02-0.08) | 1.00 (0.97-1.00) | 1.58 (1.29-1.94)* | 0.15 (0.01-2.25)* | 0.70 (0.68-0.73) |
| Saxena et al (2021), India | CPR < 1 | neonatal death | 150 | 4 (2.67) | 0.10 (0.02-0.27) | 0.99 (0.95-1.00) | 0.75 (0.19-0.99) | 0.82 (0.74-0.87) | 3.80 (0.09-168.87)* | 0.92 (0.68-1.25)* | 0.50 (0.50-0.50) |
| Stolz et al (2018), Austria | sFlt-1/PlGF ratio ≥ 655 | neonatal death | 54 | 5 (8.33) | 0.60 (0.23-0.88) | 0.51 (0.37-0.64) | 0.11 (0.04-0.28) | 0.93 (0.77-0.98) | 1.23 (0.57-2.65) | 0.78 (0.26-2.37) | 0.56 (0.30-0.81) |
| Tokalioglu et al (2023), Turkey | umbilical artery half peak systolic velocity deceleration time (UA hPSV-DT) < 5th percentile | neonatal death | 55 | 1 (1.82) | 0.00 (0.00-0.79) | 0.59 (0.46-0.71) | 0.00 (0.00-0.15) | 0.97 (0.85-0.99) | 0.61 (0.05-6.88)* | 1.27 (0.55-2.91)* | NA |
| Chadha et al (2022), India | UPCR ≥ 0.3 | fetal death/stillbirth (babies with no signs of life in utero after 24 weeks of gestation) | 130 | 14 (9.90) | 1.00 (0.78-1.00) | 0.12 (0.07-0.19) | 0.11 (0.07-0.18) | 1.00 (0.80-1.00) | 1.10 (0.98-1.23)* | 0.28 (0.02-4.37)* | 0.56 (0.53-0.59) |
| Chaiworapongsa et al (2023), US | abnormal angiogenic profile (sFlt-1/PlGF ratio < 10th percentile for gestational age) in early-onset pre-eclampsia (cohort case-control) | fetal death | 29 | 2 (6.90) | 1.00 (0.34-1.00) | 0.11 (0.04-0.28) | 0.08 (0.02-0.24) | 1.00 (0.44-1.00) | 0.95 (0.56-1.61)* | 1.33 (0.09-20.11)* | 0.56 (0.50-0.62) |
| Chaiworapongsa et al (2023), US | abnormal angiogenic profile (sFlt-1/PlGF ratio < 10th percentile for gestational age) in intermediate pre-eclampsia (cohort case-control) | fetal death | 16 | 1 (6.25) | 1.00 (0.21-1.00) | 0.00 (0.00-0.20) | 0.06 (0.01-0.28) | NA | 0.77 (0.35-1.73)* | 8.00 (0.21-302.87)* | NA |
| Chaiworapongsa et al (2023), US | abnormal angiogenic profile (sFlt-1/PlGF ratio < 10th percentile for gestational age) in early-onset pre-eclampsia (cohort case-series) | fetal death | 89 | 5 (5.62) | 1.00 (0.57-1.00) | 0.02 (0.01-0.08) | 0.06 (0.02-0.13) | 1.00 (0.34-1.00) | 0.94 (0.74-1.21)* | 2.83 (0.15-52.60)* | 0.51 (0.50-0.53) |
| Chaiworapongsa et al (2023), US | abnormal angiogenic profile (sFlt-1/PlGF ratio < 10th percentile for gestational age) in intermediate pre-eclampsia (cohort case-series) | fetal death | 105 | 3 (2.86) | 0.33 (0.06-0.79) | 0.19 (0.12-0.27) | 0.01 (0.00-0.06) | 0.90 (0.71-0.97) | 0.41 (0.08-2.04) | 3.58 (1.46-8.78) | 0.26 (0.00-0.59) |
| Chaiworapongsa et al (2023), US | abnormal angiogenic profile (sFlt-1/PlGF ratio < 10th percentile for gestational age) in term pre-eclampsia (cohort case-series) | fetal death | 258 | 5 (1.94) | 0.60 (0.23-0.88) | 0.45 (0.39-0.52) | 0.02 (0.01-0.06) | 0.98 (0.94-1.00) | 1.10 (0.53-2.27) | 0.88 (0.30-2.60) | 0.53 (0.29-0.77) |
| Chaves et al (2017), Brazil | abnormal PR (ratio of the flow velocity of the second peak to that of the initial systolic velocity peak) ≥ 0.78 | fetal death/stillbirth | 56 | 1 (1.79) | 1.00 (0.21-1.00) | 0.13 (0.06-0.24) | 0.02 (0.00-0.11) | 1.00 (0.65-1.00) | 0.87 (0.39-1.94)* | 1.87 (0.15-22.54)* | NA |
| Chaves et al (2017), Brazil | abnormal PR (ratio of the flow velocity of the second peak to that of the initial systolic velocity peak) ≥ 0.99 | fetal death/stillbirth | 56 | 1 (1.79) | 1.00 (0.21-1.00) | 0.73 (0.60-0.83) | 0.06 (0.01-0.28) | 1.00 (0.91-1.00) | 2.71 (1.10-6.70)* | 0.35 (0.03-3.83)* | NA |
| Joshi et al (2022), India | raised serum hs-CRP > 350 µg/mL | fetal death/stillbirth | 132 | 5 (3.79) | 1.00 (0.57-1.00) | 0.57 (0.48-0.65) | 0.08 (0.04-0.18) | 1.00 (0.95-1.00) | 2.11 (1.55-2.89)* | 0.15 (0.01-2.10)* | 0.78 (0.74-0.83) |
| Kesireddy et al (2021), India | GlyFn > 350 µg/mL | fetal death/stillbirth | 51 | 1 (1.96) | 1.00 (0.21-1.00) | 0.42 (0.29-0.56) | 0.03 (0.01-0.17) | 1.00 (0.85-1.00) | 1.30 (0.56-2.98)* | 0.59 (0.05-6.68)* | NA |
| Le et al (2018), Vietnam | serum uric acid level > 376 µmol/L | fetal death/stillbirth | 205 | NA (2.90) | 0.83 | 0.57 | NA | NA | NA | NA | 0.63 |
| Lei et al (2021), China | 24 h Proteinuria excretion < 0.3 g/24h | fetal death/stillbirth | 275 | 16 (5.80) | 0.00 (0.00-0.19) | 0.80 (0.74-0.84) | 0.00 (0.00-0.07) | 0.93 (0.89-0.96) | 0.14 (0.01-2.22)* | 1.22 (1.10-1.36)* | 0.40 (0.37-0.42) |
| Lei et al (2021), China | 24 h Proteinuria excretion ≥ 0.3 g/24h | fetal death/stillbirth | 275 | 16 (5.80) | 0.75 (0.51-0.90) | 0.59 (0.53-0.65) | 0.10 (0.06-0.17) | 0.97 (0.94-0.99) | 1.85 (1.34-2.55) | 0.42 (0.18-0.99) | 0.67 (0.56-0.79) |
| Lei et al (2021), China | 24 h Proteinuria excretion ≥ 2 g/24h | fetal death/stillbirth | 275 | 16 (5.80) | 0.56 (0.33-0.77) | 0.83 (0.78-0.87) | 0.17 (0.09-0.29) | 0.97 (0.94-0.98) | 3.31 (1.99-5.51) | 0.53 (0.30-0.92) | 0.70 (0.57-0.82) |
| Lei et al (2021), China | 24 h Proteinuria excretion ≥ 3.965 g/24h | fetal death/stillbirth | 275 | NA (5.80) | 0.67 | 0.86 | NA | NA | NA | NA | 0.82 (0.722–0.910) |
| Loardi et al (2021), Italy | abnormal UtA PI > 95th percentile | fetal death/stillbirth | 311 | 3 (0.96) | 0.67 (0.21-0.94) | 0.40 (0.35-0.45) | 0.01 (0.00-0.04) | 0.99 (0.96-1.00) | 1.11 (0.50-2.48) | 0.83 (0.17-4.16) | 0.53 (0.21-0.86) |
| Stolz et al (2018), Austria | sFlt-1/PlGF ratio ≥ 655 | perinatal death | 60 | 11 (18.33) | 0.55 (0.28-0.79) | 0.51 (0.37-0.64) | 0.20 (0.10-0.37) | 0.83 (0.66-0.93) | 1.11 (0.60-2.05) | 0.89 (0.44-1.80) | 0.53 (0.36-0.70) |
| Stolz et al (2018), Austria | sFlt-1/PlGF ratio ≥ 655 | fetal death/stillbirth | 60 | 6 (10.00) | 0.50 (0.19-0.81) | 0.50 (0.37-0.63) | 0.10 (0.03-0.26) | 0.90 (0.74-0.97) | 1.00 (0.43-2.32) | 1.00 (0.43-2.32) | 0.50 (0.27-0.73) |
| Webster et al (2018), UK | Ethnicity: White | fetal death/stillbirth | 4,481 | 72 (1.61) | 0.17 (0.10-0.27) | 0.52 (0.51-0.54) | 0.01 (0.00-0.01) | 0.97 (0.97-0.98) | 0.35 (0.21-0.58) | 1.60 (1.44-1.78) | 0.34 (0.30-0.39) |
| Webster et al (2018), UK | Ethnicity: Black | fetal death/stillbirth | 4,481 | 72 (1.61) | 0.68 (0.57-0.78) | 0.65 (0.63-0.66) | 0.03 (0.02-0.04) | 0.99 (0.99-0.99) | 1.93 (1.64-2.28) | 0.49 (0.35-0.69) | 0.66 (0.61-0.72) |
| Webster et al (2018), UK | Ethnicity: Asian | fetal death/stillbirth | 4,481 | 72 (1.61) | 0.08 (0.04-0.17) | 0.92 (0.91-0.92) | 0.02 (0.01-0.03) | 0.98 (0.98-0.99) | 0.99 (0.46-2.13) | 1.00 (0.93-1.07) | 0.50 (0.47-0.53) |
| Ye et al (2020), China | retinopathy staging Stage 3/4 | fetal death/stillbirth | 534 | 82 (15.36) | 0.20 (0.12-0.29) | 0.86 (0.83-0.89) | 0.21 (0.13-0.31) | 0.86 (0.82-0.88) | 1.42 (0.87-2.34) | 0.93 (0.83-1.04) | 0.53 (0.48-0.57) |
| Jampana et al (2022), India | maternal serum uric acid level > 5.5 mg/dL | perinatal death | 86 | 21 (24.42) | 0.71 (0.50-0.86) | 0.45 (0.33-0.57) | 0.29 (0.19-0.43) | 0.83 (0.67-0.92) | 1.29 (0.91-1.83) | 0.64 (0.31-1.33) | 0.58 (0.46-0.70) |
| Kurtser et al (2019), Russia | serum PlGF | perinatal death | 28 | 5 (17.86) | NA | NA | NA | NA | NA | NA | 0.98 |
| Kurtser et al (2019), Russia | serum sFlt-1 | perinatal death | 28 | 5 (17.86) | NA | NA | NA | NA | NA | NA | 0.59 |
| Kurtser et al (2019), Russia | sFlt-1/PlGF ratio ≥ 520 | perinatal death | 28 | 5 (17.86) | 1.00 | 0.88 | NA | NA | NA | NA | 0.95 |
| Fishel Bartal et al (2022), US | Ethnicity: Non-Hispanic White | infant death | 389,347 | 2228 (0.57) | 0.38 (0.36-0.40) | 0.50 (0.50-0.50) | 0.00 (0.00-0.00) | 0.99 (0.99-0.99) | 0.75 (0.71-0.79) | 1.26 (1.22-1.30) | 0.44 (0.43-0.45) |
| Fishel Bartal et al (2022), US | Ethnicity: Non-Hispanic Black | infant death | 389,347 | 2228 (0.57) | 0.46 (0.44-0.48) | 0.70 (0.70-0.71) | 0.01 (0.01-0.01) | 1.00 (1.00-1.00) | 1.55 (1.48-1.62) | 0.77 (0.74-0.80) | 0.58 (0.57-0.59) |
| Fishel Bartal et al (2022), US | Ethnicity: Hispanic | infant death | 389,347 | 2228 (0.57) | 0.13 (0.11-0.14) | 0.84 (0.84-0.85) | 0.00 (0.00-0.01) | 0.99 (0.99-0.99) | 0.82 (0.73-0.91) | 1.03 (1.02-1.05) | 0.49 (0.48-0.49) |
| Fishel Bartal et al (2022), US | Ethnicity: Non-Hispanic Asian American, Native Hawaiian and other Pacific Islander | infant death | 389,347 | 2228 (0.57) | 0.03 (0.02-0.03) | 0.96 (0.96-0.96) | 0.00 (0.00-0.01) | 0.99 (0.99-0.99) | 0.72 (0.55-0.93) | 1.01 (1.00-1.02) | 0.49 (0.49-0.50) |
| Fishel Bartal et al (2022), US | Ethnicity: Non-Hispanic American Indian and Alaska Native | infant death | 389,347 | 2228 (0.57) | 0.01 (0.01-0.02) | 0.99 (0.99-0.99) | 0.01 (0.01-0.01) | 0.99 (0.99-0.99) | 1.33 (0.94-1.87) | 1.00 (0.99-1.00) | 0.50 (0.50-0.50) |
| Chaiworapongsa et al (2023), US | abnormal angiogenic profile (sFlt-1/PlGF ratio < 10th percentile for gestational age) in early-onset pre-eclampsia (cohort case-control) | fetal or neonatal death | 29 | 4 (13.79) | 1.00 (0.51-1.00) | 0.12 (0.04-0.30) | 0.15 (0.06-0.34) | 1.00 (0.44-1.00) | 1.04 (0.75-1.45)* | 0.74 (0.04-12.27)* | 0.56 (0.49-0.63) |
| Chaiworapongsa et al (2023), US | abnormal angiogenic profile (sFlt-1/PlGF ratio < 10th percentile for gestational age) in intermediate pre-eclampsia (cohort case-control) | fetal or neonatal death | 16 | 1 (6.25) | 1.00 (0.21-1.00) | 0.00 (0.00-0.20) | 0.06 (0.01-0.28) | NA | 0.77 (0.35-1.73)* | 8.00 (0.21-302.87)* | NA |
| Chaiworapongsa et al (2023), US | abnormal angiogenic profile (sFlt-1/PlGF ratio < 10th percentile for gestational age) in early-onset pre-eclampsia (cohort case-series) | fetal or neonatal death | 89 | 7 (7.87) | 1.00 (0.65-1.00) | 0.02 (0.01-0.08) | 0.08 (0.04-0.16) | 1.00 (0.34-1.00) | 0.97 (0.81-1.16)* | 2.08 (0.11-39.58)* | 0.51 (0.50-0.53) |
| Chaiworapongsa et al (2023), US | abnormal angiogenic profile (sFlt-1/PlGF ratio < 10th percentile for gestational age) in intermediate pre-eclampsia (cohort case-series) | fetal or neonatal death | 105 | 4 (3.81) | 0.50 (0.15-0.85) | 0.19 (0.12-0.28) | 0.02 (0.01-0.08) | 0.90 (0.71-0.97) | 0.62 (0.23-1.65) | 2.66 (0.92-7.67) | 0.34 (0.06-0.63) |
| Chaiworapongsa et al (2023), US | abnormal angiogenic profile (sFlt-1/PlGF ratio < 10th percentile for gestational age) in term pre-eclampsia (cohort case-series) | fetal or neonatal death | 258 | 5 (1.94) | 0.60 (0.23-0.88) | 0.45 (0.39-0.52) | 0.02 (0.01-0.06) | 0.98 (0.94-1.00) | 1.10 (0.53-2.27) | 0.88 (0.30-2.60) | 0.53 (0.29-0.77) |
| Binder et al (2021), Austria | angiogenic imbalance (sFlt-1/PlGF ratio above the 97th percentile and/or PlGF levels below 2.5th centile) without proteinuria | stillbirth or early neonatal death | 145 | 4 (2.80) | 0.25 (0.05-0.70) | 0.72 (0.64-0.79) | 0.02 (0.00-0.13) | 0.97 (0.92-0.99) | 0.90 (0.16-5.04) | 1.04 (0.58-1.84) | 0.49 (0.24-0.73) |
| Binder et al (2021), Austria | angiogenic imbalance (sFlt-1/PlGF ratio above the 97th percentile and/or PlGF levels below 2.5th centile) without IUGR | stillbirth or early neonatal death | 145 | 4 (2.80) | 0.00 (0.00-0.49) | 0.74 (0.67-0.81) | 0.00 (0.00-0.10) | 0.96 (0.91-0.99) | 0.39 (0.03-5.48)* | 1.21 (0.89-1.65)* | 0.37 (0.34-0.41) |
| Binder et al (2021), Austria | angiogenic imbalance (sFlt-1/PlGF ratio above the 97th percentile and/or PlGF levels below 2.5th centile) and/or proteinuria | stillbirth or early neonatal death | 145 | 4 (2.80) | 0.75 (0.30-0.95) | 0.62 (0.53-0.69) | 0.05 (0.02-0.14) | 0.99 (0.94-1.00) | 1.96 (1.07-3.58) | 0.41 (0.07-2.22) | 0.68 (0.44-0.93) |
| Binder et al (2021), Austria | proteinuria, other symptoms or IUGR without angiogenic imbalance | stillbirth or early neonatal death | 145 | 4 (2.80) | 0.25 (0.05-0.70) | 0.92 (0.87-0.96) | 0.08 (0.01-0.35) | 0.98 (0.94-0.99) | 3.20 (0.54-19.19) | 0.81 (0.46-1.44) | 0.59 (0.34-0.83) |
| Morikawa et al (2020), Japan | serum total protein level ≤ 63 g/L | stillbirth or early neonatal death | 94 | 7 (7.50) | 0.43 (0.16-0.75) | 0.89 (0.81-0.94) | 0.23 (0.09-0.53) | 0.95 (0.88-0.98) | 4.05 (1.41-11.63) | 0.64 (0.34-1.22) | 0.66 (0.46-0.86) |
| Morikawa et al (2020), Japan | serum total protein level ≤ 57 g/L | stillbirth or early neonatal death | 94 | 7 (7.50) | 0.43 (0.16-0.75) | 0.71 (0.61-0.79) | 0.11 (0.04-0.26) | 0.94 (0.86-0.98) | 1.47 (0.59-3.66) | 0.81 (0.42-1.55) | 0.57 (0.36-0.77) |
| Moriakawa et al (2021), Japan | gestational weight gain during the week prior delivery ≥ 1.6 kg | stillbirth or early neonatal death | 94 | 7 (7.50) | 0.71 (0.36-0.92) | 0.52 (0.41-0.62) | 0.11 (0.05-0.23) | 0.96 (0.86-0.99) | 1.48 (0.88-2.48) | 0.55 (0.17-1.81) | 0.62 (0.43-0.80) |
| Moriakawa et al (2021), Japan | gestational weight gain during the week prior delivery ≥ 2.8 kg | stillbirth or early neonatal death | 94 | 7 (7.50) | 0.57 (0.25-0.84) | 0.78 (0.69-0.85) | 0.17 (0.07-0.36) | 0.96 (0.89-0.99) | 2.63 (1.24-5.56) | 0.55 (0.23-1.30) | 0.68 (0.47-0.88) |
| Abraham et al (2019), India | TOH ≤ 239 days | intrauterine fetal demise or perinatal death | 174 | NA (16.09) | 0.77 | 0.67 | NA | NA | NA | NA | 0.77 (0.666–0.866) |
| Abraham et al (2019), India | ADMA ≥ 0.958 µmol/L | intrauterine fetal demise or perinatal death | 174 | NA (16.09) | 0.76 | 0.61 | NA | NA | NA | NA | 0.75 (0.652–0.851) |
| Abraham et al (2019), India | TOH (days) + ADMA (µmol/L) | intrauterine fetal demise or perinatal death | 174 | NA (16.09) | 0.82 | 0.72 | NA | NA | NA | NA | 0.81(0.700–0.922) |
| Heimberger et al (2020), US | sFlt-1/PlGF ratio ≥ 85 | intrauterine fetal demise or perinatal death | 115 | 2 (1.74) | 0.50 (0.09-0.91) | 0.68 (0.59-0.76) | 0.03 (0.00-0.14) | 0.99 (0.93-1.00) | 1.57 (0.38-6.44) | 0.73 (0.18-2.95) | 0.59 (0.10-1.00) |
| Leanos-Miranda et el (2020), Mexico | sFlt-1/PlGF ratio ≥ 38 | intrauterine fetal demise or perinatal death | 810 | 118 (14.57) | 0.97 (0.92-0.99) | 0.40 (0.37-0.44) | 0.22 (0.18-0.25) | 0.99 (0.96-0.99) | 1.62 (1.51-1.74) | 0.08 (0.03-0.22) | 0.69 (0.66-0.71) |
| Leanos-Miranda et el (2020), Mexico | sFlt-1/PlGF ratio ≥ 85 | intrauterine fetal demise or perinatal death | 810 | 118 (14.57) | 0.97 (0.92-0.99) | 0.55 (0.51-0.59) | 0.27 (0.23-0.31) | 0.99 (0.97-1.00) | 2.16 (1.97-2.36) | 0.06 (0.02-0.16) | 0.76 (0.73-0.78) |
| Malik et al (2023), India | CPR < 1.08 | stillbirth/ perinatal death | 100 | 4 (4.00) | 1.00 (0.51-1.00) | 0.71 (0.61-0.79) | 0.12 (0.05-0.28) | 1.00 (0.95-1.00) | 3.06 (2.00-4.68)* | 0.14 (0.01-1.97)* | 0.85 (0.81-0.90) |
| Zarean et al (2022), Iran | CPR < 1 | intrauterine fetal demise or perinatal death | 100 | 1 (1.00) | 1.00 (0.21-1.00) | 0.74 (0.64-0.81) | 0.04 (0.01-0.18) | 1.00 (0.95-1.00) | 2.83 (1.19-6.72)* | 0.34 (0.03-3.76)* | NA |
| Sudjai et al (2022), Thailand | serum uric acid level < 5.0 mg/dL | admission and neonatal death | 400 | 8 (2.00) | 0.00 (0.00-0.32) | 0.76 (0.71-0.80) | 0.00 (0.00-0.04) | 0.97 (0.95-0.99) | 0.23 (0.02-3.36)* | 1.25 (1.06-1.48)* | 0.38 (0.36-0.40) |
| Sudjai et al (2022), Thailand | serum uric acid level ≥ 5.0 mg/dL | admission and neonatal death | 400 | 8 (2.00) | 1.00 (0.68-1.00) | 0.24 (0.20-0.29) | 0.03 (0.01-0.05) | 1.00 (0.96-1.00) | 1.25 (1.06-1.48)* | 0.23 (0.02-3.36)* | 0.62 (0.60-0.64) |
| Sudjai et al (2022), Thailand | serum uric acid level ≥ 7.0 mg/dL | admission and neonatal death | 400 | 8 (2.00) | 0.62 (0.31-0.86) | 0.70 (0.65-0.74) | 0.04 (0.02-0.09) | 0.99 (0.97-1.00) | 2.06 (1.18-3.59) | 0.54 (0.22-1.32) | 0.66 (0.48-0.84) |
| **NICU admission** | | | | | | | | | | | |
| Alanwar et al (2018), Egypt | CPR < 1 or below 5th percentile | NICU admission | 100 | 34 (34.00) | 0.62 (0.17-0.46) | 0.71 (0.82-0.96) | 0.29 (0.39-0.82) | 0.91 (0.61-0.80) | 3.24 (1.28-8.15) | 0.78 (0.62-0.98) | 0.60 (0.52-0.69) |
| Binder et al (2021), Austria | angiogenic imbalance (sFlt-1/PlGF ratio above the 97th percentile and/or PlGF levels below 2.5th centile) without proteinuria | NICU admission | 145 | 29 (20.00) | 0.55 (0.38-0.72) | 0.79 (0.71-0.86) | 0.40 (0.26-0.55) | 0.88 (0.80-0.93) | 2.67 (1.64-4.33) | 0.57 (0.37-0.86) | 0.67 (0.57-0.77) |
| Binder et al (2021), Austria | angiogenic imbalance (sFlt-1/PlGF ratio above the 97th percentile and/or PlGF levels below 2.5th centile) without IUGR | NICU admission | 145 | 29 (20.00) | 0.41 (0.26-0.59) | 0.79 (0.71-0.86) | 0.33 (0.20-0.50) | 0.84 (0.76-0.90) | 2.00 (1.14-3.50) | 0.74 (0.54-1.02) | 0.60 (0.51-0.70) |
| Binder et al (2021), Austria | angiogenic imbalance (sFlt-1/PlGF ratio above the 97th percentile and/or PlGF levels below 2.5th centile) and/or proteinuria | NICU admission | 145 | 29 (20.00) | 0.83 (0.65-0.92) | 0.72 (0.63-0.79) | 0.42 (0.30-0.55) | 0.94 (0.87-0.98) | 2.91 (2.09-4.06) | 0.24 (0.11-0.54) | 0.77 (0.69-0.85) |
| Binder et al (2021), Austria | proteinuria, other symptoms or IUGR without angiogenic imbalance | NICU admission | 145 | 29 (20.00) | 0.03 (0.01-0.17) | 0.91 (0.84-0.95) | 0.08 (0.01-0.35) | 0.79 (0.71-0.85) | 0.36 (0.05-2.70) | 1.07 (0.97-1.17) | 0.47 (0.43-0.51) |
| Carter et al (2017), US | SGA (birthweight less than 10%) | NICU admission (greater than 12 h) | 902 | 149 (16.52) | 0.10 (0.06-0.16) | 0.81 (0.78-0.84) | 0.10 (0.06-0.15) | 0.82 (0.79-0.85) | 0.54 (0.33-0.89) | 1.11 (1.04-1.18) | 0.46 (0.43-0.48) |
| Chadha et al (2022), India | UPCR ≥ 0.3 | NICU admission | 130 | 70 (49.60) | 0.97 (0.90-0.99) | 0.18 (0.11-0.29) | 0.54 (0.45-0.62) | 0.87 (0.62-0.96) | 1.19 (1.06-1.34) | 0.16 (0.04-0.67) | 0.58 (0.53-0.63) |
| Chaves et al (2017), Brazil | abnormal PR (ratio of the flow velocity of the second peak to that of the initial systolic velocity peak) ≥ 0.78 | NICU admission | 56 | 40 (71.43) | 0.90 (0.77-0.96) | 0.19 (0.07-0.43) | 0.73 (0.60-0.84) | 0.43 (0.16-0.75) | 1.11 (0.86-1.43) | 0.53 (0.13-2.12) | 0.54 (0.43-0.65) |
| Chaves et al (2017), Brazil | abnormal PR (ratio of the flow velocity of the second peak to that of the initial systolic velocity peak) ≥ 0.99 | NICU admission | 56 | 3 (5.36) | 0.67 (0.21-0.94) | 0.11 (0.05-0.23) | 0.04 (0.01-0.14) | 0.86 (0.49-0.97) | 0.75 (0.34-1.68) | 2.94 (0.50-17.27) | 0.39 (0.06-0.72) |
| Chaves et al (2017), Brazil | abnormal PR (ratio of the flow velocity of the second peak to that of the initial systolic velocity peak) ≥ 0.78 | NICU admission > 2500g | 56 | 40 (71.43) | 0.32 (0.20-0.48) | 0.81 (0.57-0.93) | 0.81 (0.57-0.93) | 0.32 (0.20-0.48) | 1.73 (0.57-5.28) | 0.83 (0.60-1.14) | 0.57 (0.45-0.69) |
| Chaves et al (2017), Brazil | abnormal PR (ratio of the flow velocity of the second peak to that of the initial systolic velocity peak) ≥ 0.99 | NICU admission > 2500g | 56 | 3 (5.36) | 0.00 (0.00-0.56) | 0.70 (0.56-0.80) | 0.00 (0.00-0.19) | 0.92 (0.80-0.97) | 0.41 (0.03-5.64)* | 1.26 (0.84-1.90)* | 0.35 (0.29-0.41) |
| Graupner et al (2019), Germany | sFlt-1/PlGF ratio ≥ 110 | NICU admission | 67 | 8 (11.90) | 0.62 (0.31-0.86) | 0.63 (0.50-0.74) | 0.19 (0.08-0.37) | 0.92 (0.80-0.97) | 1.68 (0.89-3.15) | 0.60 (0.24-1.49) | 0.63 (0.44-0.82) |
| Graupner et al (2019), Germany | CPR < 5th centile | NICU admission | 67 | 8 (11.90) | 0.00 (0.00-0.32) | 0.88 (0.77-0.94) | 0.00 (0.00-0.35) | 0.87 (0.76-0.93) | 0.44 (0.03-7.13)* | 1.08 (0.90-1.30)* | 0.44 (0.40-0.48) |
| Graupner et al (2019), Germany | mean UtA-PI > 95th centile | NICU admission | 67 | 8 (11.90) | 0.38 (0.14-0.69) | 0.66 (0.53-0.77) | 0.13 (0.05-0.32) | 0.89 (0.76-0.95) | 1.11 (0.42-2.90) | 0.95 (0.54-1.67) | 0.52 (0.33-0.71) |
| Heimberger et al (2020), US | sFlt-1/PlGF ratio ≥ 85 | NICU admission | 115 | 54 (50.00) | 0.43 (0.30-0.56) | 0.77 (0.65-0.86) | 0.62 (0.46-0.76) | 0.60 (0.49-0.70) | 1.86 (1.07-3.23) | 0.75 (0.57-0.97) | 0.60 (0.51-0.68) |
| Karge et al (2021), Germany | early-onset pre-eclampsia | NICU admission | 49 | 19 (38.78) | 0.68 (0.46-0.85) | 0.83 (0.66-0.93) | 0.72 (0.49-0.88) | 0.81 (0.64-0.91) | 4.11 (1.74-9.67) | 0.38 (0.19-0.75) | 0.76 (0.63-0.89) |
| Karge et al (2021), Germany | late-onset pre-eclampsia | NICU admission | 49 | 19 (38.78) | 0.32 (0.15-0.54) | 0.17 (0.07-0.34) | 0.19 (0.09-0.36) | 0.28 (0.12-0.51) | 0.38 (0.19-0.75) | 4.11 (1.74-9.67) | 0.24 (0.11-0.37) |
| Karge et al (2022), Germany | BMI ≥ 25 kg/m^2 | NICU admission | 141 | 55 (39.01) | 0.38 (0.27-0.51) | 0.58 (0.48-0.68) | 0.37 (0.26-0.50) | 0.60 (0.49-0.69) | 0.91 (0.60-1.39) | 1.06 (0.81-1.40) | 0.48 (0.40-0.56) |
| Karge et al (2022), Germany | BMI ≥ 30 kg/m^2 | NICU admission | 141 | 55 (39.01) | 0.20 (0.12-0.32) | 0.80 (0.71-0.87) | 0.39 (0.24-0.58) | 0.61 (0.52-0.70) | 1.01 (0.51-1.99) | 1.00 (0.84-1.18) | 0.50 (0.43-0.57) |
| Joshi et al (2022), India | raised serum hs-CRP > 3.0 mg/mL | NICU admission | 132 | 27 (20.45) | 0.96 (0.82-0.99) | 0.68 (0.58-0.76) | 0.43 (0.32-0.56) | 0.99 (0.93-1.00) | 2.97 (2.23-3.96) | 0.05 (0.01-0.38) | 0.82 (0.76-0.88) |
| Leanos-Miranda et el (2020), Mexico | sFlt-1/PlGF ratio ≥ 38 | NICU admission | 810 | 363 (44.81) | 0.81 (0.77-0.85) | 0.48 (0.44-0.53) | 0.56 (0.52-0.60) | 0.76 (0.70-0.80) | 1.56 (1.41-1.73) | 0.40 (0.31-0.50) | 0.65 (0.61-0.68) |
| Leanos-Miranda et el (2020), Mexico | sFlt-1/PlGF ratio ≥ 85 | NICU admission | 810 | 363 (44.81) | 0.70 (0.65-0.75) | 0.62 (0.58-0.67) | 0.60 (0.55-0.65) | 0.72 (0.67-0.76) | 1.86 (1.62-2.13) | 0.48 (0.40-0.57) | 0.66 (0.63-0.69) |
| Madhu et al (2023), India | increase in UA S/D ratio | NICU admission | 150 | 55 (36.67) | 0.33 (0.22-0.46) | 0.83 (0.74-0.89) | 0.53 (0.37-0.69) | 0.68 (0.59-0.76) | 1.94 (1.08-3.49) | 0.81 (0.66-0.99) | 0.58 (0.51-0.65) |
| Madhu et al (2023), India | elevated UA RI | NICU admission | 150 | 55 (36.67) | 0.18 (0.10-0.30) | 0.88 (0.80-0.93) | 0.48 (0.28-0.68) | 0.65 (0.57-0.73) | 1.57 (0.71-3.46) | 0.93 (0.80-1.07) | 0.53 (0.47-0.59) |
| Madhu et al (2023), India | elevated UA PI | NICU admission | 150 | 55 (36.67) | 0.25 (0.16-0.38) | 0.82 (0.73-0.89) | 0.45 (0.29-0.62) | 0.66 (0.57-0.73) | 1.42 (0.76-2.66) | 0.91 (0.76-1.09) | 0.54 (0.47-0.61) |
| Madhu et al (2023), India | elevated MCA PI | NICU admission | 150 | 55 (36.67) | 0.07 (0.03-0.17) | 0.93 (0.86-0.96) | 0.36 (0.15-0.65) | 0.63 (0.55-0.71) | 0.99 (0.30-3.22) | 1.00 (0.91-1.10) | 0.50 (0.46-0.54) |
| Madhu et al (2023), India | abnormal CPR <1.08 | NICU admission | 150 | 55 (36.67) | 0.64 (0.50-0.75) | 0.31 (0.22-0.40) | 0.35 (0.26-0.44) | 0.59 (0.45-0.72) | 0.92 (0.72-1.16) | 1.19 (0.75-1.89) | 0.47 (0.39-0.55) |
| Madhu et al (2023), India | UA reversed end diastolic flow | NICU admission | 150 | 55 (36.67) | 0.00 (0.00-0.07) | 0.97 (0.91-0.99) | 0.00 (0.00-0.56) | 0.63 (0.55-0.70) | 0.24 (0.01-4.65)* | 1.03 (0.98-1.08)* | 0.48 (0.47-0.50) |
| Malik et al (2023), India | CPR < 1.08 | NICU admission | 100 | 28 (28.00) | 0.79 (0.60-0.90) | 0.86 (0.76-0.92) | 0.69 (0.51-0.82) | 0.91 (0.82-0.96) | 5.66 (3.08-10.38) | 0.25 (0.12-0.51) | 0.82 (0.74-0.91) |
| Mayama et al (2021), Japan | mild thrombocytopenia platelet < 150x10^9/L | NICU admission | 264 | 151 (57.20) | 0.33 (0.26-0.41) | 0.78 (0.69-0.85) | 0.67 (0.55-0.76) | 0.47 (0.40-0.54) | 1.50 (0.99-2.26) | 0.86 (0.74-1.00) | 0.55 (0.50-0.61) |
| Mayama et al (2021), Japan | severe thrombocytopenia platelet < 100x10^9/L | NICU admission | 264 | 151 (57.20) | 0.14 (0.09-0.20) | 0.97 (0.92-0.99) | 0.88 (0.69-0.96) | 0.46 (0.40-0.52) | 5.24 (1.60-17.13) | 0.88 (0.82-0.95) | 0.56 (0.52-0.59) |
| Moawad et al (2022), Egypt | UA PI > 95th percentile | NICU admission | 60 | 15 (25.00) | 0.60 (0.36-0.80) | 0.69 (0.54-0.80) | 0.39 (0.22-0.59) | 0.84 (0.69-0.92) | 1.93 (1.06-3.51) | 0.58 (0.30-1.11) | 0.64 (0.50-0.79) |
| Moawad et al (2022), Egypt | UA RI > 95th percentile | NICU admission | 60 | 15 (25.00) | 0.60 (0.36-0.80) | 0.64 (0.50-0.77) | 0.36 (0.20-0.55) | 0.83 (0.67-0.92) | 1.69 (0.95-2.99) | 0.62 (0.32-1.20) | 0.62 (0.48-0.77) |
| Moawad et al (2022), Egypt | MCA PI < 5th percentile | NICU admission | 60 | 15 (25.00) | 0.32 (0.15-0.58) | 0.61 (0.44-0.76) | 0.27 (0.13-0.53) | 0.66 (0.47-0.80) | 0.86 (0.37-2.00) | 1.09 (0.69-1.71) | 0.47 (0.32-0.62) |
| Moawad et al (2022), Egypt | MCA RI < 5th percentile | NICU admission | 60 | 15 (25.00) | 0.14 (0.04-0.38) | 0.47 (0.19-0.81) | 0.40 (0.12-0.77) | 0.18 (0.07-0.43) | 0.27 (0.06-1.22) | 1.73 (0.76-3.95) | 0.32 (0.08-0.55) |
| Moawad et al (2022), Egypt | CPR < 1 | NICU admission | 60 | 15 (25.00) | 0.73 (0.48-0.89) | 0.56 (0.41-0.69) | 0.36 (0.21-0.53) | 0.86 (0.69-0.95) | 1.65 (1.06-2.58) | 0.48 (0.20-1.16) | 0.64 (0.51-0.78) |
| Morikawa et al (2021), Japan | gestational weight gain during the week prior delivery ≥ 1.6 kg | NICU admission | 94 | 42 (44.70) | 0.74 (0.59-0.85) | 0.69 (0.56-0.80) | 0.66 (0.52-0.78) | 0.77 (0.63-0.86) | 2.40 (1.54-3.75) | 0.38 (0.22-0.65) | 0.72 (0.62-0.81) |
| Nayak et al (2022), India | CPR < 1 | NICU admission | 62 | 17 (27.42) | 0.93 (0.70-NA) | 0.74 (0.60-0.85) | 0.54 (0.35-0.71) | 0.97 (0.86-1.00) | 3.69 (2.23-6.09) | 0.08 (0.01-0.53) | 0.84 (0.76-0.93) |
| Nayak et al (2022), India | NST | NICU admission | 62 | 17 (27.42) | 0.47 (0.24-0.70) | 0.66 (0.52-0.78) | 0.30 (0.16-0.51) | 0.79 (0.64-0.89) | 1.39 (0.74-2.60) | 0.80 (0.49-1.31) | 0.57 (0.43-0.70) |
| Saxena et al (2021), India | CPR < 1 | NICU admission | 150 | 50 (33.33) | 0.83 (0.65-0.94) | 0.79 (0.71-0.86) | 0.50 (0.36-0.64) | 0.95 (0.89-0.98) | 4.03 (2.98-5.45) | 0.20 (0.11-0.38) | 0.82 (0.76-0.87) |
| Stolz et al (2018), Austria | sFlt-1/PlGF ratio ≥ 655 | NICU admission | 54 | 51 (85.00) | 0.49 (0.36-0.62) | 0.33 (0.06-0.79) | 0.93 (0.77-0.98) | 0.04 (0.01-0.18) | 0.74 (0.32-1.72) | 1.53 (0.30-7.75) | 0.41 (0.08-0.75) |
| Sudjai et al (2022), Thailand | serum uric acid level < 5.0 mg/dL | NICU admission | 400 | 48 (12.00) | 0.15 (0.07-0.27) | 0.75 (0.70-0.79) | 0.07 (0.04-0.14) | 0.87 (0.82-0.90) | 0.58 (0.28-1.17) | 1.14 (1.00-1.30) | 0.45 (0.39-0.50) |
| Sudjai et al (2022), Thailand | serum uric acid level ≥ 5.0 mg/dL | NICU admission | 400 | 48 (12.00) | 0.85 (0.73-0.93) | 0.25 (0.21-0.30) | 0.13 (0.10-0.18) | 0.93 (0.86-0.96) | 1.14 (1.00-1.30) | 0.58 (0.28-1.17) | 0.55 (0.50-0.61) |
| Sudjai et al (2022), Thailand | serum uric acid level ≥ 7.0 mg/dL | NICU admission | 400 | 48 (12.00) | 0.50 (0.36-0.64) | 0.72 (0.67-0.76) | 0.19 (0.13-0.27) | 0.91 (0.87-0.94) | 1.76 (1.27-2.44) | 0.70 (0.52-0.93) | 0.61 (0.53-0.68) |
| Tokalioglu et al (2023), Turkey | umbilical artery half peak systolic velocity deceleration time (UA hPSV-DT) <5th percentile | NICU admission | 55 | 23 (41.82) | 0.70 (0.49-0.84) | 0.81 (0.65-0.91) | 0.73 (0.52-0.87) | 0.79 (0.62-0.89) | 3.71 (1.72-8.01) | 0.37 (0.20-0.71) | 0.75 (0.64-0.87) |
| Webster et al (2018), UK | Ethnicity: White | NICU admission | 4,481 | 413 (9.22) | 0.38 (0.33-0.43) | 0.52 (0.50-0.53) | 0.07 (0.06-0.09) | 0.89 (0.88-0.90) | 0.78 (0.69-0.89) | 1.20 (1.11-1.31) | 0.45 (0.42-0.47) |
| Webster et al (2018), UK | Ethnicity: Black | NICU admission | 4,481 | 413 (9.22) | 0.43 (0.38-0.47) | 0.65 (0.63-0.66) | 0.11 (0.10-0.13) | 0.92 (0.91-0.93) | 1.22 (1.08-1.37) | 0.88 (0.81-0.96) | 0.54 (0.51-0.56) |
| Webster et al (2018), UK | Ethnicity: Asian | NICU admission | 4,481 | 413 (9.22) | 0.10 (0.07-0.13) | 0.92 (0.91-0.92) | 0.11 (0.08-0.14) | 0.91 (0.90-0.92) | 1.16 (0.85-1.59) | 0.99 (0.95-1.02) | 0.51 (0.49-0.52) |
| Zarean et al (2022), Iran | CPR < 1 | NICU admission | 100 | 37 (37.00) | 0.37 (0.15-0.43) | 0.63 (0.61-0.82) | 0.27 (0.22-0.56) | 0.73 (0.52-0.73) | 1.00 (0.51-1.95) | 1.00 (0.78-1.28) | 0.50 (0.41-0.59) |
| **Poor neonatal outcomes** | | | | | | | | | | | |
| Tousty et al (2022), Poland | gestational age at delivery ≤ 32 weeks | PDA | 77 | 5 (6.49) | 1.00 (0.57-1.00) | 0.79 (0.68-0.87) | 0.25 (0.11-0.47) | 1.00 (0.94-1.00) | 4.32 (2.61-7.14)* | 0.11 (0.01-1.51)* | 0.90 (0.85-0.94) |
| Tousty et al (2022), Poland | sFlt-1/PlGF ratio ≥ 204 | PDA | 77 | 5 (6.49) | 0.60 (0.23-0.88) | 0.67 (0.55-0.76) | 0.11 (0.04-0.28) | 0.96 (0.87-0.99) | 1.80 (0.82-3.95) | 0.60 (0.20-1.78) | 0.63 (0.39-0.88) |
| Tousty et al (2022), Poland | gestational age at delivery ≤ 32 weeks | NEC | 77 | 1 (1.30) | 1.00 (0.21-1.00) | 0.75 (0.64-0.83) | 0.05 (0.01-0.24) | 1.00 (0.94-1.00) | 2.96 (1.22-7.19)* | 0.33 (0.03-3.71)* | NA |
| Tousty et al (2022), Poland | sFlt-1/PlGF ratio ≥ 204 | NEC | 77 | 1 (1.30) | 1.00 (0.21-1.00) | 0.66 (0.55-0.75) | 0.04 (0.01-0.18) | 1.00 (0.93-1.00) | 2.18 (0.92-5.14)* | 0.38 (0.03-4.23)* | NA |
| Loardi et al (2021), Italy | abnormal UtA PI > 95th percentile | NEC | 311 | 1 (0.32) | 1.00 (0.21-1.00) | 1.00 (0.35-0.46) | 0.01 (0.00-0.03) | 1.00 (0.97-1.00) | 1.25 (0.56-2.80)* | 0.62 (0.06-6.91)* | NA |
| Binder et al (2021), Austria | angiogenic imbalance (sFlt-1/PlGF ratio above the 97th percentile and/or PlGF levels below 2.5th centile) without proteinuria | IVH | 145 | 1 (0.70) | 1.00 (0.21-1.00) | 0.73 (0.65-0.80) | 0.02 (0.00-0.13) | 1.00 (0.96-1.00) | 2.75 (1.18-6.40)* | 0.34 (0.03-3.80)* | NA |
| Binder et al (2021), Austria | angiogenic imbalance (sFlt-1/PlGF ratio above the 97th percentile and/or PlGF levels below 2.5th centile) without IUGR | IVH | 145 | 1 (0.70) | 0.00 (0.00-0.79) | 0.75 (0.67-0.81) | 0.00 (0.00-0.10) | 0.99 (0.95-1.00) | 0.99 (0.09-11.13)* | 1.00 (0.45-2.24)* | NA |
| Binder et al (2021), Austria | angiogenic imbalance (sFlt-1/PlGF ratio above the 97th percentile and/or PlGF levels below 2.5th centile) and/or proteinuria | IVH | 145 | 1 (0.70) | 1.00 (0.21-1.00) | 0.61 (0.53-0.69) | 0.02 (0.00-0.09) | 1.00 (0.96-1.00) | 1.92 (0.84-4.40)* | 0.41 (0.04-4.53)* | NA |
| Binder et al (2021), Austria | proteinuria, other symptoms or IUGR without angiogenic imbalance | IVH | 145 | 1 (0.70) | 0.00 (0.00-0.79) | 0.92 (0.86-0.95) | 0.00 (0.00-0.24) | 0.99 (0.96-1.00) | 2.90 (0.25-33.88)* | 0.82 (0.37-1.83)* | NA |
| Govender et al (2022), South Africa | ductus venosus Doppler abnormal | IVH | 61 | 4 (6.56) | 1.00 (0.51-1.00) | 0.51 (0.38-0.63) | 0.12 (0.05-0.28) | 1.00 (0.88-1.00) | 1.83 (1.24-2.71)* | 0.20 (0.01-2.76)* | 0.75 (0.69-0.82) |
| Leanos-Miranda et el (2020), Mexico | sFlt-1/PlGF ratio ≥ 38 | IVH | 810 | 94 (11.60) | 0.96 (0.90-0.98) | 0.39 (0.36-0.43) | 0.17 (0.14-0.21) | 0.99 (0.96-0.99) | 1.57 (1.46-1.69) | 0.11 (0.04-0.29) | 0.67 (0.65-0.70) |
| Leanos-Miranda et el (2020), Mexico | sFlt-1/PlGF ratio ≥ 85 | IVH | 810 | 94 (11.60) | 0.95 (0.88-0.98) | 0.53 (0.50-0.57) | 0.21 (0.17-0.25) | 0.99 (0.97-0.99) | 2.02 (1.85-2.22) | 0.10 (0.04-0.24) | 0.74 (0.71-0.77) |
| Loardi et al (2021), Italy | abnormal UtA PI > 95th percentile | IVH | 311 | 9 (2.89) | 1.00 (0.70-1.00) | 0.41 (0.36-0.47) | 0.05 (0.03-0.09) | 1.00 (0.97-1.00) | 1.61 (1.36-1.91)* | 0.12 (0.01-1.82)* | 0.71 (0.68-0.73) |
| Tousty et al (2022), Poland | gestational age at delivery ≤ 32 weeks | IVH | 77 | 6 (7.79) | 1.00 (0.61-1.00) | 0.80 (0.70-0.88) | 0.30 (0.15-0.52) | 1.00 (0.94-1.00) | 4.61 (2.79-7.63)* | 0.09 (0.01-1.30)* | 0.90 (0.85-0.95) |
| Tousty et al (2022), Poland | sFlt-1/PlGF ratio ≥ 204 | IVH | 77 | 6 (7.79) | 0.67 (0.30-0.90) | 0.68 (0.56-0.77) | 0.15 (0.06-0.32) | 0.96 (0.87-0.99) | 2.06 (1.07-3.97) | 0.49 (0.16-1.55) | 0.67 (0.46-0.89) |
| Tokalioglu et al (2023), Turkey | umbilical artery half peak systolic velocity deceleration time (UA hPSV-DT) < 5th percentile | intercranial haemorrhage | 55 | 1 (1.82) | 1.00 (0.21-1.00) | 0.61 (0.48-0.73) | 0.05 (0.01-0.22) | 1.00 (0.90-1.00) | 1.92 (0.81-4.56)* | 0.41 (0.04-4.57)* | NA |
| Tousty et al (2022), Poland | gestational age at delivery ≤ 32 weeks | ROP | 77 | 3 (3.90) | 1.00 (0.44-1.00) | 0.77 (0.66-0.85) | 0.15 (0.05-0.36) | 1.00 (0.94-1.00) | 3.75 (2.16-6.52)* | 0.16 (0.01-2.19)* | 0.89 (0.84-0.93) |
| Tousty et al (2022), Poland | sFlt-1/PlGF ratio ≥ 204 | ROP | 77 | 3 (3.90) | 0.67 (0.21-0.94) | 0.66 (0.55-0.76) | 0.07 (0.02-0.23) | 0.98 (0.90-1.00) | 1.97 (0.83-4.67) | 0.50 (0.10-2.51) | 0.66 (0.33-1.00) |
| Fishel Bartal et al (2022), US | Ethnicity: Non-Hispanic White | neonatal seizures | 389,131 | 251 (0.06) | 0.53 (0.47-0.59) | 0.50 (0.50-0.50) | 0.00 (0.00-0.00) | 1.00 (1.00-1.00) | 1.06 (0.95-1.19) | 0.94 (0.82-1.07) | 0.52 (0.49-0.55) |
| Fishel Bartal et al (2022), US | Ethnicity: Non-Hispanic Black | neonatal seizures | 389,131 | 251 (0.06) | 0.27 (0.22-0.33) | 0.70 (0.70-0.70) | 0.00 (0.00-0.00) | 1.00 (1.00-1.00) | 0.93 (0.76-1.13) | 1.03 (0.96-1.11) | 0.49 (0.46-0.52) |
| Fishel Bartal et al (2022), US | Ethnicity: Hispanic | neonatal seizures | 389,131 | 251 (0.06) | 0.14 (0.11-0.19) | 0.85 (0.84-0.85) | 0.00 (0.00-0.00) | 1.00 (1.00-1.00) | 0.93 (0.68-1.25) | 1.01 (0.96-1.07) | 0.49 (0.47-0.52) |
| Fishel Bartal et al (2022), US | Ethnicity: Non-Hispanic Asian American, Native Hawaiian and other Pacific Islander | neonatal seizures | 389,131 | 251 (0.06) | 0.02 (0.01-0.05) | 0.96 (0.96-0.96) | 0.00 (0.00-0.00) | 1.00 (1.00-1.00) | 0.67 (0.30-1.48) | 1.01 (0.99-1.03) | 0.49 (0.48-0.50) |
| Fishel Bartal et al (2022), US | Ethnicity: Non-Hispanic American Indian and Alaska Native | neonatal seizures | 389,131 | 251 (0.06) | 0.02 (0.01-0.05) | 0.99 (0.99-0.99) | 0.00 (0.00-0.00) | 1.00 (1.00-1.00) | 2.21 (1.00-4.88) | 0.99 (0.97-1.01) | 0.51 (0.50-0.52) |
| Govender et al (2022), South Africa | ductus venosus Doppler abnormal | neonatal seizures | 61 | 9 (14.75) | 1.00 (0.70-1.00) | 0.56 (0.42-0.68) | 0.28 (0.16-0.45) | 1.00 (0.88-1.00) | 2.14 (1.53-2.99)* | 0.09 (0.01-1.35)* | 0.78 (0.71-0.85) |
| Karge et al (2022), Germany | BMI ≥ 25 kg/m^2 | neonatal seizures | 141 | 3 (2.13) | 0.67 (0.21-0.94) | 0.60 (0.52-0.68) | 0.04 (0.01-0.12) | 0.99 (0.94-1.00) | 1.67 (0.73-3.82) | 0.55 (0.11-2.76) | 0.63 (0.30-0.96) |
| Karge et al (2022), Germany | BMI ≥ 30 kg/m^2 | neonatal seizures | 141 | 3 (2.13) | 0.00 (0.00-0.56) | 0.80 (0.72-0.86) | 0.00 (0.00-0.12) | 0.97 (0.92-0.99) | 0.61 (0.04-8.32)* | 1.10 (0.75-1.61)* | 0.40 (0.36-0.43) |
| Zarean et al (2022), Iran | CPR < 1 | neonatal seizures | 100 | 1 (1.00) | 1.00 (0.21-1.00) | 0.74 (0.64-0.81) | 0.04 (0.01-0.18) | 1.00 (0.95-1.00) | 2.83 (1.19-6.72)* | 0.34 (0.03-3.76)* | NA |
| **Neonatal pulmonary outcomes** | | | | | | | | | | | |
| Binder et al (2021), Austria | angiogenic imbalance (sFlt-1/PlGF ratio above the 97th percentile and/or PlGF levels below 2.5th centile) without proteinuria | RDS | 145 | 26 (17.93) | 0.62 (0.43-0.78) | 0.80 (0.72-0.86) | 0.40 (0.26-0.55) | 0.90 (0.83-0.95) | 3.05 (1.91-4.88) | 0.48 (0.29-0.79) | 0.71 (0.60-0.81) |
| Binder et al (2021), Austria | angiogenic imbalance (sFlt-1/PlGF ratio above the 97th percentile and/or PlGF levels below 2.5th centile) without IUGR | RDS | 145 | 26 (17.93) | 0.46 (0.29-0.65) | 0.80 (0.72-0.86) | 0.33 (0.20-0.50) | 0.87 (0.80-0.92) | 2.29 (1.32-3.96) | 0.67 (0.47-0.97) | 0.63 (0.53-0.73) |
| Binder et al (2021), Austria | angiogenic imbalance (sFlt-1/PlGF ratio above the 97th percentile and/or PlGF levels below 2.5th centile) and/or proteinuria | RDS | 145 | 26 (17.93) | 0.88 (0.71-0.96) | 0.71 (0.63-0.79) | 0.40 (0.29-0.53) | 0.97 (0.90-0.99) | 3.10 (2.26-4.25) | 0.16 (0.06-0.47) | 0.80 (0.72-0.87) |
| Binder et al (2021), Austria | proteinuria, other symptoms or IUGR without angiogenic imbalance | RDS | 145 | 26 (17.93) | 0.00 (0.00-0.13) | 0.90 (0.83-0.94) | 0.00 (0.00-0.24) | 0.80 (0.73-0.86) | 0.18 (0.01-2.91)* | 1.10 (1.01-1.19)* | 0.45 (0.42-0.48) |
| Karge et al (2021), Germany | early-onset pre-eclampsia | RDS | 49 | 7 (14.29) | 0.86 (0.49-0.97) | 0.71 (0.56-0.83) | 0.33 (0.16-0.56) | 0.97 (0.84-0.99) | 3.00 (1.70-5.28) | 0.20 (0.03-1.24) | 0.79 (0.63-0.94) |
| Karge et al (2021), Germany | late-onset pre-eclampsia | RDS | 49 | 7 (14.29) | 0.14 (0.03-0.51) | 0.29 (0.17-0.44) | 0.03 (0.01-0.16) | 0.67 (0.44-0.84) | 0.20 (0.03-1.24) | 3.00 (1.70-5.28) | 0.21 (0.06-0.37) |
| Loardi et al (2021), Italy | abnormal UtA PI > 95 th percentile | RDS | 311 | 104 (33.44) | 0.83 (0.74-0.89) | 0.51 (0.44-0.58) | 0.46 (0.39-0.53) | 0.85 (0.78-0.91) | 1.69 (1.44-2.00) | 0.34 (0.22-0.53) | 0.67 (0.62-0.72) |
| Stolz et al (2018), Austria | sFlt-1/PlGF ratio ≥ 655 | RDS | 54 | 46 (76.70) | 0.50 (0.36-0.64) | 0.50 (0.22-0.78) | 0.85 (0.68-0.94) | 0.15 (0.06-0.32) | 1.00 (0.47-2.12) | 1.00 (0.47-2.12) | 0.50 (0.30-0.70) |
| Sudjai et al (2022), Thailand | serum uric acid level < 5.0 mg/dL | RDS | 400 | 177 (44.25) | 0.19 (0.14-0.25) | 0.72 (0.66-0.77) | 0.34 (0.26-0.44) | 0.53 (0.47-0.58) | 0.66 (0.45-0.96) | 1.13 (1.02-1.26) | 0.45 (0.41-0.49) |
| Sudjai et al (2022), Thailand | serum uric acid level ≥ 5.0 mg/dL | RDS | 400 | 177 (44.25) | 0.81 (0.75-0.86) | 0.28 (0.23-0.34) | 0.47 (0.42-0.53) | 0.66 (0.56-0.74) | 1.13 (1.02-1.26) | 0.66 (0.45-0.96) | 0.55 (0.51-0.59) |
| Sudjai et al (2022), Thailand | serum uric acid level ≥ 7.0 mg/dL | RDS | 400 | 177 (44.25) | 0.41 (0.34-0.48) | 0.77 (0.71-0.82) | 0.58 (0.49-0.66) | 0.62 (0.56-0.67) | 1.74 (1.30-2.35) | 0.77 (0.67-0.89) | 0.59 (0.54-0.63) |
| Tokalioglu et al (2023), Turkey | umbilical artery half peak systolic velocity deceleration time (UA hPSV-DT) <5th percentile | RDS | 55 | 13 (23.64) | 0.85 (0.58-0.96) | 0.74 (0.59-0.85) | 0.50 (0.31-0.69) | 0.94 (0.80-0.98) | 3.23 (1.85-5.65) | 0.21 (0.06-0.76) | 0.79 (0.67-0.91) |
| Tousty et al (2022), Poland | sFlt-1/PlGF ratio ≤ 204 | RDS | 77 | 14 (18.18) | 0.57 (0.33-0.79) | 0.70 (0.58-0.80) | 0.30 (0.16-0.48) | 0.88 (0.76-0.94) | 1.89 (1.05-3.41) | 0.61 (0.33-1.15) | 0.63 (0.49-0.78) |
| Tousty et al (2022), Poland | gestational age at delivery ≤ 32 weeks | RDS | 77 | 14 (18.18) | 0.86 (0.60-0.96) | 0.87 (0.77-0.93) | 0.60 (0.39-0.78) | 0.96 (0.88-0.99) | 6.75 (3.41-13.35) | 0.16 (0.05-0.59) | 0.87 (0.76-0.97) |
| Graupner et al (2019), Germany | sFlt-1/PlGF ratio ≥ 110 | RDS (ventilation support for > 4h) | 67 | 3 (4.50) | 0.00 (0.00-0.56) | 0.58 (0.46-0.69) | 0.00 (0.00-0.12) | 0.92 (0.80-0.97) | 0.30 (0.02-4.01)* | 1.52 (0.99-2.32)* | 0.29 (0.23-0.35) |
| Graupner et al (2019), Germany | CPR < 5th centile | RDS (ventilation support for > 4h) | 67 | 3 (4.50) | 0.00 (0.00-0.56) | 0.89 (0.79-0.95) | 0.00 (0.00-0.35) | 0.95 (0.86-0.98) | 1.08 (0.07-15.78)* | 0.99 (0.68-1.45)* | 0.45 (0.41-0.48) |
| Graupner et al (2019), Germany | mean UtA PI > 95th centile | RDS (ventilation support for > 4h) | 67 | 3 (4.50) | 0.00 (0.00-0.56) | 0.64 (0.52-0.75) | 0.00 (0.00-0.14) | 0.93 (0.82-0.98) | 0.35 (0.03-4.72)* | 1.37 (0.91-2.07)* | 0.32 (0.26-0.38) |
| Tousty et al (2022), Poland | gestational age at delivery ≤ 32 weeks | BPD | 77 | 8 (10.39) | 1.00 (0.68-1.00) | 0.83 (0.72-0.90) | 0.40 (0.22-0.61) | 1.00 (0.94-1.00) | 5.29 (3.12-8.96)* | 0.07 (0.00-1.00)* | 0.91 (0.87-0.96) |
| Tousty et al (2022), Poland | sFlt-1/PlGF ratio ≥ 204 | BPD | 77 | 8 (10.39) | 0.75 (0.41-0.93) | 0.70 (0.58-0.79) | 0.22 (0.11-0.41) | 0.96 (0.87-0.99) | 2.46 (1.44-4.21) | 0.36 (0.11-1.21) | 0.72 (0.55-0.89) |
| Nayak et al (2022), India | CPR < 1 | CPAP | 62 | NA (12.90) | 1.00 (0.68-1.00) | 0.67 (0.53-0.78) | 0.31 (0.17-0.50) | 1.00 (0.90-1.00) | NA | NA | NA |
| Nayak et al (2022), India | NST | CPAP | 62 | 8 (12.90) | 0.50 (0.22-0.78) | 0.65 (0.51-0.76) | 0.17 (0.70-0.37) | 0.90 (0.76-0.96) | 1.42 (0.65-3.11) | 0.77 (0.38-1.59) | 0.57 (0.38-0.77) |
| Nayak et al (2022), India | CPR < 1 | BMV | 62 | NA (11.29) | 1.00 (0.65-1.00) | 0.65 (0.52-0.77) | 0.27 (0.14-0.46) | 1.00 (0.90-1.00) | NA | NA | NA |
| Nayak et al (2022), India | NST | BMV | 62 | 7 (11.29) | 0.71 (0.36-0.92) | 0.67 (0.54-0.78) | 0.22 (0.97-0.42) | 0.95 (0.83-0.99) | 2.18 (1.19-3.99) | 0.42 (0.13-1.39) | 0.69 (0.50-0.88) |
| Binder et al (2021), Austria | angiogenic imbalance (sFlt-1/PlGF ratio above the 97th percentile and/or PlGF levels below 2.5th centile) without proteinuria | ventilation support | 145 | 28 (19.30) | 0.57 (0.39-0.73) | 0.79 (0.71-0.86) | 0.40 (0.26-0.55) | 0.89 (0.81-0.93) | 2.79 (1.72-4.50) | 0.54 (0.35-0.84) | 0.68 (0.58-0.78) |
| Binder et al (2021), Austria | angiogenic imbalance (sFlt-1/PlGF ratio above the 97th percentile and/or PlGF levels below 2.5th centile) without IUGR | ventilation support | 145 | 28 (19.30) | 0.43 (0.27-0.61) | 0.79 (0.71-0.86) | 0.33 (0.20-0.50) | 0.85 (0.77-0.91) | 2.09 (1.20-3.65) | 0.72 (0.51-1.00) | 0.61 (0.51-0.71) |
| Binder et al (2021), Austria | angiogenic imbalance (sFlt-1/PlGF ratio above the 97th percentile and/or PlGF levels below 2.5th centile) and/or proteinuria | ventilation support | 145 | 28 (19.30) | 0.86 (0.69-0.94) | 0.72 (0.63-0.79) | 0.42 (0.30-0.55) | 0.95 (0.89-0.98) | 3.04 (2.19-4.21) | 0.20 (0.08-0.50) | 0.79 (0.71-0.87) |
| Binder et al (2021), Austria | proteinuria, other symptoms or IUGR without angiogenic imbalance | ventilation support | 145 | 28 (19.30) | 0.00 (0.00-0.12) | 0.90 (0.83-0.94) | 0.00 (0.00-0.24) | 0.79 (0.71-0.85) | 0.16 (0.01-2.67)* | 1.10 (1.02-1.19)* | 0.45 (0.42-0.48) |
| Fishel Bartal et al (2022), US | Ethnicity: Non-Hispanic White | ventilation support (longer than 6 hours) | 389,131 | 15600 (4.00) | 0.49 (0.48-0.50) | 0.50 (0.50-0.50) | 0.04 (0.04-0.04) | 0.96 (0.96-0.96) | 0.98 (0.96-1.00) | 1.02 (1.00-1.04) | 0.50 (0.49-0.50) |
| Fishel Bartal et al (2022), US | Ethnicity: Non-Hispanic Black | ventilation support (longer than 6 hours) | 389,131 | 15600 (4.00) | 0.33 (0.32-0.34) | 0.70 (0.70-0.71) | 0.04 (0.04-0.05) | 0.96 (0.96-0.96) | 1.12 (1.09-1.14) | 0.95 (0.94-0.96) | 0.52 (0.51-0.52) |
| Fishel Bartal et al (2022), US | Ethnicity: Hispanic | ventilation support (longer than 6 hours) | 389,131 | 15600 (4.00) | 0.13 (0.13-0.14) | 0.84 (0.84-0.85) | 0.03 (0.03-0.04) | 0.96 (0.96-0.96) | 0.84 (0.80-0.87) | 1.03 (1.02-1.04) | 0.49 (0.48-0.49) |
| Fishel Bartal et al (2022), US | Ethnicity: Non-Hispanic Asian American, Native Hawaiian and other Pacific Islander | ventilation support (longer than 6 hours) | 389,131 | 15600 (4.00) | 0.04 (0.03-0.04) | 0.96 (0.96-0.96) | 0.04 (0.04-0.04) | 0.96 (0.96-0.96) | 1.00 (0.92-1.09) | 1.00 (1.00-1.00) | 0.50 (0.50-0.50) |
| Fishel Bartal et al (2022), US | Ethnicity: Non-Hispanic American Indian and Alaska Native | ventilation support (longer than 6 hours) | 389,131 | 15600 (4.00) | 0.01 (0.01-0.01) | 0.99 (0.99-0.99) | 0.04 (0.04-0.05) | 0.96 (0.96-0.96) | 1.12 (0.97-1.29) | 1.00 (1.00-1.00) | 0.50 (0.50-0.50) |
| Govender et al (2022), South Africa | ductus venosus Doppler abnormal | ventilation | 61 | 13 (21.31) | 0.85 (0.58-0.96) | 0.56 (0.42-0.69) | 0.34 (0.20-0.52) | 0.93 (0.78-0.98) | 1.93 (1.30-2.87) | 0.27 (0.07-1.00) | 0.70 (0.58-0.83) |
| Karge et al (2021), Germany | early-onset pre-eclampsia | ventilation support | 49 | 4 (8.16) | 0.75 (0.30-0.95) | 0.67 (0.52-0.79) | 0.17 (0.06-0.39) | 0.97 (0.84-0.99) | 2.25 (1.12-4.53) | 0.38 (0.07-2.07) | 0.71 (0.45-0.96) |
| Karge et al (2021), Germany | late-onset pre-eclampsia | ventilation support | 49 | 4 (8.16) | 0.25 (0.05-0.70) | 0.33 (0.21-0.48) | 0.03 (0.01-0.16) | 0.83 (0.61-0.94) | 0.38 (0.07-2.07) | 2.25 (1.12-4.53) | 0.29 (0.04-0.55) |
| Karge et al (2022), Germany | BMI ≥ 25 kg/m^2 | ventilation support | 141 | 42 (29.79) | 0.36 (0.23-0.51) | 0.58 (0.48-0.67) | 0.26 (0.17-0.39) | 0.68 (0.57-0.77) | 0.84 (0.53-1.34) | 1.12 (0.84-1.48) | 0.47 (0.38-0.55) |
| Karge et al (2022), Germany | BMI ≥ 30 kg/m^2 | ventilation support | 141 | 42 (29.79) | 0.17 (0.08-0.31) | 0.79 (0.70-0.86) | 0.25 (0.13-0.43) | 0.69 (0.60-0.77) | 0.79 (0.36-1.71) | 1.06 (0.89-1.25) | 0.48 (0.41-0.55) |
| Morikawa et al (2021), Japan | gestational weight gain during the week prior delivery ≥ 1.6 kg | ventilation support | 94 | 22 (23.40) | 0.64 (0.43-0.80) | 0.54 (0.43-0.65) | 0.30 (0.19-0.44) | 0.83 (0.70-0.91) | 1.39 (0.93-2.08) | 0.67 (0.37-1.21) | 0.59 (0.47-0.71) |
| Zarean et al (2022), Iran | CPR < 1 | ventilation support | 100 | NA | 0.52 | 0.67 | 0.37 | 0.79 | NA | NA | NA |
| **Preterm delivery** | | | | | | | | | | | |
| Le et al (2018), Vietnam | serum uric acid level > 410 µmol/L | preterm delivery (not defined) | 205 | NA (22.40) | 0.65 | 0.79 | NA | NA | NA | NA | 0.75 |
| Madhu et al (2023), India | increase in UA S/D ratio | preterm birth | 150 | 22 (14.67) | 0.26 (0.23-0.61) | 0.80 (0.73-0.86) | 0.26 (0.15-0.43) | 0.89 (0.82-0.93) | 2.09 (1.13-3.87) | 0.73 (0.51-1.05) | 0.61 (0.50-0.72) |
| Madhu et al (2023), India | elevated UA RI | preterm birth | 150 | 22 (14.67) | 0.24 (0.10-0.43) | 0.88 (0.81-0.92) | 0.24 (0.11-0.45) | 0.87 (0.80-0.92) | 1.82 (0.74-4.46) | 0.88 (0.70-1.12) | 0.55 (0.46-0.65) |
| Madhu et al (2023), India | elevated UA PI | preterm birth | 150 | 22 (14.67) | 0.36 (0.20-0.57) | 0.82 (0.74-0.88) | 0.26 (0.14-0.43) | 0.88 (0.81-0.93) | 2.02 (1.04-3.94) | 0.78 (0.56-1.07) | 0.59 (0.48-0.70) |
| Madhu et al (2023), India | elevated MCA PI | preterm birth | 150 | 22 (14.67) | 0.14 (0.05-0.33) | 0.94 (0.88-0.97) | 0.27 (0.10-0.57) | 0.86 (0.80-0.91) | 2.18 (0.63-7.60) | 0.92 (0.78-1.09) | 0.54 (0.46-0.61) |
| Madhu et al (2023), India | abnormal CPR <1.08 | preterm birth | 150 | 22 (14.67) | 0.64 (0.43-0.80) | 0.32 (0.25-0.41) | 0.14 (0.08-0.22) | 0.84 (0.71-0.91) | 0.94 (0.67-1.31) | 1.14 (0.62-2.08) | 0.48 (0.37-0.59) |
| Madhu et al (2023), India | UA reversed end diastolic flow | preterm birth | 150 | 22 (14.67) | 0.00 (0.00-0.15) | 0.98 (0.93-0.99) | 0.00 (0.00-0.56) | 0.85 (0.78-0.90) | 0.80 (0.04-15.00)* | 1.01 (0.94-1.08)* | 0.49 (0.48-0.50) |
| Saxena et al (2021), India | CPR < 1 | preterm delivery (not defined) | 150 | 21 (14.00) | 0.37 (0.20-0.56) | 0.92 (0.85-0.96) | 0.52 (0.30-0.74) | 0.85 (0.78-0.91) | 4.46 (1.83-10.91) | 0.68 (0.48-0.95) | 0.65 (0.54-0.76) |
| Sudjai et al (2022), Thailand | serum uric acid level < 5.0 mg/dL | preterm delivery (not defined) | 400 | 223 (55.75) | 0.14 (0.10-0.20) | 0.64 (0.57-0.71) | 0.33 (0.25-0.43) | 0.37 (0.32-0.43) | 0.40 (0.27-0.58) | 1.34 (1.19-1.52) | 0.39 (0.35-0.43) |
| Sudjai et al (2022), Thailand | serum uric acid level ≥ 5.0 mg/dL | preterm delivery (not defined) | 400 | 223 (55.75) | 0.86 (0.80-0.90) | 0.36 (0.29-0.43) | 0.63 (0.57-0.68) | 0.67 (0.57-0.75) | 1.34 (1.19-1.52) | 0.40 (0.27-0.58) | 0.61 (0.57-0.65) |
| Sudjai et al (2022), Thailand | serum uric acid level ≥ 7.0 mg/dL | preterm delivery (not defined) | 400 | 223 (55.75) | 0.39 (0.33-0.46) | 0.80 (0.73-0.85) | 0.71 (0.62-0.78) | 0.51 (0.45-0.57) | 1.94 (1.39-2.71) | 0.76 (0.67-0.87) | 0.60 (0.55-0.64) |
| Abraham et al (2019), India | TOH ≤ 258 days | preterm birth <37 weeks | 174 | NA (43.10) | 0.97 | 0.81 | NA | NA | NA | NA | 0.897 (0.845–0.94) |
| Abraham et al (2019), India | SBP ≥ 149 mmHg | preterm birth <37 weeks | 174 | NA (43.10) | 0.86 | 0.52 | NA | NA | NA | NA | 0.765 (0.698–0.833) |
| Abraham et al (2019), India | TAS ≥ 957.7 µmol/L | preterm birth <37 weeks | 174 | NA (43.10) | 0.76 | 0.58 | NA | NA | NA | NA | 0.718 (0.638–0.790) |
| Abraham et al (2019), India | TOH (days) + SBP (mmHg) + TAS (µmol/L) | preterm birth <37 weeks | 174 | NA (43.10) | 0.84 | 0.91 | NA | NA | NA | NA | 0.885 (0.833–0.937) |
| Abraham et al (2019), India | TOH (days) + SBP (mmHg) | preterm birth <37 weeks | 174 | NA (43.10) | 0.96 | 0.74 | NA | NA | NA | NA | 0.886 (0.836–0.935) |
| Abraham et al (2019), India | TOH (days) + TAS (µmol/L) | preterm birth <37 weeks | 174 | NA (43.10) | 0.91 | 0.82 | NA | NA | NA | NA | 0.882 (0.825–0.938) |
| Abraham et al (2019), India | SBP (mmHg) + TAS (µmol/L) | preterm birth <37 weeks | 174 | NA (43.10) | 0.86 | 0.62 | NA | NA | NA | NA | 0.809 (0.744–0.874) |
| Chadha et al (2022), India | UPCR ≥ 0.3 | preterm birth <37 weeks | 130 | 92 (65.20) | 0.95 (0.88-0.98) | 0.20 (0.11-0.34) | 0.69 (0.61-0.76) | 0.67 (0.42-0.85) | 1.19 (1.02-1.38) | 0.27 (0.10-0.74) | 0.57 (0.51-0.64) |
| Fishel Bartal et al (2022), US | Ethnicity: Non-Hispanic White | preterm birth <37 weeks | 389,347 | 81883 (21.03) | 0.42 (0.42-0.42) | 0.48 (0.47-0.48) | 0.18 (0.17-0.18) | 0.76 (0.75-0.76) | 0.80 (0.80-0.81) | 1.22 (1.21-1.22) | 0.45 (0.45-0.45) |
| Fishel Bartal et al (2022), US | Ethnicity: Non-Hispanic Black | preterm birth <37 weeks | 389,347 | 81883 (21.03) | 0.36 (0.36-0.36) | 0.72 (0.72-0.72) | 0.25 (0.25-0.26) | 0.81 (0.81-0.81) | 1.28 (1.27-1.30) | 0.89 (0.88-0.89) | 0.54 (0.54-0.54) |
| Fishel Bartal et al (2022), US | Ethnicity: Hispanic | preterm birth <37 weeks | 389,347 | 81883 (21.03) | 0.17 (0.17-0.17) | 0.85 (0.85-0.85) | 0.23 (0.22-0.23) | 0.79 (0.79-0.79) | 1.11 (1.09-1.13) | 0.98 (0.98-0.98) | 0.51 (0.51-0.51) |
| Fishel Bartal et al (2022), US | Ethnicity: Non-Hispanic Asian American, Native Hawaiian and other Pacific Islander | preterm birth <37 weeks | 389,347 | 81883 (21.03) | 0.04 (0.04-0.04) | 0.97 (0.96-0.97) | 0.23 (0.23-0.24) | 0.79 (0.79-0.79) | 1.14 (1.10-1.19) | 0.99 (0.99-1.00) | 0.50 (0.50-0.50) |
| Fishel Bartal et al (2022), US | Ethnicity: Non-Hispanic American Indian and Alaska Native | preterm birth <37 weeks | 389,347 | 81883 (21.03) | 0.01 (0.01-0.01) | 0.99 (0.99-0.99) | 0.24 (0.23-0.25) | 0.79 (0.79-0.79) | 1.18 (1.10-1.27) | 1.00 (1.00-1.00) | 0.50 (0.50-0.50) |
| Joshi et al (2022), India | raised serum hs-CRP > 3.0 mg/mL | preterm birth <37 weeks | 132 | 35 (26.52) | 0.91 (0.78-0.97) | 0.71 (0.61-0.79) | 0.53 (0.41-0.65) | 0.96 (0.88-0.99) | 3.17 (2.28-4.40) | 0.12 (0.04-0.36) | 0.81 (0.75-0.88) |
| Karge et al (2022), Germany | early-onset pre-eclampsia | preterm birth <37 weeks | 49 | 33 (67.35) | 0.36 (0.22-0.53) | 0.62 (0.39-0.82) | 0.67 (0.44-0.84) | 0.32 (0.19-0.50) | 0.97 (0.45-2.11) | 1.02 (0.64-1.61) | 0.49 (0.35-0.64) |
| Karge et al (2022), Germany | late-onset pre-eclampsia | preterm birth <37 weeks | 49 | 33 (67.35) | 0.64 (0.47-0.78) | 0.38 (0.18-0.61) | 0.68 (0.50-0.81) | 0.33 (0.16-0.56) | 1.02 (0.64-1.61) | 0.97 (0.45-2.11) | 0.51 (0.36-0.65) |
| Leanos-Miranda et el (2020), Mexico | sFlt-1/PlGF ratio ≥ 38 | preterm birth <37 weeks | 810 | 633 (78.15) | 0.76 (0.73-0.79) | 0.76 (0.69-0.81) | 0.92 (0.89-0.94) | 0.47 (0.41-0.53) | 3.14 (2.41-4.09) | 0.31 (0.27-0.37) | 0.76 (0.72-0.80) |
| Leanos-Miranda et el (2020), Mexico | sFlt-1/PlGF ratio ≥ 85 | preterm birth <37 weeks | 810 | 633 (78.15) | 0.65 (0.62-0.69) | 0.94 (0.90-0.97) | 0.98 (0.96-0.99) | 0.43 (0.38-0.48) | 11.58 (6.32-21.19) | 0.37 (0.33-0.41) | 0.80 (0.77-0.82) |
| Lei et al (2021), China | 24 h Proteinuria excretion <0.3 g/24h | preterm birth <37 weeks | 275 | 56 (20.40) | 0.27 (0.17-0.40) | 0.83 (0.77-0.87) | 0.28 (0.18-0.42) | 0.82 (0.76-0.86) | 1.54 (0.92-2.60) | 0.89 (0.75-1.05) | 0.55 (0.48-0.61) |
| Lei et al (2021), China | 24 h Proteinuria excretion ≥ 0.3 g/24h | preterm birth <37 weeks | 275 | 56 (20.40) | 0.45 (0.32-0.58) | 0.58 (0.51-0.64) | 0.21 (0.15-0.30) | 0.80 (0.73-0.86) | 1.06 (0.76-1.48) | 0.95 (0.74-1.24) | 0.51 (0.44-0.59) |
| Lei et al (2021), China | 24 h Proteinuria excretion ≥ 2.0 g/24h | preterm birth <37 weeks | 275 | 56 (20.40) | 0.32 (0.21-0.45) | 0.84 (0.79-0.88) | 0.34 (0.23-0.47) | 0.83 (0.77-0.87) | 2.01 (1.24-3.27) | 0.81 (0.67-0.98) | 0.58 (0.51-0.65) |
| Lei et al (2021), China | 24 h Proteinuria excretion ≥ 9.8475 g/24h | preterm birth <37 weeks | 275 | NA (20.40) | 0.62 | 0.60 | NA | NA | NA | NA | 0.618 (0.511–0.724) |
| Magee et al (2016), multinational | controlled BP ≤ 100 mmHg | preterm birth <37 weeks | 978 | 328 (33.50) | 0.53 (0.48-0.59) | 0.51 (0.47-0.55) | 0.36 (0.31-0.40) | 0.69 (0.64-0.72) | 1.09 (0.96-1.24) | 0.91 (0.79-1.05) | 0.52 (0.49-0.56) |
| Magee et al (2016), multinational | controlled BP ≤ 85 mmHg | preterm birth <37 weeks | 978 | 328 (33.50) | 0.47 (0.41-0.52) | 0.49 (0.45-0.53) | 0.31 (0.28-0.36) | 0.64 (0.60-0.69) | 0.91 (0.79-1.05) | 1.09 (0.96-1.24) | 0.48 (0.44-0.51) |
| Magee et al (2016), multinational | age ≥ 35 years | preterm birth <37 weeks | 978 | 328 (33.50) | 0.42 (0.37-0.47) | 0.56 (0.52-0.60) | 0.32 (0.28-0.37) | 0.66 (0.62-0.69) | 0.95 (0.81-1.11) | 1.04 (0.93-1.17) | 0.49 (0.46-0.52) |
| Magee et al (2016), multinational | Ethnicity: Caucasian/Asian/Other | preterm birth <37 weeks | 978 | 328 (33.50) | 0.74 (0.69-0.78) | 0.24 (0.21-0.27) | 0.33 (0.30-0.36) | 0.64 (0.58-0.70) | 0.97 (0.90-1.05) | 1.09 (0.87-1.37) | 0.49 (0.46-0.52) |
| Magee et al (2016), multinational | Ethnicity: Black/Hispanic | preterm birth <37 weeks | 978 | 328 (33.50) | 0.26 (0.22-0.31) | 0.76 (0.73-0.79) | 0.36 (0.30-0.42) | 0.67 (0.64-0.70) | 1.09 (0.87-1.37) | 0.97 (0.90-1.05) | 0.51 (0.48-0.54) |
| Magee et al (2016), multinational | BMI ≥ 25 kg/m^2 | preterm birth <37 weeks | 969 | 325 (33.50) | 0.73 (0.68-0.77) | 0.22 (0.19-0.25) | 0.32 (0.29-0.35) | 0.61 (0.55-0.67) | 0.93 (0.86-1.00) | 1.27 (1.01-1.60) | 0.47 (0.44-0.50) |
| Magee et al (2016), multinational | conceived through ART | preterm birth <37 weeks | 961 | 322 (33.50) | 0.06 (0.04-0.09) | 0.96 (0.95-0.98) | 0.45 (0.31-0.60) | 0.67 (0.64-0.70) | 1.64 (0.91-2.97) | 0.98 (0.95-1.01) | 0.51 (0.50-0.53) |
| Magee et al (2016), multinational | gestational age ≥ 20 weeks | preterm birth <37 weeks | 978 | 328 (33.50) | 0.75 (0.70-0.79) | 0.33 (0.29-0.36) | 0.36 (0.32-0.40) | 0.72 (0.66-0.77) | 1.11 (1.02-1.20) | 0.78 (0.62-0.96) | 0.54 (0.51-0.57) |
| Magee et al (2016), multinational | nulliparous | preterm birth <37 weeks | 961 | 328 (33.50) | 0.39 (0.34-0.45) | 0.69 (0.66-0.73) | 0.39 (0.34-0.45) | 0.69 (0.66-0.73) | 1.28 (1.08-1.53) | 0.87 (0.79-0.97) | 0.54 (0.51-0.58) |
| Magee et al (2016), multinational | gestational hypertension | preterm birth <37 weeks | 978 | 328 (33.50) | 0.34 (0.29-0.39) | 0.79 (0.75-0.82) | 0.44 (0.38-0.50) | 0.70 (0.67-0.73) | 1.57 (1.27-1.94) | 0.85 (0.78-0.92) | 0.56 (0.53-0.59) |
| Magee et al (2016), multinational | preexisting hypertension | preterm birth <37 weeks | 978 | 328 (33.50) | 0.66 (0.61-0.71) | 0.21 (0.18-0.25) | 0.30 (0.27-0.33) | 0.56 (0.50-0.62) | 0.85 (0.78-0.92) | 1.57 (1.27-1.94) | 0.44 (0.41-0.47) |
| Magee et al (2016), multinational | prior SBP ≥ 160 or DBP ≥ 110 mmHg in this pregnancy | preterm birth <37 weeks | 978 | 328 (33.50) | 0.21 (0.17-0.26) | 0.89 (0.86-0.91) | 0.50 (0.42-0.58) | 0.69 (0.66-0.72) | 1.95 (1.44-2.64) | 0.88 (0.83-0.94) | 0.55 (0.53-0.58) |
| Magee et al (2016), multinational | antihypertensive use at randomization | preterm birth <37 weeks | 978 | 328 (33.50) | 0.60 (0.55-0.65) | 0.44 (0.41-0.48) | 0.35 (0.31-0.39) | 0.69 (0.64-0.73) | 1.08 (0.96-1.21) | 0.90 (0.77-1.06) | 0.52 (0.49-0.55) |
| Magee et al (2016), multinational | labetalol (+other, no methyldopa) | preterm birth <37 weeks | 978 | 328 (33.50) | 0.29 (0.24-0.34) | 0.78 (0.75-0.81) | 0.40 (0.34-0.47) | 0.69 (0.65-0.72) | 1.34 (1.07-1.68) | 0.91 (0.84-0.98) | 0.54 (0.51-0.57) |
| Magee et al (2016), multinational | methyldopa (+other, no labetaol) | preterm birth <37 weeks | 978 | 328 (33.50) | 0.21 (0.17-0.25) | 0.74 (0.70-0.77) | 0.28 (0.23-0.34) | 0.65 (0.61-0.68) | 0.78 (0.61-1.00) | 1.08 (1.00-1.16) | 0.47 (0.44-0.50) |
| Magee et al (2016), multinational | other antihypertensive drugs | preterm birth <37 weeks | 978 | 328 (33.50) | 0.10 (0.08-0.14) | 0.92 (0.90-0.94) | 0.40 (0.31-0.51) | 0.67 (0.64-0.70) | 1.35 (0.89-2.04) | 0.97 (0.93-1.01) | 0.51 (0.49-0.53) |
| Magee et al (2016), multinational | SBP within 1 week before randomization < 140 mmHg | preterm birth <37 weeks | 978 | 328 (33.50) | 0.32 (0.27-0.38) | 0.59 (0.55-0.62) | 0.28 (0.24-0.33) | 0.63 (0.59-0.67) | 0.78 (0.65-0.94) | 1.15 (1.05-1.27) | 0.45 (0.42-0.49) |
| Magee et al (2016), multinational | SBP within 1 week before randomization ≥ 140 mmHg | preterm birth <37 weeks | 978 | 328 (33.50) | 0.68 (0.62-0.73) | 0.41 (0.38-0.45) | 0.37 (0.33-0.41) | 0.72 (0.67-0.76) | 1.15 (1.05-1.27) | 0.78 (0.65-0.94) | 0.55 (0.51-0.58) |
| Magee et al (2016), multinational | SBP within 1 week before randomization ≥ 150 mmHg | preterm birth <37 weeks | 978 | 328 (33.50) | 0.25 (0.21-0.30) | 0.80 (0.77-0.83) | 0.39 (0.33-0.46) | 0.68 (0.65-0.71) | 1.28 (1.00-1.63) | 0.93 (0.87-1.00) | 0.53 (0.50-0.56) |
| Magee et al (2016), multinational | DBP within 1 week before randomization < 90 mmHg | preterm birth <37 weeks | 978 | 328 (33.50) | 0.16 (0.13-0.21) | 0.80 (0.76-0.83) | 0.29 (0.23-0.36) | 0.65 (0.62-0.69) | 0.81 (0.61-1.08) | 1.05 (0.99-1.12) | 0.48 (0.46-0.51) |
| Magee et al (2016), multinational | DBP within 1 week before randomization ≥ 90 mmHg | preterm birth <37 weeks | 978 | 328 (33.50) | 0.84 (0.79-0.87) | 0.20 (0.17-0.24) | 0.35 (0.31-0.38) | 0.71 (0.64-0.77) | 1.05 (0.99-1.12) | 0.81 (0.61-1.08) | 0.52 (0.49-0.54) |
| Magee et al (2016), multinational | DBP within 1 week before randomization ≥ 95 mmHg | preterm birth <37 weeks | 978 | 328 (33.50) | 0.38 (0.32-0.43) | 0.72 (0.68-0.75) | 0.40 (0.35-0.45) | 0.69 (0.66-0.73) | 1.32 (1.09-1.59) | 0.87 (0.79-0.96) | 0.55 (0.51-0.58) |
| Magee et al (2016), multinational | DBP within 1 week before randomization ≥ 100 mmHg | preterm birth <37 weeks | 978 | 328 (33.50) | 0.19 (0.15-0.23) | 0.90 (0.88-0.92) | 0.49 (0.41-0.58) | 0.69 (0.66-0.72) | 1.92 (1.38-2.66) | 0.90 (0.85-0.95) | 0.54 (0.52-0.57) |
| Magee et al (2016), multinational | in hospital at enrolment | preterm birth <37 weeks | 978 | 328 (33.50) | 0.12 (0.09-0.16) | 0.97 (0.95-0.98) | 0.67 (0.54-0.77) | 0.69 (0.66-0.72) | 3.96 (2.36-6.67) | 0.91 (0.87-0.95) | 0.55 (0.53-0.56) |
| Magee et al (2016), multinational | GDM prior to random | preterm birth <37 weeks | 978 | 328 (33.50) | 0.06 (0.04-0.10) | 0.94 (0.91-0.95) | 0.33 (0.23-0.46) | 0.66 (0.63-0.69) | 0.99 (0.60-1.64) | 1.00 (0.97-1.04) | 0.50 (0.48-0.52) |
| Magee et al (2016), multinational | smoking during this pregnancy | preterm birth <37 weeks | 978 | 328 (33.50) | 0.07 (0.05-0.10) | 0.94 (0.92-0.95) | 0.37 (0.26-0.49) | 0.67 (0.64-0.70) | 1.14 (0.69-1.87) | 0.99 (0.96-1.03) | 0.50 (0.49-0.52) |
| Magee et al (2016), multinational | aspirin at enrolment | preterm birth <37 weeks | 978 | 328 (33.50) | 0.26 (0.21-0.31) | 0.74 (0.70-0.77) | 0.33 (0.27-0.39) | 0.66 (0.63-0.70) | 0.97 (0.77-1.21) | 1.01 (0.94-1.09) | 0.50 (0.47-0.52) |
| Magee et al (2016), multinational | folic acid or PNV vitamin at enrolment | preterm birth <37 weeks | 977 | 328 (33.50) | 0.65 (0.59-0.70) | 0.35 (0.31-0.38) | 0.33 (0.30-0.37) | 0.66 (0.61-0.71) | 0.99 (0.90-1.09) | 1.02 (0.85-1.22) | 0.50 (0.46-0.53) |
| Magee et al (2016), multinational | PMR recruiting country ≥ 10 perinatal death/100 births | preterm birth <37 weeks | 978 | 328 (33.50) | 0.18 (0.14-0.22) | 0.84 (0.81-0.87) | 0.36 (0.29-0.44) | 0.67 (0.64-0.70) | 1.14 (0.85-1.53) | 0.97 (0.92-1.03) | 0.51 (0.49-0.54) |
| Magee et al (2016), multinational | multivariable model: less tight (100 mmHg) controlled BP (vs. tight (85 mmHg) controlled BP, BMI (≥ 25 vs <25), ART, Nulliparous, in hospital, preexisting (vs. gestational) Hypertension, sBP within last week, dBP within last week, prior sBP ≥ 150 or dBP ≥ 110 mmHg in this pregnancy, PMR recruiting country (high vs low), Methyldopa (+others, no labetalol), other antihypertensive drugs, no antihypertensive drugs | preterm birth <37 weeks | 978 | NA (33.50) | NA | NA | NA | NA | NA | NA | 0.67 (0.64–0.71) |
| Magee et al (2021), multinational | pre-eclampsia definition A (development of proteinuria) in chronic hypertension | preterm birth <37 weeks | 728 | 217 (29.80) | 0.49 (0.42-0.55) | 0.83 (0.79-0.86) | 0.55 (0.48-0.62) | 0.79 (0.76-0.82) | 2.87 (2.27-3.63) | 0.62 (0.54-0.71) | 0.66 (0.62-0.70) |
| Magee et al (2021), multinational | pre-eclampsia definition B (development of proteinuria AND one or more symptoms, signs, abnormal laboratory test) in chronic hypertension | preterm birth <37 weeks | 728 | 217 (29.80) | 0.28 (0.23-0.34) | 0.92 (0.90-0.94) | 0.60 (0.51-0.69) | 0.75 (0.72-0.78) | 3.59 (2.49-5.18) | 0.78 (0.71-0.85) | 0.60 (0.57-0.63) |
| Magee et al (2021), multinational | pre-eclampsia definition C (one or more symptoms, signs) in chronic hypertension | preterm birth <37 weeks | 728 | 217 (29.80) | 0.52 (0.45-0.58) | 0.76 (0.72-0.79) | 0.48 (0.41-0.54) | 0.79 (0.75-0.82) | 2.14 (1.75-2.62) | 0.64 (0.55-0.74) | 0.64 (0.60-0.68) |
| Magee et al (2021), multinational | pre-eclampsia definition D (one or more symptoms, signs, abnormal laboratory test) in chronic hypertension | preterm birth <37 weeks | 728 | 217 (29.80) | 0.53 (0.46-0.59) | 0.75 (0.71-0.79) | 0.47 (0.41-0.54) | 0.79 (0.75-0.82) | 2.11 (1.74-2.57) | 0.63 (0.54-0.73) | 0.64 (0.60-0.68) |
| Magee et al (2021), multinational | pre-eclampsia definition E (development of proteinuria OR one or more symptoms, signs, abnormal laboratory test) in chronic hypertension | preterm birth <37 weeks | 728 | 217 (29.80) | 0.73 (0.67-0.79) | 0.66 (0.62-0.70) | 0.48 (0.42-0.53) | 0.85 (0.81-0.88) | 2.15 (1.86-2.49) | 0.41 (0.32-0.51) | 0.70 (0.66-0.73) |
| Magee et al (2021), multinational | pre-eclampsia definition F (development of proteinuria development of proteinuria OR one or more symptoms, signs) in chronic hypertension | preterm birth <37 weeks | 728 | 217 (29.80) | 0.72 (0.66-0.78) | 0.67 (0.62-0.70) | 0.48 (0.43-0.53) | 0.85 (0.81-0.88) | 2.16 (1.87-2.51) | 0.42 (0.33-0.52) | 0.69 (0.66-0.73) |
| Magee et al (2021), multinational | pre-eclampsia definition A (development of proteinuria) in gestational hypertension | preterm birth <37 weeks | 248 | 110 (44.35) | 0.51 (0.42-0.60) | 0.78 (0.71-0.84) | 0.65 (0.55-0.74) | 0.67 (0.59-0.73) | 2.34 (1.62-3.38) | 0.63 (0.51-0.77) | 0.65 (0.59-0.70) |
| Magee et al (2021), multinational | pre-eclampsia definition B (development of proteinuria AND one or more symptoms, signs, abnormal laboratory test) in gestational hypertension | preterm birth <37 weeks | 248 | 110 (44.35) | 0.34 (0.25-0.43) | 0.88 (0.82-0.93) | 0.70 (0.56-0.80) | 0.63 (0.56-0.69) | 2.90 (1.71-4.93) | 0.75 (0.65-0.87) | 0.61 (0.56-0.66) |
| Magee et al (2021), multinational | pre-eclampsia definition C (one or more symptoms, signs) in gestational hypertension | preterm birth <37 weeks | 248 | 110 (44.35) | 0.55 (0.46-0.64) | 0.70 (0.61-0.77) | 0.59 (0.50-0.68) | 0.66 (0.58-0.73) | 1.82 (1.35-2.47) | 0.64 (0.51-0.81) | 0.63 (0.56-0.69) |
| Magee et al (2021), multinational | pre-eclampsia definition D (one or more symptoms, signs, abnormal laboratory test) in gestational hypertension | preterm birth <37 weeks | 248 | 110 (44.35) | 0.56 (0.47-0.65) | 0.70 (0.61-0.77) | 0.60 (0.50-0.69) | 0.67 (0.59-0.74) | 1.85 (1.37-2.50) | 0.63 (0.49-0.80) | 0.63 (0.57-0.69) |
| Magee et al (2021), multinational | pre-eclampsia definition E (development of proteinuria OR one or more symptoms, signs, abnormal laboratory test) in gestational hypertension | preterm birth <37 weeks | 248 | 110 (44.35) | 0.74 (0.65-0.81) | 0.59 (0.51-0.67) | 0.59 (0.51-0.67) | 0.74 (0.65-0.81) | 1.81 (1.44-2.29) | 0.44 (0.32-0.62) | 0.67 (0.61-0.72) |
| Magee et al (2021), multinational | pre-eclampsia definition F (development of proteinuria development of proteinuria OR one or more symptoms, signs) in gestational hypertension | preterm birth <37 weeks | 248 | 110 (44.35) | 0.74 (0.65-0.81) | 0.59 (0.51-0.67) | 0.59 (0.51-0.67) | 0.74 (0.65-0.81) | 1.81 (1.44-2.29) | 0.44 (0.32-0.62) | 0.67 (0.61-0.72) |
| Malhamé et al (2022_1), US | obstructive sleep apnea | preterm birth <37 weeks | 71,159 | 16209 (22.78) | 0.00 (0.00-0.01) | 1.00 (1.00-1.00) | 0.26 (0.21-0.31) | 0.77 (0.77-0.78) | 1.19 (0.90-1.56) | 1.00 (1.00-1.00) | 0.50 (0.50-0.50) |
| Morikawa et al (2021), Japan | gestational weight gain during the week prior delivery ≥ 1.5 kg | preterm birth <37 weeks | 94 | 53 (56.40) | 0.66 (0.53-0.77) | 0.63 (0.48-0.76) | 0.70 (0.56-0.81) | 0.59 (0.44-0.72) | 1.81 (1.15-2.82) | 0.54 (0.34-0.83) | 0.65 (0.55-0.75) |
| Webster et al (2018), UK | Ethnicity: White | preterm birth <37 weeks | 4,481 | 701 (15.64) | 0.34 (0.30-0.37) | 0.50 (0.49-0.52) | 0.11 (0.10-0.13) | 0.80 (0.79-0.82) | 0.67 (0.61-0.75) | 1.32 (1.24-1.41) | 0.42 (0.40-0.44) |
| Webster et al (2018), UK | Ethnicity: Black | preterm birth <37 weeks | 4,481 | 701 (15.64) | 0.48 (0.44-0.51) | 0.67 (0.65-0.68) | 0.21 (0.19-0.23) | 0.87 (0.86-0.88) | 1.43 (1.30-1.56) | 0.79 (0.73-0.85) | 0.57 (0.55-0.59) |
| Webster et al (2018), UK | Ethnicity: Asian | preterm birth <37 weeks | 4,481 | 701 (15.64) | 0.11 (0.09-0.14) | 0.92 (0.91-0.93) | 0.20 (0.17-0.25) | 0.85 (0.84-0.86) | 1.37 (1.08-1.74) | 0.97 (0.94-0.99) | 0.51 (0.50-0.53) |
| Ye et al (2020), China | retinopathy staging Stage 3/4 | preterm birth <37 weeks | 452 | 303 (67.00) | 0.17 (0.13-0.21) | 0.92 (0.86-0.95) | 0.81 (0.69-0.89) | 0.35 (0.31-0.40) | 2.05 (1.13-3.73) | 0.91 (0.85-0.97) | 0.54 (0.51-0.57) |
| Karge et al (2022), Germany | early-onset pre-eclampsia | preterm birth <34 weeks | 49 | 5 (10.20) | 1.00 (0.57-1.00) | 0.70 (0.56-0.82) | 0.28 (0.12-0.51) | 1.00 (0.89-1.00) | 3.06 (1.84-5.07)* | 0.12 (0.01-1.70)* | 0.85 (0.78-0.92) |
| Karge et al (2022), Germany | late-onset pre-eclampsia | preterm birth <34 weeks | 49 | 5 (10.20) | 0.00 (0.00-0.43) | 0.30 (0.18-0.44) | 0.00 (0.00-0.11) | 0.72 (0.49-0.88) | 0.12 (0.01-1.70)* | 3.06 (1.84-5.07)* | 0.15 (0.08-0.22) |
| Leanos-Miranda et el (2020), Mexico | sFlt-1/PlGF ratio ≥ 38 | preterm birth <34 weeks | 810 | 445 (54.93) | 0.82 (0.79-0.86) | 0.56 (0.51-0.61) | 0.70 (0.66-0.74) | 0.73 (0.67-0.77) | 1.89 (1.67-2.14) | 0.31 (0.25-0.39) | 0.69 (0.66-0.73) |
| Leanos-Miranda et el (2020), Mexico | sFlt-1/PlGF ratio ≥ 85 | preterm birth <34 weeks | 810 | 445 (54.93) | 0.73 (0.69-0.77) | 0.73 (0.69-0.78) | 0.77 (0.73-0.81) | 0.69 (0.65-0.74) | 2.77 (2.31-3.31) | 0.36 (0.31-0.43) | 0.73 (0.70-0.77) |
| Magee et al (2016), multinational | controlled BP ≤ 100 mmHg | preterm birth <34 weeks | 978 | 138 (14.10) | 0.56 (0.47-0.64) | 0.51 (0.47-0.54) | 0.16 (0.13-0.19) | 0.87 (0.84-0.90) | 1.13 (0.96-1.33) | 0.87 (0.72-1.07) | 0.53 (0.49-0.58) |
| Magee et al (2016), multinational | controlled BP ≤ 85 mmHg | preterm birth <34 weeks | 978 | 138 (14.10) | 0.44 (0.36-0.53) | 0.49 (0.46-0.53) | 0.13 (0.10-0.16) | 0.84 (0.81-0.87) | 0.87 (0.72-1.07) | 1.13 (0.96-1.33) | 0.47 (0.42-0.51) |
| Magee et al (2016), multinational | age ≥ 35 years | preterm birth <34 weeks | 978 | 138 (14.10) | 0.44 (0.36-0.53) | 0.57 (0.54-0.60) | 0.14 (0.11-0.18) | 0.86 (0.83-0.89) | 1.03 (0.84-1.26) | 0.98 (0.84-1.15) | 0.51 (0.46-0.55) |
| Magee et al (2016), multinational | Ethnicity: Caucasian/Asian/Other | preterm birth <34 weeks | 978 | 138 (14.10) | 0.72 (0.64-0.79) | 0.24 (0.21-0.27) | 0.13 (0.11-0.16) | 0.84 (0.79-0.88) | 0.95 (0.85-1.06) | 1.17 (0.87-1.57) | 0.48 (0.44-0.52) |
| Magee et al (2016), multinational | Ethnicity: Black/Hispanic | preterm birth <34 weeks | 978 | 138 (14.10) | 0.28 (0.21-0.36) | 0.76 (0.73-0.79) | 0.16 (0.12-0.21) | 0.87 (0.84-0.89) | 1.17 (0.87-1.57) | 0.95 (0.85-1.06) | 0.52 (0.48-0.56) |
| Magee et al (2016), multinational | BMI ≥ 25 kg/m^2 | preterm birth <34 weeks | 969 | 137 (14.10) | 0.71 (0.63-0.78) | 0.23 (0.20-0.26) | 0.13 (0.11-0.16) | 0.82 (0.77-0.87) | 0.91 (0.82-1.02) | 1.29 (0.97-1.73) | 0.47 (0.43-0.51) |
| Magee et al (2016), multinational | conceived through ART | preterm birth <34 weeks | 961 | 136 (14.10) | 0.08 (0.05-0.14) | 0.96 (0.95-0.97) | 0.26 (0.15-0.41) | 0.86 (0.84-0.88) | 2.15 (1.11-4.18) | 0.96 (0.91-1.01) | 0.52 (0.50-0.55) |
| Magee et al (2016), multinational | gestational age ≥ 20 weeks | preterm birth <34 weeks | 978 | 138 (14.10) | 0.69 (0.61-0.76) | 0.30 (0.27-0.33) | 0.14 (0.12-0.17) | 0.85 (0.81-0.89) | 0.98 (0.87-1.11) | 1.04 (0.79-1.36) | 0.49 (0.45-0.54) |
| Magee et al (2016), multinational | nulliparous | preterm birth <34 weeks | 961 | 138 (14.10) | 0.36 (0.29-0.45) | 0.67 (0.64-0.70) | 0.15 (0.12-0.20) | 0.86 (0.84-0.89) | 1.09 (0.86-1.39) | 0.95 (0.83-1.09) | 0.52 (0.47-0.56) |
| Magee et al (2016), multinational | gestational hypertension | preterm birth <34 weeks | 978 | 138 (14.10) | 0.31 (0.24-0.39) | 0.75 (0.72-0.78) | 0.17 (0.13-0.22) | 0.87 (0.84-0.89) | 1.27 (0.97-1.67) | 0.91 (0.81-1.03) | 0.53 (0.49-0.57) |
| Magee et al (2016), multinational | preexisting hypertension | preterm birth <34 weeks | 978 | 138 (14.10) | 0.69 (0.61-0.76) | 0.25 (0.22-0.28) | 0.13 (0.11-0.16) | 0.83 (0.78-0.87) | 0.91 (0.81-1.03) | 1.27 (0.97-1.67) | 0.47 (0.43-0.51) |
| Magee et al (2016), multinational | prior SBP ≥ 160 or DBP ≥ 110 mmHg in this pregnancy | preterm birth <34 weeks | 978 | 138 (14.10) | 0.24 (0.18-0.32) | 0.87 (0.85-0.89) | 0.23 (0.17-0.31) | 0.87 (0.85-0.90) | 1.86 (1.32-2.63) | 0.87 (0.79-0.96) | 0.56 (0.52-0.59) |
| Magee et al (2016), multinational | antihypertensive use at randomization | preterm birth <34 weeks | 978 | 138 (14.10) | 0.64 (0.55-0.71) | 0.44 (0.41-0.47) | 0.16 (0.13-0.19) | 0.88 (0.85-0.91) | 1.14 (0.99-1.31) | 0.82 (0.65-1.04) | 0.54 (0.49-0.58) |
| Magee et al (2016), multinational | labetalol (+other, no methyldopa) | preterm birth <34 weeks | 978 | 138 (14.10) | 0.30 (0.23-0.38) | 0.77 (0.74-0.80) | 0.17 (0.13-0.23) | 0.87 (0.84-0.89) | 1.29 (0.97-1.71) | 0.91 (0.81-1.02) | 0.53 (0.49-0.57) |
| Magee et al (2016), multinational | methyldopa (+other, no labetaol) | preterm birth <34 weeks | 978 | 138 (14.10) | 0.22 (0.16-0.30) | 0.75 (0.72-0.78) | 0.13 (0.09-0.18) | 0.86 (0.83-0.88) | 0.90 (0.65-1.26) | 1.03 (0.94-1.14) | 0.49 (0.45-0.53) |
| Magee et al (2016), multinational | other antihypertensive drugs | preterm birth <34 weeks | 978 | 138 (14.10) | 0.12 (0.07-0.18) | 0.92 (0.90-0.94) | 0.19 (0.12-0.29) | 0.86 (0.84-0.88) | 1.43 (0.86-2.39) | 0.96 (0.90-1.03) | 0.52 (0.49-0.55) |
| Magee et al (2016), multinational | SBP within 1 week before randomization < 140 mmHg | preterm birth <34 weeks | 978 | 138 (14.10) | 0.32 (0.25-0.40) | 0.61 (0.57-0.64) | 0.12 (0.09-0.15) | 0.84 (0.81-0.87) | 0.81 (0.63-1.05) | 1.12 (0.99-1.28) | 0.46 (0.42-0.50) |
| Magee et al (2016), multinational | SBP within 1 week before randomization ≥ 140 mmHg | preterm birth <34 weeks | 978 | 138 (14.10) | 0.68 (0.60-0.75) | 0.39 (0.36-0.43) | 0.16 (0.13-0.19) | 0.88 (0.85-0.91) | 1.12 (0.99-1.28) | 0.81 (0.63-1.05) | 0.54 (0.50-0.58) |
| Magee et al (2016), multinational | SBP within 1 week before randomization ≥ 150 mmHg | preterm birth <34 weeks | 978 | 138 (14.10) | 0.26 (0.19-0.34) | 0.79 (0.77-0.82) | 0.17 (0.13-0.23) | 0.87 (0.84-0.89) | 1.27 (0.93-1.73) | 0.93 (0.84-1.03) | 0.53 (0.49-0.57) |
| Magee et al (2016), multinational | DBP within 1 week before randomization < 90 mmHg | preterm birth <34 weeks | 978 | 138 (14.10) | 0.17 (0.11-0.24) | 0.84 (0.81-0.86) | 0.14 (0.10-0.21) | 0.86 (0.83-0.88) | 1.03 (0.69-1.54) | 0.99 (0.92-1.08) | 0.50 (0.47-0.54) |
| Magee et al (2016), multinational | DBP within 1 week before randomization ≥ 90 mmHg | preterm birth <34 weeks | 978 | 138 (14.10) | 0.83 (0.76-0.89) | 0.19 (0.17-0.22) | 0.15 (0.12-0.17) | 0.88 (0.82-0.92) | 1.03 (0.95-1.12) | 0.86 (0.58-1.28) | 0.51 (0.48-0.55) |
| Magee et al (2016), multinational | DBP within 1 week before randomization ≥ 95 mmHg | preterm birth <34 weeks | 978 | 138 (14.10) | 0.39 (0.31-0.47) | 0.70 (0.67-0.73) | 0.18 (0.14-0.22) | 0.87 (0.85-0.90) | 1.29 (1.03-1.63) | 0.87 (0.76-1.00) | 0.54 (0.50-0.59) |
| Magee et al (2016), multinational | DBP within 1 week before randomization ≥ 100 mmHg | preterm birth <34 weeks | 978 | 138 (14.10) | 0.21 (0.15-0.29) | 0.89 (0.86-0.91) | 0.23 (0.17-0.32) | 0.87 (0.85-0.89) | 1.86 (1.28-2.70) | 0.89 (0.81-0.97) | 0.55 (0.51-0.58) |
| Magee et al (2016), multinational | in hospital at enrolment | preterm birth <34 weeks | 978 | 138 (14.10) | 0.17 (0.12-0.25) | 0.96 (0.94-0.97) | 0.40 (0.29-0.53) | 0.88 (0.85-0.90) | 4.06 (2.50-6.58) | 0.86 (0.80-0.93) | 0.57 (0.53-0.60) |
| Magee et al (2016), multinational | GDM prior to random | preterm birth <34 weeks | 978 | 138 (14.10) | 0.07 (0.03-0.12) | 0.94 (0.92-0.95) | 0.14 (0.08-0.25) | 0.86 (0.83-0.88) | 1.01 (0.51-2.01) | 1.00 (0.95-1.05) | 0.50 (0.48-0.52) |
| Magee et al (2016), multinational | smoking during this pregnancy | preterm birth <34 weeks | 978 | 138 (14.10) | 0.08 (0.05-0.14) | 0.94 (0.92-0.95) | 0.17 (0.10-0.29) | 0.86 (0.84-0.88) | 1.29 (0.69-2.41) | 0.98 (0.93-1.03) | 0.51 (0.48-0.53) |
| Magee et al (2016), multinational | aspirin at enrolment | preterm birth <34 weeks | 978 | 138 (14.10) | 0.33 (0.25-0.41) | 0.75 (0.72-0.78) | 0.18 (0.13-0.23) | 0.87 (0.84-0.89) | 1.30 (0.99-1.70) | 0.90 (0.80-1.02) | 0.54 (0.50-0.58) |
| Magee et al (2016), multinational | folic acid or PNV vitamin at enrolment | preterm birth <34 weeks | 977 | 138 (14.10) | 0.61 (0.53-0.69) | 0.34 (0.31-0.37) | 0.13 (0.11-0.16) | 0.84 (0.80-0.88) | 0.93 (0.80-1.07) | 1.14 (0.91-1.44) | 0.48 (0.43-0.52) |
| Magee et al (2016), multinational | PMR recruiting country ≥ 10 perinatal death/100 births | preterm birth <34 weeks | 978 | 138 (14.10) | 0.16 (0.11-0.23) | 0.84 (0.81-0.86) | 0.14 (0.09-0.20) | 0.86 (0.83-0.88) | 0.98 (0.65-1.48) | 1.00 (0.93-1.09) | 0.50 (0.47-0.53) |
| Magee et al (2016), multinational | multivariable model: less tight (100 mmHg) controlled BP (vs. tight (85 mmHg) controlled BP,, Ethnicity ( Black/HIspanic vs. Caucasian/Asian/Other), BMI (≥ 25 vs <25), gestational age (per wk increase), Folic acid or PNV,aspirin, in hospital, preexisting (vs. gestational) Hypertension, sBP within last week, dBP within last week, prior sBP ≥ 150 or dBP ≥ 110 mmHg in this pregnancy, Methyldopa (+others, no labetalol), other antihypertensive drugs, no antihypertensive drugs | preterm birth <34 weeks | 978 | NA (14.10) | NA | NA | NA | NA | NA | NA | 0.71 (0.66–0.75) |
| Magee et al (2021), multinational | pre-eclampsia definition A (development of proteinuria) in chronic hypertension | preterm birth <34 weeks | 728 | 94 (12.86) | 0.49 (0.39-0.59) | 0.77 (0.73-0.80) | 0.24 (0.18-0.30) | 0.91 (0.88-0.93) | 2.11 (1.64-2.71) | 0.66 (0.54-0.81) | 0.63 (0.58-0.68) |
| Magee et al (2021), multinational | pre-eclampsia definition B (development of proteinuria AND one or more symptoms, signs, abnormal laboratory test) in chronic hypertension | preterm birth <34 weeks | 728 | 94 (12.86) | 0.33 (0.24-0.43) | 0.89 (0.86-0.91) | 0.31 (0.23-0.40) | 0.90 (0.87-0.92) | 2.99 (2.08-4.29) | 0.75 (0.65-0.87) | 0.61 (0.56-0.66) |
| Magee et al (2021), multinational | pre-eclampsia definition C (one or more symptoms, signs) in chronic hypertension | preterm birth <34 weeks | 728 | 94 (12.86) | 0.62 (0.52-0.71) | 0.72 (0.68-0.75) | 0.25 (0.20-0.31) | 0.93 (0.90-0.95) | 2.21 (1.80-2.71) | 0.53 (0.41-0.69) | 0.67 (0.62-0.72) |
| Magee et al (2021), multinational | pre-eclampsia definition D (one or more symptoms, signs, abnormal laboratory test) in chronic hypertension | preterm birth <34 weeks | 728 | 94 (12.86) | 0.64 (0.54-0.73) | 0.71 (0.68-0.75) | 0.25 (0.20-0.31) | 0.93 (0.90-0.95) | 2.24 (1.84-2.72) | 0.51 (0.39-0.67) | 0.68 (0.62-0.73) |
| Magee et al (2021), multinational | pre-eclampsia definition E (development of proteinuria OR one or more symptoms, signs, abnormal laboratory test) in chronic hypertension | preterm birth <34 weeks | 728 | 94 (12.86) | 0.80 (0.71-0.87) | 0.59 (0.55-0.63) | 0.23 (0.18-0.27) | 0.95 (0.93-0.97) | 1.96 (1.71-2.25) | 0.34 (0.23-0.51) | 0.70 (0.65-0.74) |
| Magee et al (2021), multinational | pre-eclampsia definition F (development of proteinuria development of proteinuria OR one or more symptoms, signs) in chronic hypertension | preterm birth <34 weeks | 728 | 94 (12.86) | 0.49 (0.68-0.85) | 0.60 (0.56-0.64) | 0.22 (0.18-0.27) | 0.95 (0.92-0.97) | 1.93 (1.67-2.23) | 0.37 (0.25-0.55) | 0.69 (0.64-0.73) |
| Magee et al (2021), multinational | pre-eclampsia definition A (development of proteinuria) in gestational hypertension | preterm birth <34 weeks | 248 | 43 (17.34) | 0.49 (0.35-0.63) | 0.68 (0.62-0.74) | 0.24 (0.17-0.34) | 0.86 (0.80-0.91) | 1.54 (1.07-2.22) | 0.75 (0.55-1.02) | 0.59 (0.50-0.67) |
| Magee et al (2021), multinational | pre-eclampsia definition B (development of proteinuria AND one or more symptoms, signs, abnormal laboratory test) in gestational hypertension | preterm birth <34 weeks | 248 | 43 (17.34) | 0.35 (0.22-0.50) | 0.81 (0.76-0.86) | 0.28 (0.18-0.42) | 0.86 (0.80-0.90) | 1.88 (1.14-3.10) | 0.80 (0.64-1.00) | 0.58 (0.50-0.66) |
| Magee et al (2021), multinational | pre-eclampsia definition C (one or more symptoms, signs) in gestational hypertension | preterm birth <34 weeks | 248 | 43 (17.34) | 0.63 (0.48-0.76) | 0.63 (0.56-0.69) | 0.26 (0.19-0.35) | 0.89 (0.83-0.93) | 1.69 (1.27-2.27) | 0.59 (0.40-0.88) | 0.63 (0.55-0.71) |
| Magee et al (2021), multinational | pre-eclampsia definition D (one or more symptoms, signs, abnormal laboratory test) in gestational hypertension | preterm birth <34 weeks | 248 | 43 (17.34) | 0.63 (0.48-0.76) | 0.62 (0.56-0.69) | 0.26 (0.18-0.35) | 0.89 (0.83-0.93) | 1.67 (1.25-2.23) | 0.60 (0.40-0.89) | 0.63 (0.55-0.71) |
| Magee et al (2021), multinational | pre-eclampsia definition E (development of proteinuria OR one or more symptoms, signs, abnormal laboratory test) in gestational hypertension | preterm birth <34 weeks | 248 | 43 (17.34) | 0.77 (0.62-0.87) | 0.49 (0.43-0.56) | 0.24 (0.18-0.32) | 0.91 (0.84-0.95) | 1.51 (1.22-1.87) | 0.47 (0.27-0.83) | 0.63 (0.56-0.70) |
| Magee et al (2021), multinational | pre-eclampsia definition F (development of proteinuria development of proteinuria OR one or more symptoms, signs) in gestational hypertension | preterm birth <34 weeks | 248 | 43 (17.34) | 0.77 (0.62-0.87) | 0.49 (0.43-0.56) | 0.24 (0.18-0.32) | 0.91 (0.84-0.95) | 1.51 (1.22-1.87) | 0.47 (0.27-0.83) | 0.63 (0.56-0.70) |
| Mayama et al (2021), Japan | mild thrombocytopenia platelet < 150x10^9/L | preterm birth <34 weeks | 264 | 100 (37.87) | 0.34 (0.25-0.44) | 0.75 (0.68-0.81) | 0.45 (0.35-0.57) | 0.65 (0.58-0.72) | 1.36 (0.93-1.99) | 0.88 (0.75-1.04) | 0.54 (0.49-0.60) |
| Mayama et al (2021), Japan | severe thrombocytopenia platelet < 100x10^9/L | preterm birth <34 weeks | 264 | 100 (37.87) | 0.16 (0.10-0.24) | 0.95 (0.91-0.98) | 0.67 (0.47-0.82) | 0.65 (0.59-0.71) | 3.28 (1.46-7.38) | 0.88 (0.81-0.97) | 0.56 (0.52-0.60) |
| Tokalioglu et al (2023), Turkey | umbilical artery half peak systolic velocity deceleration time (UA hPSV-DT) <5th percentile | preterm birth <34 weeks | 55 | 12 (21.82) | 0.83 (0.55-0.95) | 0.72 (0.57-0.83) | 0.45 (0.27-0.65) | 0.94 (0.80-0.98) | 2.99 (1.74-5.14) | 0.23 (0.06-0.83) | 0.78 (0.65-0.91) |
| Webster et al (2018), UK | Ethnicity: White | preterm birth <34 weeks | 4,481 | 305 (6.81) | 0.25 (0.20-0.30) | 0.51 (0.49-0.53) | 0.04 (0.03-0.04) | 0.90 (0.89-0.91) | 0.51 (0.42-0.62) | 1.47 (1.37-1.58) | 0.38 (0.35-0.41) |
| Webster et al (2018), UK | Ethnicity: Black | preterm birth <34 weeks | 4,481 | 305 (6.81) | 0.56 (0.50-0.61) | 0.66 (0.64-0.67) | 0.11 (0.09-0.12) | 0.95 (0.94-0.96) | 1.63 (1.46-1.81) | 0.67 (0.59-0.77) | 0.61 (0.58-0.64) |
| Webster et al (2018), UK | Ethnicity: Asian | preterm birth <34 weeks | 4,481 | 305 (6.81) | 0.11 (0.08-0.16) | 0.92 (0.91-0.93) | 0.09 (0.07-0.13) | 0.93 (0.93-0.94) | 1.39 (1.00-1.93) | 0.96 (0.93-1.01) | 0.52 (0.50-0.53) |
| Chaves et al (2017), Brazil | abnormal PR (ratio of the flow velocity of the second peak to that of the initial systolic velocity peak) ≥ 0.78 | preterm birth < 32 weeks | 56 | 15 (26.79) | 0.93 (0.70-0.99) | 0.15 (0.07-0.28) | 0.29 (0.18-0.42) | 0.86 (0.49-0.97) | 1.09 (0.91-1.32) | 0.46 (0.06-3.48) | 0.54 (0.45-0.63) |
| Chaves et al (2017), Brazil | abnormal PR (ratio of the flow velocity of the second peak to that of the initial systolic velocity peak) ≥ 0.99 | preterm birth < 32 weeks | 56 | 15 (26.79) | 0.47 (0.25-0.70) | 0.78 (0.63-0.88) | 0.44 (0.23-0.67) | 0.80 (0.65-0.90) | 2.13 (0.96-4.69) | 0.68 (0.41-1.13) | 0.62 (0.48-0.77) |
| Cai et al (2021), China | urinary congophilia (CapCord Test) | iatrogenic prematurity (delivery before 37 weeks) | 102 | 86 (84.31) | 0.93 (0.86-0.97) | 0.56 (0.33-0.77) | 0.92 (0.84-0.96) | 0.60 (0.36-0.80) | 2.13 (1.22-3.72) | 0.12 (0.05-0.30) | 0.75 (0.62-0.87) |
| **Low weight** | | | | | | | | | | | |
| Abraham et al (2019), India | TOH ≤ 264 days | low birth weight (less than 2500 g) | 174 | NA (58.05) | 0.89 | 0.66 | NA | NA | NA | NA | 0.836 (0.772–0.900) |
| Abraham et al (2019), India | DBP ≥ 98.5 mmHg | low birth weight (less than 2500 g) | 174 | NA (58.05) | 0.77 | 0.60 | NA | NA | NA | NA | 0.738 (0.66–0.810) |
| Abraham et al (2019), India | TOH (days) + DBP (mm/Hg) | low birth weight (less than 2500 g) | 174 | NA (58.05) | 0.87 | 0.74 | NA | NA | NA | NA | 0.852 (0.792–0.912) |
| Chadha et al (2022), India | UPCR ≥ 0.3 | low birth weight (less than 2500 g) | 130 | 90 (63.80) | 0.96 (0.89-0.98) | 0.22 (0.12-0.35) | 0.68 (0.60-0.76) | 0.73 (0.48-0.89) | 1.22 (1.05-1.42) | 0.21 (0.07-0.61) | 0.59 (0.52-0.65) |
| Jampana et al (2022), India | maternal serum uric acid level > 5.5 mg/dL | low birth weight (less than 2500 g) | 86 | 62 (72.10) | 0.77 (0.66-0.86) | 0.88 (0.69-0.96) | 0.94 (0.84-0.98) | 0.60 (0.44-0.74) | 6.19 (2.13-18.00) | 0.26 (0.16-0.42) | 0.82 (0.74-0.91) |
| Joshi et al (2022), India | raised serum hs-CRP > 3.0 mg/mL | low birth weight (less than 2500 g) | 132 | 47 (35.61) | 0.96 (0.86-0.99) | 0.82 (0.73-0.89) | 0.75 (0.63-0.84) | 0.97 (0.90-0.99) | 5.43 (3.41-8.62) | 0.05 (0.01-0.20) | 0.89 (0.84-0.94) |
| Madhu et al (2023), India | increase in UA S/D ratio | low birth weight (less than 2500 g) | 150 | 74 (49.33) | 0.28 (0.19-0.40) | 0.83 (0.73-0.90) | 0.62 (0.45-0.76) | 0.54 (0.45-0.63) | 1.66 (0.90-3.06) | 0.86 (0.72-1.03) | 0.56 (0.49-0.62) |
| Madhu et al (2023), India | elevated UA RI | low birth weight (less than 2500 g) | 150 | 74 (49.33) | 0.15 (0.09-0.25) | 0.87 (0.77-0.93) | 0.52 (0.32-0.72) | 0.51 (0.43-0.60) | 1.13 (0.51-2.50) | 0.98 (0.86-1.12) | 0.51 (0.45-0.56) |
| Madhu et al (2023), India | elevated UA PI | low birth weight (less than 2500 g) | 150 | 74 (49.33) | 0.26 (0.17-0.37) | 0.84 (0.74-0.91) | 0.61 (0.44-0.76) | 0.54 (0.45-0.62) | 1.63 (0.85-3.11) | 0.88 (0.75-1.04) | 0.55 (0.48-0.61) |
| Madhu et al (2023), India | elevated MCA PI | low birth weight (less than 2500 g) | 150 | 74 (49.33) | 0.08 (0.04-0.17) | 0.93 (0.86-0.97) | 0.55 (0.28-0.79) | 0.51 (0.43-0.59) | 1.23 (0.39-3.86) | 0.98 (0.90-1.08) | 0.51 (0.47-0.55) |
| Madhu et al (2023), India | abnormal CPR <1.08 | low birth weight (less than 2500 g) | 150 | 74 (49.33) | 0.65 (0.54-0.75) | 0.30 (0.21-0.41) | 0.48 (0.38-0.57) | 0.47 (0.34-0.61) | 0.93 (0.74-1.16) | 1.16 (0.73-1.84) | 0.48 (0.40-0.55) |
| Madhu et al (2023), India | UA reversed end diastolic flow | low birth weight (less than 2500 g) | 150 | 74 (49.33) | 0.03 (0.01-0.09) | 0.99 (0.93-1.00) | 0.67 (0.21-0.94) | 0.51 (0.43-0.59) | 2.05 (0.19-22.17) | 0.99 (0.94-1.03) | 0.51 (0.48-0.53) |
| Madhu et al (2023), India | increase in UA S/D ratio | low birth weight (less than 2500 g) | 150 | 74 (49.33) | 0.28 (0.19-0.40) | 0.83 (0.73-0.90) | 0.62 (0.45-0.76) | 0.54 (0.45-0.63) | 1.66 (0.90-3.06) | 0.86 (0.72-1.03) | 0.56 (0.49-0.62) |
| Tokalioglu et al (2023), Turkey | umbilical artery half peak systolic velocity deceleration time (UA hPSV-DT) <5th percentile | low birth weight (less than 2500 g) | 55 | 17 (30.91) | 0.82 (0.59-0.94) | 0.79 (0.64-0.89) | 0.64 (0.43-0.80) | 0.91 (0.76-0.97) | 3.91 (2.03-7.52) | 0.22 (0.08-0.63) | 0.81 (0.69-0.92) |
| Lei et al (2021), China | 24 h Proteinuria excretion < 0.3 g/24h | low birth weight (including light for date infant and premature low weight infants) | 275 | 28 (10.20) | 0.21 (0.10-0.40) | 0.81 (0.76-0.85) | 0.11 (0.05-0.23) | 0.90 (0.85-0.93) | 1.13 (0.53-2.39) | 0.97 (0.79-1.19) | 0.51 (0.43-0.59) |
| Lei et al (2021), China | 24 h Proteinuria excretion ≥ 0.3 g/24h | low birth weight (including light for date infant and premature low weight infants) | 275 | 28 (10.20) | 0.29 (0.15-0.47) | 0.56 (0.50-0.62) | 0.07 (0.04-0.13) | 0.87 (0.81-0.92) | 0.65 (0.35-1.18) | 1.28 (0.99-1.66) | 0.42 (0.33-0.51) |
| Lei et al (2021), China | 24 h Proteinuria excretion ≥ 2.0 g/24h | low birth weight (including light for date infant and premature low weight infants) | 275 | 28 (10.20) | 0.14 (0.06-0.31) | 0.80 (0.75-0.85) | 0.08 (0.03-0.18) | 0.89 (0.84-0.93) | 0.72 (0.28-1.85) | 1.07 (0.91-1.26) | 0.47 (0.40-0.54) |
| Ryu et al (2019), Korea | serum uric acid level ≥ 6.35 mg/dL | low birth weight | 65 | NA (61.50) | 0.58 | 0.95 | NA | NA | NA | NA | 0.808 (0.700–0.916) |
| Ryu et al (2019), Korea | hemoglobin | low birth weight | 65 | NA (61.50) | NA | NA | NA | NA | NA | NA | 0.709 (0.573–0.845) |
| Ryu et al (2019), Korea | low serum total bilirubin | low birth weight | 65 | NA (61.50) | NA | NA | NA | NA | NA | NA | 0.660 (0.527–0.793) |
| Ryu et al (2019), Korea | serum uric acid levels, hemoglobin, low serum total bilirubin | low birth weight | 65 | NA (61.50) | NA | NA | NA | NA | NA | NA | 0.902 (0.817–0.986) |
| Saxena et al (2021), India | CPR < 1 | low birth weight | 150 | 97 (64.67) | 0.93 (0.78-0.99) | 0.42 (0.34-0.52) | 0.29 (0.22-0.39) | 0.96 (0.87-1.00) | 1.63 (1.47-1.81) | 0.15 (0.07-0.32) | 0.68 (0.65-0.72) |
| Sudjai et al (2022), Thailand | serum uric acid level < 5.0 mg/dL | low birth weight | 400 | 186 (46.50) | 0.35 (0.29-0.43) | 0.86 (0.81-0.90) | 0.69 (0.59-0.77) | 0.61 (0.55-0.66) | 2.53 (1.72-3.72) | 0.75 (0.67-0.85) | 0.61 (0.57-0.65) |
| Sudjai et al (2022), Thailand | serum uric acid level ≥ 5.0 mg/dL | low birth weight | 400 | 186 (46.50) | 0.65 (0.57-0.71) | 0.14 (0.10-0.19) | 0.39 (0.34-0.45) | 0.31 (0.23-0.41) | 0.75 (0.67-0.85) | 2.53 (1.72-3.72) | 0.39 (0.35-0.43) |
| Sudjai et al (2022), Thailand | serum uric acid level ≥ 7.0 mg/dL | low birth weight | 400 | 186 (46.50) | 0.20 (0.15-0.26) | 0.59 (0.53-0.66) | 0.30 (0.22-0.38) | 0.46 (0.40-0.52) | 0.49 (0.35-0.68) | 1.35 (1.18-1.54) | 0.40 (0.35-0.44) |
| Zarean et al (2022), Iran | CPR < 1 | low birth weight | 100 | 12 (12.00) | 0.58 (0.32-0.81) | 0.77 (0.67-0.85) | 0.26 (0.13-0.45) | 0.93 (0.85-0.97) | 2.57 (1.39-4.74) | 0.54 (0.27-1.06) | 0.68 (0.53-0.83) |
| Zheng et al (2022), China | multivariable model: Logistic regression (with imputation) | low birth weight | 733 | 253 (34.52) | 0.78 (0.72-0.83) | 0.87 (0.84-0.90) | 0.77 (0.71-0.81) | 0.88 (0.85-0.91) | 6.03 (4.72-7.68) | 0.25 (0.20-0.32) | 0.82 (0.79-0.85) |
| Chaiworapongsa et al (2023), US | abnormal angiogenic profile (sFlt-1/PlGF ratio < 10th percentile for gestational age) in early-onset pre-eclampsia (cohort case-control) | low birth weight < 10 % | 29 | 16 (55.17) | 0.94 (0.72-0.99) | 0.15 (0.04-0.42) | 0.58 (0.39-0.74) | 0.67 (0.21-0.94) | 1.11 (0.85-1.44) | 0.41 (0.04-4.00) | 0.55 (0.43-0.66) |
| Chaiworapongsa et al (2023), US | abnormal angiogenic profile (sFlt-1/PlGF ratio < 10th percentile for gestational age) in intermediate pre-eclampsia (cohort case-control) | low birth weight < 10 % | 16 | 6 (37.50) | 1.00 (0.61-1.00) | 0.00 (0.00-0.28) | 0.38 (0.18-0.61) | NA | 0.97 (0.76-1.24)* | 1.57 (0.04-70.50)* | 0.50 (0.50-0.50) |
| Chaiworapongsa et al (2023), US | abnormal angiogenic profile (sFlt-1/PlGF ratio < 10th percentile for gestational age) in term pre-eclampsia (cohort case-control) | low birth weight < 10 % | 106 | 23 (21.70) | 0.70 (0.49-0.84) | 0.70 (0.59-0.79) | 0.39 (0.26-0.54) | 0.89 (0.79-0.95) | 2.31 (1.51-3.53) | 0.44 (0.23-0.82) | 0.70 (0.59-0.81) |
| Chaiworapongsa et al (2023), US | abnormal angiogenic profile (sFlt-1/PlGF ratio < 10th percentile for gestational age) in early-onset pre-eclampsia (cohort case-series) | low birth weight < 10 % | 89 | 30 (33.71) | 1.00 (0.89-1.00) | 0.03 (0.01-0.12) | 0.34 (0.25-0.45) | 1.00 (0.34-1.00) | 1.03 (0.96-1.10)* | 0.39 (0.02-7.82)* | 0.52 (0.49-0.54) |
| Chaiworapongsa et al (2023), US | abnormal angiogenic profile (sFlt-1/PlGF ratio < 10th percentile for gestational age) in intermediate pre-eclampsia (cohort case-series) | low birth weight < 10 % | 105 | 42 (40.00) | 0.83 (0.69-0.92) | 0.22 (0.14-0.34) | 0.42 (0.32-0.52) | 0.67 (0.45-0.83) | 1.07 (0.89-1.29) | 0.75 (0.33-1.70) | 0.53 (0.45-0.60) |
| Chaiworapongsa et al (2023), US | abnormal angiogenic profile (sFlt-1/PlGF ratio < 10th percentile for gestational age) in term pre-eclampsia (cohort case-series) | low birth weight < 10 % | 258 | 70 (27.13) | 0.77 (0.66-0.85) | 0.54 (0.47-0.61) | 0.38 (0.31-0.47) | 0.86 (0.79-0.91) | 1.67 (1.36-2.04) | 0.43 (0.27-0.67) | 0.65 (0.59-0.72) |
| Heimberger et al (2020), US | sFlt-1/PlGF ratio ≥ 85 | low birth weight < 10 % | 115 | 8 (6.96) | 0.62 (0.31-0.86) | 0.70 (0.61-0.78) | 0.14 (0.06-0.28) | 0.96 (0.89-0.99) | 2.09 (1.14-3.85) | 0.54 (0.22-1.32) | 0.66 (0.48-0.85) |
| Magee et al (2016), multinational | controlled BP ≤ 85 mmHg | low birth weight < 10 % | 976 | 175 (17.90) | 0.45 (0.38-0.53) | 0.49 (0.45-0.52) | 0.16 (0.13-0.20) | 0.80 (0.76-0.84) | 0.88 (0.74-1.05) | 1.13 (0.97-1.31) | 0.47 (0.43-0.51) |
| Magee et al (2016), multinational | controlled BP ≤ 100 mmHg | low birth weight < 10 % | 976 | 175 (17.90) | 0.55 (0.47-0.62) | 0.51 (0.48-0.55) | 0.20 (0.16-0.24) | 0.84 (0.80-0.87) | 1.13 (0.97-1.31) | 0.88 (0.74-1.05) | 0.53 (0.49-0.57) |
| Magee et al (2016), multinational | age ≥ 35 years | low birth weight < 10 % | 976 | 175 (17.90) | 0.50 (0.43-0.58) | 0.58 (0.55-0.62) | 0.21 (0.17-0.25) | 0.84 (0.81-0.87) | 1.21 (1.02-1.43) | 0.85 (0.73-1.00) | 0.54 (0.50-0.58) |
| Magee et al (2016), multinational | Ethnicity: Caucasian/Asian/Other | low birth weight < 10 % | 976 | 175 (17.90) | 0.75 (0.69-0.81) | 0.25 (0.22-0.28) | 0.18 (0.15-0.21) | 0.82 (0.77-0.87) | 1.00 (0.91-1.10) | 0.99 (0.74-1.32) | 0.50 (0.47-0.54) |
| Magee et al (2016), multinational | Ethnicity: Black/Hispanic | low birth weight < 10 % | 976 | 175 (17.90) | 0.25 (0.19-0.31) | 0.75 (0.72-0.78) | 0.18 (0.13-0.23) | 0.82 (0.79-0.85) | 0.99 (0.74-1.32) | 1.00 (0.91-1.10) | 0.50 (0.46-0.53) |
| Magee et al (2016), multinational | BMI ≥ 25 kg/m^2 | low birth weight < 10 % | 967 | 174 (17.90) | 0.66 (0.58-0.72) | 0.21 (0.18-0.24) | 0.15 (0.13-0.18) | 0.74 (0.68-0.79) | 0.83 (0.74-0.93) | 1.63 (1.27-2.08) | 0.43 (0.40-0.47) |
| Magee et al (2016), multinational | conceived through ART | low birth weight < 10 % | 959 | 173 (17.90) | 0.05 (0.02-0.09) | 0.96 (0.94-0.97) | 0.19 (0.10-0.33) | 0.82 (0.79-0.84) | 1.07 (0.50-2.27) | 1.00 (0.96-1.03) | 0.50 (0.48-0.52) |
| Magee et al (2016), multinational | gestational age ≥ 20 weeks | low birth weight < 10 % | 976 | 175 (17.90) | 0.70 (0.63-0.76) | 0.30 (0.27-0.33) | 0.18 (0.15-0.21) | 0.82 (0.77-0.86) | 1.00 (0.90-1.11) | 1.01 (0.79-1.29) | 0.50 (0.46-0.54) |
| Magee et al (2016), multinational | nulliparous | low birth weight < 10 % | 976 | 175 (17.90) | 0.39 (0.32-0.47) | 0.68 (0.64-0.71) | 0.21 (0.17-0.26) | 0.84 (0.81-0.86) | 1.22 (0.99-1.51) | 0.89 (0.79-1.02) | 0.54 (0.50-0.58) |
| Magee et al (2016), multinational | gestational hypertension | low birth weight < 10 % | 976 | 175 (17.90) | 0.30 (0.24-0.37) | 0.76 (0.72-0.78) | 0.21 (0.17-0.27) | 0.83 (0.80-0.86) | 1.24 (0.96-1.60) | 0.92 (0.83-1.03) | 0.53 (0.49-0.57) |
| Magee et al (2016), multinational | preexisting hypertension | low birth weight < 10 % | 976 | 175 (17.90) | 0.70 (0.63-0.76) | 0.24 (0.22-0.28) | 0.17 (0.14-0.20) | 0.79 (0.73-0.83) | 0.92 (0.83-1.03) | 1.24 (0.96-1.60) | 0.47 (0.43-0.51) |
| Magee et al (2016), multinational | prior SBP ≥ 160 or DBP ≥ 110 mmHg in this pregnancy | low birth weight < 10 % | 976 | 175 (17.90) | 0.15 (0.10-0.21) | 0.86 (0.83-0.88) | 0.19 (0.13-0.26) | 0.82 (0.79-0.85) | 1.04 (0.70-1.55) | 0.99 (0.93-1.06) | 0.50 (0.47-0.53) |
| Magee et al (2016), multinational | antihypertensive use at randomization | low birth weight < 10 % | 976 | 175 (17.90) | 0.56 (0.49-0.63) | 0.43 (0.39-0.46) | 0.18 (0.15-0.21) | 0.82 (0.78-0.85) | 0.98 (0.84-1.13) | 1.03 (0.86-1.24) | 0.49 (0.45-0.53) |
| Magee et al (2016), multinational | labetalol (+other, no methyldopa) | low birth weight < 10 % | 976 | 175 (17.90) | 0.29 (0.23-0.36) | 0.77 (0.74-0.80) | 0.22 (0.17-0.28) | 0.83 (0.80-0.86) | 1.28 (0.98-1.66) | 0.92 (0.83-1.02) | 0.53 (0.49-0.57) |
| Magee et al (2016), multinational | methyldopa (+other, no labetaol) | low birth weight < 10 % | 976 | 175 (17.90) | 0.17 (0.12-0.23) | 0.74 (0.71-0.77) | 0.12 (0.09-0.17) | 0.80 (0.77-0.83) | 0.65 (0.46-0.92) | 1.12 (1.04-1.22) | 0.45 (0.42-0.49) |
| Magee et al (2016), multinational | other antihypertensive drugs | low birth weight < 10 % | 976 | 175 (17.90) | 0.10 (0.06-0.15) | 0.92 (0.90-0.93) | 0.20 (0.13-0.30) | 0.82 (0.80-0.85) | 1.16 (0.70-1.93) | 0.99 (0.93-1.04) | 0.51 (0.48-0.53) |
| Magee et al (2016), multinational | SBP within 1 week before randomization < 140 mmHg | low birth weight < 10 % | 976 | 175 (17.90) | 0.42 (0.35-0.50) | 0.63 (0.59-0.66) | 0.20 (0.16-0.24) | 0.83 (0.80-0.86) | 1.13 (0.93-1.38) | 0.92 (0.80-1.06) | 0.52 (0.48-0.57) |
| Magee et al (2016), multinational | SBP within 1 week before randomization ≥ 140 mmHg | low birth weight < 10 % | 976 | 175 (17.90) | 0.58 (0.50-0.65) | 0.37 (0.34-0.41) | 0.17 (0.14-0.20) | 0.80 (0.76-0.84) | 0.92 (0.80-1.06) | 1.13 (0.93-1.38) | 0.48 (0.43-0.52) |
| Magee et al (2016), multinational | SBP within 1 week before randomization ≥ 150 mmHg | low birth weight < 10 % | 976 | 175 (17.90) | 0.25 (0.19-0.31) | 0.79 (0.76-0.82) | 0.21 (0.16-0.27) | 0.83 (0.80-0.85) | 1.19 (0.88-1.59) | 0.95 (0.87-1.04) | 0.52 (0.48-0.55) |
| Magee et al (2016), multinational | DBP within 1 week before randomization < 90 mmHg | low birth weight < 10 % | 976 | 175 (17.90) | 0.18 (0.13-0.24) | 0.81 (0.78-0.83) | 0.17 (0.12-0.23) | 0.82 (0.79-0.84) | 0.92 (0.65-1.31) | 1.02 (0.94-1.10) | 0.49 (0.46-0.52) |
| Magee et al (2016), multinational | DBP within 1 week before randomization ≥ 90 mmHg | low birth weight < 10 % | 976 | 175 (17.90) | 0.82 (0.76-0.87) | 0.19 (0.17-0.22) | 0.18 (0.16-0.21) | 0.83 (0.77-0.88) | 1.02 (0.94-1.10) | 0.92 (0.65-1.31) | 0.51 (0.48-0.54) |
| Magee et al (2016), multinational | DBP within 1 week before randomization ≥ 95 mmHg | low birth weight < 10 % | 976 | 175 (17.90) | 0.38 (0.31-0.45) | 0.62 (0.58-0.65) | 0.18 (0.14-0.22) | 0.82 (0.79-0.85) | 0.98 (0.80-1.21) | 1.01 (0.89-1.15) | 0.50 (0.46-0.54) |
| Magee et al (2016), multinational | DBP within 1 week before randomization ≥ 100 mmHg | low birth weight < 10 % | 976 | 175 (17.90) | 0.15 (0.11-0.22) | 0.88 (0.85-0.90) | 0.22 (0.15-0.30) | 0.83 (0.80-0.85) | 1.27 (0.86-1.89) | 0.96 (0.90-1.03) | 0.52 (0.49-0.55) |
| Magee et al (2016), multinational | in hospital at enrolment | low birth weight < 10 % | 976 | 175 (17.90) | 0.10 (0.07-0.16) | 0.95 (0.93-0.96) | 0.30 (0.20-0.43) | 0.83 (0.80-0.85) | 1.96 (1.16-3.32) | 0.95 (0.90-1.00) | 0.53 (0.50-0.55) |
| Magee et al (2016), multinational | GDM prior to random | low birth weight < 10 % | 976 | 175 (17.90) | 0.06 (0.03-0.10) | 0.93 (0.91-0.95) | 0.16 (0.09-0.27) | 0.82 (0.79-0.84) | 0.86 (0.45-1.66) | 1.01 (0.97-1.05) | 0.50 (0.48-0.51) |
| Magee et al (2016), multinational | smoking during this pregnancy | low birth weight < 10 % | 976 | 175 (17.90) | 0.10 (0.07-0.16) | 0.94 (0.93-0.96) | 0.29 (0.19-0.41) | 0.83 (0.80-0.85) | 1.83 (1.09-3.08) | 0.95 (0.90-1.00) | 0.52 (0.50-0.55) |
| Magee et al (2016), multinational | aspirin at enrolment | low birth weight < 10 % | 976 | 175 (17.90) | 0.20 (0.15-0.27) | 0.72 (0.69-0.75) | 0.14 (0.10-0.18) | 0.81 (0.78-0.83) | 0.72 (0.53-1.00) | 1.10 (1.01-1.20) | 0.46 (0.43-0.50) |
| Magee et al (2016), multinational | folic acid or PNV vitamin at enrolment | low birth weight < 10 % | 975 | 175 (17.90) | 0.62 (0.54-0.69) | 0.34 (0.31-0.38) | 0.17 (0.14-0.20) | 0.80 (0.76-0.84) | 0.94 (0.83-1.07) | 1.12 (0.91-1.38) | 0.48 (0.44-0.52) |
| Magee et al (2016), multinational | PMR recruiting country ≥ 10 perinatal death/100 births | low birth weight < 10 % | 976 | 175 (17.90) | 0.17 (0.12-0.23) | 0.84 (0.81-0.86) | 0.18 (0.13-0.25) | 0.82 (0.79-0.85) | 1.02 (0.71-1.47) | 1.00 (0.93-1.07) | 0.50 (0.47-0.53) |
| Magee et al (2016), multinational | multivariable model: less tight (100 mmHg) controlled BP (vs. tight (85 mmHg) controlled BP, Maternal age (<35 vs ≥ 35), Nulliparous, smoking, aspirin, in hospital, SBP within last week, DBP within last week, methyldopa (+others, no labetalol), other antihypertensive drugs, no antihypertensive drugs | low birth weight < 10 % | 976 | NA (17.90) | NA | NA | NA | NA | NA | NA | 0.66 (0.62–0.71) |
| Magee et al (2021), multinational | pre-eclampsia definition A (development of proteinuria) in chronic hypertension | low birth weight < 10 % | 728 | 121 (16.55) | 0.35 (0.27-0.44) | 0.75 (0.72-0.78) | 0.22 (0.17-0.28) | 0.85 (0.82-0.88) | 1.40 (1.05-1.85) | 0.87 (0.76-1.00) | 0.55 (0.50-0.60) |
| Magee et al (2021), multinational | pre-eclampsia definition B (development of proteinuria AND one or more symptoms, signs, abnormal laboratory test) in chronic hypertension | low birth weight < 10 % | 728 | 121 (16.55) | 0.13 (0.08-0.20) | 0.86 (0.83-0.89) | 0.16 (0.10-0.24) | 0.83 (0.80-0.86) | 0.94 (0.57-1.55) | 1.01 (0.93-1.09) | 0.50 (0.46-0.53) |
| Magee et al (2021), multinational | pre-eclampsia definition C (one or more symptoms, signs) in chronic hypertension | low birth weight < 10 % | 728 | 121 (16.55) | 0.35 (0.27-0.44) | 0.68 (0.64-0.72) | 0.18 (0.14-0.23) | 0.84 (0.80-0.87) | 1.09 (0.83-1.43) | 0.96 (0.83-1.10) | 0.51 (0.47-0.56) |
| Magee et al (2021), multinational | pre-eclampsia definition D (one or more symptoms, signs, abnormal laboratory test) in chronic hypertension | low birth weight < 10 % | 728 | 121 (16.55) | 0.36 (0.28-0.44) | 0.67 (0.64-0.71) | 0.18 (0.14-0.23) | 0.84 (0.80-0.87) | 1.09 (0.84-1.42) | 0.96 (0.83-1.10) | 0.51 (0.47-0.56) |
| Magee et al (2021), multinational | pre-eclampsia definition E (development of proteinuria OR one or more symptoms, signs, abnormal laboratory test) in chronic hypertension | low birth weight < 10 % | 728 | 121 (16.55) | 0.57 (0.48-0.65) | 0.57 (0.53-0.60) | 0.21 (0.17-0.25) | 0.87 (0.83-0.90) | 1.31 (1.10-1.57) | 0.76 (0.61-0.94) | 0.57 (0.52-0.62) |
| Magee et al (2021), multinational | pre-eclampsia definition F (development of proteinuria development of proteinuria OR one or more symptoms, signs) in chronic hypertension | low birth weight < 10 % | 728 | 121 (16.55) | 0.56 (0.47-0.65) | 0.57 (0.53-0.61) | 0.21 (0.17-0.25) | 0.87 (0.83-0.90) | 1.31 (1.09-1.57) | 0.77 (0.62-0.95) | 0.57 (0.52-0.62) |
| Magee et al (2021), multinational | pre-eclampsia definition A (development of proteinuria) in gestational hypertension | low birth weight < 10 % | 248 | 52 (20.97) | 0.38 (0.26-0.52) | 0.66 (0.59-0.73) | 0.23 (0.16-0.33) | 0.80 (0.73-0.86) | 1.14 (0.77-1.70) | 0.93 (0.73-1.18) | 0.52 (0.45-0.60) |
| Magee et al (2021), multinational | pre-eclampsia definition B (development of proteinuria AND one or more symptoms, signs, abnormal laboratory test) in gestational hypertension | low birth weight < 10 % | 248 | 52 (20.97) | 0.27 (0.17-0.40) | 0.80 (0.74-0.85) | 0.26 (0.16-0.40) | 0.81 (0.74-0.85) | 1.35 (0.80-2.30) | 0.91 (0.76-1.09) | 0.54 (0.47-0.60) |
| Magee et al (2021), multinational | pre-eclampsia definition C (one or more symptoms, signs) in gestational hypertension | low birth weight < 10 % | 248 | 52 (20.97) | 0.62 (0.48-0.74) | 0.64 (0.57-0.70) | 0.31 (0.23-0.41) | 0.86 (0.80-0.91) | 1.70 (1.28-2.26) | 0.60 (0.42-0.86) | 0.63 (0.55-0.70) |
| Magee et al (2021), multinational | pre-eclampsia definition D (one or more symptoms, signs, abnormal laboratory test) in gestational hypertension | low birth weight < 10 % | 248 | 52 (20.97) | 0.62 (0.48-0.74) | 0.63 (0.56-0.70) | 0.31 (0.23-0.40) | 0.86 (0.80-0.91) | 1.68 (1.26-2.22) | 0.61 (0.42-0.87) | 0.62 (0.55-0.70) |
| Magee et al (2021), multinational | pre-eclampsia definition E (development of proteinuria OR one or more symptoms, signs, abnormal laboratory test) in gestational hypertension | low birth weight < 10 % | 248 | 52 (20.97) | 0.73 (0.60-0.83) | 0.49 (0.43-0.56) | 0.28 (0.21-0.36) | 0.87 (0.80-0.92) | 1.45 (1.17-1.79) | 0.54 (0.34-0.87) | 0.61 (0.54-0.68) |
| Magee et al (2021), multinational | pre-eclampsia definition F (development of proteinuria development of proteinuria OR one or more symptoms, signs) in gestational hypertension | low birth weight < 10 % | 248 | 52 (20.97) | 0.73 (0.60-0.83) | 0.49 (0.43-0.56) | 0.28 (0.21-0.36) | 0.87 (0.80-0.92) | 1.45 (1.17-1.79) | 0.54 (0.34-0.87) | 0.61 (0.54-0.68) |
| Tousty et al (2022), Poland | sFlt-1/PlGF ratio ≥ 204 | low birth weight < 10 % | 77 | 20 (25.97) | 0.65 (0.43-0.82) | 0.75 (0.63-0.85) | 0.48 (0.31-0.66) | 0.86 (0.74-0.93) | 2.65 (1.52-4.62) | 0.46 (0.25-0.86) | 0.70 (0.58-0.82) |
| Tousty et al (2022), Poland | gestational age at delivery ≤ 32 weeks | low birth weight < 10 % | 77 | 20 (25.97) | 0.40 (0.22-0.61) | 0.79 (0.67-0.88) | 0.40 (0.22-0.61) | 0.79 (0.67-0.88) | 1.90 (0.91-3.96) | 0.76 (0.52-1.11) | 0.59 (0.47-0.72) |
| Webster et al (2018), UK | Ethnicity: White | low birth weight < 10 % | 4,481 | 1047 (23.37) | 0.37 (0.34-0.40) | 0.50 (0.48-0.51) | 0.18 (0.17-0.20) | 0.72 (0.70-0.74) | 0.74 (0.68-0.80) | 1.27 (1.20-1.34) | 0.43 (0.42-0.45) |
| Webster et al (2018), UK | Ethnicity: Black | low birth weight < 10 % | 4,481 | 1047 (23.37) | 0.47 (0.44-0.50) | 0.68 (0.66-0.69) | 0.31 (0.29-0.33) | 0.81 (0.79-0.82) | 1.46 (1.34-1.58) | 0.78 (0.74-0.83) | 0.57 (0.56-0.59) |
| Webster et al (2018), UK | Ethnicity: Asian | low birth weight < 10 % | 4,481 | 1047 (23.37) | 0.08 (0.06-0.10) | 0.91 (0.90-0.92) | 0.22 (0.18-0.26) | 0.76 (0.75-0.78) | 0.91 (0.72-1.14) | 1.01 (0.99-1.03) | 0.50 (0.49-0.51) |
| Karge et al (2022), Germany | BMI ≥ 25 kg/m^2 | birth weight < 3rd centile | 141 | 13 (9.22) | 0.31 (0.13-0.58) | 0.59 (0.50-0.67) | 0.07 (0.03-0.17) | 0.89 (0.81-0.94) | 0.74 (0.32-1.72) | 1.18 (0.80-1.75) | 0.45 (0.31-0.58) |
| Karge et al (2022), Germany | BMI ≥ 30 kg/m^2 | birth weight < 3rd centile | 141 | 13 (9.22) | 0.15 (0.04-0.42) | 0.80 (0.72-0.86) | 0.07 (0.02-0.23) | 0.90 (0.83-0.94) | 0.76 (0.20-2.84) | 1.06 (0.83-1.36) | 0.48 (0.37-0.58) |
| Tousty et al (2022), Poland | sFlt-1/PlGF ratio ≥ 204 | birth weight < 3rd centile | 77 | 9 (11.69) | 0.78 (0.45-0.94) | 0.71 (0.59-0.80) | 0.26 (0.13-0.45) | 0.96 (0.87-0.99) | 2.64 (1.59-4.39) | 0.31 (0.09-1.08) | 0.74 (0.59-0.90) |
| Tousty et al (2022), Poland | gestational age at delivery ≤ 32 weeks | birth weight < 3rd centile | 77 | 9 (11.69) | 0.44 (0.19-0.73) | 0.76 (0.65-0.85) | 0.20 (0.08-0.42) | 0.91 (0.81-0.96) | 1.89 (0.81-4.41) | 0.73 (0.40-1.32) | 0.60 (0.43-0.78) |
| Webster et al (2018), UK | Ethnicity: White | birth weight < 3rd centile | 4,481 | 499 (11.14) | 0.31 (0.28-0.36) | 0.51 (0.49-0.52) | 0.07 (0.06-0.09) | 0.86 (0.84-0.87) | 0.64 (0.56-0.73) | 1.35 (1.27-1.45) | 0.41 (0.39-0.43) |
| Webster et al (2018), UK | Ethnicity: Black | birth weight < 3rd centile | 4,481 | 499 (11.14) | 0.50 (0.45-0.54) | 0.66 (0.65-0.67) | 0.15 (0.14-0.17) | 0.91 (0.90-0.92) | 1.46 (1.33-1.61) | 0.76 (0.70-0.83) | 0.58 (0.56-0.60) |
| Webster et al (2018), UK | Ethnicity: Asian | birth weight < 3rd centile | 4,481 | 499 (11.14) | 0.09 (0.07-0.12) | 0.92 (0.91-0.92) | 0.12 (0.09-0.16) | 0.89 (0.88-0.90) | 1.10 (0.82-1.48) | 0.99 (0.96-1.02) | 0.50 (0.49-0.52) |
| Graupner et al (2019), Germany | sFlt-1/PlGF ratio ≥ 110 | SGA (< 3rd centile) | 67 | 8 (11.90) | 0.88 (0.53-0.98) | 0.66 (0.53-0.77) | 0.26 (0.13-0.45) | 0.97 (0.87-1.00) | 2.58 (1.66-4.02) | 0.19 (0.03-1.19) | 0.77 (0.63-0.90) |
| Graupner et al (2019), Germany | CPR < 5th centile | SGA (< 3rd centile) | 67 | 8 (11.90) | 0.38 (0.14-0.69) | 0.93 (0.84-0.97) | 0.43 (0.16-0.75) | 0.92 (0.82-0.96) | 5.53 (1.50-20.34) | 0.67 (0.39-1.15) | 0.65 (0.47-0.84) |
| Graupner et al (2019), Germany | mean UtA-PI > 95th centile | SGA (< 3rd centile) | 67 | 8 (11.90) | 0.75 (0.41-0.93) | 0.71 (0.59-0.81) | 0.26 (0.13-0.46) | 0.95 (0.85-0.99) | 2.60 (1.48-4.59) | 0.35 (0.10-1.18) | 0.73 (0.56-0.90) |
| Graupner et al (2019), Germany | sFlt/PlGF ratio + CPR OR mean UtA-PI | SGA (< 3rd centile) | 67 | NA (11.90) | NA | NA | NA | NA | NA | NA | 0.886 (0.79–0.98) |
| Chaves et al (2017), Brazil | abnormal PR (ratio of the flow velocity of the second peak to that of the initial systolic velocity peak) ≥ 0.78 | SGA (< 10th percentile) | 56 | 24 (42.86) | 0.96 (0.80-0.99) | 0.19 (0.09-0.35) | 0.47 (0.34-0.61) | 0.86 (0.49-0.97) | 1.18 (0.98-1.42) | 0.22 (0.03-1.73) | 0.57 (0.49-0.65) |
| Chaves et al (2017), Brazil | abnormal PR (ratio of the flow velocity of the second peak to that of the initial systolic velocity peak) ≥ 0.99 | SGA (< 10th percentile) | 56 | 24 (42.86) | 0.38 (0.21-0.57) | 0.78 (0.61-0.89) | 0.56 (0.33-0.77) | 0.62 (0.47-0.76) | 1.71 (0.74-3.95) | 0.80 (0.56-1.15) | 0.58 (0.46-0.70) |
| Malik et al (2023), India | CPR < 1.08 | SGA (birth weight < 10th percentile for gestational age) | 100 | 28 (28.00) | 0.86 (0.69-0.94) | 0.89 (0.80-0.94) | 0.75 (0.58-0.87) | 0.94 (0.86-0.98) | 7.71 (3.95-15.08) | 0.16 (0.06-0.40) | 0.87 (0.80-0.95) |
| Fishel Bartal et al (2022), US | Ethnicity: Non-Hispanic White | SGA | 388,609 | 47879 (12.32) | 0.39 (0.39-0.40) | 0.48 (0.48-0.48) | 0.10 (0.10-0.10) | 0.85 (0.85-0.85) | 0.76 (0.75-0.77) | 1.26 (1.25-1.27) | 0.44 (0.44-0.44) |
| Fishel Bartal et al (2022), US | Ethnicity: Non-Hispanic Black | SGA | 388,609 | 47879 (12.32) | 0.41 (0.40-0.41) | 0.72 (0.72-0.72) | 0.17 (0.17-0.17) | 0.90 (0.90-0.90) | 1.45 (1.44-1.47) | 0.82 (0.82-0.83) | 0.56 (0.56-0.57) |
| Fishel Bartal et al (2022), US | Ethnicity: Hispanic | SGA | 388,609 | 47879 (12.32) | 0.14 (0.14-0.14) | 0.84 (0.84-0.84) | 0.11 (0.11-0.11) | 0.87 (0.87-0.88) | 0.88 (0.86-0.90) | 1.02 (1.02-1.03) | 0.49 (0.49-0.49) |
| Fishel Bartal et al (2022), US | Ethnicity: Non-Hispanic Asian American, Native Hawaiian and other Pacific Islander | SGA | 388,609 | 47879 (12.32) | 0.05 (0.05-0.05) | 0.97 (0.97-0.97) | 0.18 (0.17-0.18) | 0.88 (0.88-0.88) | 1.55 (1.48-1.61) | 0.98 (0.98-0.98) | 0.51 (0.51-0.51) |
| Fishel Bartal et al (2022), US | Ethnicity: Non-Hispanic American Indian and Alaska Native | SGA | 388,609 | 47879 (12.32) | 0.01 (0.01-0.01) | 0.99 (0.99-0.99) | 0.10 (0.09-0.11) | 0.88 (0.88-0.88) | 0.82 (0.74-0.90) | 1.00 (1.00-1.00) | 0.50 (0.50-0.50) |
| Leanos-Miranda et el (2020), Mexico | sFlt-1/PlGF ratio ≥ 38 | SGA | 810 | 354 (43.70) | 0.90 (0.86-0.93) | 0.54 (0.50-0.59) | 0.60 (0.56-0.65) | 0.87 (0.83-0.91) | 1.97 (1.77-2.19) | 0.19 (0.14-0.26) | 0.72 (0.69-0.75) |
| Leanos-Miranda et el (2020), Mexico | sFlt-1/PlGF ratio ≥ 85 | SGA | 810 | 354 (43.70) | 0.80 (0.75-0.84) | 0.69 (0.64-0.73) | 0.67 (0.62-0.71) | 0.81 (0.77-0.85) | 2.56 (2.21-2.96) | 0.30 (0.24-0.37) | 0.74 (0.71-0.77) |
| Ryu et al (2019), Korea | serum uric acid level ≥ 6.35 mg/dL | SGA | 65 | NA (43.10) | 0.52 | 0.73 | NA | NA | NA | NA | 0.63 |
| Stolz et al (2018), Austria | sFlt-1/PlGF ratio ≥ 655 | SGA | 54 | 14 (23.30) | 0.64 (0.39-0.84) | 0.55 (0.40-0.69) | 0.33 (0.19-0.52) | 0.81 (0.63-0.92) | 1.43 (0.85-2.40) | 0.65 (0.30-1.38) | 0.60 (0.44-0.75) |
| Ye et al (2020), China | retinopathy staging Stage 3/4 | SGA | 452 | 69 (15.30) | 0.19 (0.11-0.30) | 0.87 (0.83-0.90) | 0.21 (0.13-0.33) | 0.86 (0.82-0.89) | 1.47 (0.85-2.57) | 0.93 (0.83-1.05) | 0.53 (0.48-0.58) |
| Zarean et al (2022), Iran | CPR < 1 | SGA | 100 | NA | 0.26 | 0.93 | 0.58 | 0.77 | NA | NA | NA |
| Chadha et al (2022), India | UPCR ≥ 0.3 | FGR (birth weight below the 10th percentile) | 141 | 52 (36.90) | 0.96 (0.87-0.99) | 0.15 (0.09-0.23) | 0.40 (0.32-0.48) | 0.87 (0.62-0.96) | 1.13 (1.02-1.25) | 0.26 (0.06-1.12) | 0.55 (0.51-0.60) |
| Chaves et al (2017), Brazil | abnormal PR (ratio of the flow velocity of the second peak to that of the initial systolic velocity peak) ≥ 0.78 | FGR (birth weight below the 10th percentile) | 56 | 27 (48.21) | 0.93 (0.77-0.98) | 0.17 (0.08-0.35) | 0.51 (0.37-0.64) | 0.71 (0.36-0.92) | 1.12 (0.92-1.36) | 0.43 (0.09-2.03) | 0.55 (0.46-0.64) |
| Chaves et al (2017), Brazil | abnormal PR (ratio of the flow velocity of the second peak to that of the initial systolic velocity peak) ≥ 0.99 | FGR (birth weight below the 10th percentile) | 56 | 27 (48.21) | 0.41 (0.25-0.59) | 0.83 (0.65-0.92) | 0.69 (0.44-0.86) | 0.60 (0.45-0.74) | 2.36 (0.94-5.92) | 0.72 (0.50-1.02) | 0.62 (0.50-0.74) |
| Karge et al (2021), Germany | early-onset pre-eclampsia | FGR | 49 | 7 (14.29) | 0.57 (0.25-0.84) | 0.67 (0.52-0.79) | 0.22 (0.09-0.45) | 0.90 (0.75-0.97) | 1.71 (0.79-3.71) | 0.64 (0.27-1.55) | 0.62 (0.41-0.83) |
| Karge et al (2021), Germany | late-onset pre-eclampsia | FGR | 49 | 7 (14.29) | 0.43 (0.16-0.75) | 0.33 (0.21-0.48) | 0.10 (0.03-0.25) | 0.78 (0.55-0.91) | 0.64 (0.27-1.55) | 1.71 (0.79-3.71) | 0.38 (0.17-0.59) |
| Karge et al (2022), Germany | BMI ≥ 25 kg/m^2 | FGR | 141 | 36 (25.53) | 0.33 (0.20-0.50) | 0.57 (0.48-0.66) | 0.21 (0.12-0.33) | 0.71 (0.61-0.80) | 0.78 (0.47-1.30) | 1.17 (0.88-1.55) | 0.45 (0.36-0.54) |
| Karge et al (2022), Germany | BMI ≥ 30 kg/m^2 | FGR | 141 | 36 (25.53) | 0.14 (0.06-0.29) | 0.78 (0.69-0.85) | 0.18 (0.08-0.36) | 0.73 (0.64-0.80) | 0.63 (0.26-1.54) | 1.10 (0.93-1.30) | 0.46 (0.39-0.53) |
| Kesireddy et al (2021), India | GlyFn > 350 µg/mL | FGR | 51 | 28 (54.90) | 0.79 (0.60-0.90) | 0.65 (0.45-0.81) | 0.73 (0.56-0.86) | 0.71 (0.50-0.86) | 2.26 (1.25-4.08) | 0.33 (0.15-0.71) | 0.72 (0.59-0.85) |
| Loardi et al (2021), Italy | abnormal UtA PI > 95th percentile | FGR | 311 | 139 (44.69) | 0.85 (0.78-0.90) | 0.60 (0.52-0.67) | 0.63 (0.56-0.70) | 0.83 (0.75-0.89) | 2.12 (1.74-2.57) | 0.25 (0.17-0.38) | 0.72 (0.68-0.77) |
| Madhu et al (2023), India | increase in UA S/D ratio | IUGR | 150 | 33 (22.00) | 0.30 (0.17-0.47) | 0.79 (0.71-0.86) | 0.29 (0.17-0.46) | 0.80 (0.72-0.86) | 1.48 (0.79-2.77) | 0.88 (0.69-1.12) | 0.55 (0.46-0.64) |
| Madhu et al (2023), India | elevated UA RI | IUGR | 150 | 33 (22.00) | 0.12 (0.05-0.27) | 0.85 (0.78-0.91) | 0.19 (0.08-0.40) | 0.78 (0.70-0.84) | 0.83 (0.30-2.31) | 1.03 (0.89-1.19) | 0.49 (0.42-0.55) |
| Madhu et al (2023), India | elevated UA PI | IUGR | 150 | 33 (22.00) | 0.24 (0.13-0.41) | 0.80 (0.72-0.87) | 0.26 (0.14-0.43) | 0.79 (0.71-0.85) | 1.23 (0.61-2.50) | 0.94 (0.76-1.17) | 0.52 (0.44-0.61) |
| Madhu et al (2023), India | elevated MCA PI | IUGR | 150 | 33 (22.00) | 0.03 (0.01-0.15) | 0.91 (0.85-0.95) | 0.09 (0.02-0.38) | 0.77 (0.69-0.83) | 0.35 (0.05-2.67) | 1.06 (0.98-1.15) | 0.47 (0.43-0.51) |
| Madhu et al (2023), India | abnormal CPR <1.08 | IUGR | 150 | 33 (22.00) | 0.73 (0.56-0.85) | 0.34 (0.26-0.43) | 0.24 (0.17-0.33) | 0.82 (0.69-0.90) | 1.11 (0.86-1.41) | 0.80 (0.43-1.47) | 0.53 (0.45-0.62) |
| Madhu et al (2023), India | UA reversed end diastolic flow | IUGR | 150 | 33 (22.00) | 0.00 (0.00-0.10) | 0.97 (0.93-0.99) | 0.00 (0.00-0.56) | 0.78 (0.70-0.84) | 0.50 (0.03-9.36)* | 1.02 (0.96-1.07)* | 0.49 (0.47-0.50) |
| Moawad et al (2022), Egypt | UA PI > 95th percentile | IUGR (fetal abdominal circumference or estimated fetal weight <10th percentile and umbilical Doppler PI >95th percentile) | 60 | 19 (31.70) | 0.58 (0.36-0.77) | 0.71 (0.55-0.82) | 0.48 (0.29-0.67) | 0.78 (0.62-0.88) | 1.93 (1.05-3.55) | 0.60 (0.34-1.06) | 0.64 (0.50-0.77) |
| Moawad et al (2022), Egypt | UA RI > 95th percentile | IGUR (fetal abdominal circumference or estimated fetal weight <10th percentile and umbilical Doppler PI >95th percentile) | 60 | 19 (31.70) | 0.68 (0.46-0.85) | 0.71 (0.56-0.82) | 0.52 (0.33-0.70) | 0.83 (0.67-0.92) | 2.34 (1.33-4.11) | 0.45 (0.22-0.89) | 0.70 (0.57-0.82) |
| Moawad et al (2022), Egypt | MCA PI < 5th percentile | IUGR (fetal abdominal circumference or estimated fetal weight <10th percentile and umbilical Doppler PI >95th percentile) | 60 | 19 (31.70) | 0.53 (0.32-0.73) | 0.71 (0.55-0.82) | 0.46 (0.27-0.65) | 0.76 (0.60-0.87) | 1.75 (0.93-3.32) | 0.68 (0.40-1.13) | 0.61 (0.48-0.75) |
| Moawad et al (2022), Egypt | MCA RI < 5th percentile | IGUR (fetal abdominal circumference or estimated fetal weight <10th percentile and umbilical Doppler PI >95th percentile) | 60 | 19 (31.70) | 0.53 (0.32-0.73) | 0.71 (0.55-0.82) | 0.46 (0.27-0.65) | 0.76 (0.60-0.87) | 1.75 (0.93-3.32) | 0.68 (0.40-1.13) | 0.61 (0.48-0.75) |
| Moawad et al (2022), Egypt | CPR< 1 | IUGR (fetal abdominal circumference or estimated fetal weight <10th percentile and umbilical Doppler PI >95th percentile) | 60 | 19 (31.70) | 0.79 (0.57-0.91) | 0.61 (0.46-0.74) | 0.48 (0.32-0.65) | 0.86 (0.69-0.95) | 2.02 (1.29-3.17) | 0.35 (0.14-0.85) | 0.70 (0.58-0.82) |
| Sudjai et al (2022), Thailand | serum uric acid level < 5.0 mg/dL | IUGR | 400 | 15 (3.75) | 0.07 (0.01-0.30) | 0.75 (0.71-0.79) | 0.01 (0.00-0.06) | 0.95 (0.92-0.97) | 0.27 (0.04-1.81) | 1.24 (1.07-1.44) | 0.41 (0.34-0.48) |
| Sudjai et al (2022), Thailand | serum uric acid level ≥ 5.0 mg/dL | IUGR | 400 | 15 (3.75) | 0.93 (0.70-0.99) | 0.25 (0.21-0.29) | 0.05 (0.03-0.08) | 0.99 (0.94-1.00) | 1.24 (1.07-1.44) | 0.27 (0.04-1.81) | 0.59 (0.52-0.66) |
| Sudjai et al (2022), Thailand | serum uric acid level ≥ 7.0 mg/dL | IUGR | 400 | 15 (3.75) | 0.47 (0.25-0.70) | 0.70 (0.65-0.74) | 0.06 (0.03-0.11) | 0.97 (0.94-0.99) | 1.54 (0.88-2.69) | 0.77 (0.48-1.24) | 0.58 (0.45-0.71) |
| Confidence intervals for the sensitivity, specificity, positive and negative predictive value were calculated using the Wilson's score method. Confidence intervals for the positive and negative likelihood ratios were calculated using Simel's method. Confidence intervals for AUROC were calculated using the method of DeLong. * 0.5 pseudo count added to each cell in the confusion matrix (true positives, false positives, true negatives, and false negatives) to avoid division by zero. **Abbreviations:** ADMA, asymmetric dimethyl arginine; ART, assisted reproductive technology; AUROC, area under the receiver operating characteristic curve; BDA, bronchopulmonary dysplasia; BMV, requirement of bag and mask ventilation; BP, blood pressure; CPAP, requirement of continuous positive airway pressure; CPR, cerebroplacental ratio; DBP, diastolic blood pressure; FGR, fetal growth restriction; GDM, gestational diabetes mellitus; GlyFn, glycosylated fibronectin; hs-CRP, high-sensitive C-reactive protein; IUGR, intrauterine growth restriction; IVH, intraventricular haemorrhage; LR-, negative likelihood ratio; LR+, positive likelihood ratio; MCA, middle cerebral artery; NA, not available; NEC, necrotizing enterocolitis; NICU, neonatal intensive care unit; NPV, negative predictive value; NST, nonstress test; PDA, patent ductus arteriosus; PI, pulsatility index; PlGF, placental growth factor; PMR, perinatal mortality ratio; PNV, prenatal vitamin; PPV, positive predictive value; PR, peak ratio; RDS, respiratory distress syndrome; RI, resistance index; ROP, retinopathy of prematurity; SBP, systolic blood pressure; S/D, systolic/diastolic; sFlt-1, soluble fms-like tyrosine kinase-1; SGA, small for gestational age; TAS, total antioxidant status; TOH, time of onset of hypertension; UA, Umbilical artery; UPCR, urinary protein to creatinine ratio; UtA, uterine artery. | | | | | | | | | | | |

**Supplementary table 11: Predictive performance of all prediction tests for composite perinatal outcomes.**

|  | | | | | **2x2 Table** | | | |  | | | | | | |
| --- | --- | --- | --- | --- | --- | --- | --- | --- | --- | --- | --- | --- | --- | --- | --- |
| **Study** | **Predictor** | **Outcome** | **Sample size** | **Even rate n (%)** | **FP** | **TP** | **TN** | **FN** | **Sensitivity** | **Specificity** | **PPV** | **NPV** | **LR+** | **LR-** | **AUROC** |
| **maternal characteristics and preexisting disease** | | | | | | | | | | | | | | | |
| Magee et al (2016), multinational | maternal age ≥ 35 | pregnancy loss NICU admission | 987 | 307 (31.10) | 288 | 137 | 388 | 168 | NA | NA | NA | NA | NA | NA | NA |
| Magee et al (2016), multinational | BMI ≥ 25 | pregnancy loss NICU admission | 987 | 307 (31.10) | 522 | 221 | 148 | 81 | NA | NA | NA | NA | NA | NA | NA |
| Magee et al (2016), multinational | conceived through ART | pregnancy loss NICU admission | 987 | 307 (31.10) | 21 | 21 | 643 | 279 | NA | NA | NA | NA | NA | NA | NA |
| Magee et al (2016), multinational | nulliparous | pregnancy loss NICU admission | 987 | 307 (31.10) | 207 | 122 | 469 | 183 | NA | NA | NA | NA | NA | NA | NA |
| Thangaratinam et al (2017), UK | twin pregnancy | infant death BPD NEC  IVH Cystic periventricular leukomalacia ROP  HIE | 945 | 702 (74.29) | 17 | 62 | 226 | 640 | NA | NA | NA | NA | NA | NA | NA |
| Thangaratinam et al (2017), UK | triplet pregnancy | infant death BPD NEC  IVH Cystic periventricular leukomalacia ROP  HIE | 945 | 702 (74.29) | 1 | 3 | 242 | 699 | NA | NA | NA | NA | NA | NA | NA |
| Magee et al (2016), multinational | gestational age ≥ 20 weeks | pregnancy loss NICU admission | 987 | 307 (31.10) | 467 | 216 | 209 | 89 | NA | NA | NA | NA | NA | NA | NA |
| Tousty et al (2022), Poland | gestational age at delivery | Congenital or late-onset infections RDS PDA NEC IVH ROP BPD infant death | 77 | 22 (28.57) | 3 | 17 | 52 | 5 | 0.77 | NA | 0.95 | NA | NA | NA | 0.91 |
| Fishel Bartal et al (2022), US | Ethnicity: Non–Hispanic White | preterm birth (< 37 weeks) SGA 5 min Apgar score < 5  ventilation  neonatal seizures neonatal death | 388750 | 121640 (31.29) | 143739 | 51411 | 122998 | 70602 | NA | NA | NA | NA | NA | NA | NA |
| Fishel Bartal et al (2022), US | Ethnicity: Non–Hispanic Black | preterm birth (< 37 weeks) SGA 5 min Apgar score < 5  ventilation  neonatal seizures neonatal death | 388750 | 121640 (31.29) | 70277 | 45027 | 196460 | 76986 | NA | NA | NA | NA | NA | NA | NA |
| Fishel Bartal et al (2022), US | Ethnicity: Hispanic | preterm birth (< 37 weeks) SGA 5 min Apgar score < 5  ventilation  neonatal seizures neonatal death | 388750 | 121640 (31.29) | 41171 | 19057 | 225566 | 102956 | NA | NA | NA | NA | NA | NA | NA |
| Fishel Bartal et al (2022), US | Ethnicity: Non–Hispanic Asian American, Native Hawaiian and other Pacific Islander | preterm birth (< 37 weeks) SGA 5 min Apgar score < 5  ventilation  neonatal seizures neonatal death | 388750 | 121640 (31.29) | 8724 | 5138 | 258013 | 116875 | NA | NA | NA | NA | NA | NA | NA |
| Fishel Bartal et al (2022), US | Ethnicity: Non–Hispanic American Indian and Alaska Native | preterm birth (< 37 weeks) SGA 5 min Apgar score < 5  ventilation  neonatal seizures neonatal death | 388750 | 121640 (31.29) | 2826 | 1380 | 263911 | 120633 | NA | NA | NA | NA | NA | NA | NA |
| Magee et al (2016), multinational | Ethnicity: Caucasian/Asian/Other | pregnancy loss NICU admission | 987 | 307 (31.10) | 505 | 233 | 171 | 72 | NA | NA | NA | NA | NA | NA | NA |
| Magee et al (2016), multinational | Ethnicity: Black/Hispanic | pregnancy loss NICU admission | 987 | 307 (31.10) | 171 | 72 | 505 | 233 | NA | NA | NA | NA | NA | NA | NA |
| Thangaratinam et al (2017), UK | single pregnancy | infant death BPD NEC  IVH Cystic periventricular leukomalacia ROP  HIE | 945 | 702 (74.29) | 225 | 637 | 18 | 65 | NA | NA | NA | NA | NA | NA | NA |
| Magee et al (2016), multinational | hospital enrolment | pregnancy loss NICU admission | 987 | 307 (31.10) | 21 | 40 | 655 | 265 | NA | NA | NA | NA | NA | NA | NA |
| Magee et al (2016), multinational | gestational diabetes mellitus | pregnancy loss NICU admission | 987 | 307 (31.10) | 44 | 19 | 632 | 286 | NA | NA | NA | NA | NA | NA | NA |
| Magee et al (2016), multinational | smoking during the pregnancy | pregnancy loss NICU admission | 987 | 307 (31.10) | 38 | 25 | 638 | 280 | NA | NA | NA | NA | NA | NA | NA |
| Thangaratinam et al (2017), UK | history of none condition | infant death BPD NEC  IVH Cystic periventricular leukomalacia ROP  HIE | 945 | 702 (74.29) | 116 | 478 | 127 | 223 | NA | NA | NA | NA | NA | NA | NA |
| Thangaratinam et al (2017), UK | history of one condition | infant death BPD NEC  IVH Cystic periventricular leukomalacia ROP  HIE | 945 | 702 (74.29) | 83 | 167 | 160 | 534 | NA | NA | NA | NA | NA | NA | NA |
| Thangaratinam et al (2017), UK | history of two or more condition | infant death BPD NEC  IVH Cystic periventricular leukomalacia ROP  HIE | 945 | 702 (74.29) | 44 | 56 | 199 | 645 | NA | NA | NA | NA | NA | NA | NA |
| Magee et al (2016), multinational | Folic acid or PNV vitamin at enrolment | pregnancy loss NICU admission | 987 | 307 (31.10) | 450 | 188 | 225 | 117 | NA | NA | NA | NA | NA | NA | NA |
| Magee et al (2016), multinational | preexisting hypertension | pregnancy loss NICU admission | 987 | 307 (31.10) | 514 | 218 | 162 | 87 | NA | NA | NA | NA | NA | NA | NA |
| Magee et al (2016), multinational | antihypertensive use | pregnancy loss NICU admission | 987 | 307 (31.10) | 368 | 194 | 308 | 111 | NA | NA | NA | NA | NA | NA | NA |
| Magee et al (2016), multinational | other antihypertensive use | pregnancy loss NICU admission | 987 | 307 (31.10) | 50 | 34 | 626 | 271 | NA | NA | NA | NA | NA | NA | NA |
| Thangaratinam et al (2017), UK | any antihypertensive therapy | infant death BPD NEC  IVH Cystic periventricular leukomalacia ROP  HIE | 945 | 702 (74.29) | 177 | 573 | 66 | 128 | NA | NA | NA | NA | NA | NA | NA |
| Magee et al (2016), multinational | aspirin at enrolment | pregnancy loss NICU admission | 987 | 307 (31.10) | 178 | 79 | 498 | 226 | NA | NA | NA | NA | NA | NA | NA |
| Magee et al (2016), multinational | Labetalol (+ other, no methyldopa) | pregnancy loss NICU admission | 987 | 307 (31.10) | 144 | 93 | 532 | 212 | NA | NA | NA | NA | NA | NA | NA |
| Magee et al (2016), multinational | Methyldopa (+ other, no labetalol) | pregnancy loss NICU admission | 987 | 307 (31.10) | 174 | 67 | 502 | 238 | NA | NA | NA | NA | NA | NA | NA |
| Thangaratinam et al (2017), UK | magnesium sulfate administered | infant death BPD NEC  IVH Cystic periventricular leukomalacia ROP  HIE | 945 | 702 (74.29) | 9 | 135 | 234 | 566 | NA | NA | NA | NA | NA | NA | NA |
| Thangaratinam et al (2017), UK | steroids administered | infant death BPD NEC  IVH Cystic periventricular leukomalacia ROP  HIE | 945 | 702 (74.29) | 66 | 364 | 177 | 176 | NA | NA | NA | NA | NA | NA | NA |
| **blood pressure measurement** | | | | | | | | | | | | | | | |
| Magee et al (2016), multinational | DBP within 1 week before randomization < 90 mmHg | pregnancy loss NICU admission | 987 | 307 (31.10) | 135 | 53 | 541 | 252 | NA | NA | NA | NA | NA | NA | NA |
| Magee et al (2016), multinational | DBP within 1 week before randomization 90–94 mmHg | pregnancy loss NICU admission | 987 | 307 (31.10) | 340 | 145 | 336 | 160 | NA | NA | NA | NA | NA | NA | NA |
| Magee et al (2016), multinational | DBP within 1 week before randomization 95–99 mmHg | pregnancy loss NICU admission | 987 | 307 (31.10) | 131 | 53 | 545 | 252 | NA | NA | NA | NA | NA | NA | NA |
| Magee et al (2016), multinational | DBP within 1 week before randomization ≥ 100 mmHg | pregnancy loss NICU admission | 987 | 307 (31.10) | 70 | 54 | 606 | 251 | NA | NA | NA | NA | NA | NA | NA |
| Thangaratinam et al (2017), UK | DBP | infant death BPD NEC  IVH Cystic periventricular leukomalacia ROP  HIE | 945 | 702 (74.29) | 96 | 101 | 147 | 597 | NA | NA | NA | NA | NA | NA | NA |
| Magee et al (2016), multinational | SBP within 1 week before randomization < 140 mmHg | pregnancy loss NICU admission | 987 | 307 (31.10) | 272 | 104 | 404 | 201 | NA | NA | NA | NA | NA | NA | NA |
| Magee et al (2016), multinational | SBP within 1 week before randomization 140–149 mmHg | pregnancy loss NICU admission | 987 | 307 (31.10) | 266 | 130 | 410 | 175 | NA | NA | NA | NA | NA | NA | NA |
| Magee et al (2016), multinational | SBP within 1 week before randomization ≥ 150 mmHg | pregnancy loss NICU admission | 987 | 307 (31.10) | 138 | 71 | 538 | 234 | NA | NA | NA | NA | NA | NA | NA |
| Thangaratinam et al (2017), UK | SBP | infant death BPD NEC  IVH Cystic periventricular leukomalacia ROP  HIE | 945 | 702 (74.29) | 153 | 161 | 90 | 537 | NA | NA | NA | NA | NA | NA | NA |
| Simón et al (2020), Spain | MAP | perinatal death bronchopulmonary dysplasia IVH NEC ROP | 76 | 13 (17.11) | NA | NA | NA | NA | 0 (0.0-0.14) | 0.9 | 0 | 0.65 (0.63-0.67) | 0 | NA | 0.40 (0.22-0.58) |
| Kumar et al (2023), India | MAP ≥ 127.85 mmHg | preterm birth at ≤34 week  NICU admission RDS HIE NEC ROP neonatal seizures perinatal death | 91 | 39 (42.90) | NA | NA | NA | NA | 0.66 | 0.67 | NA | NA | NA | NA | NA |
| Magee et al (2016), multinational | controlled BP ≤ 100 mmHg | pregnancy loss NICU admission | 987 | 307 (31.10) | 338 | 155 | 338 | 150 | NA | NA | NA | NA | NA | NA | NA |
| Magee et al (2016), multinational | controlled BP ≤ 85 mmHg | pregnancy loss NICU admission | 987 | 307 (31.10) | 338 | 150 | 338 | 155 | NA | NA | NA | NA | NA | NA | NA |
| **proteinuria** | | | | | | | | | | | | | | | |
| Lei et al (2021), China | 24 h Proteinuria excretion < 0.3 g/24h | death stillbirth low birth weight preterm birth (<37 weeks) induced labor FGR fetal distress neonatal asphyxia neonatal hypoxic encephalopathy fetal malformation | 275 | 124 (45.10) | 37 | 16 | 114 | 108 | NA | NA | NA | NA | NA | NA | NA |
| Lei et al (2021), China | 24 h Proteinuria excretion 0.3-2.0 g/24h | death stillbirth low birth weight preterm birth (<37 weeks) induced labor FGR fetal distress neonatal asphyxia neonatal hypoxic encephalopathy fetal malformation | 275 | 124 (45.10) | 45 | 19 | 106 | 105 | NA | NA | NA | NA | NA | NA | NA |
| Lei et al (2021), China | 24 h Proteinuria excretion ≥ 2.0 g/24h | death stillbirth low birth weight preterm birth (<37 weeks) induced labor FGR fetal distress neonatal asphyxia neonatal hypoxic encephalopathy fetal malformation | 275 | 124 (45.10) | 17 | 36 | 134 | 88 | NA | NA | NA | NA | NA | NA | NA |
| Murali et al (2023), India | 24 h Proteinuria excretion ≥ 905 mg/24h | FGR stillbirth 5 min Apgar score < 7  NICU admission RDS HIE intracranial bleed sepsis neonatal death | 202 | 153 (75.74) | NA | NA | NA | NA | 0.74 | 0.51 | NA | NA | NA | NA | NA |
| Chadha et al (2022), India | UPCR ≥ 0.3 | preterm birth (<37 weeks)  FGR Low birth weight < 2500 g Intrauterine death Neonatal death | 141 | NA (NA) | NA | NA | NA | NA | 0.77 (0.69-0.84) | 0.13(0.02-0.40) | 0.88(0.86-0.90) | 0.06 (0.02-0.21) | NA | NA | 0.515 (0.403–0.627) |
| Murali et al (2023), India | UPCR ≥ 1.60 mg/mg | FGR stillbirth 5 min Apgar score < 7  NICU admission RDS HIE intracranial bleed sepsis neonatal death | 202 | 153 (75.74) | NA | NA | NA | NA | 0.73 | 0.53 | NA | NA | NA | NA | NA |
| Simón et al (2020), Spain | UPCR ≥ 4.8 mg/mg | perinatal death bronchopulmonary dysplasia IVH NEC ROP | 76 | 13 (17.11) | NA | NA | NA | NA | 0.10 (0.03-0.31) | 0.9 | 0.38 (0.14-0.70) | 0.68 (0.64-0.71) | 1.2 (0.3-4.7) | NA | 0.38 (0.19–0.58) |
| Thangaratinam et al (2017), UK | urine dipstick cutoff 1+ | infant death BPD NEC  IVH Cystic periventricular leukomalacia ROP  HIE | 945 | 702 (74.29) | 71 | 98 | 172 | 586 | NA | NA | NA | NA | NA | NA | NA |
| Thangaratinam et al (2017), UK | urine dipstick cutoff 2+ | infant death BPD NEC  IVH Cystic periventricular leukomalacia ROP  HIE | 945 | 702 (74.29) | 100 | 212 | 143 | 472 | NA | NA | NA | NA | NA | NA | NA |
| Thangaratinam et al (2017), UK | urine dipstick cutoff 3+ | infant death BPD NEC  IVH Cystic periventricular leukomalacia ROP  HIE | 945 | 702 (74.29) | 42 | 261 | 201 | 423 | NA | NA | NA | NA | NA | NA | NA |
| Thangaratinam et al (2017), UK | urine dipstick cutoff >/= 4 | infant death BPD NEC  IVH Cystic periventricular leukomalacia ROP  HIE | 945 | 702 (74.29) | 13 | 91 | 230 | 593 | NA | NA | NA | NA | NA | NA | NA |
| **angiogenic biomarkers** | | | | | | | | | | | | | | | |
| Karge et al (2022), Germany | serum PlGF | SGA neonatal mortality neonatal seizures NICU admission  respiratory support | 141 | 69 (48.94) | NA | NA | NA | NA | NA | NA | NA | NA | NA | NA | 0.866 (0.807–0.925) |
| Kumar et al (2023), India | serum PlGF ≥ 1680.0 pg/mL | preterm birth at ≤34 week  NICU admission RDS HIE NEC ROP neonatal seizures perinatal death | 91 | 39 (42.90) | NA | NA | NA | NA | 0.97 | 0.02 | NA | NA | NA | NA | 0.18 (0.09-0.27) |
| Reddy et al (2022), Australia | serum PlGF | preterm birth (< 34 weeks) NICU admission  RDS IVH HIE NEC confirmed fetal infection ROB | 126 | 48 (38.10) | NA | NA | NA | NA | 0.50 (0.13-0.69) | NA | NA | NA | NA | NA | 0.85 (0.78–0.91) |
| Simón et al (2020), Spain | serum PlGF ≥ 19.1 ng/mL | perinatal death bronchopulmonary dysplasia IVH NEC ROP | 76 | 13 (17.11) | NA | NA | NA | NA | 0.50 (0.19-0.81) | 0.9 | 0.38 (0.14-0.70) | 0.92 (0.86-0.96) | 4.1 (1.6-10.7) | NA | 0.78 (0.66–0.90) |
| Karge et al (2022), Germany | serum sFlt-1 | SGA neonatal mortality neonatal seizures NICU admission  respiratory support | 141 | 69 (48.94) | NA | NA | NA | NA | NA | NA | NA | NA | NA | NA | 0.721 (0.637–0.804) |
| Kumar et al (2023), India | serum sFlt-1 ≥ 8169.5 pg/mL | preterm birth at ≤34 week  NICU admission RDS HIE NEC ROP neonatal seizures perinatal death | 91 | 39 (42.90) | NA | NA | NA | NA | 0.71 | 0.85 | NA | NA | NA | NA | 0.83 (0.74-0.91) |
| Reddy et al (2022), Australia | serum sFlt-1 | preterm birth (< 34 weeks) NICU admission  RDS IVH HIE NEC confirmed fetal infection ROB | 126 | 48 (38.10) | NA | NA | NA | NA | 0.42 (0.17-0.60) | NA | NA | NA | NA | NA | 0.81 (0.73–0.88) |
| Binder et al (2023), Austria | sFlt-1/PlGF ratio abnormal | stillbirth neonatal death IVH ROP NEC | 323 | 47 (14.60) | 211 | 45 | 65 | 2 | NA | NA | NA | NA | NA | NA | NA |
| Chaiworapongsa et al (2023), USA | abnormal angiogenic profile (sFlt-1/PlGF ratio as a multiple of the median (MoM) < 10th percentile for gestational age in early onset pre-eclampsia (cohort 1) | requirement of oxygen supplementation CPAP or mechanical ventilation RDS confirmed sepsis NEC IVH periventricular leukomalacia meconium aspiration syndrome chronic lung disease neonatal death | 27 | 23 (85.19) | 3 | 21 | 1 | 2 | NA | NA | NA | NA | NA | NA | NA |
| Chaiworapongsa et al (2023), USA | abnormal angiogenic profile (sFlt-1/PlGF ratio as a multiple of the median (MoM) < 10th percentile for gestational age in intermediate pre-eclampsia (cohort 1) | requirement of oxygen supplementation CPAP or mechanical ventilation RDS confirmed sepsis NEC IVH periventricular leukomalacia meconium aspiration syndrome chronic lung disease neonatal death | 15 | 1 (6.25) | 14 | 1 | 0 | 0 | NA | NA | NA | NA | NA | NA | NA |
| Chaiworapongsa et al (2023), USA | abnormal angiogenic profile (sFlt-1/PlGF ratio as a multiple of the median (MoM) < 10th percentile for gestational age in term pre-eclampsia (cohort 1) | requirement of oxygen supplementation CPAP or mechanical ventilation RDS confirmed sepsis NEC IVH periventricular leukomalacia meconium aspiration syndrome chronic lung disease neonatal death | 106 | 2 (1.89) | 39 | 2 | 65 | 0 | NA | NA | NA | NA | NA | NA | NA |
| Chaiworapongsa et al (2023), USA | abnormal angiogenic profile (sFlt-1/PlGF ratio as a multiple of the median (MoM) < 10th percentile for gestational age in early onset pre-eclampsia (cohort 2) | requirement of oxygen supplementation CPAP or mechanical ventilation RDS confirmed sepsis NEC IVH periventricular leukomalacia meconium aspiration syndrome chronic lung disease neonatal death | 84 | 68 (80.95) | 16 | 66 | 0 | 2 | NA | NA | NA | NA | NA | NA | NA |
| Chaiworapongsa et al (2023), USA | abnormal angiogenic profile (sFlt-1/PlGF ratio as a multiple of the median (MoM) < 10th percentile for gestational age in intermediate pre-eclampsia (cohort 2) | requirement of oxygen supplementation CPAP or mechanical ventilation RDS confirmed sepsis NEC IVH periventricular leukomalacia meconium aspiration syndrome chronic lung disease neonatal death | 102 | 16 (15.69) | 69 | 15 | 20 | 1 | NA | NA | NA | NA | NA | NA | NA |
| Chaiworapongsa et al (2023), USA | abnormal angiogenic profile (sFlt-1/PlGF ratio as a multiple of the median (MoM) < 10th percentile for gestational age in term pre-eclampsia (cohort 2) | requirement of oxygen supplementation CPAP or mechanical ventilation RDS confirmed sepsis NEC IVH periventricular leukomalacia meconium aspiration syndrome chronic lung disease neonatal death | 253 | 10 (3.95) | 129 | 9 | 114 | 1 | NA | NA | NA | NA | NA | NA | NA |
| Graupner et al (2019), Germany | sFlt-1/PlGF ratio ≥ 110 | pH value umbilical cord artery ≤ 7.0 and/or base excess ≤ –12 mmol/L 5 min Apgar score ≤ 7 NICU admission RDS  respiratory support > 4 h SGA | 67 | 12 (17.90) | 22 | 5 | 33 | 7 | NA | NA | NA | NA | NA | NA | 0.514 (0.31–0.72) |
| Karge et al (2021), Germany | sFlt-1/PlGF ratio | NICU admission  intubation of the newborn RDS | 49 | 10 (20.41) | NA | NA | NA | NA | NA | NA | NA | NA | NA | NA | NA |
| Karge et al (2022), Germany | sFlt-1/PlGF ratio | SGA neonatal mortality neonatal seizures NICU admission  respiratory support | 141 | 69 (48.94) | NA | NA | NA | NA | NA | NA | NA | NA | NA | NA | 0.880 (0.824–0.936) |
| Kumar et al (2023), India | sFlt-1/PlGF ratio ≥ 71.92 | preterm birth at ≤34 week  NICU admission RDS HIE NEC ROP neonatal seizures perinatal death | 91 | 39 (42.90) | NA | NA | NA | NA | 0.90 | 0.89 | NA | NA | NA | NA | 0.88 (0.80-0.96) |
| Reddy et al (2022), Australia | sFlt-1/PlGF ratio | preterm birth (< 34 weeks) NICU admission  RDS IVH HIE NEC confirmed fetal infection ROB | 126 | 48 (38.10) | NA | NA | NA | NA | 0.54 (0.25-0.75) | NA | NA | NA | NA | NA | 0.87 (0.81–0.93) |
| Simón et al (2020), Spain | sFlt-1/PlGF ratio ≥ 841 | perinatal death bronchopulmonary dysplasia IVH NEC ROP | 76 | 13 (17.11) | NA | NA | NA | NA | 0.30 (0.15-0.54) | 0.9 | 0.62 (0.37-0.82) | 0.73 (0.67-0.78) | 3.3 (1.2-8.9) | NA | 0.75 (0.62–0.88) |
| Suresh et al (2017), US | sFlt-1/PlGF ratio ≥ 38 | low birth weight fetal or neonatal death | 269 | NA (NA) | NA | NA | NA | NA | 0.95 | 0.38 | 0.11 | 0.99 | 1.53 | 0.13 | 0.66 (0.61– 0.72) |
| Suresh et al (2017), US | sFlt-1/PlGF ratio ≥ 85 | low birth weight fetal or neonatal death | 269 | NA (NA) | NA | NA | NA | NA | 0.86 | 0.62 | 0.16 | 0.98 | 2.26 | 0.23 | 0.74 (0.66– 0.82) |
| Tousty et al (2022), Poland | sFlt-1/PlGF ratio ≥ 204 | Congenital or late–onset infections RDS PDA NEC IVH ROP BPD infant death | 77 | 22 (28.57) | 14 | 13 | 41 | 9 | 0.64 | 0.75 | NA | NA | NA | NA | 0.64 |
| **Doppler assessment** | | | | | | | | | | | | | | | |
| El–Demiry et al (2020), Egypt | CPR < 1.02 | 5 min Apgar score < 7 SGA Occurrence of respiratory complications NICU admission  perinatal death | 60 | 21 (35.00) | 2 | 14 | 37 | 7 | 0.67 | 0.95 | 0.88 | 0.84 | 13 | 0.35 | 0.874 (0.771–0.976) |
| Graupner et al (2019), Germany | CPR < 5th centile | pH value umbilical cord artery ≤ 7.0 and/or base excess ≤ –12 mmol/L 5 min Apgar score ≤ 7 NICU admission RDS  respiratory support > 4 h SGA | 67 | 12 (17.90) | 7 | 0 | 48 | 12 | NA | NA | NA | NA | NA | NA | 0.548 (0.35–0.75) |
| Gupta et al (2017), India | CPR < 1.08 | low birth weight perinatal death  emergency lower–segment cesarean section for fetal distress 5 min Apgar score < 7 NICU admission | 100 | 83 (83.00) | NA | NA | NA | NA | 0.89 | 0.8 | 0.77 | 0.81 | NA | NA | NA |
| Malik et al (2023), India | CPR < 1.08 | cesarean section for fetal distress instrumental delivery for fetal distress SGA meconium stained liquor  1 and 5 min Apgar score < 7 NICU admission stillbirth/perinatal death | 100 | 58 (58.00) | 0 | 32 | 42 | 26 | 0.55 (0.42-0.68) | 1 (0.92-1) | 1 (0.89-1) | 0.62 (0.49-0.73) | NA | NA | NA |
| Moawad et al (2022), Egypt | CPR < 1.0 | 1 and 5 min Apgar score <7 metabolic acidosis at birth (pH <7.20) low birth weight (< 2500g) IGUR  NICU admission | 60 | NA (NA) | NA | NA | NA | NA | 0.71 | 0.6 | 0.54 | 0.72 | NA | NA | NA |
| Nayak et al (2022), India | CPR < 1.0 | hypoglycemia polycythemia neonatal asphyxia acidosis neonatal sepsis | 62 | 13 (20.97) | NA | NA | NA | NA | 0.85 (0.58-0.96) | 0.91 (0.78-0.96) | 0.42 (0.26-0.61) | 0.94 (0.82-0.98) | NA | NA | NA |
| Peguero et al (2023), Spain | CPR < 5th centile | stillbirth neonatal death  neonatal metabolic acidosis  5 min Apgar score < 7 BDP  NEC grade III or IV IVH cystic periventricular leukomalacia stage 3–5 ROP HIE acute renal failure (serum creatinine >1.5 mg/dL) cardiac failure (requiring inotropic support) | 63 | 18 (28.57) | 14 | 16 | 31 | 2 | NA | NA | NA | NA | NA | NA | NA |
| Reddy et al (2022), Australia | CPR percentile | preterm birth (< 34 weeks) NICU admission  RDS IVH HIE NEC confirmed fetal infection ROB | 126 | 48 (38.10) | NA | NA | NA | NA | 0.33 (0.16-0.56) | NA | NA | NA | NA | NA | 0.71 (0.61–0.80) |
| Saxena et al (2021), India | CPR < 1.0 | cesarean section for fetal distress  Meconium stained Liquor 5 min Apgar score < 7 preinatal death NICU admission | 150 | NA (NA) | NA | NA | NA | NA | 1 (0.88-1) | 0.34 (0.26-0.43) | 0.28 (0.19-0.37) | 1 (0.91-1) | NA | NA | NA |
| Zarean et al (2022), Iran | CPR ≤ 1.0 | birth weight perinatal death 5 min Apgar score respiratory problems acidemia seizure | 100 | 38 (38.00) | 13 | 14 | 49 | 24 | 0.52 | 0.71 | 0.4 | 0.8 | NA | NA | NA |
| El–Demiry et al (2020), Egypt | MCA PI < 1.31 | 5 min Apgar score < 7 SGA Occurrence of respiratory complications NICU admission  perinatal death | 60 | 21 (35.00) | 5 | 12 | 34 | 9 | 0.57 | 0.87 | 0.71 | 0.79 | 4.46 | 0.49 | NA |
| Gupta et al (2017), India | MCA PI < 5th percentile | low birth weight perinatal death  emergency lower–segment cesarean section for fetal distress 5 min Apgar score < 7 NICU admission | 100 | 83 (83.00) | 17 | 39 | 0 | 44 | 0.76 | 0.8 | 0.8 | 0.8 | NA | NA | NA |
| Moawad et al (2022), Egypt | MCA PI < 5th percentile | 1 and 5 min Apgar score <7 metabolic acidosis at birth (pH <7.20) low birth weight (< 2500g) IGUR  NICU admission | 60 | NA (NA) | NA | NA | NA | NA | 0.47 | 0.71 | 0.47 | 0.77 | NA | NA | NA |
| Peguero et al (2023), Spain | MCA PI < 5th centile | stillbirth neonatal death  neonatal metabolic acidosis  5 min Apgar score < 7 BDP  NEC grade III or IV IVH cystic periventricular leukomalacia stage 3–5 ROP HIE acute renal failure (serum creatinine >1.5 mg/dL) cardiac failure (requiring inotropic support) | 63 | 18 (28.57) | 12 | 10 | 33 | 8 | NA | NA | NA | NA | NA | NA | NA |
| Reddy et al (2022), Australia | MCA PI percentile | preterm birth (< 34 weeks) NICU admission  RDS IVH HIE NEC confirmed fetal infection ROB | 126 | 48 (38.10) | NA | NA | NA | NA | 0.35 (0.15-0.52) | NA | NA | NA | NA | NA | 0.71 (0.61–0.80) |
| El–Demiry et al (2020), Egypt | MCA RI < 0.67 | 5 min Apgar score < 7 SGA Occurrence of respiratory complications NICU admission  perinatal death | 60 | 21 (35.00) | 4 | 13 | 35 | 8 | 0.62 | 0.9 | 0.76 | 0.81 | 6.04 | 0.42 |  |
| Moawad et al (2022), Egypt | MCA RI < 5th percentile | 1 and 5 min Apgar score <7 metabolic acidosis at birth (pH <7.20) low birth weight (< 2500g) IGUR  NICU admission | 60 | NA (NA) | NA | NA | NA | NA | 0.31 | 0.52 | 0.5 | 0.34 | NA | NA | NA |
| Malik et al (2023), India | MCA S/D ratio > 3 | cesarean section for fetal distress instrumental delivery for fetal distress SGA meconium stained liquor  1 and 5 min Apgar score < 7 NICU admission stillbirth/perinatal death | 100 | 58 (58.00) | 7 | 9 | 35 | 49 | 0.16 (0.07-0.27) | 0.83 (0.69-0.93) | 0.56 (0.30-0.80) | 0.42 (0.31-0.53) | NA | NA | NA |
| Graupner et al (2019), Germany | UtA PI > 95th percentile | pH value umbilical cord artery ≤ 7.0 and/or base excess ≤ –12 mmol/L 5 min Apgar score ≤ 7 NICU admission RDS  respiratory support > 4 h SGA | 67 | 12 (17.90) | 20 | 3 | 35 | 9 | NA | NA | NA | NA | NA | NA | 0.552 (0.36–0.74) |
| Loardi et al (2021), Italy | UtA PI > 95th percentile | stillbirth neonatal death RDS IVH sepsis NEC FGR | 311 | 160 (51.40) | NA | NA | NA | NA | 0.82 (0.75-0.88) | 0.63 (0.55-0.71) | 0.70 (0.63-0.77) | 0.77 (0.68-0.84) | 2.21 (1.77-2.75) | 0.288 (0.20-0.41) |  |
| Peguero et al (2023), Spain | UtA PI > 95th percentile | stillbirth neonatal death  neonatal metabolic acidosis  5 min Apgar score < 7 BDP  NEC grade III or IV IVH cystic periventricular leukomalacia stage 3–5 ROP HIE acute renal failure (serum creatinine >1.5 mg/dL) cardiac failure (requiring inotropic support) | 63 | 18 (28.57) | 37 | 15 | 8 | 3 | NA | NA | NA | NA | NA | NA | NA |
| Reddy et al (2022), Australia | UtA PI | preterm birth (< 34 weeks) NICU admission  RDS IVH HIE NEC confirmed fetal infection ROB | 126 | 48 (38.10) | NA | NA | NA | NA | 0.40 (0.25-0.58) | NA | NA | NA | NA | NA | 0.76 (0.67–0.85) |
| Thangaratinam et al (2017), UK | UtA PI abnormal | infant death BPD NEC  IVH Cystic periventricular leukomalacia ROP  HIE | 945 | 702 (74.29) | 12 | 79 | 231 | 17 | NA | NA | NA | NA | NA | NA | NA |
| El–Demiry et al (2020), Egypt | UA PI > 1.24 | 5 min Apgar score < 7 SGA Occurrence of respiratory complications NICU admission  perinatal death | 60 | 21 (35.00) | 2 | 11 | 37 | 10 | 0.52 | 0.95 | 0.85 | 0.79 | 10.21 | 0.5 | 0.859 (0.767–0.951) |
| Gupta et al (2017), India | UA PI > 95th percentile | low birth weight perinatal death  emergency lower–segment cesarean section for fetal distress 5 min Apgar score < 7 NICU admission | 100 | 83 (83.00) | 3 | 36 | 14 | 47 | 0.6 | 0.89 | 0.82 | 0.8 | NA | NA | NA |
| Li et al (2019), China | UA PI | SGA intrauterine distress premature birth (< 37 weeks) neonatal asphyxia (hypoxemia or hypercapnia due to inability of normal breathing autonomously in the intrauterine fetus within 1 minute of birth for lack of oxygen) | 124 | 35 (28.23) | NA | NA | NA | NA | NA | NA | NA | NA | NA | NA | 0.840 (0.674–1.00) |
| Moawad et al (2022), Egypt | UA PI > 95th percentile | 1 and 5 min Apgar score <7 metabolic acidosis at birth (pH <7.20) low birth weight (< 2500g) IGUR  NICU admission | 60 | NA (NA) | NA | NA | NA | NA | 0.56 | 0.74 | 0.58 | 0.69 | NA | NA | NA |
| Peguero et al (2023), Spain | UA PI > 95th percentile | stillbirth neonatal death  neonatal metabolic acidosis  5 min Apgar score < 7 BDP  NEC grade III or IV IVH cystic periventricular leukomalacia stage 3–5 ROP HIE acute renal failure (serum creatinine >1.5 mg/dL) cardiac failure (requiring inotropic support) | 63 | 18 (28.57) | 7 | 7 | 38 | 11 | NA | NA | NA | NA | NA | NA | NA |
| Reddy et al (2022), Australia | UA PI percentile | preterm birth (< 34 weeks) NICU admission  RDS IVH HIE NEC confirmed fetal infection ROB | 126 | 48 (38.10) | NA | NA | NA | NA | 0.37 (0.21-0.52) | NA | NA | NA | NA | NA | 0.65 (0.55–0.76) |
| Li et al (2019), China | UA RI | SGA intrauterine distress premature birth (< 37 weeks) neonatal asphyxia (hypoxemia or hypercapnia due to inability of normal breathing autonomously in the intrauterine fetus within 1 minute of birth for lack of oxygen) | 124 | 35 (28.23) | NA | NA | NA | NA | NA | NA | NA | NA | NA | NA | 0.770 (0.615–0.925) |
| Moawad et al (2022), Egypt | UA RI > 95th percentile | 1 and 5 min Apgar score <7 metabolic acidosis at birth (pH <7.20) low birth weight (< 2500g) IGUR  NICU admission | 60 | NA (NA) | NA | NA | NA | NA | 0.52 | 0.6 | 0.56 | 0.54 | NA | NA | NA |
| Li et al (2019), China | UA S/D ratio | SGA intrauterine distress premature birth (< 37 weeks) neonatal asphyxia (hypoxemia or hypercapnia due to inability of normal breathing autonomously in the intrauterine fetus within 1 minute of birth for lack of oxygen) | 124 | 35 (28.23) | NA | NA | NA | NA | NA | NA | NA | NA | NA | NA | 0.760 (0.570–0.949) |
| Malik et al (2023), India | UA S/D ratio < 3 | cesarean section for fetal distress instrumental delivery for fetal distress SGA meconium stained liquor  1 and 5 min Apgar score < 7 NICU admission stillbirth/perinatal death | 100 | 58 (58.00) | 0 | 15 | 42 | 43 | 0.26 (0.15-0.39) | 1 (0.92-1) | 1 (0.78-1) | 0.49 (0.38-0.60) | NA | NA | NA |
| Wu et al (2017), China | UA S/D ratio | perinatal death admission to the NICU  5 min Apgar score < 8 | 120 | 36 (30.00) | NA | NA | NA | NA | 0.74 | 0.41 | NA | NA | NA | NA | 0.58 |
| Peguero et al (2023), Spain | UA AEDV or REDV | stillbirth neonatal death  neonatal metabolic acidosis  5 min Apgar score < 7 BDP  NEC grade III or IV IVH cystic periventricular leukomalacia stage 3–5 ROP HIE acute renal failure (serum creatinine >1.5 mg/dL) cardiac failure (requiring inotropic support) | 63 | 18 (28.57) | 2 | 3 | 43 | 15 | NA | NA | NA | NA | NA | NA | NA |
| Peguero et al (2023), Spain | DV PI > 95th percentile | stillbirth neonatal death  neonatal metabolic acidosis  5 min Apgar score < 7 BDP  NEC grade III or IV IVH cystic periventricular leukomalacia stage 3–5 ROP HIE acute renal failure (serum creatinine >1.5 mg/dL) cardiac failure (requiring inotropic support) | 63 | 18 (28.57) | 3 | 4 | 42 | 14 | NA | NA | NA | NA | NA | NA | NA |
| El–Demiry et al (2020), Egypt | DV PSV > 45.1 cm/s | 5 min Apgar score < 7 SGA Occurrence of respiratory complications NICU admission  perinatal death | 60 | 21 (35.00) | 14 | 15 | 25 | 6 | 0.71 | 0.64 | 0.52 | 0.81 | 1.99 | 0.45 | 0.702 (0.555–0.850) |
| El–Demiry et al (2020), Egypt | DV PVIV > 0.58 | 5 min Apgar score < 7 SGA Occurrence of respiratory complications NICU admission  perinatal death | 60 | 21 (35.00) | 10 | 13 | 29 | 8 | 0.62 | 0.74 | 0.57 | 0.78 | 2.41 | 0.51 | 0.726 (0.590–0.863) |
| Reddy et al (2022), Australia | OA PSV ratio | preterm birth (< 34 weeks) NICU admission  RDS IVH HIE NEC confirmed fetal infection ROB | 126 | 48 (38.10) | NA | NA | NA | NA | 0.27 (0.13-0.43) | NA | NA | NA | NA | NA | 0.60 (0.49–0.70) |
| Gupta et al (2017), India | UtA diastolic notch present | low birth weight perinatal death  emergency lower–segment cesarean section for fetal distress 5 min Apgar score < 7 NICU admission | 100 | 83 (83.00) | 13 | 28 | 4 | 55 | NA | NA | NA | NA | NA | NA | NA |
| Chaves et al (2017), Brazil | abnormal PR (ratio of the flow velocity of the second peak to that of the initial systolic velocity peak) 0.78-0.98 | low birth weight neonatal acidemia (pH <7.0 and base deficit ≥2 mmol/L) 5 min Apgar score < 7 NICU admission preterm birth (< 32 weeks) fetal or neonatal death | 56 | 43 (76.79) | 9 | 24 | 4 | 19 | NA | NA | NA | NA | NA | NA | NA |
| Chaves et al (2017), Brazil | abnormal PR (ratio of the flow velocity of the second peak to that of the initial systolic velocity peak) ≥ 0.99 | low birth weight neonatal acidemia (pH <7.0 and base deficit ≥2 mmol/L) 5 min Apgar score < 7 NICU admission preterm birth (< 32 weeks) fetal or neonatal death | 56 | 43 (76.79) | 2 | 14 | 11 | 29 | NA | NA | NA | NA | NA | NA | NA |
| Chaves et al (2017), Brazil | abnormal PR (ratio of the flow velocity of the second peak to that of the initial systolic velocity peak) | low birth weight neonatal acidemia (pH <7.0 and base deficit ≥2 mmol/L) 5 min Apgar score < 7 NICU admission preterm birth (< 32 weeks) fetal or neonatal death | 56 | 43 (76.79) | NA | NA | NA | NA | NA | NA | NA | NA | NA | NA | 0.564 (0.406–0.723) |
| **signs and/or symptoms** | | | | | | | | | | | | | | | |
| Thangaratinam et al (2017), UK | headache and/or visual disturbance | infant death BPD NEC  IVH Cystic periventricular leukomalacia ROP  HIE | 945 | 702 (74.29) | 102 | 278 | 141 | 398 | NA | NA | NA | NA | NA | NA | NA |
| Thangaratinam et al (2017), UK | chest pain and/or breathlessness | infant death BPD NEC  IVH Cystic periventricular leukomalacia ROP  HIE | 945 | 702 (74.29) | 12 | 48 | 231 | 530 | NA | NA | NA | NA | NA | NA | NA |
| Thangaratinam et al (2017), UK | epigastric pain, nausea and/or vomiting | infant death BPD NEC  IVH Cystic periventricular leukomalacia ROP  HIE | 945 | 702 (74.29) | 43 | 157 | 200 | 500 | NA | NA | NA | NA | NA | NA | NA |
| Magee et al (2016), multinational | prior SBP ≥160 or DBP ≥110 mmHg in this pregnancy | pregnancy loss NICU admission | 987 | 307 (31.10) | 83 | 58 | 593 | 247 | NA | NA | NA | NA | NA | NA | NA |
| Reddy et al (2022), Australia | abdominal circumference percentile | preterm birth (< 34 weeks) NICU admission  RDS IVH HIE NEC confirmed fetal infection ROB | 126 | 48 (38.10) | NA | NA | NA | NA | 0.52 (0.25-0.70) | NA | NA | NA | NA | NA | 0.77 (0.68–0.86) |
| Thangaratinam et al (2017), UK | exaggerate tendon reflexes | infant death BPD NEC  IVH Cystic periventricular leukomalacia ROP  HIE | 945 | 702 (74.29) | 20 | 126 | 223 | 225 | NA | NA | NA | NA | NA | NA | NA |
| Thangaratinam et al (2017), UK | Clonus | infant death BPD NEC  IVH Cystic periventricular leukomalacia ROP  HIE | 945 | 702 (74.29) | 10 | 84 | 233 | 218 | NA | NA | NA | NA | NA | NA | NA |
| Magee et al (2016), multinational | gestational hypertension | pregnancy loss NICU admission | 987 | 307 (31.10) | 162 | 87 | 514 | 218 | NA | NA | NA | NA | NA | NA | NA |
| Thangaratinam et al (2017), UK | oxygen saturation: abnormal (< 94%) | infant death BPD NEC  IVH Cystic periventricular leukomalacia ROP  HIE | 945 | 702 (74.29) | 1 | 3 | 242 | 182 | NA | NA | NA | NA | NA | NA | NA |
| Loardi et al (2021), Italy | pre-eclampsia diagnosis | stillbirth neonatal death RDS IVH sepsis NEC FGR | 311 | 160 (51.40) | NA | NA | NA | NA | 0.84 (0.78-1) | 0.48 (0.40-0.56) | 0.63 (0.56-0.70) | 0.74 (0.64-0.83) | 1.61 (1.37-1.9) | 0.33 (0.22-0.49) | NA |
| **biomedical/laboratory tests** | | | | | | | | | | | | | | | |
| Binder et al (2023), Austria | thrombocytopenia | stillbirth neonatal death IVH ROP NEC | 323 | 47 (14.60) | 22 | 10 | 254 | 37 | NA | NA | NA | NA | NA | NA | NA |
| Mayama et al (2021), Japan | thrombocytopenia ≥ 100 x 10^9/L and < 150 x 10^9/L platelet count | FGR abnormal artery doppler waveform stillbirth | 264 | 78 (29.55) | 34 | 17 | 152 | 61 | NA | NA | NA | NA | NA | NA | NA |
| Mayama et al (2021), Japan | thrombocytopenia ≥ 150 x 10^9/L platelet count | FGR abnormal artery doppler waveform stillbirth | 264 | 78 (29.55) | 17 | 7 | 169 | 71 | NA | NA | NA | NA | NA | NA | NA |
| Simón et al (2020), Spain | platelet count 304.5 platelets/µL | perinatal death bronchopulmonary dysplasia IVH NEC ROP | 76 | 13 (17.11) | NA | NA | NA | NA | 0.10 (0.03-0.31) | 0.9 | 0.38 (0.14-0.70) | 0.68 (0.64-0.71) | 1.2 (0.3-4.7) | NA | 0.45 (0.25–0.64) |
| Kumar et al (2023), India | serum uric acid ≥7.55 mg/dL | preterm birth at ≤34 week  NICU admission RDS HIE NEC ROP neonatal seizures perinatal death | 91 | 39 (42.90) | NA | NA | NA | NA | 0.68 | 0.69 | NA | NA | NA | NA | NA |
| Le et al (2018), Vietnam | serum uric acid levels ≥ 393 µmol/L | IUGR  preterm birth 1 min Apgar score < 7  stillbirth neonatal death | 205 | NA (NA) | NA | NA | NA | NA | 0.64 | 0.8 | NA | NA | NA | NA | 0.75 |
| Binder et al (2023), Austria | serum transaminase > 66 IU/L | stillbirth neonatal death IVH ROP NEC | 323 | 47 (14.60) | 45 | 13 | 231 | 34 | NA | NA | NA | NA | NA | NA | NA |
| Simón et al (2020), Spain | AST (U/L) | perinatal death bronchopulmonary dysplasia IVH NEC ROP | 76 | 13 (17.11) | NA | NA | NA | NA | 0.40 (0.21-0.61) | 0.9 | 0.67 (0.43-0.84) | 0.75 (0.69-0.81) | 4.1 (1.6-10.7) | NA | 0.68 (0.49–0.88) |
| Binder et al (2023), Austria | serum creatinine > 1 mg/dL | stillbirth neonatal death IVH ROP NEC | 323 | 47 (14.60) | 19 | 2 | 257 | 45 | NA | NA | NA | NA | NA | NA | NA |
| Kumar et al (2023), India | serum creatinine ≥ 0.79 mg/dL | preterm birth at ≤34 week  NICU admission RDS HIE NEC ROP neonatal seizures perinatal death | 91 | 39 (42.90) | NA | NA | NA | NA | 0.74 | 0.62 | NA | NA | NA | NA | Kumar et al (2023), India |
| Simón et al (2020), Spain | serum creatinine ≥ 0.8 mg/dL | perinatal death bronchopulmonary dysplasia IVH NEC ROP | 76 | 13 (17.11) | NA | NA | NA | NA | 0.10 (0.03-0.31) | 0.9 | 0.38 (0.14-0.70) | 0.68 (0.64-0.71) | 1.2 (0.3-4.7) | NA | 0.46 (0.26–0.65) |
| Binder et al (2023), Austria | serum LDH > 280 IU/L | stillbirth neonatal death IVH ROP NEC | 323 | 47 (14.60) | 57 | 19 | 219 | 28 | NA | NA | NA | NA | NA | NA | NA |
| Kumar et al (2023), India | serum LDH ≥ 615 | preterm birth at ≤34 week  NICU admission RDS HIE NEC ROP neonatal seizures perinatal death | 91 | 39 (42.90) | NA | NA | NA | NA | 0.97 | 0.23 | NA | NA | NA | NA | NA |
| Joshi et al (2022), India | serum hsCRP > 3.0 mg/L | non–stress test abnormalities meconium–stained liquor  low birth weight  preterm birth (< 37 weeks) NICU admission intauterine fetal demise neonatal death | 132 | 57 (43.18) | 7 | 53 | 68 | 4 | NA | NA | NA | NA | NA | NA | NA |
| Hong et al (2021), China | B–type natriuretic peptide > 118 pg/mL | SGA fetal distress fetal death stillbirth oligohydramnios, and  neonatal asphyxia (1 min Apgar scores <7 and umbilical blood pH <7.20) | 284 | 76 (26.80) | NA | NA | NA | NA | NA | NA | NA | NA | NA | NA | 0.642 (0.583–0.698) |
| He et al (2022), China | relative expression of miRNA–204 in serum > median of study | low birth weight  birth asphyxia (wheezing, very irregular respiration, without respiration) SGA preterm birth  NICU admission perinatal death 1 min Apgar score > 7 | 196 | 108 (55.10) | 23 | 75 | 65 | 33 | NA | NA | NA | NA | NA | NA | NA |
| Elia et al (2017), UK | log transformed ACR | preterm delivery (< 34 weeks) low birth weight  abnormal umbilical artery Doppler  arterial cord pH < 7.1 need for ventilation neonatal or intrauterine death | 717 | 146 (20.36) | NA | NA | NA | NA | NA | NA | NA | NA | NA | NA | 0.557 (0.504–0.610) |
| Wu et al (2017), China | serum gamma glutamyl transferase | perinatal death admission to the NICU  5 min Apgar score < 8 | 120 | 36 (30.00) | NA | NA | NA | NA | 0.77 | 0.52 | NA | NA | NA | NA | 0.65 |
| **fetal variables** | | | | | | | | | | | | | | | |
| Peguero et al (2023), Spain | FGR (calculated by Hadlock formula) | stillbirth neonatal death  neonatal metabolic acidosis  5 min Apgar score < 7 BDP  NEC grade III or IV IVH cystic periventricular leukomalacia stage 3–5 ROP HIE acute renal failure (serum creatinine >1.5 mg/dL) cardiac failure (requiring inotropic support) | 63 | 18 (28.57) | 34 | 17 | 11 | 1 | NA | NA | NA | NA | NA | NA | NA |
| Peguero et al (2023), Spain | severe FGR (persistent AEDV or REDV in the UA or DV PI > 95th centile) | stillbirth neonatal death  neonatal metabolic acidosis  5 min Apgar score < 7 BDP  NEC grade III or IV IVH cystic periventricular leukomalacia stage 3–5 ROP HIE acute renal failure (serum creatinine >1.5 mg/dL) cardiac failure (requiring inotropic support) | 63 | 18 (28.57) | 5 | 7 | 40 | 11 | NA | NA | NA | NA | NA | NA | NA |
| Reddy et al (2022), Australia | EFW percentile | preterm birth (< 34 weeks) NICU admission  RDS IVH HIE NEC confirmed fetal infection ROB | 126 | 48 (38.10) | NA | NA | NA | NA | 0.56 (0.38-0.74) | NA | NA | NA | NA | NA | 0.81 (0.73–0.89) |
| Thangaratinam et al (2017), UK | EFW < 10 th centile | infant death BPD NEC  IVH Cystic periventricular leukomalacia ROP  HIE | 945 | 702 (74.29) | 27 | 261 | 216 | 208 | NA | NA | NA | NA | NA | NA | NA |
| Thangaratinam et al (2017), UK | CTG findings abnormal | infant death BPD NEC  IVH Cystic periventricular leukomalacia ROP  HIE | 945 | 702 (74.29) | 10 | 36 | 233 | 431 | NA | NA | NA | NA | NA | NA | NA |
| **Diagnosis** | | | | | | | | | | | | | | | |
| Magee et al (2021), multinational | pre-eclampsia definition A (development of proteinuria) in chronic hypertension | stillbirth  neonatal death NICU admission | 731 | 217 (29.69) | 49 | 53 | 465 | 164 | 0.24 | 0.9 | NA | NA | NA | NA | NA |
| Magee et al (2021), multinational | pre-eclampsia definition B (development of proteinuria AND one or more symptoms, signs, abnormal laboratory test) in chronic hypertension | stillbirth  neonatal death NICU admission | 731 | 217 (29.69) | 130 | 106 | 384 | 111 | 0.49 | 0.75 | NA | NA | NA | NA | NA |
| Magee et al (2021), multinational | pre-eclampsia definition C (one or more symptoms, sings) in chronic hypertension | stillbirth  neonatal death NICU admission | 731 | 217 (29.69) | 132 | 110 | 382 | 107 | 0.51 | 0.74 | NA | NA | NA | NA | NA |
| Magee et al (2021), multinational | pre-eclampsia definition D (one or more symptoms, signs, abnormal laboratory test) in chronic hypertension | stillbirth  neonatal death NICU admission | 731 | 217 (29.69) | 186 | 148 | 328 | 69 | 0.68 | 0.64 | NA | NA | NA | NA | NA |
| Magee et al (2021), multinational | pre-eclampsia definition E (development of proteinuria OR one or more symptoms, signs, abnormal laboratory test) in chronic hypertension | stillbirth  neonatal death NICU admission | 731 | 217 (29.69) | 185 | 144 | 329 | 73 | 0.66 | 0.64 | NA | NA | NA | NA | NA |
| Magee et al (2021), multinational | pre-eclampsia definition F (development of proteinuria development of proteinuria OR one or more symptoms, signs) in chronic hypertension | stillbirth  neonatal death NICU admission | 248 | 87 (35.08) | 47 | 39 | 114 | 48 | 0.45 | 0.71 | NA | NA | NA | NA | NA |
| Magee et al (2021), multinational | pre-eclampsia definition A (development of proteinuria) in gestational  hypertension | stillbirth  neonatal death NICU admission | 248 | 87 (35.08) | 24 | 29 | 137 | 58 | 0.33 | 0.85 | NA | NA | NA | NA | NA |
| Magee et al (2021), multinational | pre-eclampsia definition B (development of proteinuria AND one or more symptoms, signs, abnormal laboratory test) in gestational hypertension | stillbirth  neonatal death NICU admission | 248 | 87 (35.08) | 52 | 51 | 109 | 36 | 0.59 | 0.68 | NA | NA | NA | NA | NA |
| Magee et al (2021), multinational | pre-eclampsia definition C (one or more symptoms, sings) in gestational hypertension | stillbirth  neonatal death NICU admission | 248 | 87 (35.08) | 52 | 52 | 109 | 35 | 0.6 | 0.68 | NA | NA | NA | NA | NA |
| Magee et al (2021), multinational | pre-eclampsia definition D (one or more symptoms, signs, abnormal laboratory test) in gestational hypertension | stillbirth  neonatal death NICU admission | 248 | 87 (35.08) | 75 | 62 | 86 | 25 | 0.71 | 0.53 | NA | NA | NA | NA | NA |
| Magee et al (2021), multinational | pre-eclampsia definition E (development of proteinuria OR one or more symptoms, signs, abnormal laboratory test) in gestational hypertension | stillbirth  neonatal death NICU admission | 248 | 87 (35.08) | 75 | 62 | 86 | 25 | 0.71 | 0.53 | NA | NA | NA | NA | NA |
| Magee et al (2021), multinational | pre-eclampsia definition F (development of proteinuria development of proteinuria OR one or more symptoms, signs) in gestational hypertension | stillbirth  neonatal death NICU admission | 731 | 217 (29.69) | 103 | 91 | 411 | 126 | 0.42 | 0.8 | NA | NA | NA | NA | NA |
| Nayak et al (2022), India | Nonstress Test | hypoglycemia polycythemia neonatal asphyxia acidosis neonatal sepsis | 62 | 13 (20.97) | NA | NA | NA | NA | 0.62 (0.36-0.82) | 0.69 (0.55-0.80) | 0.35 (0.19-0.55) | 0.87 (0.73-0.94) | NA | NA | NA |
| Magee et al (2016), multinational | PMR recruiting country ≥10 perinatal death/100 births | pregnancy loss NICU admission | 987 | 307 (31.10) | 116 | 44 | 560 | 261 | NA | NA | NA | NA | NA | NA | NA |
| Thangaratinam et al (2017), UK | Liquor volume abnormal | infant death BPD NEC  IVH Cystic periventricular leukomalacia ROP  HIE | 945 | 702 (74.29) | 10 | 46 | 233 | 601 | NA | NA | NA | NA | NA | NA | NA |
| **multivariable models** | | | | | | | | | | | | | | | |
| Ahmad et al (2023), India | fullPIERs model:  gestational age chest pain or dyspnea oxygen saturation (SpO2) platelet count serum creatinine serum AST probability cut-off < 1 | prematurity  Apgar score < 4 at the time of birth Meconium-stained liquor NICU admission neonatal death intrauterine death | 377 | 164 (43.50) | 44 | 4 | 169 | 160 | NA | NA | NA | NA | NA | NA | NA |
| Ahmad et al (2023), India | fullPIERs model:  gestational age chest pain or dyspnea oxygen saturation (SpO2) platelet count serum creatinine serum AST probability cut-off > 1 | prematurity  Apgar score < 4 at the time of birth Meconium-stained liquor NICU admission neonatal death intrauterine death | 377 | 164 (43.50) | 169 | 160 | 44 | 4 | NA | NA | NA | NA | NA | NA | NA |
| Ahmad et al (2023), India | fullPIERs model:  gestational age chest pain or dyspnea oxygen saturation (SpO2) platelet count serum creatinine serum AST probability cut-off > 2.4 | prematurity  Apgar score < 4 at the time of birth Meconium-stained liquor NICU admission neonatal death intrauterine death | 377 | 164 (43.50) | 117 | 139 | 96 | 25 | NA | NA | NA | NA | NA | NA | NA |
| Ahmad et al (2023), India | fullPIERs model:  gestational age chest pain or dyspnea oxygen saturation (SpO2) platelet count serum creatinine serum AST probability cut-off > 4.9 | prematurity  Apgar score < 4 at the time of birth Meconium-stained liquor NICU admission neonatal death intrauterine death | 377 | 164 (43.50) | 47 | 114 | 166 | 50 | NA | NA | NA | NA | NA | NA | NA |
| Ahmad et al (2023), India | fullPIERs model:  ggestational age chest pain or dyspnea oxygen saturation (SpO2) platelet count serum creatinine serum AST probability cut-off > 9.9 | prematurity  Apgar score < 4 at the time of birth Meconium-stained liquor NICU admission neonatal death intrauterine death | 377 | 164 (43.50) | 29 | 84 | 184 | 80 | NA | NA | NA | NA | NA | NA | NA |
| Ahmad et al (2023), India | fullPIERs model:  gestational age chest pain or dyspnea oxygen saturation (SpO2) platelet count serum creatinine serum AST probability cut-off > 19.9 | prematurity  Apgar score < 4 at the time of birth Meconium-stained liquor NICU admission neonatal death intrauterine death | 377 | 164 (43.50) | 17 | 69 | 196 | 95 | NA | NA | NA | NA | NA | NA | NA |
| Ahmad et al (2023), India | fullPIERs model:  gestational age chest pain or dyspnea oxygen saturation (SpO2) platelet count serum creatinine serum AST probability cut-off ≥ 30 | prematurity  Apgar score < 4 at the time of birth Meconium-stained liquor NICU admission neonatal death intrauterine death | 377 | 164 (43.50) | 16 | 59 | 197 | 105 | NA | NA | NA | NA | NA | NA | NA |
| Peguero et al (2023), Spain | PREP-L model: maternal age maternal medical conditions (pre-existing chronic hypertension, renal disease, diabetes mellitus, autoimmune disease and/or previous history of preeclampsia) SBP urine protein/creatinine ratio serum urea concentration  platelet count gestational age need for antihypertensive treatment or magnesium sulfate | stillbirth neonatal death  neonatal metabolic acidosis  5 min Apgar score < 7 BDP  NEC grade III or IV IVH cystic periventricular leukomalacia stage 3–5 ROP HIE acute renal failure (serum creatinine >1.5 mg/dL) cardiac failure (requiring inotropic support) | 63 | 18 (28.57) | NA | NA | NA | NA | NA | NA | NA | NA | NA | NA | 0.69 (0.51-0.86) |
| Peguero et al (2023), Spain | PREP-L model: maternal age maternal medical conditions (pre-existing chronic hypertension, renal disease, diabetes mellitus, autoimmune disease and/or previous history of preeclampsia) SBP urine protein/creatinine ratio serum urea concentration  platelet count gestational age need for antihypertensive treatment or magnesium sulfate  severe FGR | stillbirth neonatal death  neonatal metabolic acidosis  5 min Apgar score < 7 BDP  NEC grade III or IV IVH cystic periventricular leukomalacia stage 3–5 ROP HIE acute renal failure (serum creatinine >1.5 mg/dL) cardiac failure (requiring inotropic support) | 63 | 18 (28.57) | NA | NA | NA | NA | NA | NA | NA | NA | NA | NA | 0.90 (0.82-0.98) |
| Peguero et al (2023), Spain | PREP-L model: maternal age maternal medical conditions (pre-existing chronic hypertension, renal disease, diabetes mellitus, autoimmune disease and/or previous history of preeclampsia) SBP urine protein/creatinine ratio serum urea concentration  platelet count gestational age need for antihypertensive treatment or magnesium sulfate  low PlGF | stillbirth neonatal death  neonatal metabolic acidosis  5 min Apgar score < 7 BDP  NEC grade III or IV IVH cystic periventricular leukomalacia stage 3–5 ROP HIE acute renal failure (serum creatinine >1.5 mg/dL) cardiac failure (requiring inotropic support) | 63 | 18 (28.57) | NA | NA | NA | NA | NA | NA | NA | NA | NA | NA | 0.91 (0.84-0.98) |
| Peguero et al (2023), Spain | PREP-L model: maternal age maternal medical conditions (pre-existing chronic hypertension, renal disease, diabetes mellitus, autoimmune disease and/or previous history of preeclampsia) SBP urine protein/creatinine ratio serum urea concentration  platelet count gestational age need for antihypertensive treatment or magnesium sulfate  severe FGR low PlGF | stillbirth neonatal death  neonatal metabolic acidosis  5 min Apgar score < 7 BDP  NEC grade III or IV IVH cystic periventricular leukomalacia stage 3–5 ROP HIE acute renal failure (serum creatinine >1.5 mg/dL) cardiac failure (requiring inotropic support) | 63 | 18 (28.57) | NA | NA | NA | NA | NA | NA | NA | NA | NA | NA | 0.91 (0.81-0.98) |
| Reddy et al (2022), Australia | UtA PI  EFW percentile  MCA PI percentile  serum PlGF | preterm birth (< 34 weeks) NICU admission  RDS IVH HIE NEC confirmed fetal infection ROB | 126 | 48 (38.10) | NA | NA | NA | NA | 0.63 (0.33-0.81) | NA | NA | NA | NA | NA | 0.88 (0.82 – 0.94) |
| Graupner et al (2019), Germany | sFlt/PlGF ratio CPR or mUtA PI | pH value umbilical cord artery ≤ 7.0 and/or base excess ≤ –12 mmol/L 5 min Apgar score ≤ 7 NICU admission RDS  respiratory support > 4 h SGA | 67 | 12 (17.90) | NA | NA | NA | NA | NA | NA | NA | NA | NA | NA | 0.606 (0.44–0.78) |
| Saxena et al (2021), India | CPR < 1 EFW < 2.5kg | cesarean section for fetal distress  Meconium stained Liquor 5 min Apgar score < 7 perinatal death NICU admission | 150 | NA (NA) | NA | NA | NA | NA | 1(0.86-1) | 0.33 (0.25-0.42) | 0.23 (0.15-0.32) | 1 (0.91-1) | NA | NA | NA |
| Wu et al (2017), China | serum gamma glutamyl transferase UA S/D ratio | perinatal death admission to the NICU  5 min Apgar score < 8 | 120 | 36 (30.00) | NA | NA | NA | NA | 0.94 | 0.8 | NA | NA | NA | NA | 0.87 |
| Hong et al (2021), China | B–type natriuretic peptide age hemoglobin BMI proteinuria | SGA fetal distress fetal death stillbirth oligohydramnios, and  neonatal asphyxia (1 min Apgar scores <7 and umbilical blood pH <7.20) | 284 | 76 (26.80) | NA | NA | NA | NA | NA | NA | NA | NA | NA | NA | 0.792 (0.740–0.838) |
| Hong et al (2021), China | age hemoglobin BMI proteinuria | SGA fetal distress fetal death stillbirth oligohydramnios, and  neonatal asphyxia (1 min Apgar scores <7 and umbilical blood pH <7.20) | 284 | 76 (26.80) | NA | NA | NA | NA | NA | NA | NA | NA | NA | NA | 0.76 |
| Magee et al (2016), multinational | less tight (100 mmHg) controlled DBP (vs. tight (85 mmHg) controlled DBP) BMI (≥ 25 vs <25) ART nulliparous folic acid or PNV in hospital SBP within last week DBP within last week Methyldopa (+others, no labetalol) other antihypertensive drugs no antihypertensive drugs | pregnancy loss NICU admission | 987 | 307 (31.10) | NA | NA | NA | NA | NA | NA | NA | NA | NA | NA | 0.65 (0.61. 0.69) |
| Elia et al (2017), UK | log transformed ACR gestational age at ACR measurement essential hypertension preexisting diabetes gestational diabetes social deprivation index BMI  MAP current smoking status parity maternal age | preterm delivery (< 34 weeks) low birth weight  abnormal umbilical artery Doppler  arterial cord pH < 7.1 need for ventilation neonatal or intrauterine death | 717 | 146 (20.36) | NA | NA | NA | NA | NA | NA | NA | NA | NA | NA | 0.718 (0.668–0.760) |
| Chen et al (2022), China | multivariable model based on boosted tree method | preterm birth fetal growth restriction  SGA  NICU admission low Apgar scores < 7 neonatal death | 1829 | 306 (16.73) | NA | NA | NA | NA | 0.62 (0.56-0.68) | 0.97 (0.97-0.98) | NA | NA | NA | NA | NA |
| Zheng et al (2022), China | multivariable model based on random forest classifier with imputation | stillbirth preterm low birth weight SGA neonatal death neonatal seizures NICU admission  respiratory support | 733 | 423 (57.71) | NA | NA | NA | NA | 0.94 | 0.88 | 0.92 | 0.91 | NA | NA | 0.97 |
| **Abbreviations:** ACR, creatinine/albumin ratio; AEDV, absent end-diastolic velocity; ART, assisted reproductive technology; AST, aspartate transaminase; AUROC, area under the receiver operating characteristic curve; BDP, bronchopulmonary dysplasia; BMI, Body mass index; CPR, cerebroplacental ratio; CTG, cardiotocograph; CPAP, continuous positive airway pressure; DBP, diastolic blood pressure; DV, ductus venosus; EFW, estimated fetal weight; FGR, fetal growth restriction; FN, false negative; FP, false positive; HIE, hypoxic-ischemic encephalopathy; hs-CRP, high-sensitive C-reactive protein; IUGR, intrauterine growth restriction; IVH, intraventricular haemorrhage; LR-, negative likelihood ratio; LR+, positive likelihood ratio; MAP, mean arterial pressure; MCA, middle cerebral artery; NA, not available; NEC, necrotizing enterocolitis; NICU, neonatal intensive care unit; NPV, negative predictive value; OA, ophthalmic artery; PDA, patent ductus arteriosus; PI, pulsatility index; PlGF, placental growth factor; PMR, perinatal mortality ratio; PNV, prenatal vitamin; PPV, positive predictive value; PR, peak ratio; PSV, peak systolic velocity; PVIV, peak velocity index; RDS, respiratory distress syndrome; REDV, reduced end-diastolic velocity; RI, resistance index; ROP, retinopathy of prematurity; S/D, systolic/diastolic ratio; SBP, systolic blood pressure; sFlt-1, soluble fms-like tyrosine kinase-1; SGA, small for gestational age; TN, true negative; TP, true positive; UA, umbilical artery; UPCR, urinary protein to creatinine ratio; UtA, uterine artery. | | | | | | | | | | | | | | | |

**Supplementary figure 1**

**A**

**B**

Forrest plot of the meta-analysis of the fullPIERS model. Pooled Area under the Curve (AUROC) value of the prediction performance of the fullPIERS model for composite PIERS outcome within 48 hours in six cohorts from eight studies (**A**), within 7 days in two cohorts from five studies (**B**). Studies are divided if study was conducted in either high-income countries or low-and middle-income countries. Subgroup meta-analysis (red) and overall meta-analysis (green) has been performed. CI, confidence interval; p, p-value; PREP, Prediction of Complications in Early-Onset Preeclampsia.

**Supplementary figure 2**

**A**

**B**

**C**

**D**

Forrest plot of the meta-analysis of the prediction of perinatal outcomes. Pooled Area under the Curve (AUROC) value of the prediction performance of the Uterine artery pulsatility index (UtA PI) > 95th percentile for fetal growth restriction (FGR) (A) and respiratory distress syndrome (RDS) (B). Pooled AUROC value of the prediction performance of sFlt-1/PlGF ratio > 85 for perinatal death (C) and neonatal intensive care unit (NICU) admission (D). CI, confidence interval; p, p-value;

**References relating to excluded studies**

1. Agrawal S, Maitra N. Prediction of Adverse Maternal Outcomes in Preeclampsia Using a Risk Prediction Model. *Journal of Obstetrics and Gynecology of India* 2016; **66**: 104-11.

2. Al Khalaf SY, O'Reilly ÉJ, McCarthy FP, Kublickas M, Kublickiene K, Khashan AS. Pregnancy outcomes in women with chronic kidney disease and chronic hypertension: a National cohort study. *Am J Obstet Gynecol* 2021; **225**(3): 298.e1-.e20.

3. Al Khalaf SY, Khashan AS, McCarthy FP, O'Reilly EJ. Prepregnancy hypertension and the risk of twelve cardiovascular diseases: the role of adverse pregnancy outcomes. *Am J Obstet Gynecol* 2023; **228**(1): S307.

4. Álvarez-Fernández I, Prieto B, Rodríguez V, Ruano Y, Escudero AI, Álvarez FV. N-terminal pro B-type natriuretic peptide and angiogenic biomarkers in the prognosis of adverse outcomes in women with suspected preeclampsia. *Clin Chim Acta* 2016; **463**: 150-7.

5. Ashwal E, Ali-Gami J, Aviram A, et al. Contribution of Second Trimester Sonographic Placental Morphology to Uterine Artery Doppler in the Prediction of Placenta-Mediated Pregnancy Complications. *J* 2022; **11**(22): 15.

6. Balyan K, Humtso BY, Meena B, Sapna S, Rana A, Kumar M. Materno-fetal outcome with PlGF above or below cutoff during second half of pregnancy in high-risk women. *International Journal of Gynecology and Obstetrics* 2023.

7. Bian X, Biswas A, Huang X, et al. Short-Term Prediction of Adverse Outcomes Using the sFlt-1 (Soluble fms-Like Tyrosine Kinase 1)/PlGF (Placental Growth Factor) Ratio in Asian Women With Suspected Preeclampsia. *Hypertension* 2019; **74**(1): 164-72.

8. Boutin A, Gasse C, Guerby P, Giguère Y, Tétu A, Bujold E. First-Trimester Preterm Preeclampsia Screening in Nulliparous Women: The Great Obstetrical Syndrome (GOS) Study. *Journal of Obstetrics and Gynaecology Canada* 2021; **43**(1): 43-9.

9. Duhig KE, Myers J, Seed PT, et al. Placental growth factor testing to assess women with suspected pre-eclampsia: a multicentre, pragmatic, stepped-wedge cluster-randomised controlled trial. *The Lancet* 2019; **393**(10183): 1807-18.

10. Elasy AN, Nafea OE. Critical Hypermagnesemia in Preeclamptic Women Under a Magnesium Sulfate Regimen: Incidence and Associated Risk Factors. *Biol Trace Elem Res* 2022.

11. Escouto DC, Green A, Kurlak L, et al. Postpartum evaluation of cardiovascular disease risk for women with pregnancies complicated by hypertension. *Pregnancy Hypertens* 2018; **13**: 218-24.

12. Gaccioli F, Sovio U, Cook E, Hund M, Charnock-Jones DS, Smith GCS. Screening for fetal growth restriction using ultrasound and the sFLT1/PlGF ratio in nulliparous women: a prospective cohort study. *The Lancet Child and Adolescent Health* 2018; **2**(8): 569-81.

13. Haritha M, Nilofer, Gaddipati J, Gogineni S, Gayathri KB. Screening of High-Risk Pregnancies by First and Second Trimester Uterine Artery Doppler for Improving Sensitivity in Prediction of Adverse Pregnancy Outcome. *European Journal of Molecular and Clinical Medicine* 2022; **9**(3): 1961-6.

14. Herraiz I, Simón E, Gómez-Arriaga PI, et al. Clinical implementation of the sFlt-1/PlGF ratio to identify preeclampsia and fetal growth restriction: A prospective cohort study. *Pregnancy Hypertens* 2018; **13**: 279-85.

15. Hopkins MK, Levine LD, Koelper NC, Durnwald C. Screening Echocardiogram in High-Risk Women with Class III Obesity to Predict the Risk of Preeclampsia. *Am J Perinatol* 2021.

16. Hughes RCE, Phillips I, Florkowski CM, Gullam J. The predictive value of the sFlt-1/PlGF ratio in suspected preeclampsia in a New Zealand population: A prospective cohort study. *Aust N Z J Obstet Gynaecol* 2022.

17. Hughes RCE, Phillips I, Florkowski CM, Gullam J. The predictive value of the sFlt-1/PlGF ratio in suspected preeclampsia in a New Zealand population: A prospective cohort study. *Aust N Z J Obstet Gynaecol* 2023; **63**(1): 34-41.

18. Jha S, Singh S. Role of cardiotocography in predicting perinatal outcome in high-risk pregnancy. *Annals of Clinical and Analytical Medicine* 2021; **12**(3): 313-6.

19. Lin W, Teng SW, Lin TY, et al. Combinatorial Analysis of Circulating Biomarkers and Maternal Characteristics for Preeclampsia Prediction in the First and Third Trimesters in Asia. *Diagnostics (Basel)* 2022; **12**(7): 23.

20. Lin X, Liu LL, Zheng LJ, Yang CY. Evaluation of Doppler indices (MCA & UA) and fetal outcomes: a retrospective case-control study in women with hypertensive disorders of pregnancy. *Journal of Maternal-Fetal and Neonatal Medicine* 2023; **36**(1).

21. Melo DCS, Sousa RP, Pais MSJDR, Felix LM, Pinto FF, Moura JPSA. The role of the soluble fms-like tyrosine kinase-1/placental growth factor (sFlt-1/PIGF) - Ratio in clinical practice in obstetrics: Diagnostic and prognostic value. *J Perinat Med* 2023; **51**(7): 896-903.

22. Nan MN, Garcia-Osuna A, Mora J, et al. Impact of Angiogenic and Cardiovascular Biomarkers for Prediction of Placental Dysfunction in the First Trimester of Pregnancy. *Biomedicines* 2023; **11**(5).

23. Naz T, Khalid M, Nawaz R, Pervaiz MJ, Arshad I. Association of maternal and perinatal mortality with hyperuricemia in females presenting with pre-eclampsia. *Pakistan Journal of Medical and Health Sciences* 2021; **15**(4 April): 714-6.

24. Neuman RI, Alblas van der Meer MM, Nieboer D, et al. PAPP-A2 and Inhibin A as Novel Predictors for Pregnancy Complications in Women With Suspected or Confirmed Preeclampsia. *Journal of the American Heart Association* 2020; **9**(19): e018219.

25. Neuman RI, van der Meer MMA, Saleh L, et al. Copeptin and mid-regional pro-atrial natriuretic peptide in women with suspected or confirmed pre-eclampsia: comparison with sFlt-1/PlGF ratio. *Ultrasound in obstetrics & gynecology : the official journal of the International Society of Ultrasound in Obstetrics and Gynecology* 2020; **56**(6): 872-8.

26. Nila SG, Bobby Z, Dorairajan G, Jacob SE. Diagnostic ability of hepcidin in predicting fetal outcome in preeclampsia. *Journal of Maternal-Fetal and Neonatal Medicine* 2021; **34**(22): 3678-83.

27. Ohkuchi A, Saito S, Yamamoto T, et al. Short-term prediction of preeclampsia using the sFlt-1/PlGF ratio: a subanalysis of pregnant Japanese women from the PROGNOSIS Asia study. *Hypertens Res* 2021; **44**(7): 813-21.

28. Oztas E, Ozler S, Ersoy AO, et al. Increased levels of serum clusterin is associated with intrauterine growth restriction and adverse pregnancy outcomes in preeclampsia. *J Perinat Med* 2016; **44**(3): 269-75.

29. Parchem JG, Brock CO, Chen HY, Kalluri R, Barton JR, Sibai BM. Placental Growth Factor and the Risk of Adverse Neonatal and Maternal Outcomes. *Obstet Gynecol* 2020; **135**(3): 665-73.

30. Rana S, Salahuddin S, Mueller A, Berg AH, Thadhani RI, Karumanchi SA. Angiogenic biomarkers in triage and risk for preeclampsia with severe features. *Pregnancy Hypertens* 2018; **13**: 100-6.

31. Rani S, Huria A, Kaur R. Prediction of perinatal outcome in preeclampsia using middle cerebral artery and umbilical artery pulsatility and resistance indices. *Hypertens Pregnancy* 2016; **35**(2): 210-6.

32. Saleh L, Verdonk K, Jan Danser AH, et al. The sFlt-1/PlGF ratio associates with prolongation and adverse outcome of pregnancy in women with (suspected) preeclampsia: analysis of a high-risk cohort. *Eur J Obstet Gynecol Reprod Biol* 2016; **199**: 121-6.

33. Saleh L, Vergouwe Y, van den Meiracker AH, et al. Angiogenic Markers Predict Pregnancy Complications and Prolongation in Preeclampsia: Continuous Versus Cutoff Values. *Hypertension* 2017; **70**(5): 1025-33.

34. Saleh L, Tahitu SIM, Danser AHJ, van den Meiracker AH, Visser W. The predictive value of the sFlt-1/PlGF ratio on short-term absence of preeclampsia and maternal and fetal or neonatal complications in twin pregnancies. *Pregnancy Hypertens* 2018; **14**: 222-7.

35. Sharp A, Chappell LC, Dekker G, et al. Placental Growth Factor informed management of suspected pre-eclampsia or fetal growth restriction: The MAPPLE cohort study. *Pregnancy Hypertens* 2018; **14**: 228-33.

36. Singh S, Mohakud S, Naik S, Jena SK, Sethi P, Nayak P. Predictors of posterior reversible encephalopathy syndrome in women with hypertension in pregnancy: A prospective observational study. *Pregnancy Hypertens* 2021; **23**: 191-5.

37. Suresh S, Patel E, Mueller A, et al. The additive role of angiogenic markers for women with confirmed preeclampsia. *Am J Obstet Gynecol* 2023; **228**(5): 573.e1-.e11.

38. Teefey CP, Durnwald CP, Srinivas SK, Levine LD. Adverse Maternal Outcomes Differ between Obese and Nonobese Women with Severe Preeclampsia. *Am J Perinatol* 2019; **36**(1): 74-8.

39. Zhang Y, Tan X, Yu F. The diagnostic and predictive values of N-terminal pro-B-type natriuretic peptides in pregnancy complications and neonatal outcomes. *American Journal of Translational Research* 2021; **13**(9): 10372-9.

40. Ajmi H, Abid D, Milouchi S, et al. Interest of speckle tracking in the detection of cardiac involvement in pregnant women with hypertensive disorder. *Pregnancy Hypertens* 2018; **11**: 136-41.

41. Awan R, Bushra N, Rizwan W, et al. Diagnostic Accuracy of Modified Biophysical Profile in Predicting Poor Perinatal Outcome in Pregnancies Complicated by Hypertension at Term. *Pakistan Journal of Medical and Health Sciences* 2022; **16**(7): 166-8.
[truncated: 35,869 more chars]
